# Leigh Syndrome in *Drosophila melanogaster*: MORPHOLOGICAL AND BIOCHEMICAL CHARACTERIZATION OF Surf1 POST-TRANSCRIPTIONAL SILENCING*[Fn FN2]

**DOI:** 10.1074/jbc.M114.602938

**Published:** 2014-08-27

**Authors:** Caterina Da-Rè, Sophia von Stockum, Alberto Biscontin, Caterina Millino, Paola Cisotto, Mauro A. Zordan, Massimo Zeviani, Paolo Bernardi, Cristiano De Pittà, Rodolfo Costa

**Affiliations:** From the Departments of ‡ Biology and; § Biomedical Sciences and; ¶ CRIBI Biotechnology Centre, University of Padova, 35121 Padova, Italy and; ‖ MRC Mitochondrial Biology Unit, University of Cambridge, Cambridge CB2 0XY, United Kingdom

**Keywords:** Cytochrome c, Cytochrome *c* oxidase (Complex IV), *Drosophila*, Mitochondria, Mitochondrial Disease, Mitochondrial Respiratory Chain Complex

## Abstract

Leigh Syndrome (LS) is the most common early-onset, progressive mitochondrial encephalopathy usually leading to early death. The single most prevalent cause of LS is occurrence of mutations in the *SURF1* gene, and LS*^Surf1^* patients show a ubiquitous and specific decrease in the activity of mitochondrial respiratory chain complex IV (cytochrome *c* oxidase, COX). *SURF1* encodes an inner membrane mitochondrial protein involved in COX assembly. We established a *Drosophila melanogaster* model of LS based on the post-transcriptional silencing of *CG9943*, the *Drosophila* homolog of *SURF1*. Knockdown of *Surf1* was induced ubiquitously in larvae and adults, which led to lethality; in the mesodermal derivatives, which led to pupal lethality; or in the central nervous system, which allowed survival. A biochemical characterization was carried out in knockdown individuals, which revealed that larvae unexpectedly displayed defects in all complexes of the mitochondrial respiratory chain and in the F-ATP synthase, while adults had a COX-selective impairment. Silencing of *Surf1* expression in *Drosophila* S2R^+^ cells led to selective loss of COX activity associated with decreased oxygen consumption and respiratory reserve. We conclude that *Surf1* is essential for COX activity and mitochondrial function in *D. melanogaster*, thus providing a new tool that may help clarify the pathogenic mechanisms of LS.

## Teaser

**Background:** Mutations in *SURF1*, a human gene important for the assembly of cytochrome-c-oxidase (COX) causes Leigh Syndrome, a mitochondrial encephalopathy.

**Results:** Knockdown of *Surf1* in *Drosophila* matches the biochemical features of the human disease.

**Conclusion:** In *Drosophila* SURF1 is essential to maintain mitochondrial function and its silencing leads to COX deficiency.

**Significance:** *Drosophila* offers new tools to investigate the pathogenic mechanism of Leigh Syndrome.

## Introduction

Mitochondrial oxidative phosphorylation (OXPHOS)^3^ is mediated by the concerted activity of the mitochondrial respiratory chain (MRC), which is composed of four respiratory complexes catalyzing electron transport, and of the F-ATP synthase, also called complex V. The subunits of the MRC and of F-ATP synthase are mostly encoded by nuclear DNA (nDNA), but a small subset is specified by mitochondrial DNA (mtDNA) (1). The biogenesis of OXPHOS complexes requires the correct assembly of both mtDNA- and nDNA-encoded subunits. mtDNA-encoded subunits form the hydrophobic core of the complexes, and their cotranslational insertion into the inner mitochondrial membrane is the first event in MRC and F-ATP synthase biogenesis (2). This process is complex and precisely organized, and requires the sequential interaction of many factors (3, 4). Complex I is formed by many subunits and its assembly takes place by their sequential insertion (5–7). Recently the assembly of complex III was proposed to be a sequential process starting from two subcomplexes of cytochrome *b*, Qcr7p and Qcr8p (8). Complex IV (cytochrome *c* oxidase, COX) dimerization is important for its activity, and the assembly of the fully dimeric complex occurs by means of three subcomplexes (9). Once individual complexes are formed, they integrate into “supercomplexes” (10) which enable more efficient electron flow and promote complex stability (11). These large macromolecular assemblies contain combinations of complex I, dimeric complex III, and COX (12, 13). COX is the terminal enzyme of the electron transfer chain, which couples electron transfer to molecular oxygen and proton pumping across the inner membrane (14). Mammalian COX is composed of 14 subunits, only 3 of which (COX1, COX2, and COX3) are encoded by mtDNA and constitute the hydrophobic core of the enzyme (15) that is assembled first, followed by insertion of the 11 nDNA-encoded subunits (9). A number of accessory factors is necessary for the formation of the active holoenzyme (16). Assembly of COX is a multi-step process involving several factors and chaperones, including COX10−11, COX15–20, COX23, SCO1, SCO2, and SURF1 (17). The importance of the correct assembly of the MRC complexes, and specifically of COX, is highlighted by mitochondrial diseases arising because of inadequate assembly/stability (9). Mutations of human *SURF1*, encoding a highly conserved protein located in the inner mitochondrial membrane (18–25), cause COX-defective Leigh syndrome (LS^COX^, OMIM 256000) (26, 27). SURF1 promotes COX biogenesis through association with different protein modules, and its loss of function leads to COX deficiency and reduced expression of the central subunit COX1 (28–30). LS^COX^ is an early-onset syndrome characterized by the accumulation of symmetric necrotizing lesions in subcortical areas of the central nervous system (CNS) that include the brainstem, cerebellum, diencephalon, and corpus striatum (31, 32). This fatal neurological disorder has a relentlessly progressive course, death usually occurring within the first decade of life, although protracted cases are known. Absence of SURF1 in mice caused mild COX deficiency, however, and hardly any clinical or pathological signs (22, 33).

The molecular mechanisms leading to COX deficiency and neurodegeneration in LS^COX^ are still poorly understood, also because of the lack of an adequate animal model of the disease. To shed light on this matter, a *Surf1*-specific knockdown (KD) model in *Drosophila melanogaster* was previously created by transgenic double-stranded RNA interference (34). Here we have explored the biochemical and morphological features of *Surf1*-specific KD in *D. melanogaster*. The GAL4/UAS binary system (35) was exploited to drive post-transcriptional silencing of the *Surf1* (*CG9943*) gene under the control of 3 promoters *i.e. Actin5C*, an early-expressed housekeeping gene; *elav*, an early-expressed neuron-specific gene (36); and *how^24B^,* a gene expressed in all embryonic and larval somatic muscle, which is required during embryogenesis, in the late stages of somatic muscle development, and during metamorphosis for muscle reorganization (37, 38). Our results demonstrate that lack of *Surf1* in *Drosophila* largely phenocopies the human disease, and provides a novel model to address pathogenesis of LS^COX^.

## EXPERIMENTAL PROCEDURES

### 

#### 

##### Fly Stocks and Breeding Conditions

Flies were raised on standard cornmeal medium and were maintained at 23 °C, 70% relative humidity, on a 12 h light: 12 h dark cycle. The *UAS* fly strain used to perform post-transcriptional silencing, carrying single *UAS-Surf1-IR* autosomal insertion, was obtained as previously described (34). The following Gal4 lines, obtained from the Bloomington Stock Center, were used to drive the expression of the *UAS-Surf1-IR* construct: [*y, w; Act-Gal4/TM6B, Tb*], [*elav-C155-Gal4*], [*y, w; Switch-Act-Gal4/TM6B, Tb*], [*y, w; how^24B^Gal4*]. Wild type [*w^1118^*] and [*y, w; UAS-GFP*] strains were used as controls.

##### Egg-to-Adult Viability

For each of the transgenic lines about 300 fertilized eggs were collected on standard yeast-glucose-agar medium in a Petri dish (60 × 15 mm). The fertilized eggs were incubated at 23 °C, and for each experimental condition the number of individuals reaching the 3rd instar larva, pupa, or adult, and the relative percentages were calculated (34).

##### Cell Cultures

The *Drosophila* S2R^+^ cell line is derived from a primary culture of late stage (20–24 h old) *D. melanogaster* embryos (39). It was obtained from *Drosophila* Genomics Resource Center (DGRC). S2R^+^ cells grow at 25 °C without CO_2_ in Schneider's medium (Invitrogen) with 10% heat-inactivated fetal bovine serum (FBS) (Sigma-Aldrich) as a loose, semi-adherent monolayer, showing a doubling time of about 48 h.

##### dsRNA Production and RNAi Procedures

dsRNAi synthesis was performed employing the T7 Megascript kit (Invitrogen) (40, 41). The oligonucleotides primers used to synthesize dsRNA starting from cDNA were *Surf1_*T7 F (agtattcccaatcaggcggctgtatgcgttttgccaaagttaaa) and R (aggtcaccaggtacgacagaatggtacaccctctcgatgcactg). These primers give two complementary 600 bp RNA products that anneal as temperature decreases, forming a final 600 bp dsRNA. About 2 × 10^6^ cells suspended in 1 ml of serum-free medium were mixed with 2 μg/ml dsRNA, plated in a 24-well plate, and incubated at room temperature for 1 h. Subsequently, one volume of complete medium 2× was added, and cells were grown in the presence of dsRNA for 2 days at 25 °C.

##### Primers

All oligonucleotides used in this work were designed using the on-line tool Primer-BLAST (42).

##### Isolation of Mitochondria

Mitochondria were prepared by differential centrifugation from ∼200 flies or larvae. Samples were homogenized with a Dounce glass potter and a loose-fitting glass pestle. A mannitol-sucrose buffer (pH 7.4) in 2% BSA was then added to a final volume of 25 ml. Samples were centrifuged at 1,500 × *g* (Beckman Avanti J-25 Centrifuge, Beckman 2550 rotor) at 4 °C for 6 min. The supernatant was filtered through a fine mesh, and centrifuged at 7,000 × *g* at 4 °C for 6 min. The mitochondrial pellet was resuspended in 20 ml of mannitol-sucrose buffer without BSA, centrifuged again at 7,000 × *g* and finally resuspended in 50 μl of buffer. Protein concentration was measured by the Biuret test. Prior to enzymatic MRC complex activity assays, samples were subjected to 3 freeze-thaw cycles using liquid nitrogen to disrupt the mitochondrial membranes.

##### Enzymatic Analysis

The activities of OXPHOS complexes were measured as described previously (43). Citrate synthase (CS) activity was determined by the reduction of dithio-bis-nitrobenzoic acid (DTNB) followed at 412 nm (extinction coefficient = 21,000 m^−1^/cm^−1^) in a reaction buffer containing 5 μg of mitochondrial proteins, 75 mm Tris-HCl, Triton 0,1%, 0.4 mm acetyl-CoA, 0,1 μm DTNB, and 0.5 mm oxaloacetate (pH 8.0). Complexes I-V activities were normalized to the activity of CS, both measured as nmol min^−1^ mg^−1^ and then expressed as a percentage (44).

##### RNA Isolation and qRT-PCR Experiments

Total RNA was extracted from ∼10 larvae or 2 × 10^6^ cells using Trizol (Invitrogen) and further purified by precipitation with LiCl 8 m. RNA samples were checked for integrity by capillary electrophoresis (RNA 6000Nano LabChip, Agilent Technologies). For each sample, 1 μg of RNA was used for first-strand cDNA synthesis, employing 10 mm deoxynucleotides, 10 μm oligo-dT and SuperScript II (Invitrogen). qRT-PCRs were performed in triplicate in a 7500 Real-Time PCR System (Invitrogen) using SYBER Green chemistry (Promega). The 2^−ΔΔCt^ (RQ, relative quantification) method implemented in the 7500 Real Time PCR System software was used to calculate the relative expression ratio (45). The *Surf1* oligonucleotides used were *Surf1*-RT F (caaatggagtaccgcctggt) and R (gcgagaagagtcctccttgg). *Rp49* was used as endogenous control, and the oligonucleotides employed were *Rp49* F (tcggttacggatcgaacaa) and R (gacaatctccttgcgcttct).

##### Determination of mtDNA Levels

Total DNA from larvae was extracted using phenol/chloroform precipitation. The amount of mtDNA was assessed by the ratio of mtDNA to nDNA copy number determined by quantitative real time amplification of the mitochondrial 16 S gene and the nuclear *Rpl32* gene. Primers used in this work (16 S F and R; *Rpl32* F and R) were those reported previously (46). We generated two gene-specific calibration curves with six 10-fold serial dilutions (100–10,000,000 copies) of plasmids containing the cloned target sequences (Invitrogen). Concentration of plasmid stock solutions was assessed with an ND-1000 spectrophotom- eter (NanoDrop), and the plasmid copy number of dilutions was calculated using Avogadro's number. Reactions were per- formed in triplicate using SYBR Green chemistry according to the manufacturer's recommendations (GoTaq qPCR Master Mix, Promega) in a 7500 Real Time PCR System instrument (Invitrogen). Data were normalized to the ratio of mtDNA/nDNA copy number in controls (arbitrary set to 100%) (47, 48).

##### Whole Mount Larval Body Wall Preparations

Third instar larvae were dissected in Ca^2+^-free hemolymph-like saline-3 buffer (49) and pinned on the silicone-coated surface (Sylgard 184; Dow Corning) of a 35-mm Petri dish using fine insect forceps, scissors, and pins (FST, Germany) (50). Specimens were analyzed with a Leica DMR microscope using 403 DIC optics, and images were taken with a Leica DFC 480 digital camera.

##### Measurements of Oxygen Consumption

Oxygen measurements were made using the XF24 Extracellular Flux Analyzer (Seahorse Bioscience) at 25 °C. S2R^+^ *Drosophila* cells were seeded onto XF-24-well plates at 20,000 cells/well and cultured for 48 h (51). The following day, the culture medium was replaced with serum-free Schneider medium (Invitrogen). Basal oxygen consumption rates, reported in picomoles per minute, were measured before injecting the first drug to be tested. Chemicals were sequentially added in each well as described in the figure legends.

##### Mitochondrial Membrane Potential and Ca^2+^ Fluxes

Mitochondrial membrane potential of isolated wild type or *Surf1* KD adult mitochondria was measured based on the fluorescence quenching of Rhodamine 123 (52) and mitochondrial Ca^2+^ fluxes were measured by Calcium Green-5N (Molecular Probes) fluorescence (52) at 25 °C using a Fluoroskan Ascent FL (Thermo Electron) plate reader (excitation and emission wavelengths of 485 and 538 nm, respectively with a 10-nm bandpass filter) at a mitochondrial concentration of 1 mg × ml^−1^. The incubation medium contained 250 mm Sucrose, 10 mm MOPS-Tris, 5 mm Pi-Tris, 10 μm EGTA, and 0.4 μm Rhodamine 123, or 0.5 μm Calcium-Green 5N, pH 7.4. The membrane potential was generated either through proton pumping by the respiratory chain by supplementing the assay medium with a respiratory substrate (5 mm glutamate plus 2.5 mm malate) or by ATP hydrolysis by supplementing the assay medium with an ATP-regenerating system providing constant [ATP] (4 mm MgCl_2_, 2 mm phosphocreatine, 0.4 mm ATP, and 1.5 units/ml creatine kinase). Further additions were made as indicated in the figure legends.

##### Electron Microscopy

For transmission electron microscopy, 3rd instar larvae were pierced and immediately transferred to ice-cold fixation solution containing 3% paraformaldehyde, 2% glutaraldehyde, 100 mm sucrose, and 2 mm EGTA in 0.1 m sodium phosphate buffer at pH 7.2. Samples were fixed for 6 h and subsequently washed overnight at 4 °C in 0.1 m phosphate buffer, pH 7.2. The next day larvae were treated for 2 h with cold (4 °C) postfixative solution (1% OsO_4_ in 0.1 m sodium cacodylate buffer pH 7.2), rinsed three to four times (5 min each) in 0.1 m sodium phosphate buffer pH 7.2, and then dehydrated through ethanol series and finally by three 15-min washes in propylene oxide. Samples were embedded in Epon resin mixture (14 ml EPOXY, 7 ml DDSA, 9 ml MNA) to which 2% DMP30 solidification accelerator was added. The following gradual embedding procedure was utilized: three 45-min infiltrations in resin:propylene oxide, 1:2, 1:1, and 2:1, respectively; embedding in Epon within plastic capsules; and polymerization at 60 °C for 3 days. Ultrathin (400Å) cross sections of larval body-wall muscle were cut with a diamond knife and stained for 20 min in 2% aqueous uranyl acetate solution, followed by 30 s staining in 5% aqueous lead citrate solution, and finally rinsed in distilled water. Sections were examined and photographed with a Philips 200 electron microscope.

##### DNA Microarray Design

Probes were designed using the Agilent eArray Custom Microarray Design Service, which applies proprietary prediction algorithms to design 60mer oligo-probes. Microarrays were synthesized *in situ* using the Agilent ink-jet technology with 8 × 60 K format. A total of 32,162 probes representing *D. melanogaster* transcripts were successfully obtained. A custom microarray platform, named “Drosophila 1.0” (eArray Design ID: 035757), showed 30,814 duplicate probes and 1,348 single probes. Each array included default positive (1,011 probes) and negative (308 probes) controls. Probe sequences and other details on the microarray platform can be found in the Gene Expression Omnibus (GEO) database under accession number: GPL18767.

##### Microarray Labeling and Hybridization

Gene expression profiling was carried out on controls and *Surf1 Act-Gal4* KD first instar *Drosophila* larvae using the *Drosophila* 1.0 custom platform (Agilent Technologies). Total RNA was obtained from the whole body of 1st instar larvae for each genotype. Four biological replicates were analyzed for controls and *Surf1* KD samples, respectively for a total of 8 microarray experiments. 800 ng total RNA was labeled with “Agilent One-Color Microarray-Based Gene Expression protocol” according to the manufacturer's instructions. The synthesized cDNA was transcribed into cRNA and labeled with Cy3-dCTP. Labeled cRNA was purified with RNeasy Mini columns (Qiagen). The quality of each cRNA sample was verified by total yield and specificity calculated with NanoDrop ND-1000 spectrophotometer measurements. Labeled cRNA (1.65 μg) was used in each reaction, and hybridization was carried out at 65 °C for 17 h in a hybridization oven rotator (Agilent). The arrays were washed using Agilent Gene expression washing buffers and Stabilization and Drying Solution, as suggested by the supplier. Slides were scanned on an Agilent microarray scanner (model G2565CA), and Agilent Feature Extraction software version 10.5.1.1 was used for image analysis. Gene expression data are available in the GEO database with the accession number GSE58301.

##### Statistical Analysis of Gene Expression Data

Inter-array normalization of expression levels was performed with the quantile method (53) to correct for possible experimental distortions. A normalization function was applied to the expression data of all the experiments and the values of within-arrays replicate spots were then averaged. Feature Extraction Software, which provided spot quality measures, was used to evaluate the quality and reliability of the hybridization. In particular, the flag “glsPosAndSignif” (set to 1 if the spot had an intensity value significantly different from the local background and to 0 when otherwise) was used to filter out unreliable probes: the flag equal to 0 was noted as “not available (NA).” Probes with a high proportion of NA values were removed from the dataset in order to carry out a more solid, unbiased, statistical analysis. 50% NA was used as the threshold in the filtering process, and a total of 24,630 *Drosophila* transcripts were obtained. Principal Cluster analysis and profile similarity searches were performed with Multi Experiment Viewer version 4.8.1 (tMev) of the TM4 Microarray Software Suite. The identification of differentially expressed mRNAs was performed with *Li*near *M*odels for *M*icroarray Data *A*nalysis (LIMMA) program with default settings (*p* value <0.05) (54, 55). The normalized expression values of the biological replicates for each genotype were log2 transformed and mediated. Pathway analyses and network visualization of differentially expressed genes was performed using the Graphite web tool that combines topological and multivariate pathway analyses (56).

##### Mifepristone (RU-486)-induced Post-transcriptional Silencing

Crosses were raised at 20 °C on standard sucrose medium. Third instar larvae or freshly enclosed flies were transferred from RU-486-free food to the food containing RU-486 (the *Switch* promoter being activated by the chemical RU-486) and kept at a temperature of 20 °C. To this end, a 10 mm stock solution of RU-486 (mifepristone, Sigma) in ethanol 80% was added during the preparation of fly medium to a final concentration of 15 μg/ml. A total number of 150 *Surf1 Switch-Act5C-Gal4 KD* flies and the same number of control flies were starved and then placed in groups of 10 into RU-486-containing food.

## RESULTS

**FIGURE 1.**
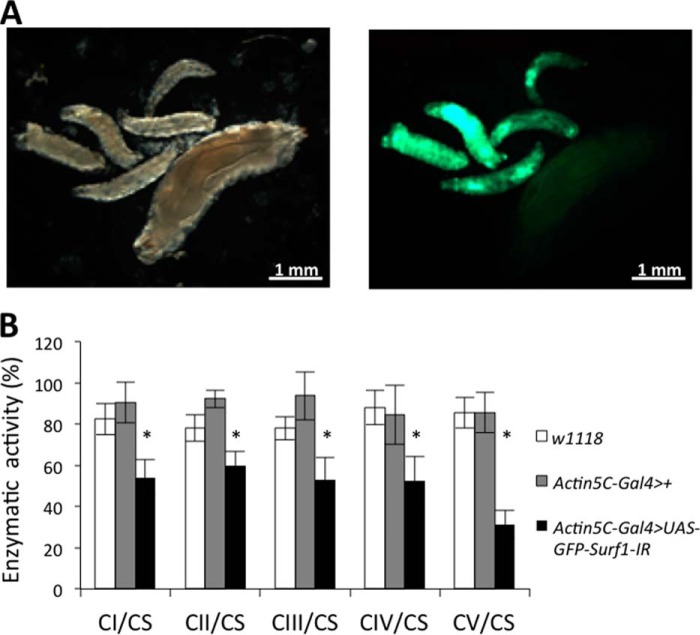
**Developmental and biochemical effects following *Surf1* ubiquitous KD.** *A*, *Surf1* KD larvae (GFP positive) were considerably smaller in comparison with control larvae (GFP negative). *B*, enzymatic activities of MRC complexes (I-IV) and F-ATP synthase (complex V) were measured in *w^1118^* (*open column*), *Actin5C-Gal4*>+ (*gray column*), and *Actin5C-Gal4*>*UAS-GFP-Surf1-IR* (*closed column*) 3rd instar larvae. Complexes I-V activities were normalized to the activity of CS. For each genotype, three replicate mitochondrial preparations were analyzed; for each, enzymatic activities from at least ten replicate reactions were performed. Data plotted are mean ± S.D. (Student's *t* test, *, *p* < 0.005).

**FIGURE 2.**
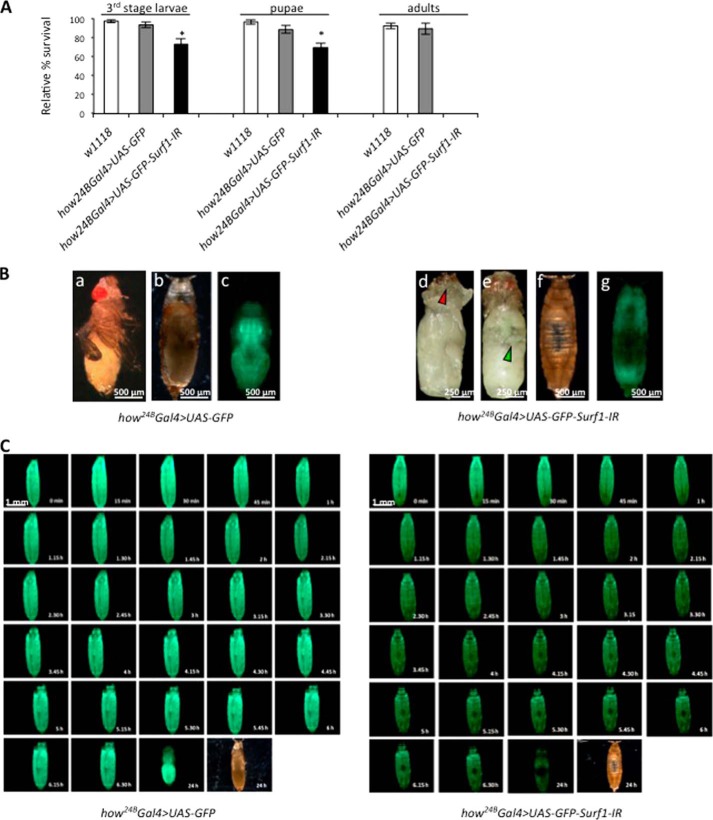
**Activation of *Surf1* post-transcriptional silencing in the mesodermal derivatives.** All experiments were carried out in *w^1118^* (*open column*) as well as in specific controls *how^24B^ Gal4>UAS-GFP* (*gray column*) and in *how^24B^ Gal4>UAS-GFP-Surf1-IR* (*closed column*) KD individuals. *A*, relative percentage of egg to adult viability calculated at three developmental stages (*i.e.* 3rd instar larvae, pupae and adults). Data plotted are mean ± S.D. (Student's *t* test, *, *p* < 0.005). *B*, images refer to 6-day-old control pupae (*panels a–c*) and *Surf1 how^24B^Gal4* KD pupae (*panels d–g*) represented without puparium (*panels a*, *d*, *e*) (*arrows* mark structure abnormalities), with puparium (*panels b*, *c*, *f*, *g*) and by fluorescence microscopy (*panels c* and *g*). *C*, monitoring of specific control and KD pupae development starting from the beginning of pupal metamorphosis (*PM*) by fluorescence microscopy.

The *Actin5C-Gal4* driver was used for early, ubiquitous expression of dsRNAi-mediated KD. Starting from individuals bearing the *UAS-Surf1-IR* construct we also introduced a *UAS-GFP* transgene to allow identification of KD larvae based on their green fluorescence. *Surf1* ubiquitous post-transcriptional silencing produced 100% egg-to adult lethality with death occurring at the larval stage. The *Surf1 Act-Gal4* KD 3rd instar larvae were smaller (Fig. 1*A*), yet they did possess mouth hooks (the distinctive character of the 3rd larval instar) indicating that their smaller size was not due to a developmental delay, as already reported (34). We next tested activity of the MRC individual complexes, to assess for the occurrence of biochemical defects, which have been reported in patients and in the mouse model (24, 57, 58). *Surf1 Act-Gal4 KD* larvae showed a marked decrease in activity of all respiratory complexes and of complex V rather than the expected selective decrease of COX (Fig. 1*B*). Also muscle-specific KD of *Surf1* resulted in pupal lethality, albeit development progressed to slightly later stages (Fig. 2). When *Surf1* was post-transcriptionally silenced in all mesodermal derivatives from early embryonic stages to pupal metamorphosis (PM) by the *how^24B^Gal4* driver, KD individuals did not develop to adulthood, and they died during the early stages of PM or at slightly earlier stages, failing to progress beyond the pupal stage (Fig. 2*A*). Dissection of the pupae revealed that death occurred at early imago stages, when adult cuticular structures had only just everted. Apolysis (separation of the cuticula from the underlying epidermal cells) (59) did not occur correctly, and larval cuticular structures, such as mouth hooks (Fig. 2*B*, *red arrowhead*), were maintained in the pupa; gas bubble that forms in the abdomen (59) was not displaced toward the anterior region of the puparium (Fig. 2*B*, *green arrowhead*), and the head did not evert (Fig. 2*B*). As clearly visible in the fluorescence microscope images of 6-day-old pupae, adult muscle did not develop in the *Surf1 how^24B^Gal4* KD pupae (Fig. 2*B*). Indeed, *Surf1 how^24B^Gal4* KD pupae displayed a GFP-negative region approximately at the center of the developing insect body, suggesting that muscle tissue was not present there (Fig. 2*B*; compare with the appearance of control pupae). In order to establish the time point at which the void region began to appear, we monitored pupal development in *Surf1 how^24B^Gal4* KD from the beginning of PM. The “holes” appeared ∼3–4 h from the beginning of PM, and they continued to expand in the following 4 h (Fig. 2*C*).

**FIGURE 3.**
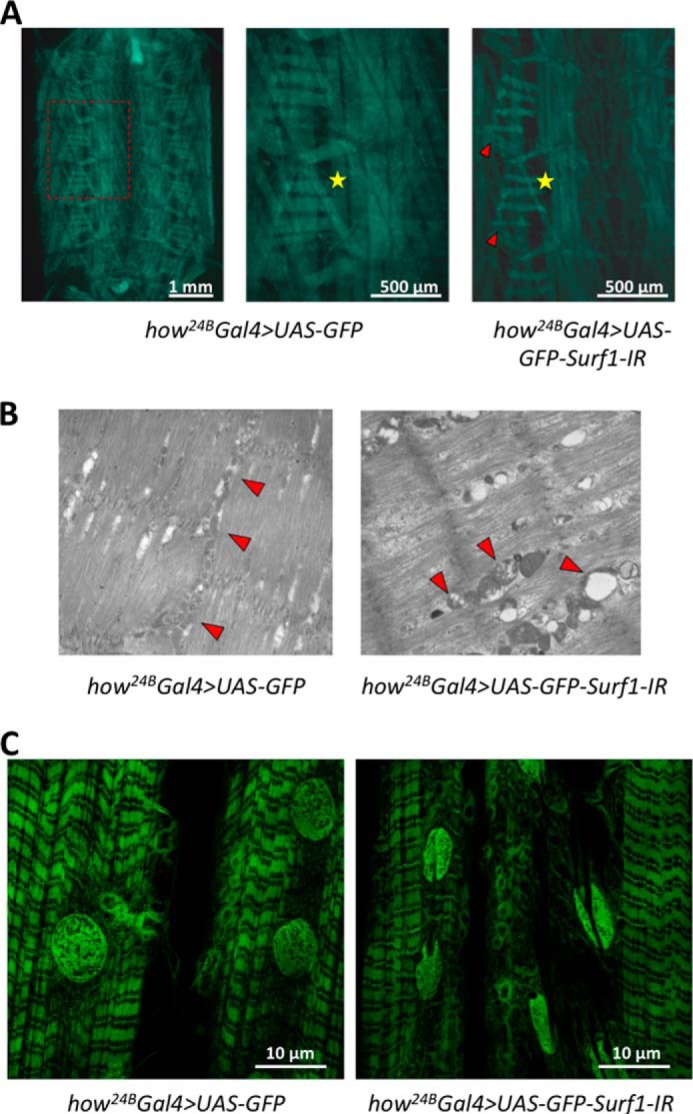
**Muscle examination of *Surf1 how^24B^ Gal4* KD larvae.** Characterization was carried out on control (*how^24B^ Gal4>UAS-GFP*) and KD (*how^24B^ Gal4>UAS-GFP-Surf1-IR*) larvae. *A*, body-wall preparation of control and KD larvae; the *red arrows* indicate impaired muscle, and the *yellow stars* mark the position in which all KD individuals miss a specific longitudinal fiber in the abdominal segment 4. *B*, cross-sectional ultrastructure of larval muscle, illustrating the distribution and morphology of mitochondria, obtained by electromicroscopy of 3rd instar larval body-wall sections. *C*, confocal microscope images showing larval muscular fibers of control and KD larvae.

We also checked the state of the muscle (the main mesodermal derivative tissue undergoing *Surf1* post-transcriptional silencing) by examining body-wall preparations of both specific controls and *Surf1 how^24B^Gal4* KD larvae. KD larvae showed impaired muscle development (Fig. 3*A*, *red arrowheads*) and also missed a specific longitudinal fiber in the abdominal segment 4 (Fig. 3*A*, *yellow stars*). Defects in muscle mitochondria of *Surf1 how^24B^Gal4* KD larvae were also documented by electron microscopy, which revealed enlarged and rounded organelles, often with a disorganized or disrupted internal structure (Fig. 3*B*, *red arrows*). In addition, muscle fibers showed vacuolizations (Fig. 3*B*). Finally, confocal microscope analysis of larval body-wall preparations revealed that *Surf1 how^24B^Gal4* KD muscle fibers have profound alterations in the morphology of the nuclei, which presented a peculiar shape reminiscent of a coffee bean (Fig. 3*C*).

**FIGURE 4.**
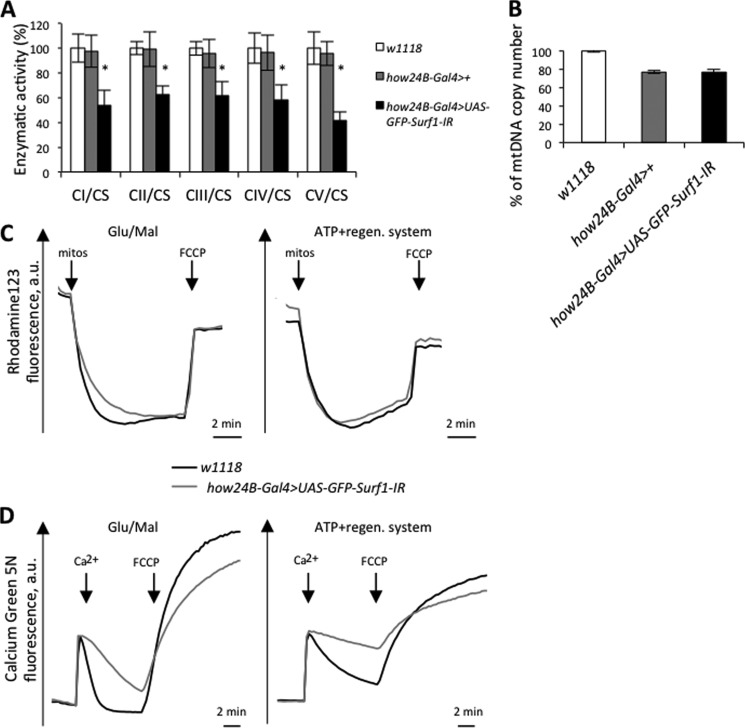
**Bioenergetic properties in *Surf1 how^24B^Gal4* KD larvae and wild type larvae mitochondria.** *A*, enzymatic activities of MRC complexes (I-IV) and F-ATP synthase (complex V). Complexes I-V activities were normalized to the activity of CS. For each genotype, three replicate mitochondrial preparations were analyzed; for each, enzymatic activities from at least ten replicate reactions were performed. *B*, mtDNA content was measured by quantitative real time-PCR. Data are ratios of mtDNA to genomic DNA relative to the control ratio in *w^1118^* larvae. *C*, measurement of mitochondrial membrane potential based on fluorescent quenching of Rhodamine123. Mitochondria were isolated as described under “Experimental Procedures” and added (*first arrow*) to the assay medium containing 250 mm sucrose, 10 mm MOPS-Tris, 5 mm Pi-Tris, 10 μm EGTA, 0.4 μm Rhodamine123, and 5 mm glutamate-Tris plus 2.5 mm malate-Tris (*left panel*), or 4 mm MgCl_2_, 2 mm phosphocreatine, 0.4 mm ATP, and 1.5 units/ml creatine kinase (*right panel*), pH 7.4. One micromolar FCCP was added as indicated (*arrows*). *D*, mitochondrial Ca^2+^ uptake assay. Mitochondria isolated from *Surf1 how^24B^Gal4* KD and wild type larvae were incubated in the assay media described in *B*, except that Rhodamine123 was substituted with 0.5 μm Calcium Green 5N. Where indicated 15 μm Ca^2+^ or 1 μm FCCP were added. All traces are representative of three independent experiments for both genotypes. *a.u*., arbitrary units.

Analysis of *Surf1 how^24B^Gal4* KD larvae mitochondria demonstrated a markedly reduced activity in all MRC complexes and the F-ATP synthase (Fig. 4*A*). We also measured the mtDNA copy number in KD individuals and we observed that *Surf1 how^24B^Gal4* KD larvae had levels of mtDNA close to controls (Fig. 4*B*). Thus, the biochemical defect is not caused by decreased mtDNA copy number.

**FIGURE 5.**
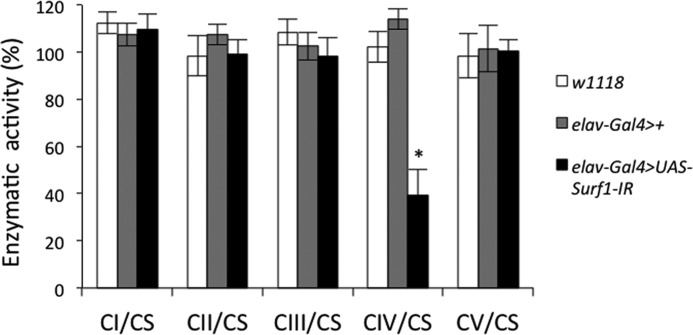
**Biochemical effects of *Surf1* central nervous system-wide silencing.** Enzymatic activities of MRC complexes (I-IV) and F-ATP synthase (complex V) carried out in *w^1118^* (*open column*) as well as in specific controls *elav-Gal4*>+ (*gray column*) and in KD *elav-Gal4*>*UAS-Surf1-IR* (*closed column*) individuals. Complexes I-V activities were normalized to the activity of CS. Mitochondria were prepared from the heads of individuals of each genotype, and enzymatic activities determined from at least ten replicate reactions for each. Data plotted are mean ± S.D. from three preparations (Student's test, *, *p* < 0.005).

The membrane potential of mitochondria was measured based on the uptake of Rhodamine123, a cationic fluorescent probe that is accumulated by energized mitochondria and undergoes fluorescence quenching, which is reverted by depolarization with the uncoupler carbonylcyanide-*p*-trifluoromethoxy phenylhydrazone (FCCP). It can be seen that the resting potential (*i.e.* the level of fluorescence quenching) was the same for mitochondria of both genotypes irrespective of whether they were energized with glutamate/malate or with ATP (Fig. 4*C*); yet, when Ca^2+^ fluxes were measured with the impermeant Ca^2+^-sensitive probe Calcium Green 5N (whose fluorescence increases when Ca^2+^ is present) a clear decrease in the rate of Ca^2+^ uptake was observed in *Surf1 how^24B^Gal4* KD mitochondria (Fig. 4*D*), consistent with decreased maximal activity of both the MRC and complex V. To bypass larval lethality caused by the ubiquitous and muscle-selective KD of *Surf1*, we restricted the expression of the dsRNAi to the CNS by using a pan-neuronal *elav-GAL4* driver (34, 36). The *elav-GAL4* driver induces dsRNAi in the developing central and peripheral nervous systems, and in the eye and antenna disc from early embryogenesis throughout larval development (36). We have already demonstrated that *Surf1 elav-Gal4* KD dsRNAi had no effects on egg-to-adult transition (34) allowing assays to be carried out in mitochondria prepared from heads of wild type (*w^1118^*), control (*elav-Gal4*>+) and *Surf1 elav-Gal4* KD adults. We found that COX activity was greatly reduced in heads of *Surf1 elav-Gal4* KD flies, whereas other MRC complexes and the F-ATP synthase were not affected (Fig. 5).

**FIGURE 6.**
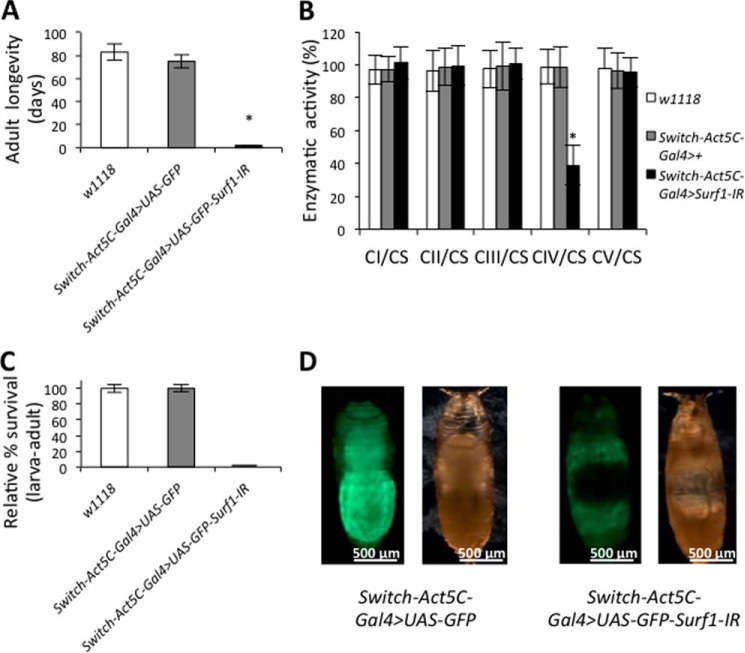
**Ubiquitous *Surf1* knockdown induced after exposure to RU-486.** All experiments were carried out on *w^1118^* (*open column*), *Switch-Act5C-Gal4*>+ (*gray column*), and *Switch-Act5C-Gal4*>*UAS-GFP-Surf1-IR* (*closed column*) larvae. *A*, longevity of adult flies, expressed as days of survival. *B*, MRC complexes (I-IV) and F-ATP synthase (complex V). Complexes I-V activities, normalized to the activity of CS. For each genotype, three replicate mitochondrial preparations were analyzed; for each, enzymatic activities from at least ten replicate reactions were performed. *C*, relative percentage of larva-adult viability. *D*, images refer to 6 days old specific control (*left panel*) and S*urf1* S*witch-Act5C-Gal4* KD pupae (*right panel*), respectively in bright field and fluorescence microscopy. Data plotted are means ± S.D. (Student's test, *, *p* < 0.005).

We next used the RU-486 sensitive-ubiquitous driver (*Switch-Act5C-Gal4*) to trigger *Surf1* post-transcriptional silencing at different developmental stages by switching flies to RU-486 containing medium. Food supplementation with RU-486 led to 100% lethality of adult *Surf1 Switch-Act5C-Gal4* KD flies within 48 h, whereas all controls survived (Fig. 6*A*). While complex I, II, III, and F-ATP synthase activities did not significantly change, *Surf1 Switch-Act5C-Gal4* KD individuals showed 60% reduction in COX activity (Fig. 6*B*). Next, we triggered *Surf1* silencing in 3rd instar larvae. *Surf1 Switch-Act5C-Gal4* KD larvae reached the pupal stage but failed to develop into adults (Fig. 6*C*). The pupal phenotype of *Surf1 Switch-Act5C-Gal4* KD individuals (Fig. 6*D*) was very similar to that observed after activation of RNAi in the mesodermal derivatives (Fig. 2*B*).

**FIGURE 7.**
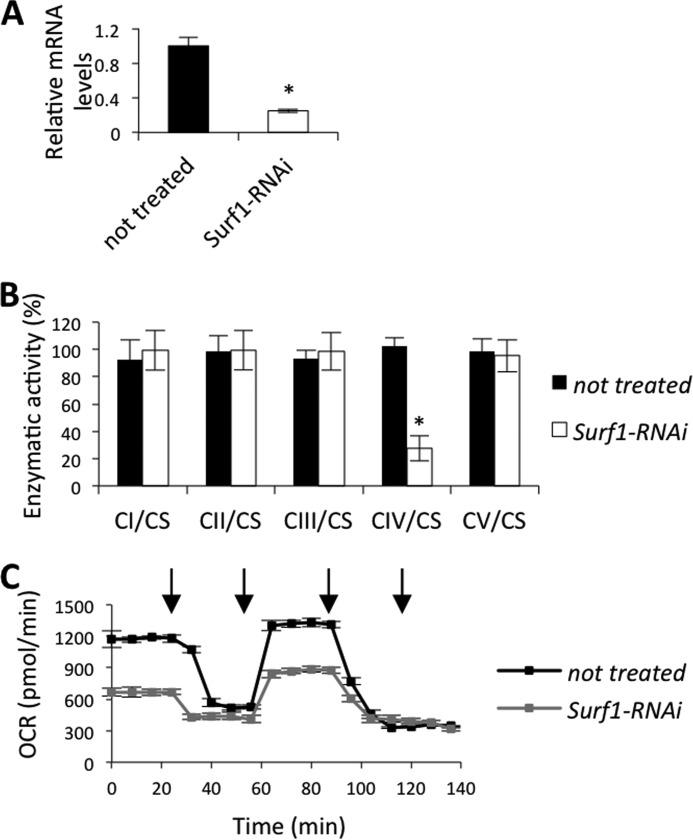
***Surf1* post-transcriptional silencing in S_2_R^+^ cells.** *A*, *Surf1* mRNA levels, expressed as relative quantity of template in the sample, were determined by qRT-PCR in untreated cells (*closed column*), and cells silenced for the *Surf1* expression (*open column*). *B*, MRC complexes (I-IV) and F-ATP synthase (complex V) were measured in untreated (*closed bars*) and silenced (*open bars*) cells. Complexes I-IV activities, normalized to the activity of CS. For each genotype, three replicate mitochondrial preparations were analyzed; for each, enzymatic activities from at least ten replicate reactions were performed. *C*, respiratory profile of control (*black symbols*) and *Surf1*-silenced (*gray symbols*) S2R^+^ cells. Five micromolar oligomycin, 1 μm FCCP, 5 μm rotenone, and 5 μm antimycin A were added at the times marked by *arrows*. Data plotted are mean ± S.D. (Student's test, *, *p* < 0.005).

Next, we down-regulated the expression of endogenous *Surf1* in S2R^+^ cells and tested the effects on OXPHOS complex activities. In cells incubated for 96 h with dsRNA, real time RT-PCR analysis showed that mRNA was decreased by 70% (Fig. 7*A*). If SURF1 has a role in COX assembly, its down-regulation should affect COX activity. To test this prediction, we assessed the complexes I-V activities of control and silenced S2R^+^ cells and we observed impairment of COX but not of other MRC complexes or F-ATP synthase (Fig. 7*B*). Measurement of oxygen consumption rates of S2R^+^ cells silenced for *Surf1* revealed that both basal (oligomycin-sensitive) and maximal (FCCP-stimulated) rates of oxygen consumption were dramatically decreased in cells silenced for 96 h, indicating that the COX defect slows down respiration *in situ* (Fig. 7*C*).

**FIGURE 8.**
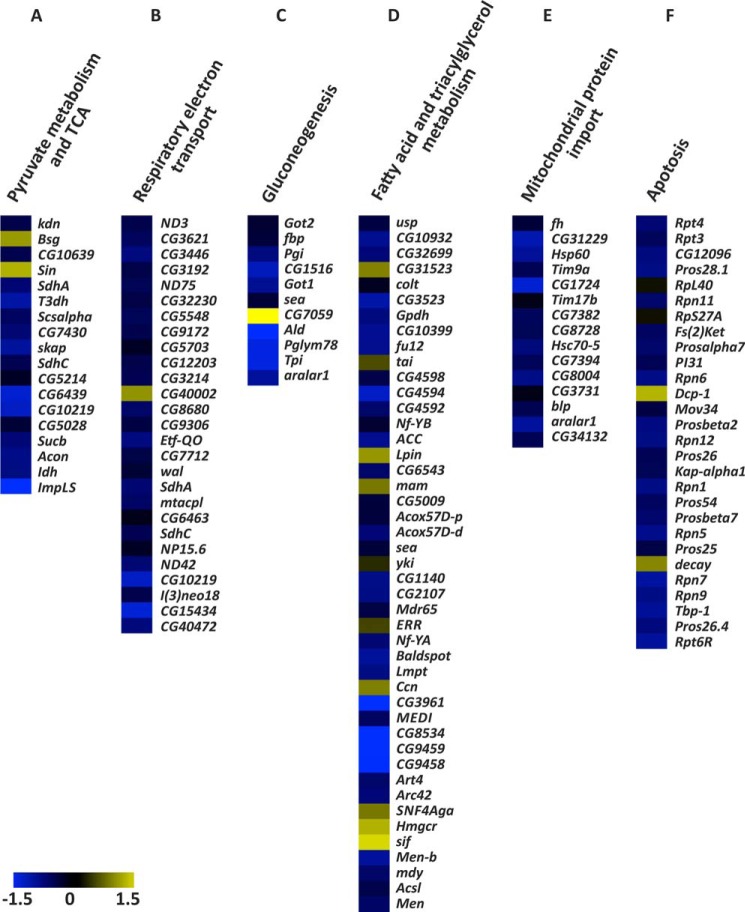
**Altered gene pathways in *Surf1* KD first larva instar.** Heat map representing a selection of deregulated transcripts, provided by Reactome tool, in *Surf1 Act-Gal4* KD (IR) *versus* control (*CTR*) samples involved in *A*) pyruvate metabolism and citric acid cycle (18 transcripts), *B*) respiratory electron transport (27 transcripts), *C*) gluconeogenesis (11 transcripts), *D*) fatty acid and triacylglycerol metabolism (45 transcripts), *F*) apoptosis (28 transcripts), and *E*) mitochondrial protein import (15 transcripts). A color-coded scale for the normalized expression values is used: *yellow* and *blue* represent high and low expression levels in *Surf1 Act-Gal4* KD compared with controls, respectively. The expression level of each transcript was calculated as the Log2 (IR */* CTR), and the complete list of differentially expressed genes identified by LIMMA algorithm is provided in the Supplementary Information (supplemental Table S1).

Finally, to define the gene expression pattern specifically associated with the *Surf1* KD we performed protein-coding microarray analyses (*Drosophila* 1.0 custom platform, Agilent Technologies) on high quality RNA from *Surf1 Act-Gal4* KD individuals. We analyzed 1st instar larvae in order to identify gene pathway(s) that are altered as an early consequence of the *Surf1* silencing, and may be rescued in later larvae stages. Using LIMMA two class analysis we identified 5,020 differentially expressed genes (adjusted *p* value <0.05), 1,974 of which were up-regulated (39%) and 3,046 down-regulated (61%) in *Surf1 Act-Gal4* KD (supplemental Table S1). The Graphite web tool was used to identify functional categories occurring in the *Surf1 Act-Gal4* KD 1st instar larvae expression signature more frequently than expected by chance. The hypergeometric test (q-value<0.05) was used to rank biological significance of deregulated genes obtained with Reactome database. We observed that biological pathways overrepresented in the down-regulated component of the expression signature included pyruvate metabolism and the citric acid cycle (TCA) (*e.g. Knockdown*, *kdn*; *Basigin*, *bsg*; *Succinate dehydrogenase A*, *SdhA*; *Aconitase*, *Acon*; *Succinate dehydrogenase C*, *SdhC*); respiratory electron transport (*e.g. mitochondrial NADH-ubiquinone oxidoreductase chain 3*, *ND3*; *NADH:ubiquinone reductase 75kD subunit precursor*, *ND75*; *Electron transfer flavoprotein-ubiquinone oxidoreductase*, *Etf-QO*; *walrus*, *wal*; *NADH:ubiquinone reductase 42kD subunit precursor*, *ND42*); gluconeogenesis (*e.g. Glutamate oxaloacetate transaminase 2*, *Got2*; *fructose-1,6-bisphosphatase*, *fbp*; *Phosphoglucose isomerase*, *Pgi*; *Aldolase*, *Ald*; *Triose phosphate isomerase*, *Tpi*); fatty acid and triacylglycerol metabolism (*e.g. Ultraspiracle*, *usp*; *Glycerol 3 phosphate dehydrogenase*, *Gpdh*; *Malic enzyme*, *Men*; *Arginine methyltransferase 4*, *Art4*; *Acyl-Coenzyme A oxidase at 57D proximal*, *Acox57D-p*); mitochondrial protein import (*e.g. frataxin homolog*, *fh*; *Heat shock protein 60*, *Hsp60*; *black pearl*, *blp*; *Heat shock protein cognate 5*, *Hsc70–5*); and apoptosis (*e.g. Regulatory particle triple-A ATPase 4*, *Rpt4*; *Regulatory particle non-ATPase 11*, *Rpn11*; *Proteasome* α*7 subunit*, *Prosalpha7*; *Regulatory particle non-ATPase 8*, *Mov34*; *Regulatory particle triple-A ATPase 6-related*, *Rpt6R*) (Fig. 8). In contrast, the up-regulated components showed an overrepresentation of the biological pathways involved in protein synthesis such as translation initiation, elongation, and termination.

## DISCUSSION

LS^COX^ is a progressive infantile mitochondrial encephalopathy (20, 31, 32) most frequently caused by mutations in *SURF1,* a nuclear gene encoding a protein located in the inner membrane (18, 19). This fatal neurological disorder usually leads to death within the first decade of life (31). The biochemical hallmark of LS^COX^ patients with mutations in *SURF1* is a marked decrease in the activity of COX (33), the terminal enzyme of the MRC, whose proper assembly requires a number of accessory factors including the SURF1 protein (21). The absence, or malfunctioning, of SURF1 determines the accumulation of COX assembly intermediates and a drastic reduction in the amount of fully assembled enzyme both in yeast and humans (18, 32). Mice with genetic inactivation of the *Surf1* gene showed a mild decrease in COX activity and lower body mass, yet failed to show neurodegeneration at any age, and displayed markedly prolonged lifespan (22, 60). Thus, an animal model of SURF1 deficiency mimicking the severe human disease is missing; and the present work fills this gap by providing an adult fly with the biochemical hallmark of the human disease and a neurological phenotype amenable to further study.

To establish an animal model of LS^COX^ it was necessary to ubiquitously silence the *Surf1* gene in adult *D. melanogaster* because we show that ubiquitous or mesodermal-selective *Surf1* KD in larvae causes a severe phenotype leading to larval lethality. As in the case of ubiquitous silencing, *Surf1* muscle-specific KD was lethal, albeit development progressed to slightly later stages. This can probably be ascribed to the cardinal role played by contractile movements during the progression through pupal development (for example during head eversion). Defective development of muscle was observed in *Surf1 how^24B^Gal4* KD pupae, which showed GFP-negative (*i.e.* without muscle tissue) body regions, although some larval muscles appear to be more affected than others (see *e.g.* the lateral transverse muscle attachment sites, Fig. 3*A*). Larval body wall preparations allowed a detailed study of mitochondrial morphology, revealing closely stacked mitochondria in a costameric arrangement on either side of the muscle Z-lines. Besides the ultrastructural alterations of mitochondria of *Surf1 how^24B^Gal4* KD larvae the “coffee-bean” nuclei suggest initiation of karyorexis, an early sign of apoptosis.

When measured in the larvae, whole-body and muscle-specific *Surf1* KD unexpectedly affected the activity of all MRC complexes and F-ATP synthase. If the function of *Surf1* is related exclusively to its role as a chaperone in the assembly of COX, the KD of *Surf1* would not be expected to have direct effects on the activity of other complexes. Yet, as noted previously (61), lack of *Surf1* can lead to a variety of phenotypes in both yeast (62, 63) and mouse models (22); together with the present findings on *Drosophila* larvae it appears possible that *Surf1* may play a more general role in organization of the OXPHOS complexes, particularly at early developmental stages. Indeed, pan-neuronal silencing (which permits the development to adulthood) of *Surf1* in adults and silencing in S2R^+^ cells yielded a COX-restricted defect even in *Drosophila*. This might be suggestive of a different involvement of the OXPHOS throughout *Drosophila* development, in particular before and after the metamorphosis (64).

Detailed analysis of the transcriptome profile following *Surf1* ubiquitous silencing clearly demonstrated a strong down-regulation of energy-conserving pathways. Specifically, enzymes of glycolysis, tricarboxylic acid cycle, and oxidative phosphorylation were strongly down-regulated, suggesting an overall impairment in energy conservation. This results paralleled the *in situ* results since both *Surf1* (*how^24B^Gal4*) and (*Act-Gal4*) KD larvae showed (i) loss of MRC activity, (ii) defects in Ca^2+^ uptake rates, which may lead to disorders in Ca^2+^ homeostasis and contractility, and (iii) altered mitochondrial morphology suggesting mitochondrial dysfunction. This condition could be worsened by decreased biogenesis, as suggested by down-regulation of the mitochondrial protein import machinery observed in gene expression profile of KD larvae.

In conclusion, we have characterized an LS^COX^ model in *D. melanogaster*. Our findings strongly suggest that *Surf1* is essential for COX assembly and activity, hence mitochondrial function in *Drosophila*. It is encouraging that the lethal effects of ubiquitous *Surf1* KD in *Drosophila* could be rescued by the concomitant expression of *Ciona intestinalis* alternative oxidase under the control of an inducible promoter (61). Thus, *Drosophila* LS^COX^ offers a new tool to clarify the pathogenesis and potential therapy of Leigh syndrome.

## Supplementary Material

Supplemental Data

## Footnotes

3 The abbreviations used are:

OXPHOS: oxidative phosphorylation
CNS: central nervous system
COX: cytochrome *c* oxidase
CS: citrate synthase
FCCP: carbonyl cyanide *p*-trifluoromethoxyphenylhydrazone
F: forward
KD: knockdown
LS: Leigh syndrome
MRC: mitochondrial respiratory chain
mt: mitochondrial
mtDNA: mitochondrial DNA
nDNA: nuclear DNA
PM: pupal metamorphosis
qRT-PCR: quantitative PCR
R: reverse.
