# Supplementary material for: Leigh Syndrome in Drosophila melanogaster: MORPHOLOGICAL AND BIOCHEMICAL CHARACTERIZATION OF Surf1 POST-TRANSCRIPTIONAL SILENCING
Source: J Biol Chem. 2014 Aug 27;289(42):29235–46. doi: 10.1074/jbc.M114.602938 (PMC4200275; doi:10.1074/jbc.M114.602938)
Supplement: Supplemental Data [file supp_289_42_29235__index.html]

Leigh Syndrome in Drosophila melanogaster: Morphological and Biochemical Characterization of Surf1 Post-transcriptional Silencing — Leigh Syndrome in Drosophila melanogaster — Post-transcriptional Silencing of the COX Assembly Factor SURF1 — Supplemental Data 

# Leigh Syndrome in *Drosophila melanogaster*

## Supplemental Data

**Files in this Data Supplement:**

- Supplemental Table 1 (.pdf, 1.2 MB) - List of differentially expressed genes following Surf1 Ubiquitous KD. Different gene expression between Surf1 Act-Gal4 KD (IR) vs. control (CTR) 1st instar larvae was detected by LIMMA two class analysis (p-value &#x26;lt;0.05). The expression level of each transcript was calculated as the Log2 [expression value]. calculated as the Log2 [expression value].
