# Supplementary material for: Leigh Syndrome in Drosophila melanogaster: MORPHOLOGICAL AND BIOCHEMICAL CHARACTERIZATION OF Surf1 POST-TRANSCRIPTIONAL SILENCING
Source: J Biol Chem. 2014 Aug 27;289(42):29235–46. doi: 10.1074/jbc.M114.602938 (PMC4200275; doi:10.1074/jbc.M114.602938)
Supplement: Supplemental Data [file supp_M114.602938_jbc.M114.602938-1.pdf]

TABLE S1. List of differentially expressed genes following Surf1 ubiquitous KD. Different gene expression between Surf1 Act-Gal4 KD (IR) *vs*. control (CTR) 1st instar larvae was detected by LIMMA two class analysis (p-value <0.05). The expression level of each transcript was calculated as the Log2 [expression value].

Probe Name	Flybase ID	Entrez Gene	Gene Symbol	CTR1	CTR2	CTR3	CTR4	IR1	IR2	IR3	IR4	Log2(IR/CTR)
A_09_P000016	FBtr0089606	2768994	CG33493	13.29	13.59	13.56	13.46	12.07	12.25	12.61	12.83	**-1.04**
A_09_P000041	FBtr0091446	3346177	CG33506	12.60	12.29	12.40	12.27	11.89	11.60	11.89	12.18	**-0.50**
A_09_P000046	FBtr0340554	3346227	dpr2	4.81	4.36	4.73	4.14	6.02	6.74	5.83	5.90	**1.61**
A_09_P000051	FBtr0091448	3346226	ppk13	6.89	8.21	7.61	8.31	9.41	9.45	9.75	9.90	**1.87**
A_09_P000056	FBtr0091449	3346225	CG33509	6.20	7.73	7.09	7.59	8.30	8.60	8.54	8.55	**1.35**
A_09_P000066	FBtr0091451	3346223	CG33511	9.71	9.62	9.86	9.49	7.45	8.04	8.73	9.25	**-1.31**
A_09_P000096	FBtr0070034	33007	D2R	5.72	5.46	5.16	5.20	6.28	7.22	6.57	6.53	**1.26**
A_09_P000111	FBtr0113464	3346201	Unc-89	14.02	14.41	14.45	14.38	13.49	13.39	13.58	13.83	**-0.74**
A_09_P000116	FBtr0310384	326128	Ada2a	10.14	10.12	10.19	10.28	9.34	9.45	9.62	9.79	**-0.63**
A_09_P000121	FBtr0310384	326128	Ada2a	10.43	10.40	10.45	10.58	9.66	9.77	9.91	10.14	**-0.59**
A_09_P000126	FBtr0091473	3346141	CG33521	11.72	12.31	12.19	12.17	10.87	10.65	10.79	11.12	**-1.24**
A_09_P000151	FBtr0302859	32941	CoRest	7.57	7.64	7.13	7.31	6.07	5.97	6.12	6.38	**-1.28**
A_09_P000176	FBtr0091491	3346192	Vmat	9.40	8.83	9.10	8.92	9.49	9.68	9.68	9.73	**0.58**
A_09_P000188	FBtr0091493	3346192	Vmat	9.88	9.75	9.75	9.54	10.60	10.88	10.39	10.75	**0.92**
A_09_P000201	FBtr0091498	3346222	lectin-37Da	6.94	7.26	7.25	6.58	9.16	9.51	9.96	10.27	**2.72**
A_09_P000236	FBtr0091512	40858	gfzf	10.56	10.15	10.44	10.00	9.29	9.24	9.34	9.51	**-0.95**
A_09_P000241	FBtr0305021	42150	Rim	7.67	7.34	7.38	7.36	8.43	8.81	8.54	8.40	**1.11**
A_09_P000246	FBtr0070421	31200	msta	12.00	12.10	12.15	11.98	10.99	10.91	11.27	11.63	**-0.86**
A_09_P000316	FBtr0113470	2768716	mim	9.31	9.84	9.58	9.86	9.06	8.93	9.13	9.24	**-0.56**
A_09_P000526	FBtr0303383	3771968	Msp-300	12.00	11.95	12.23	11.94	11.43	11.27	11.26	11.19	**-0.74**
A_09_P000621	FBtr0091775	3772288	CG33774	10.57	10.63	10.42	10.70	9.40	9.65	9.90	10.32	**-0.76**
A_09_P000821	FBtr0091930	39092	CG33926	10.39	11.16	10.44	11.11	12.96	12.79	12.74	12.59	**1.99**
A_09_P000906	FBtr0100020	3885575	CG33977	10.43	10.69	10.31	10.60	9.22	9.31	9.62	9.96	**-0.98**
A_09_P000926	FBtr0303077	3885667	CG42729	8.87	9.14	9.03	8.93	8.73	8.14	8.38	7.73	**-0.75**
A_09_P000941	FBtr0100033	37953	CG33988	9.68	9.73	9.68	9.58	10.58	11.05	10.46	10.78	**1.05**

A_09_P000976	FBtr0100058	3885611	nimB3	13.85	14.59	14.41	14.66	13.22	13.51	13.29	13.30	**-1.05**
A_09_P001021	FBtr0100070	3885570	CG34015	9.55	9.28	9.45	9.41	8.70	8.44	8.80	8.86	**-0.72**
A_09_P001041	FBtr0299943	37164	CG42351	12.15	12.51	12.20	12.39	11.17	11.40	11.44	11.71	**-0.88**
A_09_P001121	FBtr0300555	43787	PMCA	8.37	8.27	8.44	8.23	7.85	7.70	7.76	7.88	**-0.53**
A_09_P001131	FBtr0301014	3885643	CG34039	9.53	9.52	9.44	9.70	9.01	8.84	9.11	9.32	**-0.48**
A_09_P001156	FBtr0302390	3885656	CG34045	8.06	8.17	8.35	8.41	5.90	6.24	6.35	6.55	**-1.99**
A_09_P001201	FBtr0100109	3885601	CG34054	8.69	8.39	8.46	8.24	9.51	9.29	9.21	9.10	**0.84**
A_09_P001216	FBtr0100834	3885645	CG34057	6.20	6.24	5.92	6.04	6.65	7.47	7.11	7.24	**1.02**
A_09_P001236	FBtr0113773	3355155	CG40002	8.34	8.13	8.03	8.13	9.16	8.76	9.16	9.21	**0.92**
A_09_P001386	FBtr0111266	3355016	CG40127	13.04	13.02	12.85	13.04	12.68	12.34	12.62	12.65	**-0.41**
A_09_P001476	FBtr0113817	3354941	CG40178	9.30	9.24	9.21	9.15	10.23	9.73	9.88	9.86	**0.70**
A_09_P001506	FBtr0113824	3354930	CG40191	11.39	11.45	11.26	11.37	10.61	10.18	10.70	10.90	**-0.77**
A_09_P001721	FBtr0086131	35584	Eb1	7.66	8.07	7.87	7.88	7.41	7.00	7.12	7.26	**-0.67**
A_09_P001891	FBtr0075782	39554	endos	11.35	11.72	11.53	11.84	11.32	11.19	11.17	11.17	**-0.40**
A_09_P001906	FBtr0070951	31601	Ctr1A	10.38	10.56	10.38	10.46	10.13	10.32	10.13	10.08	**-0.28**
A_09_P001911	FBtr0086781	37071	CG17680	12.61	12.64	12.80	12.77	12.16	12.29	12.21	12.37	**-0.44**
A_09_P001916	FBtr0085405	43459	CG1458	13.93	14.01	13.98	14.13	13.38	13.45	13.52	13.67	**-0.50**
A_09_P001956	FBtr0100145	39864	Lasp	10.56	10.56	10.48	10.42	11.38	11.22	11.03	10.84	**0.61**
A_09_P002001	FBtr0086726	37105	GstE10	9.47	9.14	9.33	9.34	9.10	8.83	8.83	8.83	**-0.42**
A_09_P002041	FBtr0091719	3772658	CG33722	11.19	11.11	11.08	11.16	10.69	10.81	10.77	10.93	**-0.33**
A_09_P002046	FBtr0111132	3355124	RpL5	16.71	16.65	16.68	16.64	16.72	16.76	16.79	16.75	**0.09**
A_09_P002066	FBtr0113478	32696	Arpc3B	12.56	12.44	12.34	12.36	12.99	12.85	12.76	12.71	**0.40**
A_09_P002091	FBtr0100231	251466	RpL41	15.94	15.95	15.97	16.02	16.04	16.07	16.08	16.08	**0.09**
A_09_P002106	FBtr0332945	2768715	CheB42c	6.09	6.55	6.62	6.37	4.40	5.02	5.41	5.94	**-1.22**
A_09_P002151	FBtr0113895	3355107	Tim17b	13.04	12.90	12.99	12.87	12.82	12.67	12.81	12.80	**-0.17**
A_09_P002161	FBtr0082999	2768669	CG33332	9.99	9.99	9.97	10.01	9.01	8.99	9.05	9.24	**-0.92**
A_09_P002181	FBtr0072779	44100	Patj	10.83	10.99	10.98	11.16	10.50	10.57	10.58	10.66	**-0.41**
A_09_P002201	FBtr0082098	41177	eca	14.88	14.97	14.89	14.92	14.08	14.14	14.50	14.74	**-0.55**
A_09_P002416	FBtr0308892	3354890	CG40498	8.33	7.98	8.12	7.94	8.69	8.59	8.51	8.42	**0.46**
A_09_P002441	FBtr0113483	43633	tmod	9.61	10.01	9.90	10.06	9.26	8.94	9.11	9.14	**-0.78**
A_09_P002461	FBtr0111012	2768685	mld	10.41	10.11	10.21	10.23	10.85	11.06	10.82	10.85	**0.66**

A_09_P002471	FBtr0071449	50417	Neb-cGP	15.05	14.89	15.13	15.08	14.43	14.68	14.72	14.74	-0.40
A_09_P002481	FBtr0100388	36740	Zasp52	12.25	13.05	12.89	13.09	12.31	11.80	12.02	11.98	-0.79
A_09_P002536	FBtr0110806	4379844	CG34113	7.14	6.82	6.70	6.59	7.91	8.33	7.93	7.94	1.21
A_09_P002541	FBtr0110808	4379854	CG34114	4.77	4.91	4.81	5.05	5.68	5.89	5.76	5.74	0.88
A_09_P002551	FBtr0110816	4379912	CG34116	8.32	8.52	8.40	8.53	8.08	8.16	8.11	8.18	-0.31
A_09_P002636	FBtr0110879	4379884	CG34132	12.91	12.45	12.65	12.51	11.99	12.10	12.24	12.29	-0.48
A_09_P002666	FBtr0110947	4379900	CG34137	5.80	5.87	5.28	4.34	6.64	7.00	6.41	6.45	1.31
A_09_P002681	FBtr0110975	4379908	CG34140	10.18	10.25	10.25	10.35	10.17	10.02	9.97	10.00	-0.22
A_09_P002686	FBtr0301512	4379870	CG34141	9.77	9.58	9.70	9.38	9.02	8.57	9.02	9.09	-0.68
A_09_P002716	FBtr0111019	326250	CG34148	9.12	8.98	9.23	8.98	8.54	8.41	8.87	8.84	-0.41
A_09_P002721	FBtr0111020	4379861	CG34149	7.78	7.60	7.62	7.72	8.80	8.96	8.49	8.23	0.94
A_09_P003006	FBtr0112356	5740685	CG34165	13.05	12.56	12.66	12.92	14.83	15.22	14.50	13.87	1.80
A_09_P003091	FBtr0112376	5740752	CG34183	10.26	10.17	10.24	10.26	9.39	9.51	9.80	10.10	-0.53
A_09_P003146	FBtr0112387	5740534	CG34194	10.93	10.56	10.47	10.43	12.05	12.10	11.65	11.51	1.23
A_09_P003166	FBtr0112391	5740481	CG34198	8.33	7.87	8.10	7.85	10.15	9.72	9.63	9.24	1.65
A_09_P003446	FBtr0112449	5740347	blos3	9.88	9.74	9.74	9.88	8.99	9.25	9.45	9.69	-0.46
A_09_P003561	FBtr0112473	5740656	CG34278	13.42	14.80	14.82	14.92	12.62	13.18	12.34	11.72	-2.02
A_09_P003586	FBtr0112478	42248	CG34282	14.64	14.95	14.99	15.10	14.17	14.34	14.03	14.00	-0.79
A_09_P003596	FBtr0112480	5740234	CG34284	9.63	9.69	9.85	10.00	8.92	9.11	8.98	8.90	-0.82
A_09_P003626	FBtr0112487	5740331	CG34291	13.42	13.09	13.29	13.47	13.56	13.96	13.86	13.94	0.51
A_09_P003636	FBtr0112489	5740555	CG34293	8.65	8.58	8.42	8.71	8.17	8.39	8.23	8.37	-0.30
A_09_P003641	FBtr0112490	5740503	CG34294	6.77	6.81	6.45	6.42	7.07	7.45	7.07	7.10	0.56
A_09_P003721	FBtr0112506	5740536	CG34310	10.03	9.61	9.90	9.64	9.11	8.98	9.14	9.23	-0.68
A_09_P003781	FBtr0112532	2768869	CG34330	12.82	13.69	12.85	13.70	11.69	12.53	11.98	12.12	-1.19
A_09_P003846	FBtr0329841	35277	sick	6.90	6.97	6.52	6.45	7.50	8.26	7.41	7.67	1.00
A_09_P003851	FBtr0329846	35277	sick	7.68	7.43	7.62	7.17	8.91	9.07	8.92	8.67	1.42
A_09_P003881	FBtr0112557	32072	CG34348	9.99	10.80	10.43	10.47	8.97	8.97	9.26	9.56	-1.23
A_09_P003921	FBtr0331578	5740442	CG34356	6.21	5.55	5.35	4.86	7.19	7.46	7.16	7.31	1.79
A_09_P003956	FBtr0112580	35738	Dgk	6.10	6.36	6.67	6.98	5.65	4.36	4.86	5.27	-1.49
A_09_P003961	FBtr0334897	43362	CG34362	8.51	8.29	8.17	8.27	9.27	9.45	9.25	9.20	0.98
A_09_P003966	FBtr0112583	43362	CG34362	7.56	7.43	7.43	7.44	8.15	8.45	8.28	8.19	0.80

A_09_P004021	FBtr0331989	5740629	CG43795	6.69	6.23	6.29	5.81	7.16	7.53	7.29	7.39	**1.09**
A_09_P004031	FBtr0334847	34032	Rapgap1	10.25	10.01	10.15	9.94	10.32	10.26	10.39	10.45	**0.27**
A_09_P004046	FBtr0112609	42638	CG34376	9.32	9.03	9.27	9.08	10.35	10.03	9.99	9.93	**0.90**
A_09_P004066	FBtr0112614	36592	Shroom	10.81	10.94	10.75	10.85	11.70	11.60	11.52	11.54	**0.75**
A_09_P004071	FBtr0329979	5740323	CG34380	10.09	10.84	10.25	10.68	11.01	11.26	10.94	10.99	**0.59**
A_09_P004076	FBtr0330222	33812	TrissinR	5.48	4.79	4.77	4.58	6.53	6.80	6.43	6.27	**1.60**
A_09_P004086	FBtr0329899	5740131	CG34383	6.89	7.66	6.94	7.52	7.65	8.03	8.01	8.10	**0.70**
A_09_P004101	FBtr0112627	5740303	CG34386	8.81	8.01	8.13	8.08	9.91	9.75	9.34	9.09	**1.27**
A_09_P004111	FBtr0112629	5740700	CG34388	7.57	7.80	7.57	7.66	7.96	8.23	8.09	8.36	**0.51**
A_09_P004131	FBtr0112636	35588	Epac	9.14	8.83	8.87	8.60	9.89	9.76	9.40	9.28	**0.72**
A_09_P004136	FBtr0112637	33534	CG34393	5.45	4.74	4.54	4.67	5.80	5.86	5.84	5.92	**1.01**
A_09_P004146	FBtr0112641	33552	CG34394	10.43	10.54	10.33	10.26	11.74	11.53	11.24	10.94	**0.97**
A_09_P004281	FBtr0112691	2768848	mute	7.98	8.11	8.29	8.18	7.43	7.62	7.69	7.75	**-0.51**
A_09_P004301	FBtr0303125	38863	Ank2	10.09	9.97	10.14	9.91	10.91	10.88	10.65	10.63	**0.74**
A_09_P004316	FBtr0112704	31591	CG34417	11.82	12.12	11.95	12.11	11.55	11.43	11.61	11.48	**-0.48**
A_09_P004331	FBtr0112710	43892	sif	9.93	9.76	9.81	9.09	10.77	11.23	10.41	10.79	**1.15**
A_09_P004446	FBtr0112738	5740179	CG34437	9.35	10.18	9.64	10.29	8.10	8.38	8.72	9.10	**-1.29**
A_09_P004456	FBtr0112740	5740593	CG34439	13.53	13.65	13.64	13.74	13.12	13.27	13.18	13.42	**-0.39**
A_09_P004461	FBtr0112741	5740593	CG34439	10.45	10.32	10.42	10.59	9.44	9.72	9.72	9.73	**-0.80**
A_09_P004466	FBtr0079695	5740853	ImgA	7.98	8.16	7.91	7.92	7.06	6.93	7.50	7.75	**-0.68**
A_09_P004471	FBtr0112743	5740853	ImgA	8.73	8.50	8.76	8.62	7.84	7.80	8.00	8.25	**-0.68**
A_09_P004491	FBtr0112749	5740761	CG34445	11.58	11.61	11.80	11.57	11.33	11.14	11.13	10.71	**-0.56**
A_09_P004496	FBtr0112750	5740549	CG34446	8.53	8.50	8.68	8.81	8.07	7.70	7.82	7.57	**-0.84**
A_09_P004501	FBtr0112751	5740813	CG34447	8.53	NA	8.12	NA	4.93	5.53	6.44	6.94	**-2.37**
A_09_P004531	FBtr0299517	5740876	CG34452	9.14	8.46	9.01	8.70	6.26	6.73	7.01	7.36	**-1.99**
A_09_P004566	FBtr0112765	5740101	CG34459	9.49	9.80	9.82	9.92	7.29	7.36	7.60	7.88	**-2.23**
A_09_P004651	FBtr0302234	5740846	CG40625	5.78	5.36	5.29	5.63	4.78	3.98	4.21	4.00	**-1.27**
A_09_P005576	FBtr0088565	35921	CG8777	9.67	9.69	9.62	9.65	9.41	9.30	9.43	9.42	**-0.27**
A_09_P005596	FBtr0088569	35926	Phax	10.99	10.96	11.00	11.04	10.48	10.72	10.69	10.75	**-0.34**
A_09_P005601	FBtr0088579	35928	GstE13	11.78	11.66	11.78	11.81	11.52	11.60	11.59	11.61	**-0.18**
A_09_P005646	FBtr0088591	35946	Cyp4p2	9.95	10.06	9.51	9.31	11.29	11.81	10.99	10.53	**1.45**

A_09_P005651	FBtr0088593	35948	Cyp4p3	11.07	11.13	11.15	11.22	10.71	10.86	10.65	10.68	-0.42
A_09_P005656	FBtr0088600	35952	CG2063	10.13	10.07	10.00	9.92	9.74	9.48	9.66	9.85	-0.34
A_09_P005731	FBtr0088520	35981	CG1809	13.46	13.01	12.96	12.97	14.58	14.83	14.30	13.74	1.26
A_09_P005751	FBtr0088541	35987	CG12929	9.48	9.51	9.51	9.68	9.19	9.34	9.21	9.17	-0.32
A_09_P005806	FBtr0088474	36006	CG1698	11.15	11.07	11.06	11.18	13.44	13.88	13.07	12.45	2.10
A_09_P005811	FBtr0088493	36009	CG1648	13.03	13.27	13.21	13.25	14.16	14.27	13.82	13.91	0.85
A_09_P005821	FBtr0088487	36011	hebe	14.18	13.69	13.80	13.64	15.05	14.86	14.79	14.65	1.01
A_09_P005831	FBtr0088485	36013	Lsm11	7.75	7.87	7.85	7.58	7.00	7.26	7.07	7.07	-0.66
A_09_P005846	FBtr0088479	36016	CG1667	10.99	11.62	11.32	11.59	10.66	10.56	10.68	10.78	-0.71
A_09_P005856	FBtr0088375	36022	Ntmt	10.90	10.94	11.05	10.92	10.58	10.65	10.59	10.57	-0.36
A_09_P005861	FBtr0088376	36023	CG18446	9.63	10.17	9.83	10.15	8.15	8.36	8.54	8.78	-1.48
A_09_P005866	FBtr0088377	36024	CG12744	8.91	9.10	9.08	9.18	8.72	8.45	8.69	8.91	-0.38
A_09_P005871	FBtr0088451	36025	sec24	10.09	10.41	10.29	10.41	9.68	9.60	9.88	9.98	-0.52
A_09_P005891	FBtr0088384	36031	Etf-QO	13.55	13.83	13.75	13.85	12.89	12.75	13.09	13.39	-0.72
A_09_P005906	FBtr0088440	36035	CG1418	11.50	11.58	11.51	11.67	10.21	10.30	10.93	11.40	-0.85
A_09_P005916	FBtr0088388	36038	CG12134	12.05	11.66	11.87	11.69	12.22	12.37	12.21	12.12	0.41
A_09_P005941	FBtr0088395	36044	CG12129	10.43	10.27	10.38	10.47	9.89	9.63	9.84	9.88	-0.57
A_09_P005951	FBtr0088433	36046	sel	13.93	14.48	14.22	14.30	12.52	12.36	13.09	13.53	-1.36
A_09_P005956	FBtr0088401	36048	magu	7.22	8.40	7.86	8.00	6.46	6.18	6.49	6.81	-1.39
A_09_P005976	FBtr0088428	36053	CG1371	13.19	13.18	13.28	13.04	12.95	12.84	12.85	12.97	-0.27
A_09_P005986	FBtr0088409	36056	CG2269	9.61	9.44	9.63	9.50	10.21	10.44	10.36	10.39	0.81
A_09_P006041	FBtr0088336	36081	CG12911	11.18	10.64	11.12	10.85	11.97	12.11	11.89	11.86	1.01
A_09_P006051	FBtr0088341	36084	CAP	9.06	9.54	9.47	9.28	9.04	8.69	8.63	8.63	-0.59
A_09_P006121	FBtr0088314	36103	CG12895	11.48	11.25	11.24	11.19	11.04	10.96	11.04	11.11	-0.25
A_09_P006131	FBtr0088310	36107	Caf1-105	8.69	8.54	8.68	8.71	7.93	8.29	8.24	8.26	-0.48
A_09_P006136	FBtr0088273	36108	CG11777	8.95	8.97	8.82	9.06	7.76	8.04	8.18	8.40	-0.85
A_09_P006181	FBtr0339465	36129	CG7220	11.09	10.77	10.57	10.69	12.12	11.97	11.79	11.41	1.04
A_09_P006251	FBtr0089729	36154	CG18004	10.45	10.56	10.41	10.53	9.84	9.94	10.08	10.38	-0.43
A_09_P006261	FBtr0088238	36159	CG7712	10.66	10.63	10.77	10.90	10.22	10.16	10.31	10.38	-0.47
A_09_P006286	FBtr0088202	36167	BBS4	4.23	3.34	3.48	3.90	5.16	5.51	5.05	5.31	1.52
A_09_P006296	FBtr0088203	36169	CG13231	8.96	9.08	8.89	8.90	8.61	8.65	8.69	8.64	-0.31

A_09_P006301	FBtr0088205	36170	CG12391	7.88	8.16	8.01	8.04	6.98	7.30	7.58	7.73	-0.62
A_09_P006306	FBtr0088197	36172	CG9084	10.15	9.89	9.71	9.63	11.97	11.66	11.47	11.07	1.70
A_09_P006361	FBtr0088112	36189	Cpr47Eb	14.21	13.80	13.57	14.15	15.58	15.74	15.62	15.56	1.70
A_09_P006371	FBtr0088189	36191	Cpr47Ec	16.45	16.84	16.85	16.71	15.02	15.76	15.56	15.57	-1.23
A_09_P006396	FBtr0088182	36197	CG9067	10.50	10.35	10.41	10.54	9.91	9.95	10.16	10.31	-0.37
A_09_P006406	FBtr0088116	36200	CG13220	14.24	13.98	14.18	14.02	13.15	13.34	13.56	13.74	-0.66
A_09_P006426	FBtr0303157	36205	CG42336	14.47	14.47	14.52	14.58	13.82	13.91	14.14	14.31	-0.47
A_09_P006486	FBtr0088133	36234	CG7759	11.90	11.55	11.77	11.45	10.22	10.36	10.36	10.50	-1.31
A_09_P006501	FBtr0088089	36243	CG9005	7.31	7.61	7.29	7.36	7.94	8.15	7.80	7.83	0.54
A_09_P006551	FBtr0088031	36259	CG13192	9.26	9.06	9.21	8.95	8.67	8.10	8.42	8.55	-0.69
A_09_P006556	FBtr0088064	36260	Sobp	8.50	8.56	8.47	8.47	8.06	8.32	7.94	7.92	-0.44
A_09_P006566	FBtr0088032	36263	Sln	7.74	7.43	7.50	7.41	8.62	8.91	8.37	8.31	1.04
A_09_P006591	FBtr0088058	36270	ERp60	14.85	14.93	14.97	14.80	13.38	13.05	14.13	14.57	-1.11
A_09_P006621	FBtr0088052	36277	Pl31	10.94	10.98	11.06	11.07	10.61	10.38	10.76	10.78	-0.38
A_09_P006626	FBtr0088045	36281	rho-7	11.88	11.62	11.78	11.71	11.38	11.54	11.53	11.55	-0.25
A_09_P006636	FBtr0088027	36284	CG8964	7.30	7.59	7.69	7.49	7.26	7.07	7.13	6.98	-0.40
A_09_P006641	FBtr0088025	36285	Mppe	7.90	8.05	8.03	7.98	7.23	7.21	7.49	7.67	-0.59
A_09_P006656	FBtr0088022	36293	CG8888	14.19	14.75	14.51	14.78	13.73	13.62	13.56	13.61	-0.93
A_09_P006686	FBtr0088016	36304	Prp8	12.19	12.15	12.20	12.29	11.80	11.83	11.99	11.99	-0.31
A_09_P006706	FBtr0100143	3771946	CG13175	9.87	9.40	9.78	10.00	9.11	9.25	9.32	9.26	-0.53
A_09_P006761	FBtr0302923	36330	CG42700	5.91	6.27	6.24	5.88	4.42	4.29	4.68	5.19	-1.43
A_09_P006776	FBtr0087896	36335	CG13163	11.06	10.77	10.89	10.84	10.52	10.35	10.57	10.65	-0.37
A_09_P006801	FBtr0087902	36339	Den1	10.86	10.90	10.88	11.02	10.76	10.70	10.67	10.71	-0.20
A_09_P006836	FBtr0087916	36348	Cpr49Ac	12.16	13.56	12.11	13.07	13.84	14.01	13.94	13.81	1.18
A_09_P006841	FBtr0087955	36349	Cpr49Ad	10.45	8.49	8.96	8.76	10.63	11.05	10.67	10.61	1.57
A_09_P006861	NM_136932	36353	Cpr49Ag	14.51	13.74	14.03	13.73	14.92	15.01	14.71	14.47	0.78
A_09_P006871	FBtr0087925	36355	CG13157	7.23	3.17	4.32	5.42	NA	NA	8.74	9.51	4.09
A_09_P006886	FBtr0087927	36358	CG8525	13.25	13.53	13.46	13.51	12.52	12.71	12.84	12.92	-0.69
A_09_P006921	FBtr0087934	36365	CG8545	12.87	12.38	12.58	12.48	13.20	13.31	12.98	12.88	0.52
A_09_P006926	FBtr0087935	36366	CG8550	10.41	11.51	11.07	11.43	8.51	8.53	9.50	9.96	-1.98
A_09_P006976	FBtr0087826	36381	ClC-b	10.39	10.75	10.55	10.63	9.94	9.77	10.09	10.09	-0.61

A_09_P006981	FBtr0110824	36385	CG17760	6.68	6.84	6.57	6.31	7.69	7.62	7.26	7.13	0.82
A_09_P006996	FBtr0087880	36392	CG8778	12.28	12.21	12.24	12.20	11.84	11.74	11.82	11.90	-0.41
A_09_P007001	FBtr0087844	36393	CG8632	11.42	11.42	11.45	11.44	11.01	11.01	11.15	11.37	-0.30
A_09_P007031	FBtr0087871	36400	CG8768	10.00	9.97	9.98	9.97	9.91	9.75	9.87	9.82	-0.14
A_09_P007046	FBtr0302645	36403	CG42663	10.38	10.43	10.32	10.28	11.12	11.15	10.83	10.62	0.58
A_09_P007081	FBtr0087788	36423	CG13319	9.61	9.93	9.82	9.98	9.17	9.36	9.40	9.68	-0.43
A_09_P007121	FBtr0087797	36433	CG13323	13.12	13.49	13.47	13.55	15.45	14.80	15.13	14.93	1.67
A_09_P007126	FBtr0087796	36434	CG13324	13.08	13.29	13.36	13.34	15.67	14.85	15.31	15.07	1.96
A_09_P007131	FBtr0087794	36436	Drl-2	6.60	7.01	6.43	6.66	7.28	7.58	7.24	7.11	0.63
A_09_P007136	FBtr0087722	36437	CG13325	6.69	7.25	8.63	7.12	11.91	10.81	10.99	10.35	3.60
A_09_P007166	FBtr0087766	36456	AQP	9.89	10.33	10.26	10.30	9.54	9.11	9.43	9.50	-0.80
A_09_P007181	FBtr0087764	36459	CG4646	11.04	10.98	11.09	11.09	10.47	10.58	10.70	10.89	-0.39
A_09_P007231	FBtr0087748	36472	CG10799	11.41	11.93	11.37	11.82	12.45	12.40	12.67	12.83	0.96
A_09_P007291	FBtr0299757	36488	CG42321	10.97	11.18	11.21	11.14	10.17	10.20	10.42	10.64	-0.77
A_09_P007296	FBtr0087666	36492	cbs	9.64	10.36	10.03	10.54	9.25	9.34	9.33	9.51	-0.79
A_09_P007301	FBtr0087701	36494	cbc	9.77	9.75	9.90	9.92	9.37	9.31	9.53	9.63	-0.37
A_09_P007306	FBtr0087699	36496	bbc	10.84	10.89	10.76	10.81	10.61	10.48	10.47	10.47	-0.32
A_09_P007316	FBtr0087669	36499	mip120	10.28	10.29	10.40	10.28	9.93	9.94	10.02	10.23	-0.29
A_09_P007336	FBtr0087686	36506	CG6145	10.83	10.53	10.93	10.60	11.50	12.13	11.57	11.26	0.89
A_09_P007411	FBtr0087631	36526	stj	7.33	6.98	6.89	7.03	7.89	8.07	7.93	8.05	0.93
A_09_P007416	FBtr0087632	36528	CG13339	9.72	9.58	9.49	9.75	9.99	10.10	10.07	10.18	0.45
A_09_P007421	FBtr0087633	36529	CG6329	9.16	8.79	9.05	8.61	9.62	9.65	9.35	9.62	0.66
A_09_P007446	FBtr0087645	36533	synaptogyrin	9.67	9.26	9.46	9.26	9.97	10.07	10.19	10.21	0.70
A_09_P007451	FBtr0087644	36536	CG6543	15.78	15.77	15.93	15.81	15.00	15.03	15.37	15.50	-0.60
A_09_P007466	FBtr0087622	36540	CG16935	12.92	12.94	13.04	13.18	12.31	12.10	12.36	12.52	-0.70
A_09_P007501	FBtr0087606	36551	Ctf4	9.70	9.27	9.55	9.47	8.58	8.65	8.91	9.13	-0.68
A_09_P007521	FBtr0087583	36560	Rcd1	11.61	11.92	11.79	11.89	12.14	12.09	11.98	12.00	0.25
A_09_P007531	FBtr0087519	36562	CG13016	10.41	10.21	10.30	10.29	9.59	9.69	9.83	9.97	-0.53
A_09_P007536	FBtr0087580	36563	CG8257	10.35	10.22	10.30	10.33	9.46	9.47	9.72	9.89	-0.66
A_09_P007541	FBtr0087520	36564	O-fut1	9.96	10.44	10.17	10.28	8.60	8.75	9.49	9.93	-1.02
A_09_P007546	FBtr0087579	36565	Tango7	14.85	14.93	14.92	14.93	15.08	15.23	15.16	15.23	0.27

A_09_P007551	FBtr0087521	36566	CG8323	11.56	11.36	11.60	11.35	10.89	10.97	11.03	11.13	**-0.46**
A_09_P007566	FBtr0087525	36569	CG8331	15.21	15.37	15.40	15.29	14.53	14.53	14.93	15.03	**-0.56**
A_09_P007601	FBtr0087536	36581	CG8503	12.87	12.75	12.88	12.71	12.47	12.34	12.32	12.23	**-0.46**
A_09_P007626	FBtr0087546	36593	CG8613	10.89	10.77	10.91	10.92	10.42	10.45	10.56	10.64	**-0.35**
A_09_P007631	FBtr0087561	36594	CG8617	10.99	11.25	11.18	11.44	10.65	10.67	10.76	10.84	**-0.49**
A_09_P007646	FBtr0087547	36597	Arc2	8.07	8.60	8.48	8.21	10.61	10.60	10.59	10.61	**2.26**
A_09_P007656	FBtr0087556	36600	Obp50e	7.03	5.42	5.77	4.98	8.07	8.14	7.74	7.22	**1.99**
A_09_P007661	FBtr0087485	36601	Dh44-R1	6.77	5.83	6.28	5.95	7.45	7.70	7.53	7.39	**1.31**
A_09_P007671	FBtr0087517	36603	CG17385	11.60	11.61	11.56	11.59	11.39	11.44	11.40	11.31	**-0.21**
A_09_P007711	FBtr0301735	36620	CG12868	10.38	10.61	10.60	10.49	12.81	12.58	12.14	11.54	**1.74**
A_09_P007776	FBtr0087440	36640	CG12859	14.58	14.52	14.51	14.56	14.09	14.11	14.23	14.38	**-0.34**
A_09_P007801	FBtr0087445	36648	CG10200	13.12	13.02	12.94	13.35	13.52	13.99	13.72	13.61	**0.60**
A_09_P007841	FBtr0300127	36664	Cyp6a20	10.07	8.94	9.52	9.09	11.63	11.30	10.98	10.38	**1.67**
A_09_P007901	FBtr0087404	36682	CG8090	9.84	9.96	9.67	9.92	9.09	8.50	9.16	9.35	**-0.82**
A_09_P007911	FBtr0087402	36685	row	11.13	11.34	11.36	11.46	10.86	10.74	10.97	11.02	**-0.42**
A_09_P007921	FBtr0087401	36686	CG8093	10.27	9.85	10.44	9.71	13.51	13.63	13.45	13.21	**3.39**
A_09_P007941	FBtr0087398	2768836	Fs	6.41	6.67	5.83	5.93	6.96	7.35	6.97	7.02	**0.87**
A_09_P007961	FBtr0087391	36698	CG8155	8.99	9.06	9.03	9.04	8.71	8.90	8.88	8.71	**-0.23**
A_09_P007966	FBtr0087390	36700	CG8157	15.47	15.17	15.18	15.13	15.91	15.89	15.96	16.07	**0.72**
A_09_P007971	FBtr0087389	36701	CG8160	10.42	7.87	9.18	8.27	11.60	11.83	11.76	11.55	**2.75**
A_09_P007986	FBtr0344208	5740318	CG43729	7.43	7.62	7.53	7.40	8.74	8.91	8.07	8.61	**1.09**
A_09_P008016	FBtr0087336	36722	eIF2B-gamma	10.48	10.45	10.33	10.32	10.04	10.15	10.08	10.25	**-0.27**
A_09_P008031	FBtr0087353	36725	CG8195	8.77	9.19	8.96	9.08	8.51	8.20	8.45	8.60	**-0.56**
A_09_P008071	FBtr0087290	36749	CG8297	9.88	9.79	9.81	9.89	9.54	9.67	9.62	9.73	**-0.20**
A_09_P008076	FBtr0087293	36750	Mlf	12.17	12.27	12.33	12.21	11.74	11.60	11.94	12.08	**-0.40**
A_09_P008091	FBtr0087201	36754	CG8366	10.10	10.19	10.11	10.29	9.49	9.29	9.50	9.68	**-0.68**
A_09_P008121	FBtr0087264	36762	CG8386	12.49	12.49	12.45	12.59	12.02	12.15	12.21	12.33	**-0.33**
A_09_P008131	FBtr0087205	36764	CG8389	9.23	9.91	9.37	9.81	10.76	10.70	10.43	10.32	**0.97**
A_09_P008141	FBtr0087261	36767	CG8397	13.78	13.73	13.85	13.75	13.21	13.25	13.44	13.61	**-0.40**
A_09_P008146	FBtr0087207	36768	CG8399	11.70	11.39	11.59	11.26	13.07	13.30	12.72	12.10	**1.31**
A_09_P008181	FBtr0305091	36778	Asph	14.53	14.73	14.78	14.72	13.50	13.49	13.82	14.20	**-0.94**

A_09_P008236	FBtr0113084	36797	mrj	11.26	11.25	11.34	11.20	11.04	11.05	10.87	10.76	**-0.33**
A_09_P008241	FBtr0087192	36798	CG7798	8.03	9.25	8.85	9.54	5.52	5.99	6.57	7.38	**-2.55**
A_09_P008376	FBtr0087127	36843	CG15712	9.60	NA	9.80	NA	2.47	NA	7.79	8.46	**-3.46**
A_09_P008396	FBtr0087093	36848	Syn2	7.37	6.48	6.70	6.35	8.33	8.00	8.17	8.22	**1.45**
A_09_P008406	FBtr0087100	36850	CG4945	9.99	10.17	10.08	10.16	9.25	9.39	9.43	9.66	**-0.67**
A_09_P008411	FBtr0087123	36851	RpS15	15.44	15.28	15.42	15.36	15.51	15.55	15.59	15.57	**0.18**
A_09_P008421	FBtr0087122	36853	CG8317	13.44	12.12	12.90	12.34	14.04	14.36	14.01	13.86	**1.36**
A_09_P008471	FBtr0087079	36876	unc-104	9.72	9.75	9.56	9.66	10.19	10.28	10.15	10.20	**0.53**
A_09_P008496	FBtr0087033	36883	CG5550	9.94	7.35	8.66	8.13	14.47	13.91	13.82	13.01	**5.29**
A_09_P008501	FBtr0087042	36891	CG6426	13.17	13.09	12.83	13.39	14.03	14.24	13.76	13.57	**0.78**
A_09_P008526	FBtr0087054	36903	CG6665	10.07	10.30	10.23	10.23	9.91	9.68	9.78	9.87	**-0.40**
A_09_P008541	FBtr0087059	36908	ste24a	8.78	8.73	8.52	8.60	8.27	7.80	8.15	8.28	**-0.54**
A_09_P008576	FBtr0086974	36918	CG9646	10.47	10.69	10.67	10.76	11.37	11.43	11.10	10.95	**0.56**
A_09_P008591	FBtr0086979	36926	CG6984	10.55	10.60	10.70	10.82	9.98	9.88	10.22	10.40	**-0.55**
A_09_P008611	FBtr0086984	36934	Cda9	11.25	11.37	11.53	11.63	9.60	10.02	9.76	9.71	**-1.67**
A_09_P008666	FBtr0086916	36955	CG4802	12.54	12.49	12.41	12.57	11.68	11.65	11.83	11.99	**-0.71**
A_09_P008671	FBtr0086917	36957	Lhr	8.37	8.40	8.29	8.41	7.12	7.04	7.75	8.19	**-0.84**
A_09_P008681	FBtr0086957	36960	mthl4	8.03	8.87	8.47	8.66	9.46	10.10	9.46	9.32	**1.08**
A_09_P008716	FBtr0086939	36974	CG4853	8.74	8.98	9.09	9.05	8.68	8.69	8.62	8.60	**-0.32**
A_09_P008721	FBtr0086951	36975	Apc10	10.72	10.69	10.76	10.69	9.96	9.91	10.05	10.33	**-0.65**
A_09_P008756	FBtr0086906	36989	ns2	12.02	11.74	11.93	11.82	12.79	12.82	12.51	12.25	**0.71**
A_09_P008766	FBtr0086904	36993	Dcr-2	9.31	9.68	9.94	9.88	9.04	9.26	9.13	8.97	**-0.60**
A_09_P008821	FBtr0086840	37009	HPS4	8.45	8.77	8.65	8.75	7.89	8.02	7.89	7.74	**-0.77**
A_09_P008851	FBtr0086862	37017	CG4984	11.81	11.71	11.75	11.76	11.41	10.68	11.22	11.35	**-0.59**
A_09_P008861	FBtr0086882	37020	CG6401	9.52	9.50	9.49	9.40	8.63	8.76	8.96	9.18	**-0.60**
A_09_P008866	FBtr0086864	37021	CG4996	10.41	10.59	10.46	10.54	10.08	10.25	10.16	10.20	**-0.33**
A_09_P008891	FBtr0089559	37029	OstDelta	12.17	12.21	12.00	12.16	11.10	10.95	11.66	11.92	**-0.73**
A_09_P008901	FBtr0089557	37031	CG18635	7.84	6.64	7.03	6.53	9.67	9.27	9.07	8.59	**2.14**
A_09_P008926	FBtr0300215	3885598	dpr13	7.18	6.51	6.78	6.28	7.58	7.89	7.56	7.64	**0.98**
A_09_P008931	FBtr0086804	37051	CG5084	8.81	9.23	9.22	9.37	8.70	7.52	8.36	8.31	**-0.94**
A_09_P008936	FBtr0089630	37052	CG10910	15.06	15.11	15.04	15.17	16.13	15.80	15.80	15.62	**0.74**

A_09_P008951	FBtr0086811	37055	CG5767	11.35	11.04	11.16	11.32	10.06	9.13	9.23	8.75	**-1.92**
A_09_P008956	FBtr0086810	37056	CG14495	7.73	7.94	7.74	7.38	10.58	10.18	9.87	9.15	**2.25**
A_09_P008961	FBtr0086809	37057	Muc55B	13.79	13.72	13.65	13.53	15.46	14.43	15.01	14.75	**1.24**
A_09_P008966	FBtr0086808	37058	CG10911	12.93	12.87	13.01	13.05	15.40	15.20	15.11	14.60	**2.11**
A_09_P008971	FBtr0086807	37059	CG10912	13.78	13.62	13.80	13.74	15.65	14.73	15.36	15.21	**1.51**
A_09_P009001	FBtr0086746	37076	CG10915	7.90	8.10	7.82	7.85	7.40	7.65	7.55	7.52	**-0.39**
A_09_P009006	FBtr0086775	37078	Nup75	11.53	11.48	11.47	11.51	10.93	10.85	11.05	11.23	**-0.49**
A_09_P009016	FBtr0086749	37081	CG10916	10.56	10.59	10.58	10.67	10.97	11.14	11.20	11.33	**0.56**
A_09_P009036	FBtr0086752	37087	CG14500	11.55	11.68	11.49	12.08	13.05	12.69	12.56	12.20	**0.92**
A_09_P009086	FBtr0086663	37101	CG18107	7.93	7.38	7.38	6.86	10.31	10.60	9.88	9.63	**2.72**
A_09_P009091	FBtr0086728	37102	CG15067	8.42	4.68	6.16	4.75	13.73	13.51	12.95	11.78	**6.99**
A_09_P009096	FBtr0086669	37106	GstE1	11.99	11.83	11.91	11.93	12.59	12.44	12.43	12.42	**0.55**
A_09_P009102	FBtr0086684	37117	CG5174	12.41	12.77	12.68	12.71	12.31	12.22	12.32	12.31	**-0.35**
A_09_P009111	FBtr0086690	37117	CG5174	12.02	12.06	12.13	12.08	11.88	11.56	11.83	11.79	**-0.31**
A_09_P009116	FBtr0086691	37118	CG12263	11.57	11.17	11.23	11.14	10.87	10.75	10.88	10.97	**-0.41**
A_09_P009141	FBtr0304865	37129	CG43066	9.08	8.59	8.70	8.69	9.66	9.79	9.46	9.36	**0.80**
A_09_P009171	FBtr0086713	37139	CG5493	12.59	12.84	12.78	12.95	11.71	11.89	12.07	12.29	**-0.80**
A_09_P009181	FBtr0086712	37141	Atg7	8.66	9.12	9.05	9.15	8.58	8.64	8.48	8.50	**-0.45**
A_09_P009191	FBtr0086661	37144	CG5482	12.68	12.84	12.80	12.84	12.37	12.08	12.39	12.48	**-0.46**
A_09_P009196	FBtr0086658	37147	SP2637	10.15	10.56	10.41	10.50	9.89	10.01	9.95	10.00	**-0.44**
A_09_P009251	FBtr0086609	37163	Cyp12b2	8.18	9.14	8.59	9.21	7.44	7.31	7.63	7.78	**-1.24**
A_09_P009256	FBtr0299942	37164	CG42351	11.42	11.69	11.57	11.77	10.38	10.75	10.66	10.82	**-0.96**
A_09_P009266	FBtr0086639	37166	CG15093	15.71	15.63	15.91	15.68	15.45	15.49	15.50	15.39	**-0.27**
A_09_P009286	FBtr0086630	37172	CG15097	9.09	8.75	8.96	8.77	9.67	10.08	9.83	9.86	**0.97**
A_09_P009311	FBtr0086627	37177	CG15100	13.35	13.12	13.33	13.11	12.77	12.71	12.98	13.03	**-0.35**
A_09_P009326	FBtr0089970	37181	Jheh2	12.23	12.75	12.43	12.73	11.21	11.25	11.29	11.30	**-1.27**
A_09_P009341	FBtr0086566	37185	sano	7.26	7.74	7.15	6.81	9.59	9.64	8.83	8.75	**1.96**
A_09_P009356	FBtr0086572	37190	abba	14.06	14.48	14.67	14.47	13.92	13.88	13.78	13.76	**-0.58**
A_09_P009376	FBtr0086593	37197	CG15117	11.38	10.84	11.09	10.81	12.19	12.03	11.86	11.79	**0.94**
A_09_P009386	FBtr0086580	37200	CG15111	12.12	12.15	12.24	12.20	12.28	12.36	12.32	12.30	**0.14**
A_09_P009391	FBtr0100282	37202	CG10737	8.63	9.03	8.64	8.78	8.37	7.73	8.07	8.22	**-0.68**

A_09_P009416	FBtr0086562	37210	CG10476	11.31	12.78	11.52	12.46	8.03	8.63	8.43	8.63	-3.59
A_09_P009426	FBtr0300592	37212	CG18606	10.67	12.52	11.03	12.23	7.12	7.81	7.43	7.48	-4.15
A_09_P009431	FBtr0086514	37213	CG18607	10.99	11.16	11.09	11.08	9.53	9.55	9.21	8.75	-1.82
A_09_P009436	FBtr0086560	37215	mip40	11.05	11.13	11.28	11.29	10.70	10.79	10.84	10.94	-0.37
A_09_P009451	FBtr0334283	14462605	Rgk1	7.51	5.90	6.74	5.85	8.60	8.63	8.37	8.12	1.93
A_09_P009466	FBtr0086521	37222	CG10051	12.45	11.49	11.43	11.14	13.37	13.28	12.83	12.56	1.38
A_09_P009526	FBtr0085557	43571	Sry-alpha	7.86	8.68	8.25	8.58	7.15	7.53	7.38	7.58	-0.93
A_09_P009531	FBtr0085556	43570	Sry-beta	9.97	10.07	10.18	10.26	9.66	9.64	9.87	9.95	-0.34
A_09_P009546	FBtr0070877	31554	sqh	13.52	13.91	13.77	13.92	14.11	14.14	14.05	14.04	0.31
A_09_P009551	FBtr0075391	39836	st	10.63	10.68	10.78	10.84	9.95	10.14	10.27	10.47	-0.52
A_09_P009581	FBtr0077822	33379	Su(dx)	10.92	10.71	10.75	10.65	12.03	11.64	11.44	11.14	0.81
A_09_P009596	FBtr0082945	41740	su(Hw)	10.61	10.70	10.78	10.84	9.94	9.88	10.28	10.43	-0.60
A_09_P009621	FBtr0079635	34119	Su(var)205	12.62	13.00	12.85	12.88	12.52	12.15	12.50	12.57	-0.41
A_09_P009626	FBtr0088571	35927	Su(var)2-10	8.52	8.63	8.55	8.45	8.03	8.08	8.22	8.46	-0.34
A_09_P009631	FBtr0088575	35927	Su(var)2-10	7.23	7.51	7.15	7.32	6.57	6.58	6.93	6.99	-0.54
A_09_P009691	FBtr0088363	36080	Syb	10.56	10.64	10.63	10.67	10.48	10.35	10.37	10.41	-0.22
A_09_P009706	FBtr0071679	37476	Tbp	10.81	10.80	10.58	10.61	10.25	10.36	10.49	10.47	-0.31
A_09_P009741	FBtr0085059	43222	Tl	10.61	10.61	10.58	10.47	11.15	11.29	11.02	10.83	0.51
A_09_P009751	FBtr0085709	43656	tll	6.44	6.54	6.22	6.47	5.59	5.84	5.90	5.94	-0.60
A_09_P009766	FBtr0089960	41852	Tm1	11.57	11.69	11.67	11.58	11.36	11.23	11.33	11.36	-0.31
A_09_P009771	FBtr0089962	41852	Tm1	10.97	10.87	11.09	10.95	10.68	10.41	10.39	10.08	-0.58
A_09_P009806	FBtr0074922	40165	trc	9.07	8.99	8.88	8.85	8.36	8.44	8.64	8.82	-0.38
A_09_P009816	FBtr0071536	45368	Treh	12.50	12.24	12.08	11.99	13.99	13.65	13.34	12.85	1.25
A_09_P009821	FBtr0304120	38065	trh	9.54	8.63	8.96	8.49	9.69	9.84	9.85	9.82	0.90
A_09_P009836	FBtr0082947	41737	trx	9.89	10.37	9.94	10.26	10.82	10.81	10.55	10.46	0.54
A_09_P009841	FBtr0100277	41737	trx	9.62	10.29	9.70	9.57	11.17	11.31	10.48	10.96	1.19
A_09_P009866	FBtr0085829	48317	ttk	6.87	6.98	6.55	6.79	7.27	7.18	7.14	7.31	0.43
A_09_P009886	FBtr0081538	40904	alphaTub84D	13.42	13.47	13.53	13.55	13.17	13.26	13.28	13.36	-0.23
A_09_P009911	FBtr0300611	43359	betaTub97EF	14.80	14.09	14.53	14.21	15.06	15.05	15.02	14.83	0.58
A_09_P009936	FBtr0077470	33629	RpL40	15.67	15.56	15.65	15.53	15.70	15.79	15.79	15.73	0.15
A_09_P009941	FBtr0080016	34420	RpS27A	15.77	15.76	15.68	15.79	15.84	15.91	15.92	15.92	0.15

A_09_P009971	FBtr0070346	31165	usp	7.69	7.93	7.87	7.88	7.53	7.44	7.64	7.55	**-0.31**
A_09_P009996	FBtr0072037	47869	vir	9.72	9.86	9.81	9.89	9.19	9.22	9.46	9.58	**-0.46**
A_09_P010026	FBtr0070110	31003	vnd	4.62	3.63	3.84	3.22	5.70	5.70	5.63	5.06	**1.69**
A_09_P010057	FBtr0074520	32794	wupA	15.38	15.68	15.61	15.60	15.25	14.49	15.03	15.16	**-0.58**
A_09_P010066	FBtr0074524	32794	wupA	15.81	16.07	16.21	16.07	15.62	15.02	15.54	15.63	**-0.59**
A_09_P010116	FBtr0074748	32974	Zw	12.70	12.89	12.75	12.92	10.58	10.97	11.61	12.12	**-1.49**
A_09_P010121	FBtr0074210	32584	Pros28.1	14.05	14.11	14.18	14.11	13.11	13.07	13.56	13.85	**-0.72**
A_09_P010126	FBtr0110910	42003	Dhfr	10.90	10.84	10.89	11.00	9.87	10.24	10.29	10.43	**-0.70**
A_09_P010146	FBtr0080051	34411	cdc2	10.54	11.19	10.75	11.15	9.81	9.54	9.88	10.02	**-1.09**
A_09_P010151	FBtr0083922	42453	cdc2c	9.78	9.67	9.79	9.79	8.68	8.91	9.10	9.24	**-0.78**
A_09_P010166	FBtr0083078	41853	Tm2	16.67	16.60	16.72	16.79	16.02	15.67	16.10	16.27	**-0.68**
A_09_P010171	FBtr0084682	42920	nAcRbeta-96A	7.81	7.31	7.45	7.24	8.01	8.37	8.22	8.30	**0.77**
A_09_P010176	FBtr0088953	48986	boca	13.16	13.34	13.19	13.37	12.09	12.26	12.72	13.07	**-0.73**
A_09_P010196	FBtr0073072	38418	kst	11.60	11.66	11.63	11.68	12.69	12.68	12.32	12.12	**0.81**
A_09_P010201	FBtr0086577	37196	5-HT1A	7.67	7.28	7.23	6.96	8.52	8.68	8.36	8.27	**1.18**
A_09_P010206	FBtr0073853	32314	up	15.65	15.86	15.78	15.82	15.35	14.76	15.17	15.42	**-0.60**
A_09_P010246	FBtr0077641	33501	gammaTub23C	9.89	10.30	10.06	10.19	9.55	9.37	9.53	9.59	**-0.60**
A_09_P010291	FBtr0100586	32603	nonA	9.07	9.61	9.14	9.32	8.20	7.50	8.51	8.73	**-1.05**
A_09_P010296	FBtr0075602	39677	mex1	11.44	10.71	10.68	10.55	13.15	12.60	12.69	12.36	**1.85**
A_09_P010311	FBtr0086620	37183	Dpt	5.63	8.40	4.76	6.83	15.26	14.46	14.81	14.73	**8.41**
A_09_P010391	FBtr0303148	43469	Ptp99A	8.50	8.92	8.54	8.79	9.57	9.79	9.27	9.27	**0.79**
A_09_P010421	FBtr0306906	47216	Klp54D	5.74	4.84	5.04	4.71	6.03	6.29	6.28	6.40	**1.16**
A_09_P010436	FBtr0077157	38611	Klp64D	8.82	9.09	8.86	8.95	8.27	8.44	8.33	8.52	**-0.54**
A_09_P010471	FBtr0084402	42738	unk	10.00	10.62	10.11	10.29	11.05	11.07	10.75	10.73	**0.64**
A_09_P010476	FBtr0084403	42738	unk	6.56	7.08	6.75	6.85	7.54	7.63	7.28	7.14	**0.59**
A_09_P010506	FBtr0071095	47218	RpS14a	15.87	15.88	15.70	15.79	15.98	15.95	15.94	15.95	**0.14**
A_09_P010511	FBtr0071096	47219	RpS14b	14.53	14.53	14.49	14.63	14.93	14.88	14.87	14.81	**0.33**
A_09_P010521	FBtr0091627	3772064	NApol-gamma3	12.62	12.54	12.58	12.61	12.29	12.48	12.38	12.42	**-0.19**
A_09_P010586	FBtr0333101	36384	Galpha49B	7.73	7.54	7.72	7.70	8.25	7.83	8.03	8.13	**0.39**
A_09_P010626	FBtr0075359	39856	Su(P)	12.71	12.64	12.74	12.69	12.67	12.55	12.53	12.54	**-0.12**
A_09_P010631	FBtr0084115	42553	NApol-alpha18	11.09	11.26	11.14	11.38	10.77	10.75	10.90	11.05	**-0.35**

A_09_P010636	FBtr0077828	33386	GlyP	14.83	15.21	15.11	15.26	14.21	13.96	14.55	14.76	-0.73
A_09_P010646	FBtr0089983	47220	Fur1	6.72	6.92	6.63	5.80	8.05	8.35	7.28	7.90	1.38
A_09_P010651	FBtr0085088	43236	Ets97D	8.72	8.77	8.74	8.84	8.37	8.29	8.40	8.44	-0.39
A_09_P010666	FBtr0077011	38726	Mdr65	10.60	10.49	10.50	10.61	10.32	10.07	10.16	10.06	-0.40
A_09_P010676	FBtr0073275	38484	Gad1	8.82	8.39	8.64	8.36	9.28	9.44	9.33	9.54	0.84
A_09_P010681	FBtr0072215	49297	Ca-P60A	15.81	15.79	15.87	15.82	15.03	14.48	15.19	15.28	-0.83
A_09_P010711	FBtr0075408	39833	aos	6.87	6.75	6.59	6.80	7.32	7.69	7.31	7.48	0.70
A_09_P010731	FBtr0089475	41247	Syn	7.41	6.89	6.99	6.75	7.78	7.99	8.07	8.24	1.01
A_09_P010736	FBtr0089287	2768671	Pxd	8.49	9.49	8.52	8.97	5.69	6.69	6.78	6.78	-2.38
A_09_P010746	FBtr0087056	36905	Cbp53E	9.28	8.72	9.06	8.84	9.60	9.29	9.57	9.61	0.54
A_09_P010761	FBtr0077678	33500	Rrp1	9.42	10.24	9.86	10.21	9.24	9.11	9.14	9.13	-0.78
A_09_P010816	FBtr0083010	41801	CycC	10.92	10.86	10.97	10.99	10.24	10.41	10.47	10.56	-0.51
A_09_P010831	FBtr0085701	43650	zfh1	6.19	6.38	5.92	5.82	7.65	7.60	7.10	7.26	1.33
A_09_P010856	FBtr0076982	38742	Glu-RI	6.42	5.98	6.10	5.51	7.07	7.33	7.10	7.14	1.16
A_09_P010861	FBtr0079106	33788	GluRIIA	10.47	10.35	10.56	10.41	9.63	9.23	9.77	10.07	-0.77
A_09_P010866	FBtr0085507	43551	Takr99D	5.63	5.27	4.74	4.56	6.44	6.47	6.17	5.89	1.19
A_09_P010871	FBtr0074949	40148	Gbeta76C	6.60	6.15	6.21	5.95	7.09	6.97	7.06	7.10	0.83
A_09_P010906	FBtr0070441	47874	mit(1)15	10.32	10.68	10.51	10.70	10.06	10.08	10.17	10.34	-0.39
A_09_P010916	FBtr0071036	45382	ogre	9.25	9.56	9.38	9.40	8.46	8.84	8.87	9.23	-0.55
A_09_P010956	FBtr0083644	42226	fru	10.77	11.04	10.94	10.97	10.27	10.42	10.35	10.66	-0.51
A_09_P010971	FBtr0070384	31185	Pgd	14.80	14.67	14.82	14.60	13.02	13.40	13.75	14.20	-1.13
A_09_P011001	FBtr0070783	31474	Mlc-c	13.42	13.65	13.34	13.62	14.22	14.14	13.99	13.92	0.56
A_09_P011006	FBtr0087373	36697	mus210	9.88	10.25	10.10	10.26	11.04	11.21	10.96	10.91	0.91
A_09_P011056	FBtr0080917	44887	mdy	12.45	12.15	12.17	12.15	11.69	11.37	11.72	11.71	-0.61
A_09_P011066	FBtr0081317	49505	fs(2)ltoPP43	8.52	9.14	8.70	8.90	7.91	8.03	8.04	8.27	-0.75
A_09_P011106	FBtr0112789	41286	Takr86C	4.83	3.57	3.26	3.71	5.97	6.28	5.70	5.98	2.14
A_09_P011116	FBtr0074897	40180	Ac76E	11.10	11.48	11.40	11.46	10.52	10.48	10.30	10.08	-1.02
A_09_P011141	FBtr0089178	43767	ci	10.33	10.52	10.60	10.63	10.14	10.16	10.12	9.81	-0.46
A_09_P011181	FBtr0079862	34264	Gdi	14.98	15.11	15.11	14.99	14.63	14.51	14.72	14.93	-0.35
A_09_P011231	FBtr0078725	44018	cas	8.84	8.66	8.94	8.70	9.26	9.41	9.15	8.99	0.42
A_09_P011241	FBtr0073165	38469	scrt	9.24	9.57	9.22	9.48	10.46	10.75	10.30	10.29	1.07

A_09_P011261	FBtr0073151	38447	Scsalpha	13.92	14.12	14.06	14.13	13.87	13.78	13.75	13.80	**-0.26**
A_09_P011276	FBtr0077492	33602	bowl	10.58	10.86	10.71	10.81	11.23	11.42	11.16	11.03	**0.47**
A_09_P011281	FBtr0073192	38471	fd64A	8.89	10.24	10.42	9.97	8.55	8.56	8.55	8.42	**-1.36**
A_09_P011296	FBtr0084771	43011	fd96Cb	6.41	7.97	7.05	7.57	2.58	4.78	5.05	5.60	**-2.74**
A_09_P011311	FBtr0088412	36059	14-3-3zeta	11.62	11.35	11.28	11.40	11.90	11.76	11.96	12.00	**0.50**
A_09_P011331	FBtr0303422	47878	Eip63F-1	8.39	8.75	8.42	8.52	9.78	9.55	9.39	9.24	**0.97**
A_09_P011336	FBtr0100159	40607	Gnf1	9.65	9.98	9.94	9.99	9.47	9.55	9.54	9.71	**-0.32**
A_09_P011346	FBtr0080025	34430	TfIIB	10.37	10.31	10.57	10.51	10.10	9.73	10.05	10.00	**-0.47**
A_09_P011361	FBtr0088645	35881	Ggamma1	10.23	9.90	9.92	9.89	10.51	10.36	10.48	10.62	**0.51**
A_09_P011376	FBtr0100294	32458	Top1	10.23	10.13	10.09	10.27	9.57	9.74	9.77	9.72	**-0.48**
A_09_P011381	FBtr0074006	32458	Top1	10.97	10.83	11.12	10.80	10.39	10.40	10.27	10.16	**-0.63**
A_09_P011406	FBtr0074603	32857	phm	9.54	10.43	10.23	10.43	8.42	7.78	9.02	9.60	**-1.45**
A_09_P011456	FBtr0074234	32602	U2af50	11.93	12.00	12.02	12.06	12.15	12.20	12.18	12.24	**0.19**
A_09_P011476	FBtr0076479	39088	RpS17	16.28	16.24	16.26	16.28	16.41	16.41	16.41	16.39	**0.14**
A_09_P011506	FBtr0307325	32158	cac	9.35	9.01	8.99	9.15	10.15	10.26	10.13	10.49	**1.13**
A_09_P011521	FBtr0088257	36127	Rab3	9.12	8.84	9.07	8.76	9.38	9.52	9.49	9.52	**0.53**
A_09_P011556	FBtr0088473	36003	trpl	7.21	7.15	7.05	7.04	7.67	8.18	7.92	7.92	**0.81**
A_09_P011586	FBtr0304834	40515	nrm	10.71	10.57	10.87	10.89	9.63	9.73	9.63	9.85	**-1.05**
A_09_P011671	FBtr0085843	43749	faf	9.10	9.50	9.19	9.40	8.45	8.60	8.20	8.26	**-0.92**
A_09_P011681	FBtr0072399	38001	zip	11.90	12.23	12.01	12.17	12.71	12.68	12.52	12.52	**0.53**
A_09_P011696	FBtr0301275	44900	Larp7	10.16	10.35	10.33	10.35	9.29	9.34	9.73	9.95	**-0.72**
A_09_P011726	FBtr0086307	37290	mus209	11.97	11.99	12.01	12.08	10.90	10.73	11.28	11.57	**-0.89**
A_09_P011741	FBtr0078079	33229	Ets21C	4.27	4.71	3.04	4.05	8.24	8.48	7.32	6.64	**3.65**
A_09_P011751	FBtr0301340	43814	bt	15.42	15.70	15.90	15.71	14.73	14.62	15.05	15.20	**-0.78**
A_09_P011756	FBtr0070427	31188	Cyp4d1	14.78	15.30	15.11	15.12	13.58	13.59	14.22	14.47	**-1.11**
A_09_P011761	FBtr0070428	31188	Cyp4d1	14.18	14.76	14.41	14.66	12.77	12.84	13.41	13.70	**-1.32**
A_09_P011766	FBtr0082670	41550	Vha55	15.09	15.03	15.12	14.99	15.00	14.71	14.75	14.62	**-0.29**
A_09_P011771	FBtr0081268	35253	spi	10.80	11.12	10.92	11.20	11.65	11.59	11.57	11.67	**0.61**
A_09_P011801	FBtr0114543	43278	DNApol-alpha73	9.65	9.24	9.40	9.31	9.00	8.68	8.94	8.97	**-0.50**
A_09_P011816	FBtr0005088	2768940	Pp2A-29B	11.60	11.76	11.56	11.70	11.45	11.12	11.39	11.41	**-0.31**
A_09_P011821	FBtr0082128	49779	PpD3	9.22	9.09	9.32	9.01	8.80	8.76	8.93	8.97	**-0.29**

A_09_P011866	FBtr0070764	44317	CanB	8.50	8.48	8.43	8.35	8.90	9.07	8.91	8.85	0.49
A_09_P011871	FBtr0085732	43670	CanA1	9.29	9.63	9.59	9.54	7.86	7.62	8.02	8.23	-1.58
A_09_P011886	FBtr0082570	48336	GstD3	12.83	12.66	12.79	12.85	14.26	14.49	13.93	13.55	1.28
A_09_P011891	FBtr0082571	48337	GstD4	11.32	11.45	11.29	11.27	10.75	10.96	10.46	10.13	-0.76
A_09_P011906	FBtr0082574	48340	GstD7	13.65	13.46	13.64	13.45	12.45	13.07	12.94	13.07	-0.67
A_09_P011911	FBtr0082575	48341	GstD8	10.29	9.87	10.09	9.94	10.67	10.95	10.49	10.45	0.59
A_09_P011921	FBtr0087223	36780	Jhe	6.21	7.30	6.80	7.24	4.60	5.43	4.76	4.41	-2.09
A_09_P011926	FBtr0086625	251984	Jheh1	13.08	13.43	13.16	13.27	12.90	12.66	12.79	12.77	-0.45
A_09_P011931	FBtr0071897	37628	RpL23	15.82	15.85	15.71	15.87	15.95	15.98	15.94	15.98	0.15
A_09_P011946	FBtr0081473	44149	Acon	14.82	14.97	15.01	14.91	14.52	14.41	14.59	14.71	-0.37
A_09_P011971	FBtr0100119	35949	hig	8.71	8.39	8.52	8.37	9.59	9.81	9.50	9.57	1.12
A_09_P011976	FBtr0081867	40972	RpA-70	10.22	10.37	10.26	10.27	11.14	10.97	11.14	11.39	0.88
A_09_P011991	FBtr0073794	44150	RpS15Aa	15.77	15.81	15.76	15.72	15.89	15.92	15.89	15.92	0.14
A_09_P011996	FBtr0087078	36878	Sod2	13.36	13.34	13.36	13.31	12.50	12.36	12.74	13.10	-0.67
A_09_P012001	FBtr0070038	40517	alpha-Cat	11.34	11.58	11.39	11.63	11.26	11.00	11.23	11.22	-0.31
A_09_P012011	FBtr0089187	43829	ATPsyn-beta	15.52	15.32	15.60	15.34	14.89	14.34	15.08	15.20	-0.57
A_09_P012021	FBtr0088564	35917	Dbp45A	10.89	10.57	10.76	10.63	11.22	11.24	11.04	11.07	0.43
A_09_P012031	FBtr0075354	39861	Galpha73B	10.60	10.48	10.65	10.50	11.81	11.56	11.29	10.88	0.83
A_09_P012041	FBtr0087005	36927	GstS1	11.78	12.37	12.16	12.24	11.71	11.32	11.47	11.64	-0.60
A_09_P012071	FBtr0074129	32554	Lcch3	7.51	7.22	7.18	7.01	7.48	7.83	7.76	7.88	0.51
A_09_P012081	FBtr0072672	38153	Myo61F	14.38	13.97	14.08	13.85	15.60	15.38	15.21	14.89	1.20
A_09_P012091	FBtr0082275	41294	Rbp1	12.77	12.78	12.83	12.80	12.31	12.25	12.51	12.73	-0.34
A_09_P012121	FBtr0100468	33498	Rbp9	5.48	5.63	5.38	5.18	6.72	7.24	6.56	6.08	1.23
A_09_P012136	FBtr0071313	31872	Dsor1	11.54	11.86	11.58	11.82	11.39	11.33	11.31	11.42	-0.33
A_09_P012141	FBtr0072242	37767	Ssrp	11.27	11.36	11.26	11.36	11.69	11.74	11.99	12.19	0.59
A_09_P012161	FBtr0077844	33397	Uch	12.50	12.83	12.62	12.86	11.99	11.83	12.17	12.29	-0.63
A_09_P012171	FBtr0070540	31298	ng2	14.58	14.79	14.96	14.68	15.76	15.92	15.84	15.69	1.05
A_09_P012196	FBtr0073748	32256	hep	10.82	11.26	11.19	11.19	11.71	11.76	11.50	11.33	0.46
A_09_P012211	FBtr0079799	34250	Cks30A	7.65	8.15	7.88	8.50	7.26	7.16	6.86	7.26	-0.91
A_09_P012216	FBtr0300393	32551	CycD	8.32	8.41	8.21	8.25	7.74	7.82	7.81	8.07	-0.44
A_09_P012221	FBtr0088469	36001	dap	9.38	9.77	9.77	10.04	8.89	9.35	9.20	8.68	-0.71

A_09_P012246	FBtr0072730	38146	Rac1	11.73	11.97	11.99	11.59	11.52	11.45	11.27	11.49	**-0.39**
A_09_P012251	FBtr0300511	31957	alpha-Man-I	8.08	8.13	7.98	7.79	6.57	6.73	7.16	7.76	**-0.94**
A_09_P012271	FBtr0088530	35991	Map60	10.72	11.18	11.09	11.22	10.38	10.27	10.40	10.41	**-0.69**
A_09_P012276	FBtr0078573	40506	Arf79F	14.64	14.52	14.56	14.48	14.01	14.06	14.28	14.40	**-0.36**
A_09_P012336	FBtr0073073	38419	Drs	8.24	9.29	6.53	5.35	14.91	15.04	14.46	13.74	**7.18**
A_09_P012341	FBtr0080776	34924	CycE	9.41	9.74	9.71	9.64	8.87	9.01	8.83	8.99	**-0.70**
A_09_P012351	FBtr0088432	36047	Def	NA	2.78	2.73	NA	5.76	6.15	6.12	6.48	**3.37**
A_09_P012361	FBtr0309055	36635	Dro	NA	3.25	3.41	4.15	6.30	5.24	6.00	6.00	**2.28**
A_09_P012376	FBtr0081461	35368	betaInt-nu	6.25	6.09	5.94	5.37	6.82	6.81	6.78	6.70	**0.86**
A_09_P012386	FBtr0080092	34449	Lrr47	8.43	8.48	8.51	8.48	8.02	7.60	7.95	8.13	**-0.55**
A_09_P012416	FBtr0076423	39108	RpS9	16.14	16.22	16.00	16.14	16.30	16.29	16.24	16.29	**0.16**
A_09_P012431	FBtr0086947	36985	RpL18A	15.91	15.98	15.82	15.90	16.04	16.03	16.00	16.02	**0.12**
A_09_P012466	FBtr0082310	41290	TfIIFbeta	10.40	10.61	10.46	10.63	10.03	10.00	10.10	10.25	**-0.43**
A_09_P012471	FBtr0080722	34883	TfIIS	12.66	12.80	12.66	12.82	12.48	12.36	12.47	12.56	**-0.27**
A_09_P012476	FBtr0088184	36195	TpnC47D	16.19	16.24	16.12	16.20	16.00	16.01	16.04	16.09	**-0.15**
A_09_P012581	FBtr0086136	46017	l(2)01289	14.31	14.53	14.51	14.53	12.98	12.46	13.31	13.53	**-1.40**
A_09_P012586	FBtr0301423	46017	l(2)01289	11.79	12.03	12.09	12.05	10.86	10.77	10.91	10.96	**-1.12**
A_09_P012601	FBtr0072062	37729	Dcp-1	9.48	9.29	9.22	9.21	10.87	10.58	10.25	9.72	**1.05**
A_09_P012606	FBtr0088811	46027	kermit	10.70	10.39	10.70	10.70	11.77	11.48	11.33	10.98	**0.77**
A_09_P012611	FBtr0088082	36252	wal	13.62	13.87	13.86	13.86	13.49	13.32	13.48	13.63	**-0.32**
A_09_P012621	FBtr0300944	46035	CCS	11.08	11.21	11.12	11.33	10.45	10.55	10.63	10.72	**-0.60**
A_09_P012626	FBtr0308198	45398	Aldh-III	13.89	13.67	13.49	13.60	13.19	13.14	13.19	13.35	**-0.44**
A_09_P012631	FBtr0302504	47905	l(2)03659	10.91	10.63	10.80	10.66	11.10	11.33	11.34	11.39	**0.54**
A_09_P012636	FBtr0112863	46038	l(2)03709	13.81	13.56	13.82	13.61	13.24	13.15	13.34	13.46	**-0.40**
A_09_P012656	FBtr0087262	46058	Prosbeta1	13.07	13.12	13.06	13.14	12.49	12.46	12.79	12.89	**-0.44**
A_09_P012666	FBtr0078082	33226	lwr	12.31	12.46	12.46	12.48	12.06	12.01	12.09	12.00	**-0.39**
A_09_P012686	FBtr0080167	46069	l(2)06225	15.60	15.39	15.61	15.50	15.11	15.02	15.26	15.40	**-0.33**
A_09_P012696	FBtr0087987	36308	Cct5	13.81	13.79	13.71	13.71	13.52	13.27	13.39	13.55	**-0.32**
A_09_P012706	FBtr0086039	46078	vlc	9.62	9.80	9.81	9.77	9.27	9.04	9.30	9.38	**-0.50**
A_09_P012711	FBtr0087498	46080	Sec61beta	15.04	15.06	15.08	15.01	14.19	14.12	14.69	14.87	**-0.58**
A_09_P012726	FBtr0077840	33402	l(2)s5379	10.18	10.72	10.48	10.59	10.11	10.16	9.99	9.96	**-0.44**

A_09_P012731	FBtr0084590	42850	Atg6	11.19	11.35	11.29	11.30	11.60	11.69	11.52	11.60	0.32
A_09_P012736	FBtr0075945	39454	Atg1	9.70	9.69	9.65	9.56	11.34	11.50	10.84	10.55	1.41
A_09_P012746	FBtr0077169	38593	Srp54k	11.84	11.89	11.83	11.92	11.16	11.27	11.48	11.73	-0.46
A_09_P012776	FBtr0081642	44029	Ref1	13.05	13.11	13.13	13.19	12.81	12.74	12.93	13.02	-0.24
A_09_P012791	FBtr0081981	45399	Aats-trp	12.31	12.32	12.20	12.22	12.01	11.93	11.95	12.18	-0.24
A_09_P012801	FBtr0081788	44910	unc-45	11.95	11.91	11.93	11.76	10.98	11.25	11.29	11.48	-0.64
A_09_P012816	FBtr0076938	38760	sgl	11.35	10.57	10.77	10.62	12.18	11.91	11.86	11.37	1.00
A_09_P012821	FBtr0083595	47260	l(3)05822	11.53	11.55	11.46	11.45	12.65	12.48	12.18	11.79	0.78
A_09_P012826	FBtr0077136	46187	sinu	9.81	9.83	9.78	9.86	9.55	9.66	9.68	9.71	-0.17
A_09_P012836	FBtr0072956	44030	msn	8.16	7.93	7.77	7.74	9.20	9.46	8.98	8.83	1.22
A_09_P012886	FBtr0079847	34286	pelo	11.10	10.81	11.10	10.98	10.59	10.46	10.64	10.51	-0.45
A_09_P012896	FBtr0083018	41785	eff	11.89	11.84	11.98	11.81	11.39	11.03	11.32	11.37	-0.60
A_09_P012926	FBtr0087861	45401	ox	13.52	13.58	13.59	13.72	12.87	13.09	13.09	13.20	-0.54
A_09_P012941	FBtr0072129	37785	ken	10.05	9.24	9.48	9.02	10.12	10.46	10.58	10.91	1.07
A_09_P012951	FBtr0088452	47272	cbx	10.48	10.67	10.51	10.67	10.40	10.37	10.35	10.42	-0.19
A_09_P013001	FBtr0081008	35045	Dif	11.21	10.85	10.98	10.92	12.85	12.98	12.45	12.01	1.58
A_09_P013046	FBtr0075884	39484	RpS4	16.44	16.43	16.41	16.44	16.49	16.51	16.52	16.49	0.07
A_09_P013056	FBtr0088637	49090	Rya-r44F	12.68	12.88	12.94	12.90	11.63	11.84	12.06	12.33	-0.89
A_09_P013061	FBtr0111143	3355084	Snap25	8.41	7.85	7.96	7.97	8.76	8.62	8.86	8.96	0.76
A_09_P013076	FBtr0079924	34293	Taf11	10.44	10.55	10.63	10.54	10.30	10.40	10.26	10.31	-0.22
A_09_P013091	FBtr0072100	37744	l(2)efl	10.82	12.23	11.90	12.26	9.44	9.77	9.70	9.73	-2.14
A_09_P013096	FBtr0072095	37754	l(2)not	12.66	12.44	12.66	12.44	12.21	12.30	12.30	12.33	-0.26
A_09_P013111	FBtr0076472	39102	Uch-L3	11.84	12.06	12.00	12.04	10.94	10.79	11.12	11.33	-0.94
A_09_P013121	FBtr0084786	43005	OstStt3	14.42	14.61	14.50	14.43	13.43	13.61	13.80	14.33	-0.70
A_09_P013131	FBtr0072676	38154	mtacp1	14.60	14.33	14.63	14.44	14.05	14.05	14.21	14.21	-0.37
A_09_P013136	FBtr0078241	44915	DNApol-alpha6(	8.87	8.64	8.65	8.64	8.05	7.81	8.19	8.53	-0.55
A_09_P013151	FBtr0301089	41851	l(3)neo43	11.32	11.04	11.16	11.06	10.16	10.30	10.19	10.16	-0.94
A_09_P013161	FBtr0076598	47283	SrpRbeta	9.40	9.50	9.50	9.26	8.48	8.50	8.79	9.00	-0.73
A_09_P013166	FBtr0088163	36217	etaTry	12.90	12.62	12.72	12.75	13.42	13.41	13.18	12.90	0.48
A_09_P013201	FBtr0074217	32587	caz	11.00	11.17	10.90	11.04	10.30	10.12	10.50	10.66	-0.64
A_09_P013221	FBtr0084607	44324	Dms	8.87	8.17	8.76	8.47	9.34	9.48	9.42	9.18	0.79

A_09_P013236	FBtr0071266	43951	e(r)	12.73	12.95	12.94	13.01	12.43	12.47	12.54	12.48	-0.43
A_09_P013281	FBtr0081374	35305	La	13.52	13.14	13.38	13.24	13.84	13.95	13.94	14.14	0.65
A_09_P013286	FBtr0076845	38811	lark	12.44	12.42	12.50	12.54	12.18	12.25	12.36	12.32	-0.20
A_09_P013296	FBtr0299696	37853	Mlp60A	16.18	16.28	16.18	16.23	15.48	15.70	15.84	15.94	-0.48
A_09_P013326	FBtr0088447	36032	Mef2	5.89	5.19	5.33	5.13	6.43	7.06	6.55	6.24	1.18
A_09_P013346	FBtr0084882	43087	msi	7.72	7.93	7.61	7.83	7.25	7.31	7.17	7.10	-0.56
A_09_P013381	FBtr0100489	34495	Nos	6.48	5.59	5.78	5.53	7.39	8.11	7.42	7.21	1.69
A_09_P013386	FBtr0073227	38515	pav	8.76	9.75	9.24	9.61	8.58	8.24	8.33	8.33	-0.97
A_09_P013406	FBtr0083564	47285	repo	8.91	8.91	9.06	8.77	8.64	8.66	8.61	8.61	-0.28
A_09_P013421	FBtr0079782	34222	rost	8.94	8.66	8.11	8.23	10.26	10.19	9.58	9.16	1.31
A_09_P013451	FBtr0078682	40657	Snr1	11.34	11.53	11.50	11.59	11.07	11.25	11.18	11.29	-0.30
A_09_P013466	FBtr0332070	39349	byn	7.02	6.80	6.79	6.58	7.49	7.87	7.51	7.47	0.79
A_09_P013471	FBtr0084568	42880	twin	8.88	9.05	9.43	9.53	8.67	8.34	8.52	8.62	-0.68
A_09_P013491	FBtr0082031	41126	alpha-Man-II	9.12	9.60	9.46	9.61	8.35	8.30	8.69	9.03	-0.85
A_09_P013526	FBtr0079977	318911	CG31717	11.04	11.36	11.17	11.37	10.91	10.63	10.87	10.94	-0.40
A_09_P013541	FBtr0301974	318914	mthl15	6.05	4.56	5.16	4.14	7.78	7.55	7.16	6.77	2.34
A_09_P013556	FBtr0080461	34736	CG31729	9.48	9.69	9.75	9.62	9.42	9.47	9.30	9.31	-0.26
A_09_P013611	FBtr0081092	35118	CG31751	11.68	11.99	11.89	11.81	11.27	11.05	11.11	11.14	-0.70
A_09_P013636	FBtr0290240	326158	CG31759	8.92	8.52	8.72	8.44	8.12	8.19	8.13	8.35	-0.45
A_09_P013641	FBtr0331996	34642	CG31760	6.29	5.85	6.18	5.49	6.97	7.32	6.95	7.13	1.14
A_09_P013656	FBtr0080602	318934	CG31769	11.73	11.70	11.64	11.67	11.15	11.21	11.35	11.51	-0.38
A_09_P013671	FBtr0308126	33613	fred	4.68	3.86	3.35	3.34	5.56	6.08	5.22	5.45	1.77
A_09_P013741	FBtr0321311	35163	CG31792	12.74	12.74	12.78	12.81	12.08	12.09	12.33	12.40	-0.54
A_09_P013911	FBtr0308949	326165	CG42784	7.69	7.37	7.41	7.26	8.64	8.78	8.41	8.32	1.11
A_09_P013921	FBtr0080490	318981	CG31849	5.94	6.45	6.14	6.26	5.50	5.74	5.07	5.14	-0.84
A_09_P013966	FBtr0080288	318990	Qtzl	9.68	9.53	9.69	9.51	9.93	9.94	10.16	10.19	0.45
A_09_P013971	FBtr0080289	318991	Ada1-1	9.59	9.57	9.53	9.54	9.63	9.63	9.64	9.61	0.07
A_09_P014056	FBtr0079607	319014	Mur29B	11.61	11.30	11.45	11.44	12.71	12.15	12.29	11.98	0.83
A_09_P014161	FBtr0077630	319043	CG31950	11.91	11.74	11.77	11.80	10.67	10.92	11.28	11.56	-0.69
A_09_P014181	FBtr0077540	319045	CG31955	7.48	8.35	8.09	8.42	7.26	7.34	6.74	6.38	-1.15
A_09_P014186	FBtr0333248	261610	pgant4	10.28	9.95	10.31	10.04	9.11	9.12	9.01	9.26	-1.02

A_09_P014206	FBtr0077494	319047	CG31960	6.80	5.34	5.97	6.08	9.72	9.83	9.32	8.96	**3.41**
A_09_P014211	FBtr0077485	319048	CG31961	10.24	10.12	10.17	10.35	10.45	10.58	10.45	10.37	**0.24**
A_09_P014221	FBtr0330645	33174	CG31974	12.33	11.48	11.91	11.48	12.65	12.23	12.56	12.59	**0.71**
A_09_P014256	FBtr0089145	43774	CG31998	8.96	8.97	9.27	9.01	8.71	8.52	8.65	8.38	**-0.49**
A_09_P014261	FBtr0089144	43777	CG31999	11.64	12.37	12.02	12.13	11.01	10.65	10.97	11.04	**-1.12**
A_09_P014271	FBtr0113417	317815	CG32000	11.23	11.14	11.18	11.28	10.74	10.49	10.78	10.54	**-0.57**
A_09_P014281	FBtr0089233	43836	CG32016	10.65	10.55	10.77	10.72	10.34	10.10	10.28	10.16	**-0.45**
A_09_P014286	FBtr0300992	317823	CG32017	10.17	10.25	10.14	9.98	10.56	10.54	10.59	10.70	**0.46**
A_09_P014361	FBtr0076440	317835	CG32040	5.72	5.08	4.82	4.83	6.81	7.34	6.84	6.83	**1.84**
A_09_P014376	FBtr0299606	39145	CG42268	9.27	8.87	9.15	8.95	9.95	9.89	9.61	9.35	**0.64**
A_09_P014381	FBtr0302710	39111	CG42673	8.36	7.98	8.29	7.73	9.29	9.15	9.00	8.41	**0.88**
A_09_P014386	FBtr0302710	39111	CG42673	9.74	9.42	9.72	9.38	10.96	10.80	10.48	10.20	**1.04**
A_09_P014401	FBtr0076314	326185	CG32054	9.55	8.72	8.94	8.41	11.87	10.84	11.13	10.61	**2.20**
A_09_P014436	FBtr0305093	39198	A2bp1	9.69	10.13	9.90	10.04	9.57	9.49	9.42	9.37	**-0.48**
A_09_P014471	FBtr0076241	39235	CG32069	10.90	10.56	10.64	10.65	9.03	9.44	9.91	10.24	**-1.04**
A_09_P014486	FBtr0076230	326191	CG32073	13.19	14.34	14.57	14.66	10.36	11.63	12.01	12.41	**-2.59**
A_09_P014501	FBtr0076217	326193	Alg10	10.78	10.96	10.84	10.99	9.92	9.75	10.21	10.48	**-0.81**
A_09_P014566	FBtr0076077	317849	CG32095	9.84	9.91	9.98	10.06	9.31	9.41	9.55	9.78	**-0.44**
A_09_P014691	FBtr0075758	39537	CG32137	11.79	12.12	11.71	12.01	12.95	13.21	12.61	12.38	**0.88**
A_09_P014706	FBtr0299649	44297	ome	6.78	7.09	6.74	6.86	5.49	6.42	5.85	6.27	**-0.86**
A_09_P014771	FBtr0075285	39919	Rbp6	7.37	7.56	7.07	6.74	8.10	8.62	7.71	8.47	**1.04**
A_09_P014781	FBtr0075258	317894	CG32170	10.10	9.63	9.71	9.84	11.83	12.14	11.50	10.91	**1.78**
A_09_P014786	FBtr0075227	261607	CG32174	10.11	10.13	10.17	9.94	9.28	9.04	9.42	9.73	**-0.72**
A_09_P014801	FBtr0075197	326198	Krn	7.37	7.21	6.97	7.05	7.93	7.96	7.67	7.40	**0.59**
A_09_P014816	FBtr0075186	39972	Ccn	6.77	6.71	6.30	6.14	7.03	7.58	7.20	7.10	**0.75**
A_09_P014826	FBtr0075174	317900	edin	4.99	6.62	5.35	5.85	15.51	14.74	15.02	14.64	**9.28**
A_09_P014851	FBtr0075171	39978	NUCB1	12.48	12.83	12.79	12.80	11.69	11.48	12.04	12.17	**-0.88**
A_09_P014876	FBtr0273401	317908	CG32196	9.99	9.87	9.94	9.98	9.34	9.32	9.50	9.60	**-0.50**
A_09_P014906	FBtr0113429	317913	CG32204	7.11	6.65	6.48	6.02	7.56	7.68	7.41	7.22	**0.90**
A_09_P014956	FBtr0074911	317923	Csas	8.41	8.53	8.37	8.49	7.52	7.75	7.80	8.13	**-0.65**
A_09_P014976	FBtr0074848	40225	gogo	5.24	5.63	5.36	5.60	6.77	6.14	6.16	6.39	**0.91**

A_09_P014986	FBtr0070040	317928	CG32230	14.64	14.49	14.51	14.61	13.99	13.97	14.25	14.42	-0.40
A_09_P015046	FBtr0333585	38562	CG42540	7.69	6.76	7.00	6.65	8.10	7.88	8.27	8.27	1.11
A_09_P015076	FBtr0073286	326205	Claspin	9.97	10.13	10.26	10.01	9.37	9.60	9.56	9.53	-0.58
A_09_P015191	FBtr0073037	317956	CG32284	13.83	13.01	13.32	13.04	14.87	14.89	14.49	13.77	1.21
A_09_P015226	FBtr0072881	38293	CG32302	12.65	13.77	13.04	13.72	16.04	15.28	15.68	15.15	2.24
A_09_P015231	FBtr0072880	317966	obst-I	11.33	11.73	11.72	11.66	10.58	9.67	9.84	9.47	-1.72
A_09_P015261	FBtr0332119	2769001	zormin	12.54	12.71	12.69	12.79	13.63	13.31	13.44	13.35	0.75
A_09_P015271	FBtr0072732	2768976	CG32318	8.09	8.11	8.07	7.98	7.55	7.46	7.61	7.89	-0.44
A_09_P015336	FBtr0340707	38981	CG43078	13.29	13.73	13.66	13.76	13.02	12.90	12.92	12.98	-0.65
A_09_P015341	FBtr0076720	317996	CG32364	6.55	6.77	6.51	6.59	8.36	8.66	7.81	8.05	1.62
A_09_P015346	FBtr0076746	38876	CG32365	6.83	5.87	5.85	5.46	7.37	7.44	7.42	7.82	1.51
A_09_P015351	FBtr0076749	317997	CG32368	8.24	8.38	8.55	8.63	10.64	10.81	10.44	10.30	2.10
A_09_P015371	FBtr0076806	318000	CG32373	11.20	10.95	10.77	10.91	12.01	11.78	12.10	12.32	1.10
A_09_P015381	FBtr0076827	318002	CG32376	6.56	3.13	3.58	NA	NA	NA	8.32	9.05	4.26
A_09_P015396	FBtr0076870	38823	SMSr	8.26	8.16	8.15	7.90	8.83	8.74	8.58	8.47	0.54
A_09_P015471	FBtr0077037	38677	CG32406	7.35	6.77	7.13	6.71	8.30	8.36	8.06	7.94	1.17
A_09_P015476	FBtr0077032	38667	CG32407	11.43	11.80	11.38	11.27	10.61	10.24	10.32	10.29	-1.10
A_09_P015531	FBtr0078268	40310	CG32432	6.79	6.59	6.46	6.50	7.35	7.81	7.45	7.20	0.87
A_09_P015581	FBtr0303248	318032	CG32448	11.49	11.48	11.57	11.66	11.11	11.23	11.25	11.29	-0.33
A_09_P015596	FBtr0078523	318035	CG32452	8.34	8.14	8.29	8.20	7.48	7.84	7.92	8.02	-0.43
A_09_P015661	FBtr0072589	38103	CG32479	11.69	11.88	11.71	11.75	11.51	11.48	11.51	11.50	-0.26
A_09_P015671	FBtr0072984	326219	Sk2	9.13	9.62	9.46	9.63	8.94	8.99	8.88	9.11	-0.48
A_09_P015676	FBtr0072985	38363	CG32485	9.29	8.74	9.12	8.93	10.53	10.34	10.00	9.61	1.10
A_09_P015711	FBtr0077374	2768879	CG32500	12.11	12.05	12.20	12.20	12.26	12.32	12.32	12.34	0.17
A_09_P015806	FBtr0340421	32822	CG32549	11.47	11.56	11.36	11.45	11.67	11.78	11.87	11.99	0.37
A_09_P015831	FBtr0074466	326221	CG32557	11.54	11.68	11.79	11.86	13.31	12.90	12.77	12.36	1.12
A_09_P015841	FBtr0340632	32754	CG42684	10.19	10.71	10.41	10.69	11.29	11.39	10.99	11.04	0.67
A_09_P015851	FBtr0074390	326222	CG32564	14.94	14.41	14.58	14.58	15.66	15.67	15.51	15.26	0.90
A_09_P015871	FBtr0074325	318094	TwdlX	12.23	12.63	12.80	12.47	11.25	11.76	11.65	11.74	-0.93
A_09_P015961	FBtr0073913	32387	dpr8	7.73	7.50	7.50	7.21	8.14	8.78	8.42	8.32	0.93
A_09_P015996	FBtr0073839	318128	CG32625	5.86	5.71	5.31	5.45	6.46	6.33	6.52	6.86	0.96

A_09_P016021	FBtr0073800	326225	CG32633	11.05	10.70	10.85	10.50	12.04	11.92	11.58	11.20	0.91
A_09_P016066	FBtr0073703	326226	CG32647	9.68	8.75	9.29	9.20	10.32	10.71	10.30	10.32	1.18
A_09_P016106	FBtr0113445	32209	Sec16	10.12	10.37	10.27	10.23	9.53	9.69	9.80	10.12	-0.46
A_09_P016136	FBtr0073474	318146	CG32667	13.91	14.06	14.14	14.34	12.45	12.87	13.02	13.21	-1.22
A_09_P016181	FBtr0071493	31997	X11Lbeta	8.71	8.98	8.72	8.96	9.45	10.07	9.55	9.51	0.80
A_09_P016196	FBtr0299633	31957	alpha-Man-I	6.56	6.98	6.48	6.82	4.99	5.36	5.66	5.80	-1.26
A_09_P016261	FBtr0071386	31915	CG32698	7.96	10.40	9.64	10.30	4.00	5.30	4.41	4.84	-4.94
A_09_P016296	FBtr0071285	31835	APC4	6.92	7.28	7.13	7.02	6.10	6.37	6.45	6.68	-0.69
A_09_P016351	FBtr0071039	31638	CG32732	11.26	11.16	11.28	10.97	10.81	10.74	10.62	10.78	-0.42
A_09_P016361	FBtr0071008	50395	CG32736	12.78	12.42	12.78	12.58	11.94	12.30	12.17	12.23	-0.48
A_09_P016376	FBtr0070881	326238	CG32750	8.45	8.16	7.98	7.48	8.83	8.94	8.65	8.49	0.71
A_09_P016436	FBtr0301099	31344	CG42541	6.03	5.59	5.45	4.78	6.55	7.21	6.42	6.60	1.23
A_09_P016451	FBtr0070615	318207	CG32783	6.99	6.92	7.14	7.13	6.68	6.67	6.55	6.67	-0.40
A_09_P016456	FBtr0070614	318210	CG32786	7.27	7.33	7.34	7.32	6.79	6.68	7.00	7.11	-0.42
A_09_P016461	FBtr0070600	318211	HIP	13.30	13.49	13.22	13.61	12.68	12.46	12.74	12.82	-0.73
A_09_P016496	FBtr0070356	326240	CG32803	10.85	11.59	11.30	11.62	10.24	10.53	10.45	10.55	-0.90
A_09_P016591	FBtr0087755	36475	Dh31-R1	7.75	7.63	7.56	7.55	8.95	8.42	8.46	8.26	0.90
A_09_P016626	FBtr0083239	318251	CG32856	9.76	9.93	10.00	10.07	9.11	9.29	9.39	9.54	-0.61
A_09_P016636	FBtr0339973	40560	CG32944	5.06	4.86	4.91	4.98	6.04	6.21	5.93	5.83	1.05
A_09_P016706	FBtr0332249	36342	CG33012	10.79	11.29	11.27	11.32	10.26	9.15	9.56	9.59	-1.53
A_09_P016741	FBtr0078311	326246	CG33056	9.29	9.53	9.41	9.74	9.81	9.85	9.85	9.80	0.34
A_09_P016746	FBtr0076678	318833	CG33057	9.32	9.58	9.45	9.62	9.98	10.15	9.91	9.96	0.51
A_09_P016771	FBtr0331380	35799	LRP1	8.85	8.92	9.02	8.91	7.70	8.05	8.10	8.13	-0.93
A_09_P016781	FBtr0084287	42654	CG33093	5.76	5.66	5.63	6.47	6.51	7.15	6.89	7.17	1.05
A_09_P016791	FBtr0100204	43012	CG33095	11.04	11.30	11.19	11.25	10.70	10.69	10.84	11.00	-0.39
A_09_P016811	FBtr0084288	326254	CG33099	10.61	11.07	11.05	11.26	10.36	10.34	10.30	10.26	-0.68
A_09_P016826	FBtr0084279	326256	CG33107	8.16	7.69	7.91	7.74	9.03	9.11	8.69	8.42	0.94
A_09_P016831	FBtr0084504	326257	CG33108	9.52	9.50	9.53	9.54	9.10	9.33	9.29	9.35	-0.26
A_09_P016851	FBtr0079038	33721	Rtnl1	15.10	15.22	15.23	15.26	14.32	14.27	14.59	14.67	-0.74
A_09_P016866	FBtr0081118	326260	CG33120	8.44	9.88	9.27	9.64	5.82	6.94	6.90	7.24	-2.58
A_09_P016906	FBtr0080169	34514	CG33129	13.58	13.83	13.83	13.65	11.95	11.50	12.69	12.93	-1.45

A_09_P016921	FBtr0087732	326264	CG33138	13.96	14.32	14.43	14.35	12.67	12.92	13.04	13.23	-1.30
A_09_P016936	FBtr0071858	37583	CG33143	7.10	6.97	6.70	6.58	7.49	7.41	7.43	7.52	0.62
A_09_P016946	FBtr0088048	36279	CG33145	8.11	7.82	8.01	7.74	6.97	6.86	7.03	7.23	-0.90
A_09_P016951	FBtr0086608	37161	Hs3st-A	5.86	5.29	5.52	5.15	6.83	6.53	6.47	6.37	1.09
A_09_P016966	FBtr0075390	39834	CG33158	10.33	10.22	10.44	10.19	11.58	11.51	11.58	11.57	1.27
A_09_P016991	FBtr0089836	326267	CG33170	11.81	12.00	12.03	12.24	11.56	11.80	11.55	11.49	-0.42
A_09_P017156	FBtr0299851	39696	comm3	6.46	6.53	6.34	5.81	7.50	7.13	7.29	6.81	0.90
A_09_P017181	FBtr0085271	338395	CG33213	7.61	7.80	7.72	8.02	7.20	7.19	7.27	7.54	-0.49
A_09_P017201	FBtr0342899	2768860	CG33225	7.86	7.41	7.58	7.71	9.50	10.37	9.45	9.22	1.99
A_09_P017211	FBtr0072499	2768996	CG33229	10.02	9.76	9.58	9.67	10.78	10.83	10.54	10.34	0.86
A_09_P017291	FBtr0299614	2768892	CG42271	10.50	10.82	10.76	10.86	10.47	10.40	10.19	10.23	-0.41
A_09_P017296	FBtr0299615	2768892	CG42271	9.18	9.29	9.27	9.30	9.00	9.14	9.04	9.13	-0.18
A_09_P017351	FBtr0305649	2768969	CG33267	8.31	6.80	5.97	5.66	11.24	11.02	10.78	9.70	4.00
A_09_P017371	FBtr0114530	2768949	CG33276	9.76	9.57	9.71	9.62	8.78	8.88	9.09	9.33	-0.64
A_09_P017396	FBtr0113458	2768939	CG33282	9.17	10.35	9.79	10.42	7.20	7.15	7.67	8.02	-2.42
A_09_P017476	FBtr0079935	2768917	CG33301	10.87	10.97	11.06	11.30	10.45	10.39	10.19	10.12	-0.76
A_09_P017491	FBtr0080564	2768915	CG33306	13.42	12.64	12.67	12.57	14.43	14.33	14.16	13.77	1.35
A_09_P017496	FBtr0343672	2768914	CG33307	12.89	13.32	13.06	13.30	12.03	12.26	12.40	12.71	-0.80
A_09_P017551	FBtr0304612	2768678	CG33337	5.66	3.73	3.96	4.13	10.40	9.76	9.64	8.93	5.31
A_09_P017606	FBtr0089859	2768847	CG33459	10.85	11.26	11.04	11.51	9.73	10.05	10.09	10.37	-1.10
A_09_P017611	FBtr0100642	3885588	CG33460	9.25	9.91	9.65	9.93	8.30	8.49	8.64	8.82	-1.12
A_09_P017616	FBtr0302528	2768840	CG33461	7.61	7.89	7.64	8.14	7.12	7.23	7.13	7.31	-0.62
A_09_P017646	FBtr0087503	2768833	CG33468	6.21	5.87	5.69	5.48	8.41	8.39	7.96	7.73	2.31
A_09_P017651	FBtr0087504	2768832	CG33469	7.46	7.15	6.85	6.73	8.58	8.43	8.32	8.00	1.28
A_09_P017716	FBtr0077344	33128	CG14621	7.86	7.89	7.78	7.89	6.93	6.96	7.27	7.40	-0.72
A_09_P017721	FBtr0077369	33129	CG14615	8.48	6.91	7.50	6.81	9.04	9.28	8.85	8.75	1.55
A_09_P017731	FBtr0077348	33132	CG14619	9.83	9.99	10.10	9.90	9.43	9.44	9.26	9.54	-0.54
A_09_P017756	FBtr0077364	33140	CG17598	10.91	11.19	11.07	11.07	10.74	10.52	10.80	10.85	-0.33
A_09_P017801	FBtr0078103	33165	Zir	10.60	10.81	10.73	10.85	10.62	10.41	10.46	10.33	-0.29
A_09_P017806	FBtr0078104	33166	CG11377	11.83	11.65	11.81	11.56	10.82	10.46	11.23	11.48	-0.72
A_09_P017811	FBtr0078160	33169	CG13694	9.42	9.40	9.79	9.44	8.83	9.19	8.80	8.55	-0.67

A_09_P017841	FBtr0078118	33179	CG11455	13.85	13.86	13.90	14.02	13.13	13.22	13.35	13.58	-0.59
A_09_P017846	FBtr0078119	33180	CG3436	11.91	11.89	11.86	11.89	11.41	11.27	11.54	11.71	-0.41
A_09_P017861	FBtr0078146	33183	CG11617	10.08	10.05	10.16	9.85	8.41	8.66	8.97	9.28	-1.21
A_09_P017901	FBtr0330650	33195	CG17078	9.16	9.17	9.11	9.27	8.19	8.24	8.75	8.77	-0.69
A_09_P017906	FBtr0078137	33197	CG11601	9.72	9.45	9.56	9.51	10.16	10.01	9.95	9.90	0.45
A_09_P017911	FBtr0078136	33198	CG3625	11.34	11.63	11.40	11.49	10.95	10.53	10.70	10.75	-0.73
A_09_P017916	FBtr0078130	33200	CG11562	10.18	10.09	10.24	10.04	9.66	9.89	9.72	9.83	-0.36
A_09_P017926	FBtr0078098	33206	CG11911	15.45	15.41	14.92	14.93	15.85	15.77	15.73	15.65	0.57
A_09_P017941	FBtr0078090	33213	CG13690	7.69	7.68	7.62	7.57	6.14	6.44	6.71	6.95	-1.08
A_09_P017961	FBtr0078060	33220	CG4164	14.04	14.18	14.15	14.05	12.95	12.82	13.50	13.90	-0.81
A_09_P017966	FBtr0078061	33221	CG4133	7.75	7.59	7.67	7.54	7.83	8.17	7.96	8.07	0.37
A_09_P017991	FBtr0078066	33231	CG2789	14.47	14.72	14.67	14.81	15.32	15.55	15.26	14.95	0.60
A_09_P017996	FBtr0078076	33232	CG11835	9.83	11.37	10.92	11.22	8.30	8.15	8.45	8.75	-2.42
A_09_P018011	FBtr0078074	33236	Ipk2	10.43	10.86	10.70	10.89	11.58	11.52	11.19	11.00	0.60
A_09_P018031	FBtr0078001	33248	GABA-B-R3	6.37	5.82	5.95	5.65	6.87	7.36	6.91	6.96	1.08
A_09_P018036	FBtr0078003	33249	CG12506	8.10	8.79	8.35	8.10	9.05	9.80	9.35	9.30	1.04
A_09_P018101	FBtr0301416	33277	IA-2	8.09	7.92	7.81	7.62	9.24	9.14	8.87	9.01	1.20
A_09_P018106	FBtr0301416	33277	IA-2	10.74	10.27	10.05	10.09	11.50	11.70	11.48	11.92	1.36
A_09_P018156	FBtr0077957	33291	CG4577	8.56	8.00	8.42	8.19	9.09	9.03	8.89	8.61	0.61
A_09_P018196	FBtr0077964	33301	CG14341	11.33	11.60	11.45	11.75	10.80	10.79	11.07	11.30	-0.54
A_09_P018201	FBtr0077979	33303	CG5118	9.77	9.79	9.70	9.95	11.53	10.61	11.08	10.87	1.22
A_09_P018216	FBtr0077978	33306	CG5126	11.16	11.38	11.22	11.25	10.77	10.69	10.82	11.04	-0.42
A_09_P018221	FBtr0077971	33307	Tgt	10.57	10.07	10.43	10.08	10.75	10.89	10.71	10.71	0.47
A_09_P018226	FBtr0077972	33308	CG5001	12.85	13.20	13.15	13.21	12.64	12.49	12.48	12.31	-0.62
A_09_P018386	FBtr0077813	33369	Der-1	12.94	12.99	12.98	13.00	11.81	11.93	12.44	12.75	-0.75
A_09_P018401	FBtr0077815	33372	CG7289	11.78	11.82	11.86	11.93	11.58	11.57	11.61	11.61	-0.25
A_09_P018426	FBtr0077853	326153	tho2	9.08	9.47	9.47	9.46	8.91	8.90	8.92	9.02	-0.43
A_09_P018431	FBtr0077852	33388	CG11723	9.31	9.48	9.45	9.36	9.19	9.08	9.11	9.32	-0.23
A_09_P018436	FBtr0330657	33390	AIF	11.59	11.13	11.37	11.01	10.67	10.58	10.71	10.84	-0.58
A_09_P018456	FBtr0077845	33396	CG15385	8.53	9.40	9.14	9.21	7.78	7.71	8.24	8.31	-1.06
A_09_P018466	FBtr0077835	33399	mio	10.63	10.92	10.74	11.05	11.66	11.74	11.36	11.18	0.65

A_09_P018476	FBtr0113015	33403	CG15387	8.13	8.47	8.22	8.50	7.56	7.47	7.83	8.10	-0.59
A_09_P018496	FBtr0077810	33409	gho	12.63	12.85	12.82	12.88	12.01	12.10	12.37	12.68	-0.51
A_09_P018531	FBtr0077791	33421	CG3609	15.10	15.21	15.17	15.20	14.53	14.57	14.67	14.85	-0.51
A_09_P018546	FBtr0077767	33424	Eogt	11.22	11.81	11.55	11.90	10.67	11.01	10.84	10.77	-0.80
A_09_P018551	FBtr0077768	33425	CG9870	9.17	8.99	9.01	9.03	7.90	7.78	8.43	8.76	-0.83
A_09_P018561	FBtr0077769	33427	VGlut	9.23	8.84	9.03	8.84	9.46	9.35	9.55	9.58	0.50
A_09_P018566	FBtr0307079	33429	CG18641	7.79	4.11	6.67	4.57	8.55	8.88	8.51	8.40	2.80
A_09_P018606	FBtr0077745	33443	CG3214	14.10	14.11	14.18	14.22	13.49	13.62	13.78	14.02	-0.43
A_09_P018611	FBtr0077694	33444	Arpc5	12.91	13.05	12.94	13.06	13.33	13.30	13.16	13.16	0.25
A_09_P018616	FBtr0335172	33446	CG15394	7.77	6.75	7.06	7.11	9.35	9.68	9.27	9.05	2.17
A_09_P018656	FBtr0089750	33458	Hrs	11.93	12.29	12.11	12.22	12.66	12.75	12.47	12.36	0.42
A_09_P018681	FBtr0077715	33465	Trn-SR	9.41	9.38	9.35	9.21	8.70	8.80	8.97	9.22	-0.41
A_09_P018706	FBtr0077721	33474	daw	11.89	11.87	11.97	11.84	11.36	11.29	11.35	11.45	-0.53
A_09_P018741	FBtr0077619	33481	CG2975	4.80	4.46	3.99	3.94	6.05	6.64	6.00	5.67	1.79
A_09_P018756	FBtr0300356	33484	CG3117	6.66	7.24	6.68	7.32	6.00	5.85	5.68	5.36	-1.25
A_09_P018811	FBtr0077671	33510	CG17224	10.35	10.84	10.70	10.80	9.53	9.25	9.76	9.87	-1.07
A_09_P018821	FBtr0077670	33512	alpha4GT1	9.24	9.49	9.47	9.58	8.72	8.83	8.86	8.83	-0.63
A_09_P018851	FBtr0077667	33518	CG17259	13.90	13.88	13.84	13.90	13.79	13.76	13.76	13.81	-0.10
A_09_P018866	FBtr0077655	33521	CG17221	12.32	12.75	12.42	12.60	11.42	11.56	11.76	12.03	-0.83
A_09_P018881	FBtr0077617	33528	CG12400	14.44	14.38	14.47	14.50	13.90	14.14	14.09	14.22	-0.36
A_09_P018921	FBtr0114460	33543	CG3277	7.88	7.27	7.36	6.82	9.15	8.74	8.53	8.15	1.31
A_09_P018941	FBtr0077587	33548	CG15408	10.74	11.58	11.28	11.59	9.42	9.38	9.79	10.01	-1.64
A_09_P018971	FBtr0077516	33559	CG2772	10.63	9.63	9.81	9.15	13.06	12.44	12.11	11.23	2.41
A_09_P018976	FBtr0077517	33560	Snx1	10.71	11.07	10.90	11.01	10.69	10.46	10.48	10.43	-0.41
A_09_P018981	FBtr0113019	33561	CG12795	7.71	7.96	7.83	7.68	6.67	6.65	6.92	7.24	-0.92
A_09_P019036	FBtr0301590	33578	Spindly	7.26	7.69	7.61	7.81	7.21	6.95	6.92	6.80	-0.62
A_09_P019061	FBtr0077547	33591	CG16713	11.82	11.99	11.75	12.06	12.21	12.36	12.20	12.24	0.35
A_09_P019066	FBtr0077546	33592	CG16712	14.94	15.02	15.07	15.06	15.21	15.25	15.12	15.19	0.17
A_09_P019076	FBtr0077544	33594	CG10031	11.51	10.69	11.12	10.79	11.56	11.62	11.58	11.67	0.58
A_09_P019101	FBtr0077515	33606	CG3407	8.44	8.48	8.35	8.48	7.78	7.99	8.01	8.14	-0.46
A_09_P019116	FBtr0077505	33615	CG15422	7.20	6.89	6.59	6.51	8.37	8.11	7.78	7.36	1.11

A_09_P019126	FBtr0331498	33617	CG43773	8.96	9.31	9.66	9.52	8.60	8.87	8.64	8.13	-0.80
A_09_P019141	FBtr0077477	33628	CG3702	13.35	13.33	13.18	13.15	12.88	12.81	12.82	13.07	-0.36
A_09_P019171	FBtr0331449	33641	Psf2	9.50	10.01	9.76	9.91	8.77	8.99	9.06	9.30	-0.77
A_09_P019176	FBtr0077460	33643	CG3652	10.99	10.86	10.89	11.06	10.32	10.26	10.54	10.65	-0.51
A_09_P019181	FBtr0077435	33645	Dim1	11.13	11.11	11.11	11.22	10.54	10.55	10.82	10.99	-0.42
A_09_P019191	FBtr0077438	33647	CG15432	10.54	10.77	10.63	10.94	10.24	10.13	10.38	10.50	-0.41
A_09_P019206	FBtr0077454	33652	CG15440	9.86	9.90	9.85	10.00	9.61	9.72	9.68	9.82	-0.19
A_09_P019286	FBtr0077383	33679	CG15629	11.78	10.88	10.83	10.89	12.52	12.65	12.48	12.60	1.47
A_09_P019336	FBtr0077387	33689	CG11926	10.83	11.06	10.95	10.98	10.23	10.45	10.55	10.71	-0.47
A_09_P019341	FBtr0077389	33693	CG3008	11.29	11.31	11.40	11.17	12.31	12.51	12.29	12.26	1.05
A_09_P019396	FBtr0079052	33711	Scox	13.57	13.41	13.40	13.35	12.97	12.96	13.03	13.19	-0.39
A_09_P019406	FBtr0078995	33714	CG14043	11.34	11.30	11.29	11.38	11.05	10.93	11.00	11.06	-0.32
A_09_P019441	FBtr0079000	33725	CG3887	12.47	12.62	12.57	12.51	12.28	12.04	12.33	12.31	-0.30
A_09_P019476	FBtr0079018	33742	CG5828	11.25	11.66	11.36	11.59	10.98	10.78	11.01	11.07	-0.50
A_09_P019486	FBtr0079025	33744	CG8680	13.62	13.51	13.55	13.68	12.79	12.84	13.12	13.25	-0.59
A_09_P019501	FBtr0079062	33749	Cyp28d1	11.40	11.92	11.84	11.85	12.76	12.91	12.61	12.48	0.94
A_09_P019516	FBtr0079063	33752	TpnC25D	14.51	14.86	14.94	14.90	14.18	14.22	14.09	14.10	-0.65
A_09_P019521	FBtr0079066	33754	Cyp4ac1	12.00	12.11	11.93	12.10	11.41	11.21	11.54	11.69	-0.57
A_09_P019531	FBtr0079068	33756	Cyp4ac3	6.95	7.64	6.87	7.57	9.89	9.08	9.07	8.56	1.89
A_09_P019541	FBtr0079082	33760	CG14024	7.93	8.33	8.19	7.76	8.88	9.29	8.49	8.77	0.81
A_09_P019576	FBtr0079130	33772	CG7382	11.89	12.01	11.89	11.99	11.36	11.18	11.50	11.65	-0.52
A_09_P019671	FBtr0079169	33800	CG11149	10.12	9.65	9.58	9.31	11.89	11.52	11.25	10.74	1.69
A_09_P019721	FBtr0306841	33810	CG11034	11.07	10.42	10.63	10.27	12.17	11.93	11.70	11.24	1.16
A_09_P019726	FBtr0079158	33814	CG8965	8.58	8.35	8.07	7.97	9.74	9.91	9.37	9.18	1.31
A_09_P019806	FBtr0079222	33846	CG9117	8.11	8.23	8.19	8.12	7.62	7.90	7.82	7.80	-0.38
A_09_P019816	FBtr0079186	33850	CG9135	8.95	9.54	9.56	9.59	8.63	8.81	8.68	8.75	-0.69
A_09_P019836	FBtr0079215	33854	Fbw5	11.56	11.31	11.43	11.28	10.87	10.81	11.08	11.25	-0.39
A_09_P019851	FBtr0079191	33857	CG13993	12.43	12.14	12.35	12.35	11.80	12.04	11.97	12.12	-0.33
A_09_P019876	FBtr0333407	33865	WDR79	9.40	9.28	9.39	9.50	9.97	10.00	9.91	9.84	0.53
A_09_P019941	FBtr0079280	33888	CG9500	5.36	4.50	5.01	5.02	6.52	6.47	5.88	5.88	1.21
A_09_P019956	FBtr0079255	33892	CG9507	6.63	7.58	6.97	7.40	5.48	6.08	5.67	5.56	-1.45

A_09_P019961	FBtr0299969	33893	CG42370	11.19	11.80	11.29	11.65	9.47	9.55	10.29	10.54	**-1.52**
A_09_P019966	FBtr0299968	33894	CG42369	11.92	11.64	11.71	11.63	13.27	13.48	13.13	13.00	**1.49**
A_09_P019976	FBtr0079257	33897	Fic	10.34	10.56	10.57	10.46	9.56	9.45	9.76	9.95	**-0.80**
A_09_P019981	FBtr0089948	33898	CG9527	9.07	8.95	8.91	8.99	8.86	8.51	8.39	8.23	**-0.48**
A_09_P019991	FBtr0079259	33900	frj	11.57	11.78	11.82	11.67	11.40	11.20	11.33	11.26	**-0.41**
A_09_P020001	FBtr0079270	33902	CG9531	11.02	10.72	10.95	10.75	10.52	10.38	10.28	10.22	**-0.51**
A_09_P020006	FBtr0079267	33904	CG9536	11.75	11.79	11.97	11.98	11.35	11.47	11.49	11.50	**-0.42**
A_09_P020016	FBtr0079265	33907	KFase	10.39	11.04	10.76	11.21	9.97	10.17	9.56	9.09	**-1.15**
A_09_P020021	FBtr0079264	33909	CG9548	12.58	12.56	12.38	12.46	11.80	11.67	12.03	12.38	**-0.52**
A_09_P020061	FBtr0089641	33923	CG11319	8.36	7.83	7.97	7.85	8.87	8.69	8.75	8.76	**0.77**
A_09_P020066	FBtr0079291	33924	CG11050	11.75	12.33	12.44	12.55	11.67	11.60	11.55	11.35	**-0.73**
A_09_P020076	FBtr0079295	33930	Tango1	13.12	13.24	13.27	13.42	12.62	12.41	12.91	13.04	**-0.52**
A_09_P020086	FBtr0079322	33935	Galt	13.53	13.69	13.63	13.68	13.07	13.26	13.23	13.34	**-0.41**
A_09_P020106	FBtr0079314	33941	Tsp	10.91	10.50	10.67	10.49	11.31	11.29	11.40	11.49	**0.73**
A_09_P020171	FBtr0079345	33964	Rat1	8.96	8.58	8.83	8.64	7.84	8.25	8.40	8.45	**-0.52**
A_09_P020176	FBtr0079367	33969	CG18304	8.69	9.06	8.82	8.70	7.62	7.50	7.67	8.12	**-1.09**
A_09_P020201	FBtr0079350	33976	CG3430	8.48	8.37	8.23	8.30	6.94	7.15	7.52	7.81	**-0.99**
A_09_P020206	FBtr0079362	33977	Atac1	9.34	9.41	9.33	9.44	9.03	9.24	9.01	9.09	**-0.28**
A_09_P020211	FBtr0079351	33978	CG10399	11.20	11.58	11.38	11.55	10.46	10.44	10.64	10.96	**-0.80**
A_09_P020236	FBtr0079428	33988	Rab30	8.17	8.07	8.05	8.17	8.63	8.46	8.45	8.35	**0.36**
A_09_P020246	FBtr0079402	33991	Mnn1	6.03	5.62	5.58	5.68	7.53	7.84	7.31	6.91	**1.67**
A_09_P020251	FBtr0110978	33991	Mnn1	8.34	8.26	8.05	8.00	8.74	8.99	8.71	8.69	**0.62**
A_09_P020271	FBtr0113028	33999	CG4495	11.81	12.14	11.96	12.02	10.82	10.68	11.06	11.53	**-0.96**
A_09_P020346	FBtr0079441	34017	CG5177	15.53	15.71	15.52	15.65	14.83	14.75	15.16	15.34	**-0.59**
A_09_P020351	FBtr0079467	34018	CG5181	9.28	9.55	9.49	9.45	9.25	9.05	9.15	9.19	**-0.28**
A_09_P020361	FBtr0079444	34021	CG5261	13.98	13.92	13.96	13.97	13.16	13.45	13.42	13.81	**-0.50**
A_09_P020366	FBtr0079466	34022	CG5958	15.61	15.31	15.40	15.27	14.32	14.28	14.65	14.86	**-0.86**
A_09_P020381	FBtr0330726	34030	CG42533	8.28	8.09	8.30	8.15	8.90	8.85	8.90	8.95	**0.70**
A_09_P020416	FBtr0079516	34041	CG18585	12.47	11.06	11.18	10.77	12.79	13.01	12.68	12.63	**1.41**
A_09_P020431	FBtr0302518	34047	CG13793	6.88	7.28	6.66	7.10	6.25	5.85	5.97	5.74	**-1.03**
A_09_P020441	FBtr0300709	34049	CG13795	8.70	9.46	9.13	9.62	6.92	7.46	7.40	7.88	**-1.81**

A_09_P020476	FBtr0079485	34058	CG7191	7.45	6.82	7.19	6.33	9.41	8.60	8.58	8.27	1.77
A_09_P020496	FBtr0079493	34065	Herp	12.42	12.66	12.60	12.58	12.02	11.87	12.13	12.24	-0.50
A_09_P020501	FBtr0079489	34066	r2d2	10.97	10.90	10.99	11.00	10.16	9.98	10.41	10.56	-0.68
A_09_P020551	FBtr0114550	34087	pes	8.94	8.65	8.88	8.44	10.47	10.34	9.83	9.41	1.28
A_09_P020561	FBtr0343382	34089	CG7224	12.06	12.32	12.32	12.38	14.13	14.65	13.86	13.28	1.71
A_09_P020596	FBtr0079540	34097	CG7429	9.43	9.76	9.60	9.95	8.77	8.77	8.83	8.88	-0.87
A_09_P020606	FBtr0079541	34099	CG7466	8.63	8.42	8.25	8.25	7.34	7.74	7.88	7.99	-0.65
A_09_P020611	FBtr0301229	326157	mon2	10.59	10.61	10.56	10.64	10.22	10.26	10.47	10.45	-0.25
A_09_P020621	FBtr0079642	34106	CG12375	12.24	12.25	12.15	12.26	11.83	11.60	11.80	12.04	-0.41
A_09_P020626	FBtr0079576	34107	CG8668	11.55	11.41	11.43	11.41	12.06	11.85	11.77	11.62	0.37
A_09_P020631	FBtr0079641	34109	CG8552	12.52	12.49	12.61	12.58	12.40	12.35	12.42	12.47	-0.14
A_09_P020651	FBtr0079639	34114	CG8475	11.95	12.51	12.24	12.51	11.21	11.04	11.47	11.78	-0.93
A_09_P020656	FBtr0079586	34115	CG8460	11.03	11.42	11.21	11.40	10.23	10.03	10.45	10.57	-0.94
A_09_P020661	FBtr0079587	34116	CG8455	9.05	9.18	9.15	9.21	8.90	8.59	8.78	8.84	-0.37
A_09_P020676	FBtr0079634	34121	CG8372	12.22	12.46	12.35	12.50	11.86	11.96	12.13	12.20	-0.34
A_09_P020736	FBtr0079597	34135	Scgalpha	8.98	8.55	8.98	8.62	7.92	7.42	7.95	8.05	-0.95
A_09_P020746	FBtr0079613	34137	Ostgamma	13.06	13.37	13.07	13.20	12.15	12.05	12.68	12.91	-0.73
A_09_P020771	FBtr0079601	34142	CG7787	11.02	11.10	11.11	11.05	10.41	10.31	10.81	10.92	-0.45
A_09_P020781	FBtr0079602	34144	CG14275	11.22	10.68	10.76	10.77	11.45	11.48	11.70	12.00	0.80
A_09_P020791	FBtr0079605	34146	CG14273	12.57	12.47	12.55	12.37	11.77	11.46	11.98	12.29	-0.62
A_09_P020836	FBtr0079719	34159	l(2)k12914	13.83	13.90	13.79	13.89	12.78	12.90	13.31	13.71	-0.68
A_09_P020871	FBtr0079681	34180	CG13088	7.37	7.32	7.65	7.62	7.09	6.66	6.90	7.18	-0.53
A_09_P020876	FBtr0079700	34181	Dh31	7.51	6.49	6.90	6.19	8.24	8.39	8.02	8.06	1.41
A_09_P020896	FBtr0079682	34185	CG13089	11.00	10.96	10.87	10.96	10.73	10.59	10.71	10.79	-0.25
A_09_P020926	FBtr0079750	34196	PrBP	10.56	10.92	10.79	11.04	9.54	9.60	9.64	9.81	-1.18
A_09_P020931	FBtr0079730	34197	Trs23	10.57	10.44	10.48	10.56	9.94	10.12	10.25	10.41	-0.33
A_09_P020946	FBtr0079744	34203	CG9463	11.61	10.28	10.89	10.93	12.61	12.53	12.29	11.95	1.42
A_09_P020951	FBtr0302992	34204	CG9465	8.70	7.11	7.48	6.96	9.40	9.11	9.01	8.81	1.52
A_09_P020971	FBtr0079754	34211	Tsp29Fa	13.22	13.28	13.13	13.26	14.02	13.63	13.72	13.59	0.52
A_09_P020976	FBtr0079756	34212	Tsp29Fb	14.24	14.14	14.23	14.15	15.01	14.76	14.73	14.42	0.54
A_09_P020986	FBtr0079788	34215	C1GalTA	10.15	10.14	10.14	10.20	10.84	10.66	10.54	10.41	0.46

A_09_P021026	FBtr0079765	34227	CG9568	12.85	12.16	12.35	12.36	14.15	13.80	13.72	13.50	1.36
A_09_P021031	FBtr0309842	34228	CG13102	7.05	6.13	5.50	6.38	9.63	10.01	9.18	8.55	3.08
A_09_P021066	FBtr0079774	34241	CG9586	12.32	12.47	12.48	12.59	11.93	11.95	12.08	12.19	-0.43
A_09_P021076	FBtr0079818	34245	borr	10.00	10.58	10.52	10.79	9.76	9.29	9.43	9.14	-1.07
A_09_P021151	FBtr0079861	34274	jp	12.30	12.49	12.37	12.50	11.83	11.46	11.78	11.86	-0.68
A_09_P021161	FBtr0079836	34278	CG3841	7.84	7.46	6.66	6.82	9.34	8.61	8.81	8.50	1.62
A_09_P021171	FBtr0079842	34282	GlcAT-S	9.24	9.08	9.06	9.06	9.58	9.79	9.45	9.42	0.45
A_09_P021181	FBtr0079848	34287	CG4364	14.31	14.27	14.15	14.32	15.04	15.30	14.93	14.94	0.79
A_09_P021216	FBtr0079869	34300	IP3K1	10.66	10.33	10.51	10.24	11.45	11.48	11.12	10.91	0.81
A_09_P021226	FBtr0079917	34303	CG13123	8.86	8.90	8.90	8.90	8.73	8.82	8.76	8.79	-0.12
A_09_P021231	FBtr0079912	34304	nAcRalpha-30D	9.23	9.23	9.11	9.17	10.17	10.60	10.14	10.12	1.07
A_09_P021241	FBtr0113031	34306	CG4537	7.79	7.98	7.87	7.96	7.08	7.52	7.60	7.55	-0.46
A_09_P021261	FBtr0079877	34315	CG4598	13.06	13.17	13.14	13.27	12.59	12.45	12.85	13.05	-0.43
A_09_P021266	FBtr0079878	34316	CG4594	8.43	8.78	8.67	8.71	7.33	7.33	7.65	7.86	-1.10
A_09_P021271	FBtr0079879	34317	CG4592	12.94	13.41	13.40	13.51	12.50	12.82	12.72	12.84	-0.60
A_09_P021311	FBtr0079890	34327	CG5850	9.62	9.77	9.59	9.75	8.83	8.74	9.17	9.44	-0.64
A_09_P021326	FBtr0079938	34339	Spn31A	9.46	9.04	9.32	8.99	8.52	8.16	8.57	8.70	-0.72
A_09_P021401	FBtr0079954	34362	CG5694	10.03	10.12	9.91	10.11	9.78	9.68	9.82	9.85	-0.26
A_09_P021411	FBtr0080086	34368	CG5676	8.08	8.58	8.09	8.35	6.78	6.82	7.40	7.84	-1.07
A_09_P021416	FBtr0089735	34371	CG18619	10.52	10.97	10.63	10.98	12.50	12.35	12.00	11.78	1.38
A_09_P021421	FBtr0079983	34372	Mob3	10.10	10.24	10.15	10.29	9.81	9.72	9.84	9.93	-0.37
A_09_P021456	FBtr0080070	34380	CYLD	9.14	9.70	9.43	9.48	8.76	8.43	8.74	8.77	-0.76
A_09_P021471	FBtr0079987	34384	CG4968	12.28	12.46	12.40	12.45	11.80	11.72	11.79	11.83	-0.61
A_09_P021476	FBtr0080062	34386	CG5385	10.55	10.88	10.65	10.72	9.89	9.47	9.99	10.19	-0.82
A_09_P021481	FBtr0080061	34387	CG5384	12.81	12.97	12.76	12.84	11.98	11.97	12.41	12.70	-0.58
A_09_P021486	FBtr0079988	34388	CG4972	11.60	11.29	11.33	11.14	10.54	10.23	10.70	11.11	-0.69
A_09_P021496	FBtr0079989	34390	CG4995	11.03	10.73	11.01	10.99	9.54	9.75	10.16	10.46	-0.96
A_09_P021516	FBtr0079998	34397	Sps2	9.99	9.80	9.98	9.92	10.45	10.20	10.29	10.19	0.36
A_09_P021521	FBtr0079999	34398	CG5022	7.98	8.18	8.09	8.18	8.49	8.73	8.47	8.45	0.43
A_09_P021541	FBtr0080003	34406	CG5056	9.50	9.52	9.55	9.59	9.41	9.39	9.35	9.38	-0.16
A_09_P021556	FBtr0080006	34410	CG5096	12.98	12.72	12.55	12.56	13.87	13.28	13.51	13.38	0.81

A_09_P021576	FBtr0080047	34421	CG5355	12.59	12.85	12.64	12.80	11.90	11.60	12.07	12.27	-0.76
A_09_P021586	FBtr0080019	34423	RfC3	10.16	9.76	9.94	9.91	8.90	8.83	9.36	9.59	-0.77
A_09_P021601	FBtr0080044	34429	CG5343	11.14	11.27	11.14	11.38	11.00	11.03	10.95	11.00	-0.24
A_09_P021611	FBtr0080026	34432	holn1	9.92	10.19	10.10	10.34	9.78	9.40	9.77	9.86	-0.43
A_09_P021626	FBtr0080042	34436	CG5322	11.44	10.46	10.51	10.41	12.31	12.01	12.01	11.77	1.32
A_09_P021641	FBtr0100565	34443	CG6144	9.24	9.43	9.28	9.36	8.76	8.72	8.81	9.08	-0.49
A_09_P021711	FBtr0080130	34468	CG7299	16.62	16.64	16.57	16.66	16.73	16.72	16.72	16.71	0.10
A_09_P021721	FBtr0080128	34470	CG7294	13.70	13.50	13.80	14.07	15.96	16.05	15.71	14.61	1.82
A_09_P021726	FBtr0080127	34471	CG17108	13.83	15.33	15.20	15.41	10.27	12.03	13.24	13.86	-2.59
A_09_P021736	FBtr0080108	34474	CG6415	12.46	13.08	12.82	13.08	10.61	11.02	11.47	11.90	-1.61
A_09_P021761	FBtr0080112	34480	Dpy-30L1	8.86	8.59	8.77	8.91	7.81	8.12	8.34	8.35	-0.63
A_09_P021786	FBtr0080118	34485	CG17124	9.34	9.49	9.30	9.41	9.86	9.99	9.86	9.76	0.48
A_09_P021816	FBtr0080187	34496	CG6700	10.95	11.28	11.14	11.16	11.52	11.45	11.38	11.48	0.33
A_09_P021871	FBtr0080147	34517	CG16743	11.59	11.15	11.27	11.38	12.57	12.41	12.38	12.13	1.03
A_09_P021921	FBtr0080232	34541	Ge-1	10.03	10.19	10.24	10.16	9.69	9.82	9.82	10.00	-0.32
A_09_P021926	FBtr0080230	34542	Reps	9.30	9.64	9.32	9.65	8.85	8.99	9.10	9.08	-0.47
A_09_P021951	FBtr0080199	34549	Nup160	10.57	10.52	10.53	10.52	10.26	10.24	10.39	10.45	-0.20
A_09_P021956	FBtr0080200	34551	CG4751	9.66	10.07	9.78	10.02	9.30	9.36	9.46	9.65	-0.44
A_09_P021971	FBtr0080202	34559	CG4788	7.91	8.06	7.94	7.81	6.69	6.94	7.33	7.46	-0.83
A_09_P022076	FBtr0080259	34593	CycY	10.91	11.18	11.12	11.15	10.78	10.84	10.59	10.69	-0.37
A_09_P022081	FBtr0334140	34594	Pde1c	6.77	6.69	6.47	6.24	7.72	8.14	7.47	7.63	1.20
A_09_P022116	FBtr0089572	34608	CG6686	11.45	11.61	11.58	11.69	11.77	11.81	11.78	11.74	0.20
A_09_P022126	FBtr0080285	34610	dgt2	10.07	10.50	10.37	10.51	10.78	10.96	10.79	10.84	0.48
A_09_P022131	FBtr0339524	318990	Qtzl	10.03	10.24	10.17	10.23	10.63	10.66	10.78	11.22	0.66
A_09_P022146	FBtr0080312	34616	CG6734	8.43	8.61	8.55	8.41	8.11	8.24	8.17	8.32	-0.29
A_09_P022181	FBtr0080296	34625	RpL7-like	12.23	11.68	12.00	11.91	12.80	12.98	12.78	12.51	0.81
A_09_P022221	FBtr0302854	34638	Phae2	11.50	12.14	11.85	12.11	13.92	13.18	13.53	13.26	1.57
A_09_P022271	FBtr0080388	34655	CG5446	10.94	11.07	11.06	11.29	10.81	10.47	10.70	10.71	-0.42
A_09_P022301	FBtr0080382	34663	CG5418	8.05	7.34	7.88	7.46	4.99	5.03	6.31	7.21	-1.80
A_09_P022321	FBtr0080411	34674	CG5525	13.91	13.87	13.91	13.79	13.47	13.40	13.55	13.69	-0.34
A_09_P022326	FBtr0080394	34675	CG6153	10.48	10.58	10.57	10.52	9.92	9.96	10.04	10.22	-0.50

A_09_P022366	FBtr0080398	34683	CG6180	14.62	14.67	14.67	14.68	14.01	13.93	14.26	14.51	**-0.48**
A_09_P022371	FBtr0080405	34684	CG5787	9.99	9.98	9.97	10.13	9.43	9.22	9.71	9.86	**-0.46**
A_09_P022376	FBtr0089908	34685	CG5792	10.69	10.78	10.57	10.76	10.47	10.30	10.40	10.51	**-0.28**
A_09_P022381	FBtr0090027	34686	MRP	13.70	13.69	13.77	13.73	14.22	14.31	14.22	14.27	**0.53**
A_09_P022441	FBtr0080492	34709	Sfmbt	10.78	11.13	11.03	11.18	10.49	10.55	10.65	10.72	**-0.43**
A_09_P022461	FBtr0080489	34713	CG16974	11.98	12.25	12.15	12.13	11.32	11.43	11.67	11.78	**-0.58**
A_09_P022491	FBtr0080484	34719	CG9426	8.34	8.78	8.68	8.75	7.99	7.85	8.03	8.02	**-0.66**
A_09_P022506	FBtr0080445	34722	CG16812	10.03	9.96	10.13	9.91	9.63	9.54	9.72	9.83	**-0.33**
A_09_P022591	FBtr0080465	34745	CG6523	13.37	13.40	13.44	13.33	13.20	13.10	13.24	13.23	**-0.19**
A_09_P022596	FBtr0080472	34747	CG9306	15.28	15.09	15.33	15.20	14.67	14.74	14.92	15.01	**-0.39**
A_09_P022606	FBtr0080468	34749	CG6565	11.37	11.00	11.24	10.95	10.77	10.68	10.52	10.57	**-0.50**
A_09_P022611	FBtr0080470	34750	CG9302	11.09	11.16	11.03	11.12	8.89	9.08	10.28	10.81	**-1.34**
A_09_P022616	FBtr0100220	34751	loqs	10.98	11.18	11.18	11.26	10.73	10.73	10.78	10.97	**-0.35**
A_09_P022661	FBtr0080519	34762	CG9267	11.50	11.61	11.51	11.48	10.69	10.62	10.88	11.11	**-0.70**
A_09_P022696	FBtr0100327	34808	Ance-3	7.27	8.65	8.25	8.31	6.62	6.98	6.54	6.33	**-1.51**
A_09_P022701	FBtr0080558	34811	CG16885	15.26	15.46	15.56	15.61	14.78	15.13	14.76	14.29	**-0.73**
A_09_P022796	FBtr0080891	35000	CG17904	12.40	12.33	12.42	12.43	12.49	12.66	12.67	12.79	**0.26**
A_09_P022831	FBtr0080908	35009	CG17928	11.97	11.82	11.84	11.74	11.52	11.39	11.25	11.10	**-0.53**
A_09_P022861	FBtr0080947	326156	CG31743	10.69	10.88	10.74	10.62	10.17	9.86	10.16	10.27	**-0.62**
A_09_P022931	FBtr0080979	35044	CG15141	9.73	10.18	9.97	10.22	9.43	9.07	9.43	9.78	**-0.60**
A_09_P022946	FBtr0081004	35049	CG6639	9.20	9.20	8.13	9.11	11.49	11.61	11.42	11.28	**2.54**
A_09_P022956	FBtr0080982	35052	Sgt	12.29	12.30	12.35	12.36	11.75	11.66	11.92	12.09	**-0.47**
A_09_P022961	FBtr0080983	35055	CG5110	10.88	10.70	10.90	10.97	10.36	10.46	10.41	10.32	**-0.48**
A_09_P022981	FBtr0080994	35060	CG6412	13.42	13.16	13.43	13.22	13.07	12.98	13.01	13.08	**-0.27**
A_09_P023051	FBtr0081029	35077	rdo	8.94	8.73	8.34	8.40	9.20	9.05	9.32	9.42	**0.64**
A_09_P023056	FBtr0081045	35080	CG15153	8.02	8.37	8.51	8.52	7.58	7.81	7.51	7.57	**-0.74**
A_09_P023066	FBtr0081034	35082	CG15152	11.83	11.69	11.46	11.59	12.81	12.60	12.33	11.96	**0.78**
A_09_P023076	FBtr0081038	35086	CG17681	8.96	9.09	8.58	8.93	7.76	7.90	7.95	8.22	**-0.93**
A_09_P023081	FBtr0081048	35087	CG15155	11.45	12.63	12.30	12.86	7.40	7.74	9.38	10.05	**-3.67**
A_09_P023091	FBtr0081062	35089	CG7200	10.09	10.18	10.14	10.25	9.50	9.21	9.40	9.26	**-0.82**
A_09_P023141	FBtr0081068	35105	CG10178	8.33	9.16	8.42	8.85	5.28	5.85	6.74	7.48	**-2.35**

A_09_P023176	FBtr0081077	35115	Cyp310a1	10.28	10.97	10.59	10.91	7.43	7.76	8.46	8.89	-2.55
A_09_P023181	FBtr0081073	35116	MESR3	9.95	9.96	9.94	9.97	11.13	10.89	10.63	10.33	0.79
A_09_P023186	FBtr0273194	35122	CG10336	8.58	8.72	8.72	8.91	8.22	8.55	8.29	8.31	-0.39
A_09_P023196	FBtr0081096	35124	CG10338	10.16	10.51	10.46	10.38	9.64	9.73	9.77	9.89	-0.62
A_09_P023211	FBtr0081098	35127	CG10343	11.89	11.76	11.81	11.86	11.34	11.52	11.55	11.70	-0.30
A_09_P023221	FBtr0081099	35130	Grip71	6.38	7.04	6.67	6.64	5.88	5.63	5.85	6.12	-0.81
A_09_P023226	FBtr0081125	35131	Irk3	10.03	10.60	10.64	10.37	9.83	9.27	9.35	9.23	-0.99
A_09_P023241	FBtr0081104	35138	CG17323	10.80	9.69	9.84	9.70	11.65	12.04	11.66	11.48	1.70
A_09_P023246	FBtr0081121	35140	CG17597	14.31	14.38	14.24	14.39	13.90	13.88	13.80	14.00	-0.43
A_09_P023256	FBtr0081110	35144	CG17321	6.90	6.68	6.59	6.20	7.54	7.75	7.49	7.50	0.98
A_09_P023266	FBtr0308069	35146	CG10602	14.23	14.15	14.10	14.12	13.35	13.23	13.55	13.71	-0.69
A_09_P023281	FBtr0081192	35151	Nedd8	13.14	13.15	13.18	13.27	12.24	12.27	12.62	12.88	-0.68
A_09_P023301	FBtr0081186	35156	CG10639	14.77	14.81	14.87	14.83	14.19	14.10	14.49	14.69	-0.45
A_09_P023346	FBtr0081146	35174	CG10470	13.06	12.91	12.86	12.90	12.35	12.02	12.51	12.79	-0.51
A_09_P023366	FBtr0081151	35180	CG17343	7.67	7.74	7.91	7.87	6.89	6.74	7.01	7.27	-0.82
A_09_P023371	FBtr0081174	35181	CG10702	8.95	9.00	8.97	9.07	9.87	9.98	9.61	9.41	0.72
A_09_P023421	FBtr0081222	35211	fon	16.05	16.13	16.03	16.04	15.81	15.71	15.90	15.97	-0.22
A_09_P023426	FBtr0081220	35212	CG17549	14.39	15.01	14.70	15.07	15.41	15.48	15.34	15.15	0.55
A_09_P023441	FBtr0081203	35219	CG13085	7.99	8.06	8.05	7.91	7.75	7.70	7.67	7.41	-0.37
A_09_P023461	FBtr0081236	35226	CG10026	10.56	11.37	11.04	11.23	8.78	8.55	9.51	9.94	-1.85
A_09_P023481	FBtr0081285	35231	CG10194	10.61	10.52	10.62	10.59	10.06	10.17	10.31	10.31	-0.37
A_09_P023491	FBtr0081284	35234	CG10189	9.81	9.78	9.67	9.71	10.42	10.67	10.58	10.67	0.84
A_09_P023506	FBtr0081247	35239	CG10132	11.53	11.49	11.72	11.62	10.27	10.31	10.43	10.85	-1.13
A_09_P023521	FBtr0081278	35242	CG10165	12.91	12.39	12.57	12.35	13.15	13.25	12.96	12.92	0.51
A_09_P023546	FBtr0081256	35251	CG13078	3.45	4.85	3.89	5.32	9.04	8.17	8.22	7.60	3.88
A_09_P023551	FBtr0081257	35252	CG13077	9.23	8.58	8.81	8.72	11.18	10.71	10.54	10.00	1.77
A_09_P023581	FBtr0081289	35261	Nf-YB	9.33	9.51	9.43	9.42	9.08	9.10	9.08	9.19	-0.31
A_09_P023606	FBtr0081318	35267	CdGAPr	7.66	7.94	7.60	7.59	8.06	8.10	8.08	8.21	0.41
A_09_P023611	FBtr0081291	35268	CG10466	8.96	8.91	8.79	8.96	8.34	8.49	8.56	8.78	-0.36
A_09_P023671	FBtr0343848	35291	CG10730	6.57	7.35	7.07	7.05	5.27	5.58	5.92	6.31	-1.24
A_09_P023691	FBtr0081335	35298	nesd	8.26	9.00	8.74	8.98	7.79	7.72	7.68	7.86	-0.98

A_09_P023731	FBtr0081358	35311	Arpc2	11.78	11.76	11.65	11.79	12.33	12.14	12.09	12.00	0.39
A_09_P023746	FBtr0081363	35316	CG2493	10.21	10.56	10.42	10.48	9.00	9.06	9.56	9.82	-1.06
A_09_P023771	FBtr0081384	35323	CG2608	11.08	11.03	11.03	10.98	10.72	10.55	10.75	10.90	-0.30
A_09_P023776	FBtr0081387	35324	CG2611	10.21	10.73	10.50	10.88	9.78	9.92	9.87	9.88	-0.72
A_09_P023791	FBtr0081399	35330	Cen	9.66	9.68	9.88	9.83	9.35	9.39	9.45	9.49	-0.34
A_09_P023796	FBtr0081391	35331	CG2617	7.78	8.18	7.99	8.11	7.33	7.19	7.41	7.50	-0.66
A_09_P023816	FBtr0081460	35337	CG9319	11.95	12.27	12.10	12.35	11.53	11.42	11.66	11.82	-0.56
A_09_P023821	FBtr0081409	35338	ns4	10.69	10.77	10.87	10.95	10.64	10.59	10.58	10.66	-0.20
A_09_P023866	FBtr0081426	35353	CG9335	9.67	9.63	9.80	9.57	10.29	10.24	10.05	10.01	0.48
A_09_P023876	FBtr0081427	35355	CG9336	11.81	12.29	12.14	12.38	13.50	13.82	13.22	12.63	1.14
A_09_P023881	FBtr0081428	35357	CG9338	12.55	12.68	12.81	12.93	12.17	12.40	12.42	12.46	-0.38
A_09_P023896	FBtr0081444	35359	sky	8.65	8.59	8.86	8.73	8.03	8.16	7.90	7.60	-0.79
A_09_P023901	FBtr0081439	35362	Mtp	13.58	13.43	13.55	13.35	11.32	11.33	12.71	13.17	-1.34
A_09_P023946	FBtr0081465	35375	CG9257	12.43	12.60	12.36	12.67	11.65	11.65	11.88	12.10	-0.69
A_09_P023961	FBtr0081500	35383	CG9249	9.42	9.19	9.22	9.16	8.91	8.77	8.86	9.05	-0.35
A_09_P023971	FBtr0081499	35385	Nbr	9.15	9.49	9.19	9.46	8.55	8.97	8.73	8.86	-0.55
A_09_P023981	FBtr0306974	35387	CG43345	12.84	12.65	12.82	12.78	12.27	12.39	12.40	12.44	-0.40
A_09_P023986	FBtr0081475	35390	Mcm10	8.47	8.24	8.34	8.13	6.74	6.81	7.33	7.63	-1.17
A_09_P024026	FBtr0081520	35408	nrv3	11.47	11.31	11.40	11.28	12.00	12.23	12.09	12.20	0.77
A_09_P024036	FBtr0081375	35410	CG17570	7.89	7.74	7.41	7.81	10.12	10.13	9.56	8.56	1.88
A_09_P024046	FBtr0085923	35417	CG2201	10.56	9.83	10.03	9.87	10.97	10.86	10.78	10.62	0.74
A_09_P024051	FBtr0085899	35420	Cul-2	10.81	10.80	10.80	10.83	10.41	10.34	10.55	10.69	-0.31
A_09_P024071	FBtr0085903	35426	CG1416	13.59	13.62	13.72	13.64	12.56	12.30	13.10	13.34	-0.82
A_09_P024176	FBtr0331294	35454	Tif-IA	9.83	9.56	9.90	9.36	9.40	8.86	9.07	8.98	-0.58
A_09_P024236	FBtr0086091	35492	CG10417	12.12	12.15	12.26	12.25	11.93	11.55	11.91	11.97	-0.36
A_09_P024271	FBtr0086061	35505	scaf	13.45	12.92	13.25	13.19	14.08	14.14	13.94	13.83	0.79
A_09_P024281	FBtr0086057	35511	CG7791	11.50	11.30	11.42	11.31	10.97	11.04	11.11	11.24	-0.29
A_09_P024286	FBtr0086044	35512	gp210	11.80	11.84	11.75	11.79	11.22	10.93	11.35	11.54	-0.54
A_09_P024316	FBtr0085966	35520	CG7882	11.54	11.46	11.50	11.42	10.19	10.68	10.87	11.11	-0.77
A_09_P024341	FBtr0086028	35528	dream	7.53	7.65	7.55	7.62	9.25	9.27	8.61	8.12	1.23
A_09_P024351	FBtr0086026	35531	CG14591	8.46	8.32	8.24	8.28	8.66	8.85	8.70	8.77	0.42

A_09_P024366	FBtr0085975	35535	CCHa2r	5.08	4.48	4.69	4.23	5.36	6.09	5.86	5.98	**1.20**
A_09_P024381	FBtr0086022	35538	CG14590	9.66	9.42	9.57	9.22	8.44	8.15	8.73	9.00	**-0.89**
A_09_P024386	FBtr0086020	35539	Ars2	11.83	12.00	12.00	11.94	11.18	11.06	11.55	11.82	**-0.54**
A_09_P024396	FBtr0085987	35543	Cyp6w1	10.84	11.62	11.04	11.52	10.56	10.57	10.47	10.40	**-0.76**
A_09_P024431	FBtr0085995	35554	Pld	10.16	10.03	10.11	10.06	11.09	10.68	10.54	10.45	**0.60**
A_09_P024446	FBtr0086147	35563	geminin	11.30	11.38	11.20	11.32	10.98	10.97	10.94	11.10	**-0.30**
A_09_P024466	FBtr0086145	35569	Hsepi	6.47	6.86	6.77	6.47	5.82	6.04	6.08	6.21	**-0.60**
A_09_P024491	FBtr0086123	35575	CG3270	13.05	13.79	13.60	13.85	12.40	12.65	12.68	12.83	**-0.93**
A_09_P024516	FBtr0086135	35585	CG3420	12.93	12.56	12.88	12.62	12.23	12.24	12.36	12.42	**-0.43**
A_09_P024541	FBtr0086207	35596	coro	14.47	14.71	14.62	14.70	14.24	13.95	14.25	14.37	**-0.42**
A_09_P024551	FBtr0300358	35599	Spn42Db	7.25	7.95	7.67	8.03	5.18	5.75	5.93	6.49	**-1.89**
A_09_P024581	FBtr0086179	35612	Tsp42Ec	12.11	12.02	12.21	12.08	13.48	12.82	12.95	12.63	**0.87**
A_09_P024591	FBtr0089024	35616	Tsp42Eg	13.38	13.43	13.43	13.48	13.15	13.03	12.78	12.59	**-0.54**
A_09_P024626	FBtr0089034	35624	Tsp42En	10.38	10.03	10.41	10.13	10.95	10.96	10.74	10.57	**0.57**
A_09_P024631	FBtr0089035	35625	Tsp42Eo	9.88	9.49	10.00	9.88	9.08	9.01	9.34	9.52	**-0.58**
A_09_P024676	FBtr0089049	35646	Gadd45	6.21	5.01	5.94	4.80	8.54	7.86	7.91	7.31	**2.42**
A_09_P024706	FBtr0088966	35654	CG11107	11.38	11.39	11.47	11.37	11.20	11.36	11.19	11.27	**-0.15**
A_09_P024796	FBtr0088904	35685	CG2144	11.28	11.25	11.17	11.23	10.61	10.40	10.66	10.88	**-0.59**
A_09_P024816	FBtr0088955	35690	CG1598	12.35	12.43	12.32	12.30	11.84	11.65	11.98	12.18	**-0.44**
A_09_P024826	FBtr0088910	35693	Vps13	11.55	11.93	11.73	11.81	11.45	11.36	11.28	11.39	**-0.39**
A_09_P024861	FBtr0088923	35708	CG2064	11.87	12.05	11.90	12.07	13.68	13.53	13.55	13.51	**1.60**
A_09_P024866	FBtr0088944	35709	CG12042	9.86	10.20	9.73	10.07	8.67	9.02	8.56	8.85	**-1.19**
A_09_P024881	FBtr0088924	35712	CG12107	12.53	12.50	12.51	12.44	11.91	11.67	12.10	12.38	**-0.48**
A_09_P024886	FBtr0088941	35713	U2A	11.36	11.39	11.28	11.38	10.13	10.46	10.60	11.19	**-0.76**
A_09_P024891	FBtr0088939	35715	CG1399	10.36	10.21	10.46	10.24	11.37	11.48	11.18	10.78	**0.88**
A_09_P024901	FBtr0088928	35719	CG1942	10.10	10.41	10.08	10.07	9.82	9.41	9.67	9.70	**-0.51**
A_09_P024911	FBtr0088829	35726	CG12825	13.39	13.30	13.20	13.34	14.12	13.96	13.91	13.78	**0.63**
A_09_P024921	FBtr0088901	35730	Nop17l	12.24	12.52	12.43	12.44	11.96	11.90	12.03	12.04	**-0.43**
A_09_P024931	FBtr0088834	35733	CG1882	12.60	12.41	12.57	12.58	14.20	14.40	13.99	13.67	**1.52**
A_09_P024946	FBtr0088886	35744	Kdm4A	8.55	8.77	8.62	8.50	8.21	8.07	8.00	8.31	**-0.46**
A_09_P024956	NM_136489	35748	CG8728	13.34	13.16	13.27	13.17	12.53	12.52	12.91	13.03	**-0.49**

A_09_P024961	FBtr0088880	35749	CG14764	9.46	9.66	9.65	9.62	9.31	9.23	9.19	9.12	-0.38
A_09_P024976	FBtr0088878	35754	CG2915	14.36	14.32	14.33	14.38	13.78	13.79	13.88	13.98	-0.49
A_09_P024991	FBtr0088871	35761	ACC	13.71	13.73	13.70	13.58	12.35	12.78	13.04	13.32	-0.80
A_09_P025036	FBtr0088821	35780	Cul-4	10.64	10.50	10.72	10.54	10.42	10.26	10.38	10.34	-0.25
A_09_P025041	FBtr0088782	35781	CG18316	13.58	13.95	13.75	14.05	11.79	11.76	11.85	11.83	-2.02
A_09_P025061	FBtr0300658	35787	Pbp49	10.25	10.40	10.42	10.57	9.85	10.01	9.95	10.17	-0.41
A_09_P025071	FBtr0088815	35790	Lpin	8.87	8.90	8.90	9.06	10.23	10.11	9.64	9.30	0.88
A_09_P025081	FBtr0088807	35793	RagC	11.84	12.02	11.94	12.02	11.74	11.70	11.59	11.65	-0.28
A_09_P025091	FBtr0088805	35797	CG14757	6.38	6.33	7.00	6.54	5.66	5.68	5.82	5.44	-0.91
A_09_P025161	FBtr0088748	35827	Mal-A4	12.55	12.06	11.96	12.25	13.67	12.80	13.28	13.05	1.00
A_09_P025211	FBtr0088730	35842	CG8735	12.09	12.53	12.40	12.68	11.76	11.56	11.65	11.65	-0.77
A_09_P025236	FBtr0088695	35849	CG14749	10.79	10.84	10.85	10.81	10.50	10.48	10.54	10.65	-0.28
A_09_P025301	FBtr0088684	35880	MrgBP	12.79	12.80	12.83	12.92	12.56	12.74	12.62	12.61	-0.20
A_09_P025306	FBtr0088683	35882	CG8258	13.52	13.30	13.63	13.26	12.85	12.86	13.25	12.99	-0.44
A_09_P025321	FBtr0290033	35889	CG8247	7.82	8.01	7.85	7.92	6.77	6.80	7.21	7.47	-0.84
A_09_P025331	FBtr0088653	35891	CG8237	12.09	12.10	12.09	12.14	11.45	11.24	11.69	11.87	-0.54
A_09_P025336	FBtr0088676	35892	CG8235	13.01	13.11	13.10	13.11	12.49	12.65	12.75	12.86	-0.39
A_09_P025376	FBtr0088671	35901	CG8216	7.53	7.54	7.61	7.38	7.89	8.21	7.90	7.88	0.46
A_09_P025406	FBtr0088664	35909	Ance-4	12.87	12.66	12.81	12.66	13.24	13.17	13.26	13.21	0.47
A_09_P025411	FBtr0088663	35910	proPO45	12.72	13.12	13.41	13.58	11.81	11.65	12.00	12.02	-1.34
A_09_P025441	FBtr0086502	37244	TBCB	11.63	11.87	11.84	11.90	11.37	11.17	11.46	11.52	-0.43
A_09_P025451	FBtr0086500	37247	hpo	10.28	10.33	10.46	10.36	9.89	9.77	9.78	9.55	-0.61
A_09_P025526	FBtr0086327	37267	Obp56e	9.96	11.68	11.45	11.75	7.43	8.78	8.53	8.87	-2.80
A_09_P025566	FBtr0086325	37276	CG16898	7.97	8.21	7.99	8.40	10.20	10.23	9.68	9.37	1.73
A_09_P025581	FBtr0086313	37281	CG11044	9.85	10.19	10.25	10.22	8.55	8.59	8.88	9.10	-1.35
A_09_P025616	NM_166382	37289	CG11055	9.52	9.55	9.51	9.45	9.74	9.84	9.85	9.96	0.34
A_09_P025641	FBtr0086303	37297	CG9090	13.68	14.19	14.01	14.28	12.58	12.58	12.94	13.30	-1.19
A_09_P025671	FBtr0086297	37305	MED8	10.34	10.34	10.35	10.45	9.93	10.04	9.89	9.99	-0.41
A_09_P025711	FBtr0086214	37313	CG13422	6.81	3.98	4.57	4.38	13.66	12.96	12.64	11.37	7.72
A_09_P025716	FBtr0086216	37314	CG18067	7.32	8.65	7.49	8.28	11.83	11.91	11.33	10.50	3.46
A_09_P025721	FBtr0086217	37315	CG13423	8.33	8.34	8.27	8.26	7.85	7.38	7.85	8.01	-0.53

A_09_P025751	FBtr0086259	37323	Mgat1	10.19	10.48	10.32	10.79	9.16	9.32	9.74	10.11	**-0.86**
A_09_P025761	FBtr0086225	37329	CG9945	8.66	8.73	8.69	8.83	9.30	9.39	9.26	9.36	**0.60**
A_09_P025776	FBtr0086253	37332	Rcd6	9.14	8.79	8.61	8.67	9.98	10.30	9.92	9.87	**1.21**
A_09_P025786	JV226083	37337	CG11110	10.45	10.57	10.44	10.75	10.75	10.98	10.91	11.00	**0.36**
A_09_P025791	FBtr0086231	37337	CG11110	9.76	9.76	9.82	9.84	9.50	9.60	9.43	9.29	**-0.34**
A_09_P025821	FBtr0086245	37344	Fem-1	11.13	11.44	11.15	11.28	12.60	11.89	12.02	11.88	**0.84**
A_09_P025826	FBtr0086236	246485	CG30152	12.13	12.21	12.24	12.25	12.27	12.47	12.38	12.49	**0.19**
A_09_P025926	FBtr0071547	37382	CG3295	11.54	11.81	11.53	11.80	12.61	12.55	12.34	12.26	**0.77**
A_09_P025956	FBtr0071542	37392	Cht9	14.78	14.41	13.99	14.56	15.87	15.81	15.58	15.24	**1.19**
A_09_P025971	FBtr0071567	37399	CG9394	12.69	12.77	12.24	12.32	14.79	14.76	14.24	13.41	**1.79**
A_09_P025991	FBtr0113100	37411	CG4266	11.85	12.11	12.02	11.99	11.29	11.62	11.64	11.79	**-0.41**
A_09_P025996	FBtr0071575	37412	CG9437	10.08	10.20	10.18	10.31	9.54	9.62	9.78	10.04	**-0.45**
A_09_P026001	FBtr0071628	37413	LSm1	11.35	11.34	11.37	11.51	10.79	10.92	11.08	11.21	**-0.39**
A_09_P026026	FBtr0110814	37421	CG15661	10.72	9.52	10.32	9.36	11.22	11.25	11.31	11.30	**1.29**
A_09_P026046	FBtr0071616	37431	CG9752	11.34	11.40	11.24	11.37	10.11	10.11	10.63	11.08	**-0.86**
A_09_P026056	FBtr0071600	37435	CG9485	13.28	13.90	13.66	13.83	11.73	12.19	12.66	13.16	**-1.23**
A_09_P026071	FBtr0071611	37441	CG17974	10.63	11.48	11.14	11.43	3.63	6.51	9.57	10.42	**-3.64**
A_09_P026076	FBtr0071609	37443	CG10795	9.05	9.23	9.10	9.28	8.90	8.88	8.87	8.95	**-0.26**
A_09_P026086	FBtr0071606	37445	Acox57D-p	10.46	10.46	10.58	10.48	10.11	10.03	10.14	10.25	**-0.36**
A_09_P026091	FBtr0071607	37446	Acox57D-d	10.84	10.73	10.81	10.61	10.19	9.99	10.08	10.12	**-0.65**
A_09_P026106	FBtr0071692	37456	CG10440	7.51	8.47	7.46	7.98	10.29	10.63	10.33	10.38	**2.55**
A_09_P026116	FBtr0342900	37459	CG15673	7.90	7.06	7.15	7.11	8.98	9.07	8.59	8.20	**1.40**
A_09_P026156	FBtr0071682	37468	pirk	10.98	9.84	10.48	10.27	12.94	12.02	12.25	11.82	**1.86**
A_09_P026161	FBtr0299964	37469	CG42365	9.27	9.44	9.33	10.01	11.90	11.98	11.26	10.57	**1.91**
A_09_P026166	FBtr0071680	37470	CG9865	12.05	11.87	11.96	11.94	11.81	11.73	11.65	11.55	**-0.27**
A_09_P026186	FBtr0071671	37477	CG10307	7.04	7.71	7.48	7.87	6.01	6.24	6.72	6.94	**-1.05**
A_09_P026196	FBtr0071711	37482	LBR	6.72	6.81	6.81	6.67	5.11	4.78	5.49	6.08	**-1.39**
A_09_P026221	FBtr0302446	37487	CG13492	13.37	12.69	12.94	12.73	13.92	13.75	13.67	13.28	**0.72**
A_09_P026291	FBtr0071802	37514	CG11474	12.01	12.02	11.99	11.98	10.88	10.78	11.22	11.42	**-0.92**
A_09_P026296	FBtr0071800	37515	CG2921	11.37	11.47	11.52	11.66	10.86	10.70	11.01	11.23	**-0.56**
A_09_P026356	FBtr0071769	37534	CG11275	11.24	11.86	11.24	11.80	12.13	12.28	12.27	12.34	**0.72**

A_09_P026366	FBtr0071771	37536	Vps35	11.49	11.63	11.59	11.57	10.92	10.71	11.01	11.09	**-0.64**
A_09_P026376	FBtr0071783	37538	CG3292	12.41	13.55	12.86	13.42	8.89	9.32	11.08	11.90	**-2.76**
A_09_P026381	FBtr0071782	37539	CG3290	6.43	7.76	7.70	7.83	NA	4.44	4.09	5.37	**-2.79**
A_09_P026386	FBtr0071781	37540	CG3264	14.41	14.33	14.27	14.42	12.79	13.58	13.73	14.07	**-0.81**
A_09_P026401	FBtr0071777	37545	Oatp58Dc	12.68	13.15	13.21	13.32	11.71	11.95	12.13	12.52	**-1.01**
A_09_P026416	FBtr0071846	37552	Liprin-gamma	8.72	8.45	8.41	8.34	9.03	9.12	9.25	9.31	**0.69**
A_09_P026436	FBtr0071822	37556	CG13506	11.92	11.75	11.84	11.94	12.95	12.78	12.56	12.21	**0.76**
A_09_P026441	FBtr0071845	37557	babos	12.01	11.87	11.90	11.58	12.97	13.27	13.09	12.85	**1.20**
A_09_P026461	FBtr0301668	37564	rad50	10.44	10.98	10.74	11.05	12.01	12.02	11.93	12.00	**1.19**
A_09_P026471	FBtr0071830	37566	ppk12	6.36	6.27	6.19	5.81	4.62	5.48	4.04	4.54	**-1.49**
A_09_P026486	FBtr0071832	37569	CG4752	12.74	13.26	13.02	13.30	12.20	12.11	12.41	12.56	**-0.76**
A_09_P026501	FBtr0071835	37572	CG6018	9.37	9.22	9.11	9.13	10.72	10.29	10.25	10.09	**1.13**
A_09_P026536	FBtr0071855	37580	RpS16	15.47	15.53	15.54	15.54	15.70	15.70	15.73	15.74	**0.20**
A_09_P026551	FBtr0071938	37585	CG17807	10.30	10.09	10.19	10.10	10.79	10.83	10.58	10.33	**0.46**
A_09_P026561	FBtr0071935	37589	RpS24	16.00	16.04	16.03	16.06	16.08	16.16	16.21	16.23	**0.14**
A_09_P026576	FBtr0071928	37594	Cyp6d2	8.17	9.62	9.01	9.52	11.88	11.90	12.03	12.12	**2.90**
A_09_P026581	FBtr0309799	37596	CG13510	11.37	10.92	10.90	10.90	12.83	12.69	12.55	12.18	**1.54**
A_09_P026586	FBtr0071863	37597	CG13511	8.31	7.62	7.60	7.37	10.08	10.30	9.59	9.10	**2.04**
A_09_P026591	FBtr0301269	37598	CG42565	7.51	6.53	6.64	6.45	9.90	10.06	9.21	8.58	**2.66**
A_09_P026601	FBtr0071865	37599	CG4250	10.00	9.77	10.11	10.12	10.84	10.81	10.62	10.40	**0.67**
A_09_P026606	FBtr0071868	37601	RYBP	9.14	9.24	8.98	9.03	9.87	9.89	9.89	9.88	**0.79**
A_09_P026706	FBtr0071905	37638	Gmer	11.06	10.84	11.00	11.15	10.30	10.00	10.48	10.57	**-0.68**
A_09_P026731	FBtr0071994	37646	CG3800	15.33	15.27	15.41	15.33	14.93	15.25	15.14	14.98	**-0.26**
A_09_P026736	FBtr0071951	37647	CG9849	11.59	11.68	11.59	11.65	11.43	11.28	11.31	11.39	**-0.28**
A_09_P026741	FBtr0071993	37648	CG3831	8.91	9.11	9.02	8.88	7.97	7.76	8.24	8.53	**-0.85**
A_09_P026751	FBtr0071988	37652	CG9896	6.72	6.91	6.91	6.59	5.64	5.75	5.63	5.89	**-1.06**
A_09_P026766	FBtr0071984	37659	CG9890	11.24	11.12	11.23	11.19	11.69	11.86	11.56	11.50	**0.46**
A_09_P026901	FBtr0072036	37702	CG3493	10.63	10.92	10.76	10.83	10.51	10.53	10.57	10.62	**-0.23**
A_09_P026926	FBtr0113111	37710	CG9815	9.96	9.46	9.63	9.67	10.46	10.87	10.32	10.11	**0.76**
A_09_P026961	FBtr0072057	37720	CG13559	7.51	7.78	7.49	7.40	5.99	6.56	7.01	7.24	**-0.84**
A_09_P026971	FBtr0072058	37723	mi	9.47	9.54	9.49	9.35	8.98	9.00	9.08	9.16	**-0.41**

A_09_P026986	FBtr0072112	37730	pita	10.72	10.92	10.73	11.05	11.24	11.18	11.18	11.04	**0.30**
A_09_P027001	FBtr0072109	3771883	CG10332	7.37	9.43	7.99	8.66	15.18	14.56	14.76	14.62	**6.42**
A_09_P027016	FBtr0306160	37738	CG17662	8.80	8.20	8.63	7.89	7.10	6.51	6.83	6.77	**-1.58**
A_09_P027021	FBtr0072105	37739	CG4019	15.04	15.13	15.13	15.14	14.49	14.46	14.70	14.86	**-0.48**
A_09_P027056	FBtr0072097	37751	CG4091	10.67	10.82	10.54	10.76	11.22	11.36	11.24	11.25	**0.57**
A_09_P027071	FBtr0072088	37758	CG13560	12.63	13.28	13.92	13.67	6.98	9.43	10.99	11.58	**-3.63**
A_09_P027076	FBtr0072089	37759	CG12491	7.18	7.77	8.43	7.88	2.87	4.35	5.44	6.27	**-3.08**
A_09_P027086	FBtr0072091	37761	CG5532	12.29	12.32	12.32	12.41	11.93	11.86	12.07	12.08	**-0.35**
A_09_P027146	FBtr0072235	37776	eIF6	13.42	12.72	13.11	12.93	13.75	13.77	13.81	13.80	**0.74**
A_09_P027151	FBtr0072233	37780	wibg	10.49	10.49	10.50	10.41	10.08	9.83	9.87	10.22	**-0.47**
A_09_P027171	FBtr0072132	37791	DNA-ligI	9.91	9.86	9.87	9.95	9.34	9.07	9.41	9.70	**-0.51**
A_09_P027176	FBtr0072221	37792	Upf3	9.08	9.09	9.07	9.08	8.65	8.50	8.69	8.91	**-0.39**
A_09_P027186	FBtr0072220	37794	CG5339	10.54	10.61	10.71	10.66	9.91	10.07	10.14	10.18	**-0.55**
A_09_P027231	FBtr0072147	37810	thoc5	10.18	10.04	10.11	10.09	9.16	9.19	9.64	9.72	**-0.68**
A_09_P027251	FBtr0072205	37817	CG10904	9.86	10.03	10.02	9.98	9.80	9.73	9.73	9.62	**-0.26**
A_09_P027266	FBtr0072160	37824	Pask	5.72	5.52	5.15	5.13	6.04	6.14	6.24	6.52	**0.86**
A_09_P027301	FBtr0072166	37838	MAN1	11.13	11.32	11.28	11.38	10.76	10.83	10.90	11.08	**-0.38**
A_09_P027331	FBtr0072174	45329	RpL12	14.53	14.62	14.53	14.74	14.76	14.82	14.82	14.86	**0.21**
A_09_P027336	FBtr0290258	37851	yki	11.67	11.80	11.78	11.86	12.14	12.14	12.04	11.90	**0.28**
A_09_P027341	FBtr0072179	37852	CG3209	13.56	13.59	13.64	13.62	13.50	13.42	13.37	13.29	**-0.21**
A_09_P027371	FBtr0072252	37862	CG3257	8.04	8.18	7.88	8.00	8.53	8.96	8.64	8.75	**0.69**
A_09_P027391	FBtr0072277	37872	mRpS17	11.66	11.47	11.60	11.46	11.17	11.24	11.22	11.41	**-0.29**
A_09_P027396	FBtr0072260	37874	CG3363	9.42	9.32	9.39	9.44	9.22	9.04	9.16	9.08	**-0.27**
A_09_P027476	FBtr0072355	37901	CG4563	13.89	14.32	14.45	14.08	12.77	12.38	12.77	12.94	**-1.47**
A_09_P027556	FBtr0072307	37923	CG11414	11.97	12.05	11.96	12.09	12.42	12.35	12.27	12.22	**0.29**
A_09_P027561	FBtr0072308	37924	uri	8.67	8.35	8.64	8.58	7.93	7.87	8.17	8.17	**-0.53**
A_09_P027601	FBtr0072317	37934	CG3570	10.48	10.16	10.43	10.38	11.07	11.26	10.81	10.74	**0.61**
A_09_P027631	FBtr0072336	37940	CG13594	7.00	6.74	6.54	6.19	7.65	8.06	7.70	7.84	**1.19**
A_09_P027641	FBtr0072335	37943	CG4781	12.30	11.91	12.11	11.94	13.41	13.07	12.95	12.48	**0.91**
A_09_P027706	FBtr0112777	37962	egg	11.50	11.75	11.74	11.97	11.15	11.31	11.25	11.44	**-0.45**
A_09_P027761	FBtr0072412	37988	CG2811	9.58	9.93	9.55	9.84	10.98	10.88	10.45	10.16	**0.89**

A_09_P027791	FBtr0072395	37996	CG3776	13.00	12.99	13.06	13.00	12.65	12.35	12.66	12.75	-0.41
A_09_P027806	FBtr0072396	38000	CG3829	10.58	10.09	10.36	10.26	10.74	11.09	11.11	11.24	0.72
A_09_P027811	FBtr0072443	38003	Nplp1	9.23	8.89	9.23	8.89	9.35	9.48	9.43	9.39	0.35
A_09_P027846	FBtr0301889	38021	p130CAS	10.53	10.52	10.56	10.61	11.34	11.32	11.15	10.93	0.63
A_09_P027876	FBtr0072484	38031	CG13876	7.65	7.62	7.71	7.54	7.39	7.15	7.33	7.19	-0.36
A_09_P027881	FBtr0072527	38033	thoc7	11.35	11.33	11.41	11.40	10.69	10.72	11.00	11.11	-0.49
A_09_P027896	FBtr0072524	38037	pyx	8.07	8.38	8.06	8.21	8.74	8.83	8.53	8.42	0.45
A_09_P027901	NR_048204	10178964	CR42862	6.40	6.67	6.29	6.20	7.78	7.63	7.05	6.94	0.96
A_09_P027911	FBtr0332106	38044	wac	10.19	10.35	10.35	10.40	9.51	9.33	9.87	10.04	-0.64
A_09_P027916	FBtr0072519	38045	Tudor-SN	14.57	14.49	14.46	14.49	13.87	13.85	14.17	14.30	-0.45
A_09_P027946	FBtr0333962	38050	RhoGEF3	7.91	8.01	7.98	7.84	8.79	8.73	8.42	8.33	0.63
A_09_P027976	FBtr0072549	38063	CG1233	9.53	9.81	9.71	9.67	10.10	10.07	9.99	9.92	0.34
A_09_P027991	FBtr0072550	38068	CG17180	10.86	10.96	10.96	10.97	10.79	10.72	10.68	10.59	-0.24
A_09_P028051	FBtr0072611	38081	CG3386	10.12	10.34	10.29	10.31	9.82	9.91	9.96	10.12	-0.31
A_09_P028066	FBtr0072577	38084	RabX6	9.28	9.27	9.21	9.23	9.03	8.94	8.95	9.06	-0.25
A_09_P028121	FBtr0300712	38097	CG13902	8.25	8.31	8.30	8.38	NA	NA	3.09	4.72	-4.40
A_09_P028141	FBtr0072594	38101	dpr20	6.32	5.59	5.50	5.22	7.15	7.69	7.22	7.12	1.63
A_09_P028151	FBtr0072593	38104	CG13907	9.99	10.28	10.06	10.08	9.73	9.84	9.47	9.16	-0.55
A_09_P028156	FBtr0289973	38107	CG13905	12.31	12.54	12.28	12.26	10.60	11.77	11.47	11.51	-1.01
A_09_P028166	FBtr0072619	2768997	CG12038	8.10	8.10	8.10	8.23	8.52	8.52	8.34	8.32	0.29
A_09_P028176	FBtr0072625	38120	CG13912	10.75	10.68	10.79	10.83	9.59	9.82	10.18	10.45	-0.75
A_09_P028186	FBtr0072635	38124	CG9119	13.62	13.51	13.64	13.62	12.71	12.96	12.97	12.98	-0.69
A_09_P028196	FBtr0072651	38133	CG9194	10.32	10.34	10.36	10.41	9.88	9.68	9.69	9.77	-0.60
A_09_P028206	FBtr0072731	38136	Psf1	7.71	7.27	7.44	7.25	6.50	5.93	6.56	7.00	-0.91
A_09_P028211	FBtr0072652	38137	Sac1	12.60	12.70	12.59	12.62	13.15	13.20	13.16	13.26	0.57
A_09_P028231	FBtr0072663	38144	Rabex-5	9.14	9.07	9.08	9.15	9.96	9.71	9.65	9.43	0.58
A_09_P028246	FBtr0072668	38149	CG2469	9.18	9.92	9.29	9.99	10.50	10.77	10.52	10.56	0.99
A_09_P028251	FBtr0072727	38150	CG9186	11.47	11.81	11.29	11.60	10.81	10.68	10.64	10.80	-0.81
A_09_P028256	FBtr0072671	38151	CG9153	13.79	14.07	14.02	14.10	12.93	13.09	13.47	13.81	-0.67
A_09_P028261	FBtr0072725	38152	CG9184	4.90	3.75	3.53	3.17	6.84	6.16	7.24	7.63	3.13
A_09_P028271	FBtr0072724	38156	msd5	9.30	9.53	9.52	9.72	9.02	8.84	9.11	9.13	-0.49

A_09_P028276	FBtr0072723	38157	CG2211	6.73	7.23	6.87	7.15	6.42	6.38	6.23	6.38	-0.64
A_09_P028361	FBtr0072825	38187	CG12104	8.06	7.97	7.91	7.95	7.38	7.07	7.32	7.42	-0.68
A_09_P028366	FBtr0100190	3772196	CG18170	9.05	9.01	9.54	9.64	8.54	8.05	8.21	8.12	-1.08
A_09_P028376	FBtr0072823	38192	CG13926	12.06	12.10	12.26	12.28	11.12	11.29	11.61	11.72	-0.74
A_09_P028381	FBtr0072754	38193	ABCB7	6.45	6.13	6.16	5.65	8.40	8.03	7.76	7.38	1.79
A_09_P028406	FBtr0072810	38201	CG17249	10.57	10.54	10.54	10.65	10.31	10.35	10.38	10.44	-0.20
A_09_P028416	FBtr0072762	38205	CG7970	13.81	13.86	13.80	13.90	13.43	13.48	13.38	13.37	-0.43
A_09_P028476	FBtr0072800	38216	CG18171	5.70	6.27	6.32	6.16	3.80	4.67	3.69	2.69	-2.40
A_09_P028511	FBtr0072792	38227	CG13933	8.52	8.80	8.64	8.62	9.50	9.54	9.49	9.53	0.87
A_09_P028516	FBtr0072791	38229	CG2021	7.42	7.39	7.56	7.28	6.30	6.30	6.46	6.63	-0.99
A_09_P028531	FBtr0332874	2768999	CG42676	7.12	6.92	7.09	6.83	8.84	9.01	8.88	8.89	1.91
A_09_P028561	FBtr0072832	38243	CG12024	10.19	10.46	10.37	10.46	9.74	9.64	9.83	9.75	-0.63
A_09_P028576	FBtr0300717	38249	CG13937	11.61	11.30	11.57	11.45	11.18	11.03	11.03	11.02	-0.42
A_09_P028581	FBtr0072837	38251	Tmhs	11.19	10.92	10.86	10.88	11.35	11.72	11.38	11.45	0.51
A_09_P028601	FBtr0072858	38255	CG5687	11.73	11.11	11.39	11.23	10.39	10.69	10.73	10.90	-0.69
A_09_P028606	FBtr0072841	38256	Mfap1	11.21	11.43	11.34	11.41	10.94	10.99	11.07	11.20	-0.30
A_09_P028626	FBtr0072843	38261	CG1140	12.50	13.03	12.93	13.06	11.96	12.25	12.04	12.22	-0.76
A_09_P028646	FBtr0072916	38274	CG15879	9.11	9.13	9.30	9.16	8.55	8.58	8.69	8.69	-0.55
A_09_P028671	FBtr0332661	38282	osm-1	5.58	4.62	4.74	5.05	5.99	6.22	6.17	6.07	1.11
A_09_P028676	FBtr0072910	38283	CG9018	9.56	9.66	9.59	9.70	9.11	9.12	9.22	9.37	-0.42
A_09_P028686	FBtr0072878	38290	CG13807	12.51	12.62	12.66	12.77	11.84	11.98	12.17	12.34	-0.55
A_09_P028746	FBtr0072968	38310	CG16762	11.06	10.87	11.06	10.91	13.02	12.11	12.38	11.99	1.40
A_09_P028751	FBtr0072967	38311	Cyp4d20	9.18	8.78	8.99	8.83	10.21	10.29	10.12	10.11	1.24
A_09_P028771	FBtr0072929	38323	CG16985	11.26	11.17	11.28	11.26	10.48	10.42	10.53	10.74	-0.70
A_09_P028796	FBtr0072938	38330	CG1246	11.61	11.28	11.18	11.11	13.23	12.94	12.80	12.47	1.57
A_09_P028811	FBtr0333630	38337	CG43955	5.79	5.62	5.29	5.11	6.13	6.46	6.64	6.91	1.08
A_09_P028816	FBtr0072969	38341	CG1240	12.12	12.61	12.42	12.51	11.57	11.40	11.52	11.82	-0.84
A_09_P028836	FBtr0072973	38346	pgant6	11.23	11.34	11.49	11.77	10.64	10.79	10.90	10.91	-0.65
A_09_P028841	FBtr0333881	38347	CG43444	6.33	6.35	6.00	5.92	7.63	8.21	7.39	7.34	1.49
A_09_P028856	FBtr0073011	38350	spz5	6.72	4.74	5.99	3.97	8.71	8.96	8.90	9.04	3.55
A_09_P028876	FBtr0072978	38355	CG2107	10.30	10.52	10.32	10.39	9.71	9.32	9.68	9.88	-0.73

A_09_P028896	FBtr0073005	38362	scramb2	11.38	12.10	11.78	12.18	11.23	10.86	10.95	10.85	-0.89
A_09_P028901	FBtr0072989	38364	CG1271	10.79	10.41	10.61	10.39	11.59	11.41	11.24	11.10	0.78
A_09_P028926	FBtr0073116	38373	CG11537	11.14	11.59	11.33	10.99	12.58	12.64	11.83	11.94	0.99
A_09_P028941	FBtr0073114	38375	CG12082	12.22	12.59	12.29	12.34	11.26	11.12	11.48	11.70	-0.97
A_09_P028961	FBtr0073112	38381	CG14962	8.19	7.98	8.08	7.97	7.45	7.63	7.67	7.72	-0.44
A_09_P028971	FBtr0307018	38384	CG14964	7.01	7.57	6.97	6.50	4.02	NA	4.35	5.19	-2.50
A_09_P028981	FBtr0073038	38386	CG14957	11.17	11.32	11.35	11.47	11.73	12.06	11.87	11.96	0.57
A_09_P028991	FBtr0073109	38388	CG14965	6.90	7.23	6.78	7.10	6.24	6.42	6.42	6.53	-0.60
A_09_P028996	FBtr0073108	38390	CG14966	11.27	11.39	11.31	11.62	10.39	10.67	10.76	11.23	-0.63
A_09_P029011	FBtr0073043	38396	CG12034	11.88	12.14	12.06	12.06	11.81	11.84	11.73	11.82	-0.23
A_09_P029021	FBtr0073044	38398	CG17737	13.87	13.87	13.84	13.95	13.30	13.08	13.44	13.61	-0.53
A_09_P029041	FBtr0073050	38402	ckd	12.94	13.29	13.13	13.08	12.51	12.86	12.16	11.60	-0.83
A_09_P029061	FBtr0073089	38406	CG14968	11.30	10.97	11.08	10.89	12.24	12.40	12.08	11.81	1.08
A_09_P029071	FBtr0073062	38409	Drsl5	6.59	7.92	7.39	7.72	5.49	5.27	5.24	4.98	-2.16
A_09_P029081	FBtr0073063	38411	CG12016	10.87	10.63	10.92	10.68	11.06	11.15	11.10	11.02	0.31
A_09_P029091	FBtr0073066	38413	PHGPx	13.57	13.91	13.76	13.97	13.16	13.07	13.27	13.21	-0.63
A_09_P029106	FBtr0073075	38421	CG12010	9.05	9.03	9.01	8.92	9.48	9.64	9.35	9.40	0.47
A_09_P029116	FBtr0073077	38423	CG12014	5.67	5.64	5.29	5.32	6.40	7.10	6.65	6.67	1.23
A_09_P029121	FBtr0073143	38429	CG14971	11.41	11.76	11.73	11.91	11.10	10.76	11.09	11.23	-0.66
A_09_P029131	FBtr0334636	38430	CG42324	6.07	6.10	6.23	5.78	3.12	4.35	4.33	5.38	-1.75
A_09_P029161	FBtr0073185	38443	ntc	9.12	9.43	9.44	9.68	8.68	8.64	8.50	8.51	-0.83
A_09_P029166	FBtr0073148	38444	IntS10	7.74	7.86	7.88	7.82	7.21	7.40	7.42	7.70	-0.39
A_09_P029171	FBtr0073149	38446	CG12006	12.51	12.41	12.44	12.33	11.64	11.66	11.99	12.29	-0.53
A_09_P029181	FBtr0073158	38454	CG14977	8.99	8.88	8.78	8.92	8.52	8.46	8.64	8.39	-0.39
A_09_P029206	FBtr0073172	38462	CG12766	9.86	8.47	9.39	8.52	11.24	10.86	10.88	10.67	1.85
A_09_P029226	FBtr0333139	38467	CG14984	11.83	11.91	11.85	11.92	12.40	12.33	12.06	12.11	0.35
A_09_P029231	FBtr0073167	38468	CG12605	7.88	7.99	7.74	7.75	8.31	8.75	8.30	8.34	0.59
A_09_P029241	FBtr0073193	38472	Mul1	9.67	9.72	9.58	9.65	9.46	9.38	9.36	9.46	-0.24
A_09_P029246	FBtr0073284	38473	CG11594	12.10	12.39	12.17	12.29	11.07	11.40	11.63	11.97	-0.72
A_09_P029266	FBtr0073202	38478	Rcd5	10.49	10.80	10.53	10.90	11.31	11.38	11.19	11.13	0.57
A_09_P029271	FBtr0073203	38479	CG1136	10.70	9.62	9.83	9.68	11.94	11.67	11.35	10.83	1.49

A_09_P029281	FBtr0308363	38485	CG14989	10.75	10.78	10.81	10.73	11.62	11.99	11.59	11.58	0.93
A_09_P029311	FBtr0073215	38496	CG1299	12.28	12.21	12.34	12.27	13.68	13.96	13.34	12.72	1.15
A_09_P029331	FBtr0089173	43770	Ank	12.61	12.74	12.81	12.76	12.60	12.33	12.44	12.28	-0.31
A_09_P029341	FBtr0090002	32120	PhKgamma	9.85	10.65	10.26	10.49	8.26	8.44	8.52	9.29	-1.68
A_09_P029351	FBtr0070707	31405	ctp	14.75	14.80	14.85	14.81	14.59	14.63	14.62	14.66	-0.18
A_09_P029366	FBtr0087740	36461	Dp	10.96	11.01	10.97	11.03	10.83	10.67	10.71	10.74	-0.25
A_09_P029391	FBtr0071472	31960	Gip	13.69	13.82	13.76	13.81	12.90	12.87	13.06	13.21	-0.76
A_09_P029401	FBtr0082385	117419	Irbp	9.38	9.32	9.45	9.34	10.67	10.73	10.57	10.54	1.26
A_09_P029416	FBtr0076292	39195	Gem3	9.07	9.13	9.05	9.01	8.73	8.43	8.67	8.78	-0.41
A_09_P029436	FBtr0070100	30984	pcl	10.58	9.98	10.33	10.21	13.01	12.46	12.22	11.41	2.00
A_09_P029441	FBtr0079937	34338	Pen	10.94	12.03	11.77	12.15	10.64	10.16	10.44	10.41	-1.31
A_09_P029466	FBtr0081493	43875	Ret	5.85	6.28	5.83	6.00	5.20	4.77	5.31	5.25	-0.86
A_09_P029481	FBtr0076361	39164	Taf2	11.55	11.80	11.75	11.91	10.82	10.97	11.10	11.36	-0.69
A_09_P029491	FBtr0078413	40348	AcCoAS	15.04	15.01	14.87	14.87	13.57	13.95	14.08	14.34	-0.97
A_09_P029506	FBtr0087437	36636	AttA	11.81	12.98	11.62	12.59	16.63	15.87	16.28	16.04	3.96
A_09_P029521	FBtr0112869	37232	CalpA	12.40	12.24	12.34	12.28	13.01	13.05	12.71	12.59	0.52
A_09_P029531	FBtr0075522	39746	DNApol-delta	10.25	10.27	10.26	10.34	9.95	9.90	10.00	10.04	-0.31
A_09_P029541	FBtr0075703	2768981	Trl	8.05	7.47	7.98	7.65	7.01	6.77	7.28	7.09	-0.75
A_09_P029561	FBtr0083120	41860	FK506-bp1	13.47	13.16	13.34	13.27	13.71	13.72	13.56	13.58	0.33
A_09_P029606	FBtr0088863	35766	Odc1	12.40	11.83	12.12	11.93	12.49	12.93	13.14	13.37	0.91
A_09_P029611	FBtr0088864	35767	Odc2	11.77	11.70	11.79	11.96	11.33	11.36	11.38	11.41	-0.43
A_09_P029621	FBtr0086533	37235	RpL11	16.16	16.07	16.16	16.11	15.98	15.96	16.02	16.05	-0.12
A_09_P029626	FBtr0083204	41938	Sap47	11.19	11.02	11.04	10.96	11.72	11.81	11.60	11.77	0.67
A_09_P029646	FBtr0086102	35473	TpnC41C	10.39	10.22	10.77	10.94	8.94	8.76	9.57	9.40	-1.41
A_09_P029651	FBtr0080157	34529	dUTPase	13.50	13.84	13.71	13.89	13.05	13.12	13.21	13.32	-0.56
A_09_P029661	FBtr0080806	34947	beat-Ia	6.19	5.99	5.81	5.72	6.72	6.80	6.55	6.50	0.72
A_09_P029666	FBtr0088429	36051	cdc2rk	8.78	8.73	8.67	8.70	8.08	8.13	8.25	8.31	-0.53
A_09_P029676	FBtr0087361	36681	igl	10.37	9.79	10.10	9.86	10.82	10.69	10.78	10.81	0.74
A_09_P029697	FBtr0075777	45460	Pex1	8.73	8.72	8.70	8.47	8.27	8.29	8.20	8.40	-0.37
A_09_P029741	FBtr0100884	192472	CYTB	16.15	16.25	16.17	16.23	16.30	16.25	16.32	16.29	0.09
A_09_P029756	FBtr0100870	192475	ND3	13.94	13.71	13.63	13.61	13.35	13.00	13.32	13.45	-0.44

A_09_P029811	FBtr0086110	35564	sced	7.18	7.31	7.13	7.34	6.78	6.55	6.80	6.99	-0.46
A_09_P029821	FBtr0078854	40575	5-HT2	6.49	6.43	6.43	6.04	7.20	7.08	6.96	6.95	0.70
A_09_P029831	FBtr0079780	34225	alien	10.66	10.70	10.63	10.71	9.96	9.45	10.18	10.39	-0.68
A_09_P029836	FBtr0079781	34225	alien	6.52	6.93	6.48	6.59	4.73	5.31	5.84	6.21	-1.11
A_09_P029841	FBtr0089192	43823	Arf102F	13.21	13.05	13.10	13.19	12.27	12.21	12.71	12.97	-0.60
A_09_P029901	FBtr0087592	36546	Cp1	14.34	14.43	14.31	14.31	14.71	14.59	14.76	14.86	0.38
A_09_P029911	FBtr0087455	36666	Cyp6a8	10.83	11.03	10.88	11.10	8.04	7.92	8.50	8.77	-2.65
A_09_P029981	FBtr0086723	37123	Prp19	11.66	11.56	11.57	11.52	11.20	11.04	11.28	11.42	-0.34
A_09_P030011	FBtr0084121	42549	InR	8.77	8.48	8.16	7.89	11.45	11.66	10.39	10.51	2.68
A_09_P030041	FBtr0082883	41694	Nsf2	10.27	10.08	10.31	10.08	8.54	8.63	8.76	8.89	-1.49
A_09_P030046	FBtr0081306	44038	Hf	11.07	11.37	11.26	11.26	9.76	9.97	10.38	10.77	-1.02
A_09_P030066	FBtr0075913	39443	Ptp69D	9.71	9.68	9.74	9.75	9.31	9.16	9.22	9.23	-0.49
A_09_P030086	FBtr0089338	41087	Rel	12.28	12.04	12.15	12.50	15.16	14.95	14.55	13.75	2.36
A_09_P030126	FBtr0070916	31588	RpL7A	16.45	16.43	16.48	16.47	16.56	16.51	16.53	16.52	0.07
A_09_P030131	FBtr0075878	39480	RpS12	16.02	15.92	15.88	15.94	16.00	16.09	16.05	16.09	0.12
A_09_P030151	FBtr0071241	31781	Sptr	11.86	12.16	12.00	12.23	10.29	10.56	11.21	11.64	-1.14
A_09_P030166	FBtr0073323	38559	Tie	10.39	9.67	10.16	9.65	11.17	11.48	10.96	10.64	1.10
A_09_P030171	FBtr0075064	40055	Ugt	11.79	12.16	11.89	12.03	10.32	10.40	11.24	11.64	-1.07
A_09_P030181	FBtr0080834	34970	CG4278	10.48	10.44	10.42	10.42	10.07	9.75	10.09	10.22	-0.41
A_09_P030196	FBtr0083795	42356	bnl	6.78	5.60	5.89	5.08	8.00	7.96	7.53	7.01	1.79
A_09_P030221	FBtr0088014	36307	Oda	15.53	15.61	15.67	15.55	15.98	15.83	15.81	15.75	0.25
A_09_P030226	FBtr0079124	33781	Hel25E	12.71	12.54	12.58	12.61	12.03	11.80	12.25	12.43	-0.48
A_09_P030241	FBtr0089313	40667	mia	9.25	8.67	9.21	8.84	10.64	10.19	10.03	9.54	1.11
A_09_P030256	FBtr0111017	40436	mub	6.97	6.99	6.91	6.42	5.85	6.42	5.78	5.77	-0.87
A_09_P030266	FBtr0076214	64867	nol	15.83	15.46	16.04	15.59	14.33	15.28	14.68	14.24	-1.10
A_09_P030271	FBtr0082124	41136	RhoL	12.55	12.46	12.45	12.53	13.67	13.67	13.31	12.84	0.88
A_09_P030276	FBtr0073145	38424	sty	11.17	10.94	10.57	10.91	12.45	12.40	12.04	11.30	1.15
A_09_P030311	FBtr0079711	46386	CG13397	9.90	10.34	10.06	10.23	9.30	9.60	9.41	9.36	-0.72
A_09_P030316	FBtr0085390	46391	CG11899	12.38	11.68	11.82	11.57	13.77	13.83	13.51	13.39	1.76
A_09_P030356	FBtr0088745	35822	Cyp4e2	10.60	11.13	10.80	11.02	9.41	9.20	9.64	9.78	-1.38
A_09_P030396	FBtr0081827	40973	Mcm2	10.96	11.20	11.09	11.17	10.43	10.16	10.50	10.67	-0.66

A_09_P030401	FBtr0081616	40849	Mlp84B	15.00	15.35	15.42	15.32	14.21	14.31	14.27	14.61	**-0.92**
A_09_P030406	FBtr0087386	36708	Mtk	6.69	6.88	5.00	4.49	13.99	13.06	13.48	13.15	**7.66**
A_09_P030416	FBtr0085384	43447	Pglym78	16.03	16.04	16.10	16.14	14.92	14.93	15.65	15.85	**-0.74**
A_09_P030431	FBtr0083062	41844	Set	12.34	12.66	12.53	12.71	11.68	11.65	11.85	12.10	**-0.74**
A_09_P030441	FBtr0081763	40936	CG2846	12.06	12.00	12.11	12.10	11.55	11.67	11.69	11.76	**-0.40**
A_09_P030456	FBtr0303788	40793	gpp	8.52	8.70	8.58	8.55	8.92	9.13	8.75	8.91	**0.34**
A_09_P030466	FBtr0088125	36223	iotaTry	12.45	12.11	11.63	12.03	13.39	13.67	13.06	12.57	**1.11**
A_09_P030506	FBtr0079072	46040	cype	14.43	14.45	14.46	14.57	14.05	14.18	14.17	14.25	**-0.32**
A_09_P030526	FBtr0079925	34291	Cyp4e3	5.23	4.92	4.95	5.14	9.27	9.57	8.56	7.54	**3.67**
A_09_P030541	FBtr0089056	35634	Cyp9b1	9.63	10.17	10.16	10.24	9.56	9.34	9.45	9.67	**-0.54**
A_09_P030546	FBtr0089055	35635	Cyp9b2	12.91	13.76	13.29	13.49	12.22	11.88	12.07	12.07	**-1.31**
A_09_P030556	FBtr0078492	40457	Ddx1	11.51	11.65	11.47	11.67	10.90	10.51	10.95	11.10	**-0.71**
A_09_P030566	FBtr0076489	45525	eIF-4E	14.16	14.13	14.17	14.23	13.94	13.78	13.92	14.01	**-0.26**
A_09_P030596	FBtr0078392	40378	Hr78	11.74	12.10	12.01	12.09	11.13	11.48	11.43	11.67	**-0.56**
A_09_P030607	FBtr0073434	32045	Hsp60	11.63	11.56	11.47	11.30	10.61	10.51	10.75	11.19	**-0.73**
A_09_P030616	FBtr0072219	37798	Nap1	15.21	14.92	15.10	14.93	15.58	15.59	15.48	15.35	**0.46**
A_09_P030626	FBtr0082866	41703	Orc2	6.50	6.41	6.23	6.08	5.18	5.24	5.59	5.96	**-0.82**
A_09_P030631	FBtr0080543	34794	Orc5	7.46	7.67	7.44	7.41	6.14	6.74	6.60	7.03	**-0.87**
A_09_P030641	FBtr0072064	37733	Pi3K59F	10.46	10.77	10.59	10.59	11.33	11.52	11.24	11.25	**0.74**
A_09_P030661	FBtr0084520	42828	Pros26.4	11.93	12.11	11.80	11.93	11.06	10.86	11.40	11.70	**-0.69**
A_09_P030666	FBtr0078368	40388	Pros54	13.98	14.19	14.03	14.06	13.27	13.24	13.69	13.94	**-0.53**
A_09_P030701	FBtr0079590	34120	Ssb-c31a	11.67	11.47	11.50	11.72	11.09	10.92	11.31	11.39	**-0.41**
A_09_P030721	FBtr0076395	39133	UbcD4	12.15	12.24	12.18	12.34	12.00	11.66	11.88	12.01	**-0.34**
A_09_P030736	FBtr0078967	40530	abs	9.00	9.16	8.88	9.14	8.62	8.68	8.75	8.86	**-0.32**
A_09_P030756	FBtr0336486	42030	CG14906	10.05	9.87	9.95	9.90	11.79	12.16	11.68	11.43	**1.83**
A_09_P030761	FBtr0072877	38287	CG2034	12.11	12.06	12.20	12.14	11.80	11.98	11.86	11.96	**-0.23**
A_09_P030781	FBtr0072120	44172	RabX1	10.21	10.27	10.29	10.26	10.87	11.02	10.80	10.67	**0.58**
A_09_P030786	FBtr0074283	44054	crl	11.50	11.63	11.70	11.57	10.96	10.73	11.19	11.49	**-0.50**
A_09_P030791	FBtr0077541	44637	cutlet	9.03	9.07	9.25	9.20	8.51	8.56	8.61	8.49	**-0.60**
A_09_P030836	FBtr0078766	40660	ksr	7.91	8.40	8.36	8.44	7.76	7.43	7.86	7.92	**-0.54**
A_09_P030846	FBtr0088845	35742	lin19	12.24	12.19	12.14	12.05	11.96	12.05	12.00	11.94	**-0.17**

A_09_P030856	FBtr0289988	31767	nAcRalpha-7E	6.52	6.61	5.96	6.10	7.00	7.68	7.06	7.08	0.91
A_09_P030866	FBtr0335131	33487	RpS21	11.33	11.21	11.51	11.38	10.96	11.05	11.11	11.12	-0.29
A_09_P030876	FBtr0086799	37039	olf186-M	10.67	10.57	10.78	10.61	12.20	12.49	11.75	11.37	1.30
A_09_P030901	FBtr0072256	43958	spag	10.79	10.84	10.95	10.95	10.50	10.15	10.53	10.50	-0.46
A_09_P030916	FBtr0081131	35119	tos	8.24	8.21	8.21	8.16	7.45	7.33	7.65	7.84	-0.64
A_09_P030951	FBtr0081544	40909	alpha-Est1	7.83	8.01	8.16	7.82	8.80	8.85	8.64	8.52	0.75
A_09_P030956	FBtr0289953	40896	alpha-Est10	10.44	10.31	10.43	10.17	9.18	9.37	9.70	10.07	-0.76
A_09_P030961	FBtr0081540	40908	alpha-Est2	11.76	12.80	12.54	12.92	9.89	10.16	11.08	11.64	-1.81
A_09_P031001	FBtr0088176	36203	dare	11.91	11.93	11.90	11.85	11.63	11.67	11.72	11.84	-0.18
A_09_P031031	FBtr0085351	44642	Apc	10.30	10.45	10.47	10.40	9.62	9.43	9.25	9.24	-1.02
A_09_P031036	FBtr0084744	42947	Ast	9.86	9.82	9.66	9.60	10.92	11.13	10.41	10.29	0.95
A_09_P031071	FBtr0083052	41836	Caf1	12.15	12.24	12.20	12.33	11.52	11.27	11.69	11.76	-0.67
A_09_P031076	FBtr0088930	46456	CanB2	12.83	13.04	12.89	13.10	12.25	12.16	12.40	12.51	-0.64
A_09_P031091	FBtr0076369	39157	Cdk8	9.33	9.41	9.46	9.37	8.90	8.96	9.02	9.22	-0.36
A_09_P031101	FBtr0110909	44643	Cnx99A	13.68	13.85	13.73	13.78	12.83	12.98	13.21	13.50	-0.63
A_09_P031116	FBtr0084728	42971	CycB3	6.31	6.95	6.36	6.45	5.32	5.45	5.65	5.72	-0.98
A_09_P031136	FBtr0087451	45556	Cyp6a17	10.71	10.89	10.71	11.00	11.93	12.10	11.69	11.51	0.98
A_09_P031151	FBtr0081181	44059	RpL30	15.73	15.64	15.72	15.68	15.78	15.79	15.84	15.81	0.11
A_09_P031166	FBtr0073720	32257	lic	10.77	10.92	10.87	10.80	10.52	10.41	10.36	10.38	-0.42
A_09_P031206	FBtr0079384	33952	nrv1	12.59	12.87	12.98	12.90	12.47	12.33	12.27	12.06	-0.55
A_09_P031216	FBtr0082799	47998	rin	11.95	12.24	12.10	12.09	11.48	11.50	11.49	11.66	-0.56
A_09_P031221	FBtr0083713	42284	P5cr	12.13	11.99	12.09	12.08	11.94	11.89	11.94	12.00	-0.13
A_09_P031246	FBtr0070849	44360	Rab18	12.58	12.65	12.67	12.74	13.23	13.22	13.13	13.02	0.49
A_09_P031256	FBtr0074921	40168	Rab8	9.74	9.92	9.82	9.83	10.24	10.18	10.21	10.30	0.40
A_09_P031271	FBtr0072427	37968	Reg-5	11.89	11.68	11.67	11.49	12.88	13.11	12.63	12.55	1.11
A_09_P031291	FBtr0073317	38565	Rpd3	12.30	12.57	12.41	12.52	12.00	12.05	12.11	12.31	-0.33
A_09_P031301	FBtr0081109	44183	ScpX	14.41	14.71	14.46	14.64	14.09	14.15	14.07	14.16	-0.44
A_09_P031311	FBtr0070894	31558	Spx	9.49	9.32	9.47	9.38	8.97	8.92	9.01	8.98	-0.45
A_09_P031316	FBtr0076108	39313	TfIIEalpha	12.27	12.51	12.40	12.58	11.77	12.11	12.02	12.10	-0.44
A_09_P031321	FBtr0073234	38527	TfIIEbeta	10.01	10.02	9.93	10.18	9.60	9.56	9.66	9.86	-0.37
A_09_P031336	FBtr0088566	35922	Vang	9.63	9.52	9.30	9.67	8.62	8.75	9.10	9.11	-0.64

A_09_P031341	FBtr0071576	37414	Xpd	9.58	9.59	9.71	9.68	9.50	9.50	9.45	9.40	-0.18
A_09_P031346	FBtr0088141	36236	Drip	12.98	12.70	12.84	12.57	13.32	13.16	13.17	13.06	0.40
A_09_P031356	FBtr0075908	39439	ara	8.85	9.22	8.97	9.03	8.70	8.60	8.60	8.61	-0.39
A_09_P031366	FBtr0086221	43862	bl	10.79	11.05	10.80	10.80	10.63	10.46	10.55	10.68	-0.28
A_09_P031386	FBtr0074007	32459	dah	7.26	8.25	7.91	8.04	6.82	6.83	6.58	6.49	-1.19
A_09_P031391	FBtr0088982	35679	dpa	11.73	11.84	11.91	11.89	11.12	11.11	11.30	11.30	-0.64
A_09_P031426	FBtr0077160	38610	lama	11.21	11.13	11.17	10.91	11.39	11.33	11.30	11.42	0.26
A_09_P031491	FBtr0079071	33759	vri	8.10	9.30	7.30	8.81	9.58	10.09	10.22	10.57	1.74
A_09_P031511	FBtr0084432	42759	ATPsyn-Cf6	15.08	14.87	15.21	15.09	14.30	14.49	14.71	14.83	-0.48
A_09_P031521	FBtr0079685	34189	Acer	12.88	12.95	12.94	12.83	11.10	11.24	12.22	12.62	-1.11
A_09_P031526	FBtr0085733	43671	Aph-4	14.31	14.38	14.23	14.25	12.68	13.24	13.13	13.49	-1.16
A_09_P031546	FBtr0071686	37461	PTP-ER	10.66	10.76	10.74	10.73	10.29	10.38	10.44	10.52	-0.32
A_09_P031556	FBtr0079076	33769	H15	7.59	7.78	7.44	7.49	7.91	8.52	8.15	8.09	0.59
A_09_P031561	FBtr0082865	41701	Ipp	10.63	10.84	10.86	10.98	10.19	10.26	10.22	10.23	-0.61
A_09_P031616	FBtr0077589	33555	Ptpa	10.47	10.54	10.50	10.66	10.09	10.17	10.28	10.38	-0.32
A_09_P031656	FBtr0112888	42824	sba	7.93	7.81	7.91	7.57	8.48	8.55	8.37	8.17	0.59
A_09_P031666	FBtr0072083	37748	angel	11.10	11.09	11.01	11.03	11.23	11.45	11.38	11.27	0.28
A_09_P031676	FBtr0072927	38321	dos	8.95	9.11	9.05	9.10	10.02	10.02	9.67	9.35	0.72
A_09_P031711	FBtr0089290	34831	smi35A	7.04	6.10	6.56	5.83	7.35	7.55	7.12	7.31	0.95
A_09_P031716	FBtr0075952	44668	SmD1	9.85	9.78	9.70	9.83	9.42	9.42	9.50	9.74	-0.27
A_09_P031721	FBtr0088505	48373	l(2)k10201	9.11	9.19	9.06	9.11	8.51	8.66	8.66	8.79	-0.46
A_09_P031726	FBtr0344111	4379834	stnB	7.90	7.49	7.63	7.61	7.92	8.10	8.31	8.37	0.52
A_09_P031736	FBtr0079355	33982	snRNP-U1-70K	11.97	11.88	11.90	11.88	11.37	11.45	11.46	11.56	-0.45
A_09_P031741	FBtr0076851	38824	smid	9.19	9.09	9.15	9.21	8.74	8.72	8.77	8.67	-0.44
A_09_P031766	FBtr0088239	36157	cag	9.29	9.52	9.29	9.52	9.06	8.99	9.14	9.14	-0.32
A_09_P031771	FBtr0089508	32796	ari-1	9.72	10.00	9.97	10.02	9.52	9.66	9.52	9.58	-0.35
A_09_P031796	FBtr0075493	39764	Zn72D	11.75	11.88	11.91	11.91	11.42	11.45	11.60	11.78	-0.30
A_09_P031806	FBtr0078133	33201	U2af38	11.67	11.62	11.75	11.69	11.16	11.33	11.29	11.38	-0.39
A_09_P031816	FBtr0086553	37228	SdhA	14.55	14.53	14.64	14.47	14.26	13.98	14.14	14.11	-0.42
A_09_P031831	FBtr0078741	40683	Rga	12.30	12.61	12.39	12.62	12.92	12.96	12.79	12.76	0.37
A_09_P031866	FBtr0071181	31762	ND75	12.56	12.67	12.52	12.61	12.09	11.72	12.17	12.43	-0.49

A_09_P031881	FBtr0082279	41296	Mcm5	9.57	9.71	9.57	9.75	8.74	8.74	8.87	9.03	-0.81
A_09_P031891	FBtr0076633	38983	RpL14	15.96	15.93	15.85	15.88	16.03	16.02	16.00	15.99	0.11
A_09_P031896	FBtr0082534	44672	Lk6	13.55	13.90	13.69	14.00	14.56	14.55	14.30	14.07	0.58
A_09_P031901	FBtr0084337	42707	klg	7.51	7.49	7.31	7.35	8.16	8.33	7.99	7.88	0.67
A_09_P031926	FBtr0089185	43833	toy	9.08	9.54	9.28	9.37	11.08	11.22	10.54	10.49	1.52
A_09_P031951	FBtr0077731	33470	colt	13.98	14.00	14.01	14.01	13.67	13.84	13.81	13.83	-0.21
A_09_P031986	FBtr0083964	42464	RpS20	15.69	15.73	15.70	15.79	15.81	15.84	15.86	15.88	0.12
A_09_P031991	FBtr0087511	36617	Rpl1	13.08	12.57	12.73	12.59	13.41	13.61	13.22	13.20	0.62
A_09_P031996	FBtr0083146	41889	Rh6	9.52	9.30	9.47	9.47	9.66	10.08	9.99	10.05	0.50
A_09_P032016	FBtr0084599	42891	Orct	12.22	12.76	12.14	12.44	10.27	10.99	11.25	11.69	-1.34
A_09_P032021	FBtr0084173	42591	ND42	12.84	12.75	12.76	12.69	12.14	11.56	12.24	12.52	-0.64
A_09_P032026	FBtr0290271	42587	CG6455	13.23	13.38	13.41	13.41	13.16	12.96	12.99	12.97	-0.34
A_09_P032031	FBtr0329956	36718	Khc-73	6.29	6.14	5.98	5.93	6.60	6.82	6.83	7.00	0.73
A_09_P032066	FBtr0301271	37869	DnaJ-60	6.43	6.65	6.58	6.55	7.13	6.97	7.10	7.02	0.51
A_09_P032081	FBtr0085540	43507	ATPsyn-gamma	13.63	13.51	13.54	13.48	12.96	12.40	13.02	13.15	-0.66
A_09_P032094	FBtr0083565	42186	14-3-3epsilon	15.48	15.44	15.55	15.26	15.17	15.13	15.22	15.26	-0.24
A_09_P032101	FBtr0087495	36614	ttv	9.17	9.31	9.27	9.22	9.10	9.14	9.03	9.08	-0.16
A_09_P032126	FBtr0073472	44072	ran	13.25	13.29	13.45	13.13	12.63	12.19	12.69	12.85	-0.69
A_09_P032151	FBtr0080213	34565	mre11	9.56	9.76	9.72	9.96	11.36	11.31	11.38	11.31	1.59
A_09_P032166	FBtr0084273	42672	loco	11.10	11.20	11.00	11.17	13.14	13.11	12.57	12.04	1.60
A_09_P032171	FBtr0088776	35771	lig	11.98	12.02	12.02	12.09	11.55	11.57	11.75	11.84	-0.35
A_09_P032186	FBtr0078324	40330	ko	10.75	10.59	10.47	10.53	11.30	11.50	11.18	10.87	0.63
A_09_P032201	FBtr0310558	44380	fok	11.28	11.59	11.16	11.57	12.46	12.74	12.20	11.84	0.91
A_09_P032236	FBtr0071638	37401	CG4050	9.25	9.36	9.22	9.39	8.03	8.30	8.78	9.02	-0.77
A_09_P032246	FBtr0080429	45668	Vha68-1	11.82	11.77	11.87	11.66	11.45	11.14	11.34	11.61	-0.40
A_09_P032256	FBtr0087774	36444	TppII	12.55	12.90	12.59	12.87	12.37	12.24	12.41	12.44	-0.36
A_09_P032271	FBtr0100255	37786	TM4SF	9.66	9.09	9.62	9.43	10.26	10.42	10.21	10.14	0.81
A_09_P032306	FBtr0072465	38017	Pdk1	9.87	9.68	9.94	9.80	11.07	11.35	10.77	10.45	1.09
A_09_P032311	FBtr0075969	39431	Pcaf	11.27	11.43	11.45	11.51	12.30	12.39	12.44	12.54	1.00
A_09_P032326	FBtr0076728	38909	Nmt	12.30	12.17	12.26	12.14	11.59	11.57	11.69	11.75	-0.57
A_09_P032336	FBtr0083259	41971	asun	8.24	8.33	8.26	8.24	7.67	7.88	7.84	8.04	-0.41

A_09_P032346	FBtr0080864	34981	Idgf3	14.00	14.21	14.09	14.18	14.46	14.62	14.43	14.31	0.33
A_09_P032356	FBtr0080860	34978	Idgf1	11.37	10.87	11.02	10.91	12.54	12.74	12.16	11.56	1.21
A_09_P032361	FBtr0089577	33789	GluRIIB	8.78	8.97	9.03	8.76	8.50	8.50	8.51	8.68	-0.34
A_09_P032386	FBtr0086542	37233	Fak	10.60	10.60	10.74	10.78	10.35	10.27	10.31	10.15	-0.41
A_09_P032391	FBtr0080369	34658	Elf	13.10	13.24	13.16	13.15	12.90	12.84	12.97	13.07	-0.22
A_09_P032416	FBtr0079663	34167	emb	9.72	9.79	9.93	9.69	9.66	9.52	9.50	9.41	-0.26
A_09_P032436	FBtr0073938	44079	Ag5r2	15.07	15.32	14.91	15.19	15.75	15.67	15.66	15.63	0.56
A_09_P032471	FBtr0078016	33265	kraken	13.27	13.64	13.47	13.67	14.30	14.37	14.07	13.91	0.65
A_09_P032481	FBtr0087564	36587	SelD	12.96	12.98	13.00	12.98	12.30	12.30	12.61	12.90	-0.45
A_09_P032526	FBtr0089500	37746	Pal2	8.68	8.73	8.60	8.47	9.28	8.97	9.06	9.03	0.47
A_09_P032536	FBtr0076585	39014	Mcm7	9.70	9.92	9.78	10.03	9.13	8.74	9.03	9.28	-0.82
A_09_P032541	FBtr0077059	38702	Lcp65Ag2	15.84	15.72	15.81	15.80	15.99	16.03	15.96	16.03	0.21
A_09_P032546	FBtr0077058	38703	Lcp65Ag1	15.64	15.82	15.61	15.73	15.90	15.90	15.85	15.89	0.18
A_09_P032581	FBtr0076995	38709	Lcp65Aa	10.30	10.80	10.81	10.91	9.96	10.05	9.44	8.78	-1.15
A_09_P032591	FBtr0071168	31760	Trxr-1	13.61	13.37	13.43	13.40	13.62	13.68	13.67	13.85	0.25
A_09_P032601	FBtr0334940	3355041	eIF-4B	11.38	11.48	11.77	11.24	11.04	10.44	10.92	10.98	-0.62
A_09_P032606	FBtr0113681	3355041	eIF-4B	13.62	13.66	13.78	13.63	13.34	13.21	13.36	13.54	-0.31
A_09_P032616	FBtr0112899	33636	Atet	10.60	10.24	10.15	10.08	11.83	12.31	11.62	11.22	1.48
A_09_P032631	FBtr0087594	36547	Aats-phe	13.04	12.97	13.07	12.97	12.55	12.58	12.64	12.78	-0.37
A_09_P032641	FBtr0301151	34059	CG7179	10.47	7.60	9.43	7.86	10.76	10.96	11.41	11.68	2.37
A_09_P032656	FBtr0083361	42015	Scp2	12.78	12.96	13.05	12.57	12.28	12.16	12.02	11.95	-0.74
A_09_P032726	FBtr0074511	32789	scu	15.84	15.69	16.01	15.79	15.13	15.03	15.46	15.58	-0.53
A_09_P032736	FBtr0075328	39879	nudC	13.18	13.16	13.21	13.08	12.40	12.12	12.73	13.00	-0.59
A_09_P032746	FBtr0088153	45680	Tapdelta	15.09	15.13	15.16	15.05	14.04	14.16	14.62	14.90	-0.68
A_09_P032751	FBtr0080422	47396	Tor	12.12	12.22	12.23	12.34	11.51	11.61	11.84	11.96	-0.50
A_09_P032771	FBtr0088641	44086	Dmn	12.31	12.51	12.34	12.55	12.13	12.14	12.14	12.25	-0.26
A_09_P032806	FBtr0072253	45021	Zfrp8	11.21	11.07	11.15	11.22	11.30	11.63	11.41	11.45	0.29
A_09_P032846	FBtr0079030	45682	eIF-3p40	14.58	14.58	14.55	14.62	14.75	14.79	14.76	14.81	0.20
A_09_P032876	FBtr0077524	33569	Thor	12.77	12.21	12.38	12.29	14.67	14.94	14.45	14.16	2.14
A_09_P032921	FBtr0080930	35016	Cas	11.19	11.37	11.23	11.40	10.44	10.42	10.70	10.90	-0.68
A_09_P032956	FBtr0072391	37987	CG3760	8.09	8.05	7.97	7.69	7.33	7.00	7.41	7.56	-0.63

A_09_P032961	FBtr0083358	42020	CG10340	13.69	13.49	13.66	13.50	13.22	13.17	13.30	13.48	-0.29
A_09_P032971	FBtr0085839	43741	CG1910	11.67	12.05	11.94	11.94	11.52	11.56	11.29	11.52	-0.42
A_09_P032976	FBtr0074559	32821	Tsf1	13.68	14.64	14.17	14.63	15.53	14.99	15.39	15.26	1.01
A_09_P032991	FBtr0088438	36041	Pka-R2	9.79	9.96	10.00	9.62	8.94	8.80	8.70	8.73	-1.05
A_09_P033006	FBtr0071543	49815	Cht4	11.69	11.09	10.79	11.46	14.60	14.29	14.03	13.43	2.83
A_09_P033026	FBtr0076018	39396	Adk1	13.28	12.62	12.80	12.66	13.59	13.16	13.53	13.70	0.65
A_09_P033071	FBtr0088959	35686	Orc1	8.35	8.46	8.34	8.37	6.90	7.07	7.53	7.78	-1.06
A_09_P033076	FBtr0074961	40145	Oat	13.34	14.07	13.75	14.11	11.13	11.12	12.22	12.67	-2.03
A_09_P033091	FBtr0084874	43026	Cad96Ca	11.48	10.63	10.60	10.43	11.69	11.90	11.90	12.07	1.11
A_09_P033106	FBtr0078439	40429	CycH	10.77	10.67	10.82	10.85	10.55	10.43	10.56	10.63	-0.24
A_09_P033111	FBtr0070684	44409	Cbp80	10.65	10.82	10.75	10.76	10.22	10.18	10.29	10.49	-0.45
A_09_P033116	FBtr0083551	42166	Cbp20	11.03	10.91	11.08	11.16	10.30	10.47	10.65	10.94	-0.46
A_09_P033126	FBtr0086182	35609	vimar	9.00	8.86	8.96	8.76	8.56	8.79	8.63	8.59	-0.26
A_09_P033141	FBtr0071828	37562	qkr58E-2	11.91	11.84	11.98	11.85	11.47	11.55	11.49	11.44	-0.40
A_09_P033146	FBtr0071843	37561	qkr58E-1	8.63	8.55	8.51	8.51	7.82	7.67	8.03	8.27	-0.60
A_09_P033151	FBtr0331464	36966	qkr54B	7.56	7.68	7.60	7.65	7.20	7.40	7.38	7.40	-0.28
A_09_P033176	FBtr0089685	40675	CRMP	10.31	10.62	10.84	10.62	8.82	8.76	9.31	9.64	-1.47
A_09_P033191	FBtr0072287	37858	gek	10.62	10.78	10.71	10.77	10.43	10.15	10.44	10.58	-0.32
A_09_P033196	FBtr0083637	44233	fray	12.04	11.95	12.01	11.93	12.72	12.58	12.33	12.24	0.49
A_09_P033246	FBtr0088499	35998	Uba1	12.52	12.79	12.59	12.67	12.46	12.35	12.33	12.36	-0.27
A_09_P033261	FBtr0072185	37849	RpL39	15.40	15.19	15.26	15.28	15.52	15.53	15.61	15.54	0.27
A_09_P033276	FBtr0075629	39628	Prosbeta2	11.64	11.55	11.43	11.36	10.68	10.20	10.97	11.26	-0.72
A_09_P033281	FBtr0089998	36018	Prosalpha7	14.37	14.52	14.35	14.46	13.65	13.65	14.01	14.33	-0.52
A_09_P033296	FBtr0085048	43215	amon	7.91	7.28	7.46	7.40	8.17	8.22	8.14	8.12	0.65
A_09_P033301	FBtr0088482	36017	Orc6	8.71	8.51	8.53	8.47	8.07	8.27	8.24	8.25	-0.35
A_09_P033306	FBtr0072425	37970	Orc4	8.13	8.04	8.22	8.05	7.57	7.76	7.83	7.83	-0.36
A_09_P033316	FBtr0075514	39740	CG17029	13.34	13.45	13.45	13.48	13.18	13.21	13.00	12.90	-0.36
A_09_P033326	FBtr0075516	39742	CG17027	11.96	12.84	12.53	12.89	11.39	11.46	11.32	11.30	-1.19
A_09_P033366	FBtr0075497	39758	CG5235	6.27	6.68	6.38	6.48	5.74	5.36	5.64	5.50	-0.89
A_09_P033371	FBtr0302503	39759	ClC-c	11.12	11.33	11.22	11.37	10.93	10.77	10.81	10.87	-0.41
A_09_P033391	FBtr0075495	39763	IntS9	9.99	10.13	9.90	10.18	9.32	9.25	9.64	9.83	-0.54

A_09_P033406	FBtr0089713	39770	CG16838	9.50	9.44	9.37	9.39	8.84	8.98	8.88	9.14	**-0.46**
A_09_P033411	FBtr0075490	39771	CG5157	7.12	6.55	6.10	5.88	8.79	8.69	8.27	7.74	**1.96**
A_09_P033436	FBtr0075483	39776	PDCD-5	12.51	12.34	12.34	12.33	11.55	11.49	11.83	12.00	**-0.66**
A_09_P033581	FBtr0110866	39808	CG4998	14.29	7.10	13.64	6.90	15.30	14.98	14.84	14.65	**4.46**
A_09_P033596	FBtr0075381	39811	CG4933	11.23	11.16	11.10	11.09	10.38	10.21	10.66	10.89	**-0.61**
A_09_P033641	FBtr0075387	39821	RAF2	8.47	8.48	8.42	8.21	7.83	7.59	7.89	7.95	**-0.58**
A_09_P033661	FBtr0075411	39828	CG4573	9.84	9.56	9.74	9.51	9.14	9.05	9.22	9.10	**-0.54**
A_09_P033686	FBtr0075395	39844	Smn	11.60	11.60	11.70	11.79	11.02	11.09	11.29	11.28	**-0.51**
A_09_P033696	FBtr0075365	39847	Syx8	5.69	6.26	5.85	5.92	6.46	6.54	6.60	6.79	**0.66**
A_09_P033706	FBtr0075361	39854	CG4098	10.18	10.52	10.28	10.54	9.97	9.89	9.90	9.79	**-0.49**
A_09_P033711	FBtr0075356	39860	Baldspot	12.30	12.32	12.24	12.17	10.99	11.02	11.56	11.97	**-0.87**
A_09_P033736	FBtr0075321	39872	CG9701	13.22	13.99	13.84	14.08	12.82	13.23	12.76	12.44	**-0.97**
A_09_P033756	FBtr0075344	39878	CG9674	12.42	12.84	12.66	12.72	13.10	13.06	12.98	12.96	**0.37**
A_09_P033771	FBtr0075339	39882	CG9669	12.02	12.03	12.00	12.03	10.80	11.09	11.45	11.67	**-0.77**
A_09_P033776	FBtr0075330	39883	CG9715	11.73	11.74	11.61	11.71	12.18	12.22	11.94	11.86	**0.35**
A_09_P033796	FBtr0302785	39889	Lmpt	12.44	12.86	12.95	12.78	12.22	12.29	11.76	11.81	**-0.74**
A_09_P033801	FBtr0302786	39889	Lmpt	11.83	12.29	12.31	12.46	11.51	11.31	11.25	11.13	**-0.92**
A_09_P033856	FBtr0075294	39903	CG6664	8.80	9.18	9.07	9.10	8.45	8.73	8.52	8.45	**-0.50**
A_09_P033881	FBtr0290221	39909	Ilp8	7.05	4.77	6.13	5.08	8.44	8.67	9.01	9.15	**3.06**
A_09_P033911	FBtr0075251	39922	CG6512	13.43	13.43	13.44	13.39	12.62	12.68	12.84	13.09	**-0.61**
A_09_P033951	FBtr0075245	39930	CG6479	11.24	11.00	11.13	10.99	10.34	9.95	10.56	10.87	**-0.66**
A_09_P033956	FBtr0075243	39931	CG13727	7.46	6.19	7.07	6.38	7.95	8.54	8.09	8.11	**1.40**
A_09_P034001	FBtr0075220	39948	CG7603	13.74	13.74	13.75	13.76	13.38	13.34	13.32	13.40	**-0.39**
A_09_P034011	FBtr0075222	39950	CG7580	15.49	15.34	15.52	15.47	15.07	15.00	15.18	15.18	**-0.34**
A_09_P034086	FBtr0075195	39971	CG6052	6.19	5.96	5.89	6.01	4.61	4.93	5.31	5.47	**-0.93**
A_09_P034096	FBtr0075194	39974	CG6034	5.39	6.67	6.48	6.73	3.08	3.81	4.16	4.16	**-2.51**
A_09_P034136	FBtr0075161	39986	CG5567	12.59	12.51	12.63	12.50	12.01	11.80	12.20	12.45	**-0.44**
A_09_P034146	FBtr0075140	39988	CG7430	14.14	14.24	14.23	14.28	13.46	13.50	13.65	13.71	**-0.64**
A_09_P034151	FBtr0075141	39989	CG7441	7.96	7.67	7.82	7.77	7.51	6.98	7.25	7.32	**-0.54**
A_09_P034166	FBtr0075157	39992	CG5506	13.20	13.09	13.23	13.23	15.47	14.34	14.99	14.60	**1.66**
A_09_P034171	FBtr0290228	39993	CG16775	10.44	8.24	10.01	8.92	12.95	11.88	12.47	12.07	**2.94**

A_09_P034191	FBtr0075144	39997	CG14353	9.49	9.55	9.60	9.63	9.12	8.93	9.14	9.23	**-0.46**
A_09_P034256	FBtr0075119	40016	skl	5.81	6.56	6.16	6.01	5.17	5.02	4.74	4.77	**-1.21**
A_09_P034301	FBtr0075049	40032	MYPT-75D	10.77	10.97	10.85	10.88	12.24	11.92	11.66	11.24	**0.90**
A_09_P034306	FBtr0113179	40035	Sgf11	10.84	10.88	10.95	10.96	10.39	10.44	10.46	10.48	**-0.46**
A_09_P034311	FBtr0075092	40036	Chmp1	11.95	12.23	12.05	12.33	13.61	13.66	13.31	12.93	**1.23**
A_09_P034316	FBtr0075091	40037	Cyp12c1	11.94	12.01	12.22	11.80	13.77	13.47	13.36	13.10	**1.43**
A_09_P034341	FBtr0075054	40042	MED11	9.36	9.54	9.41	9.64	9.76	9.98	9.93	10.06	**0.45**
A_09_P034366	FBtr0075082	40049	Indy	14.05	14.19	14.03	13.96	13.86	13.46	13.53	13.43	**-0.49**
A_09_P034376	FBtr0308812	40051	CG14074	7.47	7.37	7.29	7.37	6.55	6.73	6.93	7.04	**-0.56**
A_09_P034381	FBtr0075062	40052	dysb	9.03	9.31	9.05	9.21	8.76	8.69	8.73	8.88	**-0.38**
A_09_P034386	FBtr0075063	40053	CG6852	12.80	12.78	12.78	12.76	12.01	12.05	12.02	11.84	**-0.80**
A_09_P034391	FBtr0075077	40056	CG3961	10.47	11.68	11.34	11.60	9.34	9.08	9.18	9.17	**-2.08**
A_09_P034401	FBtr0075076	40059	CG3902	14.74	15.12	14.98	15.00	13.82	14.06	13.90	13.92	**-1.03**
A_09_P034411	FBtr0075075	40061	CG3893	8.82	8.64	8.83	8.78	8.07	7.92	8.29	8.43	**-0.59**
A_09_P034451	FBtr0075023	40070	CG6836	10.77	9.36	9.47	9.46	10.74	11.06	11.29	11.57	**1.40**
A_09_P034476	FBtr0075040	40075	CG18136	11.24	10.94	11.18	11.07	10.58	10.42	10.31	10.10	**-0.75**
A_09_P034481	FBtr0075037	40079	CG3797	10.49	10.31	10.48	10.43	9.65	9.92	9.71	9.64	**-0.70**
A_09_P034486	FBtr0075025	40080	CG6812	11.87	12.50	12.37	12.53	10.15	10.18	10.57	10.71	**-1.92**
A_09_P034491	FBtr0075035	40081	Mkp3	8.47	8.46	8.36	8.25	10.08	10.13	9.56	9.09	**1.33**
A_09_P034496	FBtr0075026	40083	MESR6	11.17	11.49	11.25	11.43	9.95	9.89	10.63	11.03	**-0.96**
A_09_P034501	FBtr0075034	40084	CG11577	11.78	11.90	11.99	11.94	10.10	10.23	11.01	11.51	**-1.18**
A_09_P034506	FBtr0075033	40085	CG10424	11.69	12.26	12.04	12.08	11.59	11.44	11.45	11.49	**-0.52**
A_09_P034531	FBtr0074990	40094	Bet1	11.71	11.73	11.80	11.85	10.75	11.00	11.29	11.57	**-0.62**
A_09_P034541	FBtr0075017	40097	CG9629	12.56	12.87	12.74	12.84	12.24	12.17	12.13	12.03	**-0.61**
A_09_P034611	FBtr0074979	40118	CG9451	11.39	11.25	11.16	11.36	13.15	13.19	12.88	12.60	**1.67**
A_09_P034631	FBtr0113180	40122	Cpr76Bc	8.44	9.49	9.23	9.60	7.44	8.13	7.30	6.74	**-1.79**
A_09_P034656	FBtr0074972	40130	CG9231	12.91	12.93	12.83	12.93	12.11	11.94	12.22	12.48	**-0.71**
A_09_P034661	FBtr0074940	40131	CG9330	13.38	13.48	13.43	13.43	13.55	13.61	13.60	13.62	**0.16**
A_09_P034701	FBtr0074963	40143	wnd	11.06	11.02	10.90	10.86	10.77	10.72	10.53	10.29	**-0.38**
A_09_P034746	FBtr0074927	40158	CG14103	11.47	11.49	11.44	11.56	10.66	10.45	11.02	11.32	**-0.63**
A_09_P034751	FBtr0074879	40159	CG15881	12.25	12.01	12.09	12.03	11.51	11.41	11.71	11.96	**-0.45**

A_09_P034756	FBtr0074925	40161	Cyp305a1	10.10	8.58	9.36	8.67	10.79	10.77	10.43	10.18	**1.36**
A_09_P034761	FBtr0089596	40163	Fibp	11.33	11.36	11.41	11.40	10.92	10.87	11.00	11.13	**-0.40**
A_09_P034771	FBtr0074891	40172	Prp3	10.84	11.13	11.03	11.17	10.72	10.79	10.69	10.83	**-0.28**
A_09_P034781	FBtr0074894	40176	CG7770	13.55	13.51	13.55	13.45	12.72	12.86	13.01	13.14	**-0.58**
A_09_P034786	FBtr0074895	40177	Grasp65	10.53	11.01	10.46	10.70	9.69	9.35	9.80	10.30	**-0.89**
A_09_P034801	FBtr0074913	40181	CG14182	9.58	9.61	9.73	9.65	8.70	8.66	9.17	9.46	**-0.64**
A_09_P034831	FBtr0302049	40188	CG7433	6.15	6.04	5.95	5.51	3.53	3.78	4.19	4.77	**-1.84**
A_09_P034856	FBtr0074872	40193	CG14184	10.04	10.09	10.14	10.18	9.92	9.84	9.97	9.64	**-0.27**
A_09_P034866	FBtr0074870	40196	CG14186	7.04	6.77	6.74	6.39	8.71	8.86	8.26	7.91	**1.70**
A_09_P034906	FBtr0302714	40205	CG42674	6.76	6.70	6.10	6.12	7.59	7.71	7.47	7.12	**1.05**
A_09_P034916	FBtr0074820	40207	Ssk	12.83	12.46	12.57	12.46	14.06	13.81	13.63	13.54	**1.18**
A_09_P034926	FBtr0074860	40209	obst-F	11.78	11.99	12.11	12.25	10.90	11.57	11.16	11.15	**-0.84**
A_09_P034931	FBtr0074859	40210	CG7298	15.76	15.52	15.56	15.43	16.15	16.08	15.97	15.95	**0.47**
A_09_P034946	FBtr0074856	40213	CG7017	12.38	12.28	12.56	12.44	13.13	12.68	12.96	12.96	**0.52**
A_09_P034951	FBtr0074854	40214	CG6933	15.52	15.47	15.57	15.43	16.12	15.67	15.97	15.87	**0.41**
A_09_P034966	FBtr0074825	40217	Su(var)3-3	8.13	8.00	8.18	8.04	7.03	7.11	7.52	7.75	**-0.74**
A_09_P034976	FBtr0306327	40220	CG17233	8.52	8.80	8.65	8.71	7.74	8.08	7.86	7.90	**-0.78**
A_09_P035001	FBtr0074838	40231	CG6597	10.98	11.24	11.32	11.51	10.65	10.58	10.68	10.72	**-0.61**
A_09_P035021	FBtr0074844	40238	CG6434	5.22	6.65	5.73	6.11	2.88	3.84	3.47	3.55	**-2.49**
A_09_P035036	FBtr0078179	40241	CG5618	12.02	12.21	12.11	12.25	12.32	12.50	12.53	12.58	**0.33**
A_09_P035091	FBtr0078194	40262	CG5872	8.72	8.67	8.70	8.61	8.41	8.16	8.33	8.54	**-0.31**
A_09_P035106	FBtr0078224	40265	CG5199	9.29	9.48	9.36	9.34	8.99	9.01	9.08	9.20	**-0.30**
A_09_P035121	FBtr0078197	40268	CG5955	11.29	10.58	10.98	10.68	9.97	10.37	9.88	9.73	**-0.89**
A_09_P035136	FBtr0078221	40271	CG5130	8.19	8.04	7.45	7.64	5.38	5.70	5.50	5.73	**-2.25**
A_09_P035161	FBtr0078217	40278	CG5059	10.52	11.00	10.41	10.73	12.58	12.47	12.20	11.56	**1.54**
A_09_P035186	FBtr0078213	40283	Rcd2	8.93	8.75	8.48	8.51	10.80	10.60	10.16	9.68	**1.64**
A_09_P035236	FBtr0078276	40296	CG11396	11.19	11.28	11.22	11.31	10.74	10.52	10.75	11.06	**-0.48**
A_09_P035261	FBtr0078256	40302	CG3680	9.82	10.09	10.11	10.14	9.68	9.28	9.52	9.45	**-0.55**
A_09_P035356	FBtr0078297	40339	CG10512	13.33	13.83	13.83	14.06	13.14	12.82	13.09	13.11	**-0.73**
A_09_P035366	FBtr0307387	40342	CG10508	7.72	7.83	7.85	8.02	8.41	8.82	8.47	8.38	**0.66**
A_09_P035391	FBtr0078336	40351	pzg	12.40	12.42	12.39	12.50	11.97	11.99	12.21	12.25	**-0.32**

A_09_P035456	FBtr0088102	40373	Syx6	9.01	9.08	8.97	9.06	9.42	9.45	9.31	9.31	**0.34**
A_09_P035486	FBtr0078357	40385	Cdk12	10.90	10.88	10.99	10.99	10.71	10.69	10.69	10.70	**-0.24**
A_09_P035496	FBtr0078382	40390	CoVIII	14.84	14.83	14.85	14.87	14.42	14.38	14.58	14.77	**-0.31**
A_09_P035516	FBtr0078371	40394	CG7634	7.40	6.73	7.19	7.13	4.92	6.24	5.32	6.04	**-1.48**
A_09_P035521	FBtr0078377	40395	CG7172	12.49	12.47	12.59	12.38	12.75	12.71	12.80	12.79	**0.28**
A_09_P035536	FBtr0333391	40400	CG11307	8.78	8.94	8.66	8.75	8.30	8.23	8.31	8.51	**-0.45**
A_09_P035546	FBtr0078422	40402	CG11306	12.09	12.31	12.18	12.27	11.61	11.49	11.64	11.71	**-0.60**
A_09_P035551	FBtr0078464	40403	MED1	8.00	8.59	8.31	8.43	7.68	7.87	7.71	7.91	**-0.54**
A_09_P035556	FBtr0078423	40404	ORMDL	9.42	9.49	9.45	9.56	8.97	9.05	9.03	8.98	**-0.47**
A_09_P035576	FBtr0078431	40411	CG11248	8.57	8.69	8.57	8.59	8.38	8.40	8.30	8.40	**-0.24**
A_09_P035611	FBtr0078454	40420	CG14567	10.04	9.51	9.78	10.01	13.59	13.55	12.82	11.60	**3.06**
A_09_P035631	FBtr0078450	40424	Syn1	7.82	7.59	7.59	7.52	8.35	8.54	8.35	8.26	**0.75**
A_09_P035661	FBtr0078445	40434	P5CDh1	9.56	9.88	9.84	9.96	8.40	8.07	8.57	8.62	**-1.39**
A_09_P035681	FBtr0078472	40439	CG14562	7.85	8.01	8.09	8.02	7.40	7.34	7.63	7.64	**-0.49**
A_09_P035711	FBtr0078485	40449	CG7133	10.20	10.46	10.53	10.47	9.89	10.08	9.91	9.92	**-0.46**
A_09_P035741	FBtr0078508	40468	laza	10.02	10.09	10.10	10.14	11.72	11.93	11.28	10.92	**1.37**
A_09_P035746	FBtr0078504	40469	CG11438	11.34	10.78	10.66	10.87	11.86	12.50	12.38	12.57	**1.42**
A_09_P035791	FBtr0078517	40479	CG14457	9.63	9.20	9.25	9.02	10.13	10.17	10.08	10.00	**0.82**
A_09_P035826	FBtr0078522	40486	CG11370	16.01	15.59	15.82	15.55	16.21	16.25	16.18	16.05	**0.43**
A_09_P035831	FBtr0078539	40487	ArfGAP3	11.35	11.54	11.48	11.48	10.92	11.08	11.17	11.33	**-0.33**
A_09_P035841	FBtr0078537	40490	CG14450	9.02	8.84	8.93	8.90	8.56	8.48	8.54	8.68	**-0.36**
A_09_P035851	FBtr0078536	40492	CG11241	9.07	10.14	9.68	10.06	8.91	8.73	8.69	8.66	**-0.99**
A_09_P035911	FBtr0078561	40514	Mes2	9.07	9.06	8.96	8.89	8.73	8.86	8.68	8.56	**-0.29**
A_09_P035926	FBtr0078962	40522	CG12581	12.02	12.01	11.88	12.01	12.21	12.52	12.31	12.49	**0.40**
A_09_P035941	FBtr0078965	40527	aux	10.80	10.82	10.90	10.94	10.69	10.72	10.71	10.66	**-0.17**
A_09_P035946	FBtr0078986	40528	DhpD	12.24	12.41	12.33	12.29	11.77	11.93	11.79	11.91	**-0.47**
A_09_P035951	FBtr0078985	40529	CG14641	12.19	12.09	12.25	12.09	11.85	11.60	11.84	11.86	**-0.37**
A_09_P035981	FBtr0336480	40538	CG1092	11.69	12.18	11.94	12.00	10.07	10.30	10.86	11.24	**-1.34**
A_09_P035991	FBtr0078959	40540	CG9780	12.41	11.36	12.13	11.43	13.22	13.25	13.09	12.88	**1.28**
A_09_P036046	FBtr0078919	40555	CG14646	9.12	9.38	9.14	9.18	8.46	8.46	8.64	8.98	**-0.57**
A_09_P036051	FBtr0078950	40556	CG9855	9.61	9.75	9.72	9.82	9.15	9.08	9.22	9.31	**-0.53**

A_09_P036056	FBtr0290222	40558	CG14647	10.84	11.11	11.04	11.49	10.65	10.47	10.55	10.50	-0.58
A_09_P036071	FBtr0078944	40563	eIF3-S10	14.18	14.32	14.28	14.31	14.44	14.47	14.38	14.47	0.17
A_09_P036076	FBtr0078925	40564	CG1074	10.18	10.09	10.21	9.86	9.57	9.95	9.49	9.66	-0.42
A_09_P036081	FBtr0078943	40565	CG9804	11.24	11.34	11.34	11.35	9.94	9.96	10.31	10.39	-1.17
A_09_P036086	FBtr0078926	40566	CG14650	10.96	11.36	11.11	11.27	10.66	10.70	10.76	10.99	-0.39
A_09_P036121	FBtr0078861	40582	CG12001	9.59	10.05	9.95	9.97	7.24	7.29	8.00	8.25	-2.20
A_09_P036146	FBtr0303915	40596	ctrip	9.42	9.55	9.68	9.51	9.10	9.24	9.22	9.29	-0.33
A_09_P036166	FBtr0078875	40602	CG14657	8.73	8.78	8.73	8.88	8.38	8.63	8.47	8.51	-0.28
A_09_P036181	FBtr0078848	40611	CG14661	9.08	9.94	9.64	9.81	8.35	8.64	8.33	8.13	-1.25
A_09_P036196	FBtr0078846	40614	CG14662	6.33	6.50	6.40	6.35	5.05	4.10	4.65	4.04	-1.93
A_09_P036206	FBtr0078842	40617	CG12007	11.71	11.79	11.73	11.72	12.48	12.17	12.06	12.10	0.46
A_09_P036226	FBtr0078774	40622	CG1116	9.16	9.21	9.29	9.31	8.84	8.87	8.92	9.12	-0.30
A_09_P036231	FBtr0078828	40623	CG2604	12.39	12.63	12.55	12.72	12.12	12.26	12.36	12.29	-0.32
A_09_P036256	FBtr0078823	40628	CG12163	12.68	13.10	12.68	12.92	13.75	13.71	13.64	13.52	0.81
A_09_P036306	FBtr0078802	40642	hd	9.98	9.82	9.94	9.86	9.17	9.47	9.55	9.67	-0.44
A_09_P036376	FBtr0078761	40666	CG2519	9.36	9.63	9.27	9.56	9.14	9.07	9.10	9.03	-0.37
A_09_P036381	FBtr0078688	40669	Snm1	6.91	7.14	6.88	7.01	6.61	6.59	6.53	6.57	-0.41
A_09_P036391	FBtr0078754	40671	CG14671	12.31	12.20	12.27	12.23	11.80	11.91	11.97	12.01	-0.33
A_09_P036401	FBtr0078753	40673	CG2931	11.65	11.51	11.59	11.57	11.17	11.31	11.29	11.34	-0.30
A_09_P036411	FBtr0078699	40677	rev7	8.24	8.52	8.46	8.54	8.94	9.10	8.97	9.23	0.62
A_09_P036451	FBtr0078708	40690	CG12171	12.97	12.98	13.10	13.00	13.99	13.81	13.56	13.42	0.68
A_09_P036506	FBtr0078724	40707	CG2100	9.90	9.73	9.81	9.82	9.58	9.36	9.49	9.63	-0.30
A_09_P036511	FBtr0078721	40708	CG1236	12.42	13.02	12.51	12.89	11.58	11.63	12.01	12.35	-0.81
A_09_P036521	FBtr0078678	40711	CG2091	11.10	11.20	11.22	11.20	10.52	10.55	10.69	10.83	-0.53
A_09_P036531	FBtr0078624	40714	jagn	12.10	12.10	12.16	12.18	11.33	11.25	11.59	11.71	-0.67
A_09_P036541	FBtr0078669	40716	CG2051	8.73	8.97	8.76	8.75	8.17	7.65	8.14	8.46	-0.70
A_09_P036571	FBtr0078667	40723	Hpr1	8.84	8.77	8.69	8.81	8.39	8.27	8.43	8.58	-0.36
A_09_P036656	FBtr0078590	40754	NPFR1	6.94	6.42	6.07	5.94	8.32	8.05	7.88	7.93	1.70
A_09_P036771	FBtr0081711	40780	CG17919	12.51	12.47	12.48	12.57	11.81	12.14	12.14	12.34	-0.40
A_09_P036776	FBtr0081737	40781	SmD2	10.79	10.80	10.77	10.86	10.30	10.04	10.33	10.51	-0.51
A_09_P036791	FBtr0081717	40786	CG10286	11.99	11.62	11.75	11.62	12.81	12.97	12.64	12.55	1.00

A_09_P036796	FBtr0081734	40787	CG1041	10.38	11.02	10.64	10.83	9.63	9.93	10.01	10.14	-0.79
A_09_P036801	FBtr0301268	40788	CG42564	5.73	6.15	5.73	5.84	4.04	4.54	4.50	5.43	-1.23
A_09_P036811	FBtr0289939	40792	CG1021	10.68	10.28	10.31	10.23	11.67	11.81	11.34	10.81	1.03
A_09_P036826	FBtr0081729	40796	Neurochondrin	14.11	14.49	14.39	14.39	13.16	13.19	13.24	13.32	-1.12
A_09_P036891	FBtr0081645	40841	CG1105	10.34	10.56	10.58	10.48	9.52	9.63	9.90	10.21	-0.68
A_09_P036911	FBtr0081638	40846	Dpck	11.65	11.53	11.58	11.67	10.99	10.95	11.05	11.10	-0.58
A_09_P036951	FBtr0081568	40857	CG2656	11.12	11.01	11.02	11.05	11.20	11.19	11.23	11.20	0.15
A_09_P036956	FBtr0081606	40860	MAGE	9.69	9.73	9.83	9.85	9.43	9.21	9.35	9.39	-0.43
A_09_P036976	FBtr0081603	40866	CG14606	8.06	8.31	8.22	7.98	9.80	9.61	9.38	8.99	1.30
A_09_P037006	FBtr0081598	40872	CG1227	10.72	10.55	10.67	10.58	10.31	10.21	10.33	10.49	-0.29
A_09_P037061	FBtr0081780	40916	pyd3	13.00	13.07	13.02	13.23	11.26	11.22	11.85	12.14	-1.46
A_09_P037121	FBtr0081765	40933	CG2943	11.42	11.19	11.28	11.22	10.74	10.68	10.92	11.10	-0.42
A_09_P037126	FBtr0300685	40938	CG10445	4.73	4.83	5.53	4.91	6.15	6.20	6.23	6.32	1.23
A_09_P037131	FBtr0081824	40941	CD98hc	14.01	14.18	14.14	14.03	14.34	14.36	14.27	14.22	0.21
A_09_P037156	FBtr0081785	40947	CG3223	11.03	11.41	11.18	11.29	10.91	10.73	10.87	10.98	-0.35
A_09_P037186	FBtr0081789	40955	CG7918	6.48	5.81	6.20	5.31	6.90	6.93	6.83	6.88	0.94
A_09_P037211	FBtr0081794	40961	Gie	10.12	10.34	10.27	10.16	9.92	9.65	9.62	9.55	-0.54
A_09_P037216	FBtr0081795	40963	CG7800	10.69	10.48	10.53	10.41	9.52	9.18	9.88	10.17	-0.84
A_09_P037221	FBtr0302178	40964	CG18249	8.57	9.88	9.36	9.62	6.56	7.59	7.26	7.55	-2.12
A_09_P037226	FBtr0081798	40965	DNApol-iota	6.24	6.31	5.82	5.69	6.57	6.91	6.94	6.82	0.80
A_09_P037231	FBtr0081807	40966	Ada2b	10.70	11.03	10.83	11.05	11.65	11.99	11.56	11.44	0.76
A_09_P037236	FBtr0081806	40967	CG9636	8.89	9.41	8.89	9.25	8.24	8.27	8.33	8.62	-0.75
A_09_P037271	FBtr0071423	31940	CG15312	8.19	8.17	8.36	8.31	8.67	9.15	8.79	8.61	0.55
A_09_P037311	FBtr0071441	31958	CG2909	4.86	5.03	3.77	4.04	6.50	7.00	6.69	6.84	2.33
A_09_P037336	FBtr0071465	31967	Psf3	8.60	8.63	8.53	8.53	8.13	8.01	8.15	8.27	-0.43
A_09_P037356	FBtr0071460	31976	nocte	10.90	11.17	11.07	11.17	9.26	9.85	9.99	10.22	-1.25
A_09_P037371	FBtr0071451	31979	PPP4R2r	10.86	11.71	11.09	11.53	9.79	9.88	10.24	10.67	-1.15
A_09_P037426	FBtr0071502	32002	Rph	7.03	6.76	6.79	6.38	7.91	7.76	7.62	7.52	0.96
A_09_P037436	FBtr0073415	32009	Imp	9.25	9.13	9.28	9.10	9.54	9.83	9.49	9.50	0.40
A_09_P037446	FBtr0073379	32013	CG17333	12.77	12.77	12.69	12.86	11.93	12.09	12.29	12.49	-0.57
A_09_P037461	FBtr0073409	32016	sofe	7.37	7.76	7.70	7.83	6.75	6.40	6.60	7.01	-0.97

A_09_P037466	FBtr0073381	32017	CG2186	9.96	10.35	10.15	10.13	9.49	9.63	9.48	9.88	-0.53
A_09_P037471	FBtr0073382	32018	CG2157	13.05	11.70	12.69	11.78	13.91	14.14	13.58	13.22	1.41
A_09_P037491	FBtr0073403	32021	CG1582	11.44	11.46	11.57	11.36	12.30	12.65	12.17	12.27	0.89
A_09_P037536	FBtr0073396	32035	CG1552	7.84	7.47	7.62	7.67	9.11	9.60	8.85	8.45	1.35
A_09_P037551	FBtr0073392	32038	CG15203	10.62	10.69	10.55	10.65	9.76	9.67	9.94	10.25	-0.72
A_09_P037561	FBtr0073428	32041	CG2076	13.20	13.25	13.34	13.29	12.63	12.71	12.93	13.05	-0.44
A_09_P037591	FBtr0073473	32051	CG15199	10.57	10.18	10.18	10.28	8.70	8.84	8.60	8.22	-1.71
A_09_P037651	FBtr0073448	32070	CG1657	10.51	10.69	10.65	10.65	11.57	11.55	11.21	10.95	0.70
A_09_P037656	FBtr0307491	32073	CG1597	9.48	9.50	9.78	9.54	9.17	9.27	9.22	9.10	-0.39
A_09_P037671	FBtr0073452	32076	CG11752	14.09	14.01	14.15	14.14	13.45	13.75	13.82	13.98	-0.35
A_09_P037741	FBtr0073506	32095	Spase25	13.83	14.24	14.05	14.29	12.64	12.64	13.47	13.82	-0.96
A_09_P037786	FBtr0073518	32109	rho-4	10.27	10.34	10.04	10.16	10.75	10.58	10.80	10.83	0.54
A_09_P037816	FBtr0073531	32121	CG2444	10.43	10.39	10.40	10.45	14.39	13.79	13.57	12.76	3.21
A_09_P037821	FBtr0073620	32122	FucT6	8.89	9.21	9.19	9.17	8.45	8.44	8.42	8.60	-0.63
A_09_P037836	FBtr0073552	32125	Tango10	9.78	10.14	9.92	10.13	9.37	9.67	9.53	9.72	-0.42
A_09_P037876	FBtr0073604	32140	p24-1	12.10	12.26	12.34	12.41	11.25	11.35	11.63	11.81	-0.77
A_09_P037881	FBtr0073603	32141	CG10347	9.53	9.39	9.56	9.39	8.80	8.91	8.99	8.99	-0.54
A_09_P037891	FBtr0073601	32143	CG2025	11.51	11.39	11.38	11.40	10.60	10.33	10.86	11.12	-0.69
A_09_P037896	FBtr0073567	32144	CG1847	8.66	8.40	8.59	8.35	8.05	7.75	8.00	8.08	-0.53
A_09_P037906	FBtr0073569	32146	CG15739	10.12	9.86	9.88	9.91	12.05	11.77	11.39	10.76	1.55
A_09_P037926	FBtr0073571	32150	CG1840	11.02	10.87	11.09	10.84	9.82	9.99	10.45	10.79	-0.69
A_09_P038091	FBtr0073650	32198	Cpr11A	14.56	14.40	14.17	14.43	15.12	15.16	14.89	14.74	0.59
A_09_P038101	FBtr0073652	32201	CG2556	12.45	12.50	12.51	12.36	13.39	12.92	12.95	12.77	0.55
A_09_P038151	FBtr0073709	32215	Aven	8.99	8.67	8.61	8.67	8.17	7.84	8.34	8.48	-0.53
A_09_P038156	FBtr0073675	32216	CG2540	9.95	9.29	9.57	9.37	11.09	10.47	10.51	10.18	1.02
A_09_P038191	FBtr0073690	32234	CG3775	8.46	9.11	8.90	9.12	7.68	8.29	8.10	8.27	-0.81
A_09_P038271	FBtr0073721	32258	CG2200	8.50	8.65	8.47	8.74	8.23	8.23	8.21	8.30	-0.35
A_09_P038286	FBtr0073723	32262	CG15717	13.20	13.46	13.49	13.36	12.39	12.54	12.69	12.92	-0.74
A_09_P038301	FBtr0073726	32267	CG4332	9.87	10.17	10.05	10.15	8.87	8.86	9.21	9.38	-0.98
A_09_P038306	FBtr0073727	32269	CG12096	11.55	11.54	11.59	11.71	10.56	10.59	11.10	11.26	-0.72
A_09_P038331	FBtr0073756	2768909	CG15744	8.73	9.04	8.99	9.14	8.32	8.49	8.59	8.78	-0.43

A_09_P038376	FBtr0073775	32294	Tim9a	12.33	11.79	12.13	11.93	11.42	11.57	11.60	11.72	**-0.47**
A_09_P038381	FBtr0073776	32296	CG1662	11.13	11.46	11.45	11.40	10.77	10.94	10.63	10.56	**-0.64**
A_09_P038386	FBtr0073777	32297	CG1673	12.60	13.39	13.17	13.34	12.45	12.59	12.24	12.13	**-0.77**
A_09_P038401	FBtr0073787	32300	CG1998	9.46	10.22	10.02	10.36	9.02	8.82	9.06	9.06	**-1.03**
A_09_P038446	FBtr0073806	32317	BthD	9.12	9.66	9.42	9.75	8.97	9.03	8.83	8.91	**-0.56**
A_09_P038451	FBtr0073849	32318	tth	9.57	9.81	9.74	9.87	9.16	9.40	9.32	9.40	**-0.43**
A_09_P038476	FBtr0073809	32323	CG11164	8.70	8.56	8.57	8.59	7.84	7.96	8.09	8.46	**-0.52**
A_09_P038486	FBtr0073811	32325	CG11162	9.47	8.59	8.25	8.07	9.88	9.60	9.84	9.78	**1.18**
A_09_P038511	FBtr0073818	32334	CG11134	13.39	13.51	13.66	13.64	12.86	13.00	13.17	13.43	**-0.44**
A_09_P038516	FBtr0073819	32335	CG11151	14.79	14.60	14.60	14.59	14.35	14.44	14.39	14.32	**-0.27**
A_09_P038521	FBtr0073840	32337	Pdcd4	12.55	13.32	13.00	13.48	14.22	14.30	13.99	13.81	**0.99**
A_09_P038626	FBtr0073903	32377	CG1434	10.11	9.71	9.94	9.69	10.84	11.21	10.65	10.52	**0.94**
A_09_P038631	FBtr0073923	32378	Fbxl4	8.70	9.04	8.91	8.95	8.26	8.61	8.60	8.63	**-0.38**
A_09_P038651	FBtr0073920	32385	CG9400	6.83	5.59	5.81	5.52	6.79	7.02	7.14	7.40	**1.15**
A_09_P038691	FBtr0073944	32397	CG9413	10.23	10.46	10.11	10.52	9.88	9.87	9.83	9.46	**-0.57**
A_09_P038736	FBtr0073989	32410	CG14407	13.44	13.33	13.51	13.44	12.92	13.22	13.19	13.25	**-0.28**
A_09_P038776	FBtr0073979	32419	CG9512	13.88	14.03	13.96	13.87	13.38	13.46	13.49	13.65	**-0.44**
A_09_P038781	FBtr0073978	32420	CG9509	11.43	12.28	12.19	11.92	10.88	10.93	10.80	10.99	**-1.05**
A_09_P038821	FBtr0073960	32434	CG5548	14.61	14.31	14.47	14.59	13.86	14.15	14.17	14.16	**-0.41**
A_09_P038831	FBtr0073961	32436	dob	8.75	10.50	9.67	10.29	7.06	7.61	7.32	7.75	**-2.37**
A_09_P038836	FBtr0340428	32437	Lsd-2	13.28	13.63	13.77	13.74	13.16	13.20	13.04	12.96	**-0.51**
A_09_P038851	FBtr0073964	32441	CG5599	11.25	11.28	11.30	11.19	10.51	10.67	10.53	10.78	**-0.63**
A_09_P038866	FBtr0074028	32444	Cyp4s3	8.59	9.44	9.26	9.04	6.88	7.39	8.08	8.59	**-1.35**
A_09_P038871	FBtr0074027	32446	RpL37a	7.90	7.37	7.63	7.14	9.30	10.03	9.49	9.33	**2.03**
A_09_P038936	FBtr0074019	32462	CG9123	10.81	10.64	10.78	10.75	10.57	10.58	10.41	10.48	**-0.24**
A_09_P038946	FBtr0074011	32464	CG6227	10.18	10.15	10.15	10.23	9.68	9.76	9.79	9.98	**-0.37**
A_09_P038961	FBtr0074031	32472	CG11655	10.16	10.60	10.73	10.64	9.47	10.00	9.77	9.76	**-0.78**
A_09_P038971	FBtr0074032	32475	CG6299	12.59	12.48	12.58	12.51	13.03	13.07	12.92	12.80	**0.41**
A_09_P038986	FBtr0074036	32479	CG6308	10.34	10.45	10.50	10.46	9.72	9.97	10.15	10.28	**-0.41**
A_09_P039016	FBtr0074045	32488	CG7860	11.45	12.64	12.19	12.62	10.44	10.07	10.44	10.58	**-1.85**
A_09_P039031	FBtr0089702	32492	cerv	8.99	9.17	8.98	9.12	8.37	8.74	8.70	8.86	**-0.40**

A_09_P039091	FBtr0074060	32505	CG9240	10.80	10.99	11.01	11.03	10.66	10.60	10.60	10.64	-0.33
A_09_P039116	FBtr0074055	32509	CG15601	7.70	7.86	7.83	7.97	7.36	7.44	7.46	7.70	-0.35
A_09_P039136	FBtr0074202	32515	CG11679	12.21	12.08	12.15	12.14	11.39	11.44	11.71	11.85	-0.55
A_09_P039141	FBtr0074085	32516	CG8206	13.31	13.63	13.47	13.72	14.32	14.27	14.04	13.76	0.56
A_09_P039186	FBtr0074094	32530	mRpS30	13.80	13.30	13.61	13.35	12.93	12.86	13.11	13.16	-0.50
A_09_P039201	FBtr0074183	32534	PGRP-LE	11.63	11.82	11.74	11.81	12.19	12.10	11.93	11.90	0.28
A_09_P039246	FBtr0074125	32552	CG8909	7.59	7.28	7.46	7.22	7.70	7.79	7.99	7.91	0.46
A_09_P039266	FBtr0340310	32561	mmd	6.84	6.58	6.76	6.69	7.19	7.71	7.27	7.40	0.67
A_09_P039281	FBtr0074153	32563	CG9170	8.71	8.99	8.81	9.03	9.24	9.52	9.14	9.23	0.40
A_09_P039286	FBtr0074134	32564	CG8931	10.97	10.89	10.86	10.85	10.60	10.35	10.51	10.68	-0.36
A_09_P039291	FBtr0074152	32565	CG9172	14.05	14.06	14.15	14.09	13.70	13.53	13.81	14.00	-0.33
A_09_P039316	FBtr0074206	32572	dpr18	6.43	5.64	5.81	5.38	6.48	6.82	6.75	6.76	0.89
A_09_P039321	FBtr0074258	32573	Nipsnap	10.86	11.41	11.14	11.25	10.19	9.48	10.06	10.32	-1.16
A_09_P039361	FBtr0074240	32592	CG9914	13.97	14.20	14.08	14.11	12.97	12.82	13.16	13.36	-1.02
A_09_P039391	FBtr0333303	32599	CG9921	12.28	12.16	12.38	12.29	11.69	11.66	11.85	11.83	-0.52
A_09_P039396	FBtr0074308	32605	CG9992	9.08	9.12	8.94	9.08	9.40	9.23	9.29	9.22	0.23
A_09_P039481	FBtr0074376	32639	CG9723	10.31	10.75	10.66	10.58	10.14	10.09	10.12	10.10	-0.46
A_09_P039506	FBtr0074364	32647	CG9676	7.14	5.32	5.82	5.54	8.69	8.27	8.03	7.30	2.12
A_09_P039511	FBtr0074363	32648	spheroide	9.51	8.93	9.88	9.16	10.85	10.78	10.27	10.05	1.12
A_09_P039546	FBtr0074330	32662	mRpL22	10.86	10.68	10.95	10.43	10.25	9.97	10.21	10.46	-0.51
A_09_P039626	FBtr0074347	32679	CG9099	13.76	13.93	13.84	13.95	13.12	13.09	13.42	13.70	-0.54
A_09_P039631	FBtr0074342	32680	CG4880	7.67	7.63	7.95	7.63	6.88	6.89	7.20	7.04	-0.72
A_09_P039646	FBtr0074417	32684	CG13001	6.69	6.82	6.73	6.76	5.95	6.10	6.12	6.51	-0.58
A_09_P039656	FBtr0110990	32686	RhoGAP15B	8.49	8.81	8.72	8.71	8.93	9.22	9.03	9.04	0.37
A_09_P039716	FBtr0074387	32701	CG5010	12.56	12.40	12.43	12.56	11.37	11.77	11.95	11.99	-0.72
A_09_P039786	FBtr0339779	32719	CG8664	13.55	14.57	13.87	14.35	12.39	11.65	12.18	11.91	-2.05
A_09_P039856	FBtr0074452	32740	CG5703	15.05	14.95	15.02	14.96	14.67	14.62	14.81	14.93	-0.24
A_09_P039866	FBtr0074453	32742	CG5800	11.55	11.10	11.40	11.19	11.65	11.98	11.74	11.80	0.48
A_09_P039906	FBtr0074462	32761	CG6398	10.69	10.35	10.16	10.32	11.32	11.46	11.33	11.40	1.00
A_09_P039911	FBtr0074472	32763	CG8142	10.68	10.55	10.69	10.64	10.11	10.09	10.28	10.41	-0.42
A_09_P039926	FBtr0074465	32766	CG6506	9.63	9.78	9.78	9.68	9.39	9.44	9.42	9.46	-0.29

A_09_P039941	FBtr0074493	32770	CG6769	11.27	10.87	11.04	10.79	11.62	11.65	11.55	11.52	0.59
A_09_P039986	FBtr0089574	32782	CG6867	11.01	11.16	11.28	11.01	10.79	10.49	10.58	10.61	-0.49
A_09_P040011	FBtr0343310	32790	RhoGAP16F	9.98	9.86	9.94	9.82	10.74	11.06	10.76	10.31	0.82
A_09_P040071	FBtr0074553	32816	CG6106	8.88	9.25	9.43	9.28	7.00	7.13	7.53	7.56	-1.90
A_09_P040106	FBtr0074628	32833	CG15046	6.12	5.82	5.54	5.45	7.66	7.33	7.03	6.51	1.40
A_09_P040111	FBtr0074627	32834	CG15044	11.19	10.80	10.77	10.75	11.30	11.45	11.37	11.38	0.50
A_09_P040116	FBtr0074626	32835	CG15043	13.55	13.75	13.70	13.62	15.97	15.32	15.75	15.52	1.99
A_09_P040136	FBtr0074622	32839	Ggt-1	11.11	10.91	11.19	10.99	11.93	11.69	11.67	11.62	0.67
A_09_P040176	FBtr0074601	32851	CG6540	11.30	11.30	11.16	11.18	10.70	10.80	10.81	10.90	-0.43
A_09_P040181	FBtr0074615	32852	CG6617	11.95	12.33	12.14	12.36	11.76	11.79	11.83	11.83	-0.39
A_09_P040231	FBtr0074661	32866	CG18259	8.72	8.82	8.86	8.66	8.31	8.30	8.48	8.44	-0.38
A_09_P040241	FBtr0074660	32869	CG6961	9.96	9.89	9.93	9.88	9.64	9.48	9.67	9.74	-0.28
A_09_P040246	FBtr0074635	32871	CG7053	9.42	9.08	9.22	9.05	10.62	10.33	9.98	9.62	0.95
A_09_P040261	FBtr0074639	32875	CG7101	8.07	8.25	8.21	8.29	7.43	7.56	7.72	7.88	-0.56
A_09_P040281	FBtr0074643	32881	CG7288	11.10	11.08	11.07	11.13	10.84	10.68	10.76	10.91	-0.30
A_09_P040346	FBtr0299624	32898	RhoGAP18B	8.31	8.74	8.43	8.66	9.60	9.68	9.10	8.96	0.79
A_09_P040366	FBtr0074666	32903	CG7453	11.27	11.55	11.53	11.65	10.67	10.69	10.83	10.73	-0.77
A_09_P040376	FBtr0074669	32905	Mec2	11.75	11.71	11.61	11.54	13.13	13.09	12.65	12.29	1.14
A_09_P040381	FBtr0074670	32906	HP1D3csd	5.79	5.39	4.80	4.70	7.39	7.55	6.90	6.50	1.91
A_09_P040391	FBtr0074671	32908	CG14194	10.93	11.25	11.16	11.25	10.84	10.97	10.84	10.78	-0.29
A_09_P040396	FBtr0308082	32909	CG7990	7.67	7.16	7.22	7.16	8.12	7.86	7.90	7.79	0.62
A_09_P040411	NM_133137	32913	Muc18B	13.00	13.06	13.00	13.06	14.62	14.26	14.14	14.02	1.23
A_09_P040451	FBtr0332394	32923	CG8028	8.97	9.21	9.64	9.34	7.95	8.67	8.65	8.75	-0.79
A_09_P040486	FBtr0074712	32936	CG12203	13.48	13.48	13.61	13.58	13.06	12.99	13.15	13.21	-0.43
A_09_P040521	FBtr0074769	32951	CG14219	10.06	8.68	8.26	8.49	12.66	11.98	12.00	11.65	3.20
A_09_P040541	FBtr0074734	32955	CG14207	15.14	15.38	15.35	15.25	15.04	15.04	14.73	14.48	-0.46
A_09_P040566	FBtr0074741	32963	MKP-4	9.00	9.00	9.22	8.86	9.49	9.81	9.44	9.34	0.50
A_09_P040586	FBtr0074745	32968	Sec61gamma	11.29	11.73	11.74	11.62	10.56	10.45	10.90	11.22	-0.81
A_09_P040596	FBtr0074759	32970	Ranbp21	10.13	10.21	10.22	10.18	9.72	9.71	9.79	9.97	-0.38
A_09_P040616	FBtr0308570	32975	et	9.32	10.01	9.89	9.83	8.52	9.16	9.05	9.28	-0.76
A_09_P040621	FBtr0074750	32977	Ubqn	12.97	12.95	13.03	13.10	12.76	12.75	12.75	12.78	-0.25

A_09_P040656	FBtr0074790	32988	CG14234	8.39	8.42	8.32	8.17	8.83	8.77	8.69	8.96	0.49
A_09_P040661	FBtr0074815	32989	CoVIb	15.11	14.99	15.25	15.17	14.69	14.73	14.99	14.93	-0.30
A_09_P040711	FBtr0070000	33005	Nep3	7.21	7.64	7.09	7.16	8.55	8.46	7.92	7.87	0.93
A_09_P040746	FBtr0070027	33015	Phf7	9.13	9.18	9.11	9.23	8.70	8.23	8.72	8.93	-0.52
A_09_P040756	FBtr0070026	33017	CG9581	12.62	12.65	12.75	12.67	12.49	12.55	12.46	12.55	-0.16
A_09_P040761	FBtr0070008	33018	CG9578	9.89	9.95	9.95	10.06	9.47	9.24	9.57	9.74	-0.46
A_09_P040801	FBtr0077330	33034	Syx16	12.17	12.48	12.37	12.49	12.60	12.79	12.64	12.62	0.28
A_09_P040846	FBtr0077310	33047	GstT3	10.60	10.82	10.72	10.76	10.32	10.19	10.16	10.21	-0.51
A_09_P040946	FBtr0077268	33076	CG1532	14.08	14.25	14.08	14.09	12.82	12.80	13.37	13.71	-0.95
A_09_P040956	FBtr0077241	33078	Ntf-2	13.45	13.64	13.49	13.68	12.75	13.24	13.11	13.19	-0.49
A_09_P040961	FBtr0113007	33078	Ntf-2	12.37	12.46	12.54	12.53	11.55	11.62	11.76	11.95	-0.75
A_09_P040976	FBtr0077264	33082	CG1518	12.35	12.80	12.36	12.70	11.52	11.49	11.96	12.29	-0.74
A_09_P041011	FBtr0077257	33097	CG1724	6.47	6.97	6.29	6.78	5.89	5.00	5.50	5.49	-1.16
A_09_P041046	FBtr0077218	33123	CG10918	13.21	14.45	14.26	14.88	15.00	15.40	15.18	15.18	0.99
A_09_P041106	FBtr0085609	48445	Acph-1	10.85	10.95	10.90	10.95	10.61	10.26	10.40	10.43	-0.49
A_09_P041116	FBtr0072368	37892	mAcR-60C	8.03	7.91	7.85	7.81	8.32	8.57	8.54	8.70	0.64
A_09_P041121	FBtr0073299	38545	nAcRbeta-64B	7.44	6.82	7.31	6.96	8.10	8.54	8.10	7.99	1.05
A_09_P041126	FBtr0073300	38545	nAcRbeta-64B	6.70	6.15	5.85	5.89	6.97	7.84	7.36	7.40	1.25
A_09_P041136	FBtr0100662	31521	Act5C	15.60	15.38	15.51	15.33	15.90	15.86	15.79	15.78	0.38
A_09_P041156	FBtr0082786	48632	Act87E	15.46	15.96	16.04	16.03	15.00	14.71	14.85	14.70	-1.06
A_09_P041176	FBtr0100353	33986	ade3	11.37	11.43	11.37	11.37	10.66	11.06	11.00	11.20	-0.41
A_09_P041186	FBtr0100591	3771877	Adh	9.83	10.19	9.87	10.03	9.41	9.19	9.21	9.26	-0.71
A_09_P041193	FBtr0100594	3771877	Adh	16.75	16.73	16.68	16.79	16.02	16.24	16.51	16.60	-0.40
A_09_P041196	FBtr0086744	37073	adp	8.55	9.11	8.94	8.89	8.09	8.17	8.14	8.41	-0.67
A_09_P041241	FBtr0086983	36932	Amy-d	12.49	13.71	12.05	12.74	14.73	14.26	14.20	13.64	1.46
A_09_P041246	FBtr0087004	47764	Amy-p	12.21	12.84	11.58	12.16	14.52	13.84	13.78	13.15	1.63
A_09_P041251	FBtr0089519	42492	AnnIX	14.58	14.76	14.75	14.79	14.89	15.03	14.92	14.94	0.23
A_09_P041291	FBtr0077850	33392	aop	7.31	6.65	6.32	6.48	8.19	7.92	7.83	7.55	1.18
A_09_P041296	FBtr0086058	35509	ap	8.63	9.37	8.94	9.27	8.23	8.27	8.33	8.29	-0.77
A_09_P041306	FBtr0070109	31002	Appl	9.86	9.99	9.65	9.97	10.52	10.58	10.46	10.54	0.65
A_09_P041311	FBtr0089420	48224	Aprt	11.53	11.35	11.38	11.38	10.89	10.85	11.00	11.12	-0.45

A_09_P041326	FBtr0076543	39041	Argk	15.97	16.09	15.88	16.01	15.63	15.59	15.74	15.88	-0.28
A_09_P041336	FBtr0087702	44279	arr	8.44	8.81	8.70	8.64	8.16	8.41	8.10	7.92	-0.50
A_09_P041341	FBtr0081030	35078	Arr1	9.01	8.57	8.75	8.59	9.19	9.62	9.57	9.81	0.82
A_09_P041356	FBtr0084660	42936	ash2	10.17	10.19	10.24	10.23	11.00	11.00	10.77	10.63	0.65
A_09_P041361	FBtr0084692	42946	asp	8.68	9.48	9.28	9.59	8.17	8.09	8.07	7.88	-1.21
A_09_P041391	FBtr0074372	32645	Axs	11.09	11.63	11.43	11.62	10.94	11.12	10.91	10.91	-0.47
A_09_P041416	FBtr0302291	37044	proPO-A1	12.82	13.59	13.78	13.93	11.57	11.36	11.89	12.20	-1.77
A_09_P041426	FBtr0334319	45826	bel	10.63	10.62	10.82	10.45	10.19	10.05	9.75	9.87	-0.66
A_09_P041491	FBtr0075526	39744	brm	11.56	11.91	11.78	11.93	11.06	11.08	11.35	11.55	-0.53
A_09_P041496	FBtr0075621	39620	Brd	6.37	7.49	6.97	7.85	4.90	5.35	4.99	4.83	-2.15
A_09_P041541	FBtr0082119	41144	by	6.17	5.18	5.02	4.96	7.12	7.33	6.54	6.28	1.49
A_09_P041556	FBtr0080847	34969	cact	11.50	11.43	11.42	11.30	12.00	11.75	11.64	11.69	0.36
A_09_P041596	FBtr0075058	40048	Cat	15.11	15.53	15.61	15.54	14.43	14.41	14.64	14.70	-0.90
A_09_P041611	FBtr0299892	40305	Pka-R1	10.98	11.19	11.29	11.19	10.27	10.17	10.25	10.27	-0.92
A_09_P041616	FBtr0299891	40305	Pka-R1	12.41	12.73	12.75	12.87	11.49	11.41	11.68	11.95	-1.05
A_09_P041626	FBtr0085614	43597	CecA2	4.10	6.22	3.92	4.41	13.29	11.80	12.30	11.49	7.56
A_09_P041676	FBtr0089368	42249	Cha	8.03	7.86	7.84	7.32	9.13	9.41	8.83	9.22	1.39
A_09_P041701	FBtr0070065	30973	cin	11.10	11.12	11.06	11.10	10.54	10.44	10.77	10.95	-0.42
A_09_P041746	FBtr0080849	34967	cni	11.39	11.51	11.38	11.48	11.29	11.03	11.27	11.28	-0.22
A_09_P041751	FBtr0334913	40620	cno	7.59	7.50	7.58	7.43	8.89	8.78	8.37	7.95	0.97
A_09_P041766	FBtr0089015	35653	cos	7.60	7.58	7.51	7.66	6.85	6.81	6.86	7.10	-0.68
A_09_P041801	FBtr0330275	45840	cpo	9.48	9.43	9.40	9.21	10.47	10.46	10.22	10.21	0.96
A_09_P041821	FBtr0081512	47767	crc	15.04	15.15	15.18	15.07	15.41	15.33	15.34	15.33	0.24
A_09_P041836	FBtr0070495	31268	crm	7.23	7.74	7.35	7.41	7.78	8.00	7.87	7.88	0.45
A_09_P041851	FBtr0111126	3355131	cta	8.26	8.23	8.33	8.23	7.77	7.60	7.81	7.72	-0.53
A_09_P041856	FBtr0112506	5740536	CG34310	13.26	12.91	13.16	13.09	12.64	12.80	12.78	12.90	-0.32
A_09_P041866	FBtr0071610	45280	cv-2	5.48	5.38	4.92	4.82	6.38	6.80	6.41	6.47	1.37
A_09_P041871	FBtr0076075	39340	CycA	9.42	9.79	9.77	9.98	9.03	8.83	8.87	8.92	-0.83
A_09_P041876	FBtr0071911	37618	CycB	6.99	7.66	7.56	7.64	5.82	5.86	5.80	5.91	-1.61
A_09_P041901	FBtr0075746	39570	D	7.76	7.77	7.78	7.67	8.51	8.92	8.19	8.33	0.74
A_09_P041906	FBtr0081987	41095	D1	12.63	12.98	13.02	13.12	12.43	12.36	12.50	12.24	-0.56

A_09_P041946	FBtr0078042	33253	Pkg21D	10.84	11.08	10.88	11.02	10.16	10.36	10.38	10.62	-0.57
A_09_P041981	FBtr0082597	47769	Dip-C	12.31	12.41	12.19	12.36	13.30	13.06	12.94	12.57	0.65
A_09_P041991	FBtr0081006	35047	dl	9.72	9.66	9.38	9.67	11.33	11.59	10.79	10.28	1.39
A_09_P042016	FBtr0070525	31310	dm	8.87	8.78	8.86	8.67	10.08	10.53	9.72	9.56	1.18
A_09_P042026	FBtr0083538	48228	DNaseII	13.83	13.94	13.88	13.83	12.84	12.76	13.35	13.74	-0.70
A_09_P042046	FBtr0302290	44513	proPO59	7.70	5.91	8.03	5.63	8.50	9.61	9.05	9.02	2.22
A_09_P042076	FBtr0078989	45845	Dsk	7.86	7.23	7.51	7.49	8.21	8.31	8.20	8.28	0.73
A_09_P042081	FBtr0081759	40940	dsx	6.71	7.43	6.95	7.25	4.30	4.51	4.77	4.59	-2.54
A_09_P042096	FBtr0070914	31589	dx	8.86	9.35	9.14	9.27	8.32	8.28	8.45	8.48	-0.77
A_09_P042121	FBtr0074214	32585	eas	11.60	11.12	11.49	11.08	12.60	12.13	12.11	11.88	0.86
A_09_P042126	FBtr0072521	44811	E(bx)	8.18	8.13	8.22	8.20	7.99	8.01	7.89	7.77	-0.26
A_09_P042146	FBtr0086010	35540	EcR	8.60	9.74	9.02	9.26	7.49	8.01	7.93	8.28	-1.23
A_09_P042201	FBtr0086710	37143	Eip55E	13.84	13.99	13.97	14.00	13.46	13.52	13.48	13.63	-0.42
A_09_P042206	FBtr0075202	39962	Eip74EF	11.41	11.69	11.70	11.46	10.23	10.23	10.35	10.59	-1.21
A_09_P042216	FBtr0075148	39999	Eip75B	10.16	10.08	9.99	9.68	10.80	11.23	10.39	10.94	0.86
A_09_P042261	FBtr0080325	34595	esc	9.69	9.77	9.74	9.71	9.25	8.95	9.38	9.58	-0.44
A_09_P042286	FBtr0074135	32567	exd	11.46	11.69	11.75	11.73	11.32	11.31	11.23	11.12	-0.41
A_09_P042291	FBtr0086242	37345	exu	9.26	8.90	9.15	8.96	8.65	8.44	8.63	8.70	-0.46
A_09_P042301	FBtr0074463	32762	e(y)1	11.15	11.24	11.34	11.31	10.82	10.96	10.89	11.03	-0.33
A_09_P042316	FBtr0076279	39203	E(z)	11.04	11.14	11.14	11.24	11.02	10.93	11.00	10.89	-0.18
A_09_P042326	FBtr0087625	36521	fas	9.13	9.22	9.17	9.42	9.71	9.96	9.68	9.63	0.51
A_09_P042386	FBtr0070345	31166	Actn	14.15	14.50	14.72	14.62	13.20	13.09	13.37	13.49	-1.21
A_09_P042416	FBtr0082052	41118	Fps85D	9.70	9.68	9.30	9.56	10.00	10.15	10.01	9.97	0.47
A_09_P042451	FBtr0305585	35336	Fs(2)Ket	9.94	10.18	9.90	9.75	9.42	9.57	9.19	9.55	-0.51
A_09_P042456	FBtr0087387	47121	dup	9.86	9.87	9.92	9.94	9.20	9.25	9.28	9.28	-0.65
A_09_P042476	FBtr0074602	32855	fu	8.32	8.34	8.34	8.36	7.76	7.91	7.96	7.99	-0.44
A_09_P042536	FBtr0091757	3772583	gdl	8.99	8.90	9.17	9.18	8.40	7.93	8.15	8.20	-0.89
A_09_P042541	FBtr0076934	38765	G-ialpha65A	11.32	11.49	11.25	11.40	12.03	11.81	11.76	11.66	0.45
A_09_P042576	FBtr0088271	36104	G-oalpha47A	10.60	10.72	10.67	10.51	11.44	11.18	10.91	11.04	0.52
A_09_P042581	FBtr0072143	37805	G-salpha60A	12.11	12.46	12.34	12.33	11.74	11.36	11.72	11.82	-0.65
A_09_P042586	FBtr0087232	36782	Got1	14.08	14.43	14.32	14.56	13.38	13.21	13.59	13.90	-0.83

A_09_P042591	FBtr0077867	33373	Got2	15.51	15.54	15.54	15.51	15.19	15.16	15.23	15.29	-0.31
A_09_P042596	FBtr0079147	33824	Gpdh	13.17	12.93	12.88	12.88	12.17	11.91	12.20	12.30	-0.82
A_09_P042611	FBtr0079708	34171	grk	10.46	10.60	10.57	10.54	9.75	9.44	9.43	9.26	-1.07
A_09_P042616	FBtr0081808	40962	grn	7.94	7.88	7.98	7.44	9.27	8.93	8.68	8.46	1.03
A_09_P042656	FBtr0100153	38995	h	12.23	11.84	12.34	12.04	13.39	13.37	12.92	12.44	0.92
A_09_P042731	FBtr0084451	42803	Hmgcr	12.01	11.31	11.42	11.12	12.47	12.33	12.55	12.69	1.04
A_09_P042746	FBtr0085298	43385	Hrb98DE	11.89	11.86	11.70	11.93	11.62	11.29	11.56	11.66	-0.31
A_09_P042776	FBtr0087568	36583	Hsc70-5	14.04	13.60	13.86	13.65	13.39	13.35	13.43	13.43	-0.39
A_09_P042816	FBtr0076497	39071	Hsp67Bc	10.34	11.26	10.73	11.10	12.30	12.01	11.85	11.56	1.07
A_09_P042856	FBtr0074357	32661	if	11.78	12.00	11.95	12.07	11.51	11.73	11.30	10.97	-0.57
A_09_P042886	FBtr0073249	38513	ImpL2	12.73	12.75	12.64	12.58	14.47	14.22	13.97	14.06	1.50
A_09_P042891	FBtr0077008	45880	ImpL3	14.71	15.19	15.10	15.29	12.15	12.51	13.88	14.60	-1.79
A_09_P042916	FBtr0085595	43569	janA	10.23	10.12	10.21	10.24	9.61	9.38	9.66	9.69	-0.61
A_09_P042926	FBtr0088704	35853	Jon44E	14.92	13.95	13.83	13.90	15.68	15.93	15.55	15.15	1.43
A_09_P042931	FBtr0088410	36057	Jra	7.20	7.26	7.07	6.87	8.49	8.40	7.89	7.63	1.01
A_09_P042951	FBtr0087184	36810	Khc	12.84	12.87	12.93	12.87	12.71	12.53	12.59	12.57	-0.28
A_09_P042991	FBtr0112809	45318	kn	6.80	6.27	6.36	6.56	7.52	7.42	7.10	6.94	0.75
A_09_P043016	FBtr0072449	38012	Kr	6.16	7.29	6.79	7.40	3.37	3.64	3.28	3.02	-3.58
A_09_P043056	FBtr0073460	32069	l(1)10Bb	11.98	12.04	12.10	12.06	11.68	11.79	11.82	11.96	-0.23
A_09_P043066	FBtr0077221	33118	Hlc	12.36	12.30	12.38	12.40	13.39	13.80	13.40	13.03	1.05
A_09_P043071	FBtr0073841	48481	l(1)dd4	8.13	8.70	8.29	8.44	7.82	7.62	7.70	7.62	-0.70
A_09_P043086	FBtr0073488	32083	dlg1	9.80	10.19	10.19	10.05	9.64	9.87	9.27	9.42	-0.51
A_09_P043091	FBtr0079179	33836	ifc	12.16	12.23	12.10	12.31	13.08	13.07	12.66	12.47	0.62
A_09_P043096	FBtr0079177	33835	eIF-4a	15.85	15.77	15.73	15.81	15.99	15.97	15.94	15.96	0.18
A_09_P043106	FBtr0080541	34790	Sos	10.70	10.98	11.03	11.24	10.46	10.57	10.60	10.53	-0.45
A_09_P043131	FBtr0080705	34888	stc	12.41	12.47	12.51	12.59	12.68	13.00	12.78	12.82	0.33
A_09_P043161	FBtr0080771	34925	l(2)35Di	8.88	8.81	8.66	8.75	7.93	7.56	7.91	8.20	-0.88
A_09_P043166	FBtr0080764	48785	wek	8.15	8.04	8.11	7.93	7.61	7.84	7.83	7.59	-0.34
A_09_P043196	FBtr0081143	48805	Catsup	12.59	13.02	12.74	12.84	11.98	11.82	12.28	12.55	-0.64
A_09_P043211	FBtr0081155	35189	CG10561	10.07	10.19	10.36	10.40	9.49	9.54	9.56	9.56	-0.72
A_09_P043216	FBtr0081244	35235	swm	11.82	12.07	12.22	12.04	11.65	11.67	11.62	11.76	-0.36

A_09_P043246	FBtr0075360	39855	Pros26	13.73	13.87	13.69	13.78	13.05	13.12	13.36	13.65	-0.48
A_09_P043321	FBtr0088763	35817	Lcp1	14.80	16.01	15.60	16.07	12.12	12.17	11.42	10.43	-4.08
A_09_P043326	FBtr0088761	35818	Lcp2	16.57	16.76	16.56	16.74	14.77	15.54	14.43	12.98	-2.23
A_09_P043336	FBtr0088744	35820	Lcp4	13.68	14.87	14.45	14.80	12.66	13.52	12.25	10.80	-2.14
A_09_P043341	FBtr0081758	45894	lds	8.31	8.50	8.55	8.70	8.22	7.97	8.07	8.03	-0.44
A_09_P043366	FBtr0078025	33274	Lsp1beta	15.59	15.85	15.48	15.67	5.00	7.82	8.12	8.74	-8.23
A_09_P043371	FBtr0072463	38015	Lsp1gamma	16.52	16.64	16.56	16.59	9.54	10.54	9.23	8.38	-7.16
A_09_P043376	FBtr0089324	45326	Lsp2	15.41	16.05	15.84	15.90	2.89	5.13	4.40	3.97	-11.70
A_09_P043386	FBtr0089931	35940	ltd	11.74	11.60	11.52	11.54	12.06	12.07	11.84	11.81	0.35
A_09_P043391	FBtr0088747	35825	Mal-A2	6.77	5.80	6.19	5.64	7.91	7.48	7.47	6.96	1.35
A_09_P043441	FBtr0078056	33214	RpLP1	16.13	16.13	16.10	16.20	16.20	16.23	16.21	16.24	0.08
A_09_P043461	FBtr0085593	43573	RpL32	16.35	16.28	16.39	16.38	16.42	16.44	16.47	16.43	0.09
A_09_P043496	FBtr0339956	36555	mam	8.06	8.38	7.95	8.10	8.84	8.97	8.75	8.78	0.71
A_09_P043506	FBtr0085876	43765	Map205	8.99	9.40	9.44	9.28	8.49	8.41	8.08	7.96	-1.04
A_09_P043541	FBtr0082660	47173	Men	12.98	13.16	13.01	12.98	12.10	12.27	12.46	12.84	-0.62
A_09_P043556	FBtr0084982	43152	E(spl)mbeta-HLH	11.32	11.29	11.40	11.39	12.12	12.24	12.17	12.07	0.80
A_09_P043571	FBtr0071569	37402	mago	10.43	10.38	10.33	10.15	9.51	9.19	9.56	9.94	-0.77
A_09_P043601	FBtr0300508	32771	mnb	7.93	7.94	7.59	7.88	8.59	8.54	8.58	8.48	0.71
A_09_P043611	FBtr0075627	39625	mnd	10.61	10.02	10.41	10.05	8.76	9.17	9.45	9.78	-0.98
A_09_P043621	FBtr0084061	49228	mod(mdg4)	7.93	8.09	8.07	8.05	7.22	7.53	7.58	7.54	-0.57
A_09_P043631	FBtr0084065	49228	mod(mdg4)	7.14	7.31	7.14	7.24	6.44	6.84	6.84	6.69	-0.51
A_09_P043646	FBtr0084072	49228	mod(mdg4)	7.24	7.41	7.34	7.32	6.58	6.97	6.87	7.12	-0.44
A_09_P043651	FBtr0084073	49228	mod(mdg4)	9.74	10.36	10.15	10.32	9.47	9.45	9.51	9.73	-0.60
A_09_P043656	FBtr0084074	49228	mod(mdg4)	8.99	9.54	9.22	9.44	8.36	8.62	8.74	8.97	-0.63
A_09_P043661	FBtr0084079	49228	mod(mdg4)	10.28	10.73	10.62	10.62	9.85	9.72	9.88	10.06	-0.68
A_09_P043676	FBtr0072290	37894	Mov34	14.04	14.26	14.15	14.13	13.61	13.57	13.88	13.98	-0.39
A_09_P043681	FBtr0087745	36468	Mp20	16.08	16.14	16.06	16.13	15.83	15.60	15.85	15.84	-0.32
A_09_P043756	FBtr0073817	48309	mus101	7.41	7.87	7.78	7.51	6.63	7.09	7.11	7.12	-0.66
A_09_P043761	FBtr0100473	3772069	mus201	8.81	8.73	8.64	8.76	9.04	9.23	9.24	9.49	0.52
A_09_P043766	FBtr0088831	47186	mus205	7.65	7.86	7.45	7.84	8.54	8.76	8.36	8.34	0.80
A_09_P043771	FBtr0076689	38905	mus301	8.39	8.54	8.45	8.28	7.59	7.87	7.79	7.87	-0.64

A_09_P043776	FBtr0075122	40003	mus304	7.84	8.09	8.22	8.13	7.54	7.54	7.71	7.88	-0.40
A_09_P043781	FBtr0082662	41571	mus308	7.56	7.51	7.65	7.85	7.22	7.19	7.37	7.28	-0.38
A_09_P043806	FBtr0089512	48971	Atpalpha	11.38	11.19	11.12	10.79	12.27	12.42	11.86	12.18	1.07
A_09_P043811	FBtr0089513	48971	Atpalpha	12.19	12.39	12.42	12.48	11.64	11.53	11.71	11.69	-0.73
A_09_P043816	FBtr0084472	42799	nau	8.65	8.56	8.56	8.50	7.29	7.12	7.63	7.87	-1.09
A_09_P043846	FBtr0070523	31300	ng1	14.86	15.19	15.12	14.64	15.35	15.59	15.65	15.62	0.60
A_09_P043871	FBtr0081031	326160	ninaD	13.50	13.89	13.28	13.55	10.96	11.70	10.88	10.56	-2.53
A_09_P043986	FBtr0074298	32619	para	6.53	5.71	5.98	5.72	7.21	7.68	7.06	7.25	1.32
A_09_P043991	FBtr0076772	38879	pbl	8.98	9.31	9.42	9.28	8.66	8.64	8.47	8.16	-0.76
A_09_P044016	FBtr0070680	31391	peb	11.16	11.26	11.23	11.07	12.53	12.61	12.13	12.13	1.17
A_09_P044051	FBtr0088420	36060	Pfk	13.95	14.35	14.28	14.42	12.37	12.41	13.17	13.53	-1.38
A_09_P044056	FBtr0088679	35886	Pgi	15.73	15.90	15.77	15.85	14.77	14.73	15.29	15.62	-0.71
A_09_P044061	FBtr0077739	33461	Pgk	15.22	15.31	15.36	15.41	14.12	14.39	14.83	15.15	-0.70
A_09_P044081	FBtr0070537	31303	Pig1	11.93	12.44	12.23	12.43	11.73	11.88	11.59	11.44	-0.60
A_09_P044146	FBtr0087049	48311	Pkc53E	8.05	7.48	7.80	7.53	8.26	8.45	8.20	8.30	0.59
A_09_P044151	FBtr0085359	43428	Pkc98E	9.85	10.03	9.86	10.09	10.41	10.47	10.27	10.12	0.36
A_09_P044161	FBtr0070389	31194	pn	8.12	8.76	8.16	8.56	7.64	7.83	7.64	7.75	-0.69
A_09_P044206	FBtr0072719	38160	Ptp61F	9.61	10.08	9.70	9.84	10.56	10.39	10.15	10.14	0.51
A_09_P044211	FBtr0070921	31582	PpV	11.34	11.44	11.51	11.40	11.30	11.21	11.19	11.10	-0.22
A_09_P044231	FBtr0076593	39002	Prm	16.21	16.35	16.26	16.28	15.76	15.65	15.95	15.98	-0.44
A_09_P044266	FBtr0082977	41772	put	9.87	10.15	9.87	10.00	9.58	9.57	9.45	9.56	-0.43
A_09_P044306	FBtr0084214	42620	PyK	15.70	15.73	15.91	15.85	14.54	15.32	15.07	15.40	-0.71
A_09_P044356	FBtr0079746	44851	raw	8.55	8.21	8.13	7.86	10.31	10.43	9.65	8.97	1.65
A_09_P044361	FBtr0070702	31381	rb	8.85	9.11	9.14	9.23	8.35	8.37	8.60	8.53	-0.62
A_09_P044376	FBtr0081253	35246	ref(2)P	13.03	13.50	13.29	13.54	14.85	14.71	14.35	13.86	1.10
A_09_P044396	FBtr0075338	39887	Rh4	4.85	4.18	3.34	3.82	5.55	5.66	6.16	6.53	1.93
A_09_P044406	FBtr0080610	34819	rk	6.51	5.14	5.49	4.44	8.32	8.11	7.51	7.12	2.37
A_09_P044456	FBtr0087105	36855	RpLP2	16.00	16.00	15.91	16.01	16.12	16.11	16.13	16.13	0.14
A_09_P044466	FBtr0082892	41721	RpII140	11.90	12.08	11.78	12.06	11.15	11.11	11.41	11.60	-0.64
A_09_P044496	FBtr0072681	44856	ru	6.78	6.92	6.84	6.59	6.31	6.29	6.23	5.97	-0.58
A_09_P044516	FBtr0082704	41605	ry	10.52	10.94	10.90	10.75	9.83	9.98	10.19	10.59	-0.63

A_09_P044571	FBtr0082102	41168	Scm	9.06	9.05	9.24	9.11	8.82	8.96	8.93	8.88	-0.22
A_09_P044586	FBtr0081234	35223	RanGap	12.54	12.55	12.59	12.60	11.97	11.67	12.21	12.42	-0.50
A_09_P044621	FBtr0073393	32039	sev	8.30	8.76	8.30	8.38	6.98	7.50	7.80	8.11	-0.83
A_09_P044671	FBtr0089658	32780	Sh	8.76	8.58	8.58	8.49	9.33	9.66	9.18	9.23	0.75
A_09_P044676	FBtr0089663	32780	Sh	8.53	8.54	8.39	8.55	9.20	9.55	9.04	8.98	0.69
A_09_P044706	FBtr0070978	31617	shf	10.39	10.59	10.29	10.27	10.96	11.06	10.86	10.77	0.53
A_09_P044746	FBtr0112830	43906	skd	8.12	8.21	7.96	8.13	8.72	8.98	8.60	8.39	0.57
A_09_P044756	FBtr0077210	33117	slgA	12.35	12.59	12.35	12.65	11.80	11.58	11.96	12.06	-0.63
A_09_P044776	FBtr0072848	44013	sls	12.70	13.21	13.24	13.33	11.73	11.62	11.79	11.85	-1.37
A_09_P044781	FBtr0332674	44013	sls	11.06	11.73	11.49	11.73	10.66	10.71	10.61	10.79	-0.81
A_09_P044791	FBtr0078129	33196	smo	9.71	10.05	9.79	9.80	9.66	9.06	9.20	9.30	-0.53
A_09_P044806	FBtr0070748	31442	snf	11.76	11.73	11.62	11.85	11.36	11.25	11.47	11.64	-0.31
A_09_P044821	FBtr0088970	35662	so	9.35	9.54	9.47	9.51	9.25	9.29	9.06	8.98	-0.32
A_09_P044826	Y00367	39251	Sod	14.76	14.90	14.81	14.93	14.29	14.38	14.49	14.68	-0.39
A_09_P044836	FBtr0089678	44014	sol	11.62	11.89	11.76	12.02	12.80	12.94	12.54	12.24	0.81
A_09_P044866	FBtr0082933	41746	spn-B	7.42	7.19	7.45	7.15	6.38	6.66	6.64	6.78	-0.69
A_09_P044886	FBtr0085137	43256	spz	11.27	11.35	11.40	11.44	11.64	11.83	11.62	11.67	0.33
A_09_P044926	FBtr0088001	36329	Cam	14.30	14.36	14.24	14.03	14.82	14.80	14.37	14.75	0.45
A_09_P044936	FBtr0086553	37228	SdhA	9.76	10.02	9.75	9.71	9.22	8.61	8.94	9.13	-0.84
A_09_P044941	FBtr0303096	38447	Scsalpha	8.51	8.67	8.40	8.39	7.74	7.37	7.67	7.88	-0.83
A_09_P044961	FBtr0335200	42587	CG6455	10.61	10.89	10.77	10.53	10.42	9.81	10.11	10.18	-0.57
A_09_P044966	FBtr0084599	42891	Orct	11.92	12.43	11.91	12.16	9.95	10.60	10.89	11.24	-1.44
A_09_P044971	FBtr0089489	33211	alpha-Adaptin	9.78	9.95	10.02	9.96	9.19	9.14	9.35	9.29	-0.68
A_09_P045041	FBtr0079801	34251	Eaat1	7.39	7.66	7.79	7.44	6.78	6.53	6.39	6.35	-1.06
A_09_P045166	FBtr0082106	41158	Kap-alpha3	12.38	12.43	12.39	12.32	12.23	11.70	12.04	11.97	-0.40
A_09_P045196	FBtr0087840	36387	spt4	9.85	9.80	9.46	9.76	8.65	8.82	9.23	9.50	-0.67
A_09_P045246	FBtr0084470	42802	Rpn9	11.92	12.15	11.73	11.87	11.07	10.73	11.33	11.58	-0.74
A_09_P045266	FBtr0079801	34251	Eaat1	10.81	11.10	10.83	10.87	10.58	10.00	10.16	10.00	-0.72
A_09_P045276	FBtr0072925	44251	RpL8	16.46	16.44	16.50	16.50	16.58	16.55	16.58	16.51	0.08
A_09_P045286	FBtr0343382	34089	CG7224	13.51	13.79	13.80	13.82	15.52	15.91	15.34	14.64	1.62
A_09_P045301	FBtr0077659	33524	CG3523	14.92	15.31	14.82	14.92	13.63	13.96	14.22	14.43	-0.93

A_09_P045311	FBtr0088001	36329	Cam	15.55	15.50	15.40	15.35	15.80	15.68	15.66	15.60	0.24
A_09_P045321	FBtr0075714	39594	RecQ5	9.87	9.79	10.00	9.90	10.82	10.82	10.57	10.39	0.76
A_09_P045331	FBtr0077173	38605	shep	7.73	7.73	7.42	7.32	8.55	8.82	8.67	8.67	1.13
A_09_P045336	FBtr0300844	37102	CG15067	7.67	4.51	5.45	4.22	12.97	12.73	12.15	11.04	6.76
A_09_P045371	FBtr0100293	35418	Df31	11.27	11.95	11.52	11.91	10.93	10.87	11.08	11.07	-0.67
A_09_P045386	FBtr0077174	38605	shep	8.72	8.75	8.38	8.89	9.27	9.64	9.43	9.23	0.71
A_09_P045436	FBtr0309232	50003	CG43462	9.80	9.73	9.58	9.66	10.60	11.02	10.60	10.66	1.03
A_09_P045471	FBtr0076103	59176	CG18815	13.69	13.48	13.61	13.51	13.20	12.98	13.24	13.34	-0.38
A_09_P045496	FBtr0083762	42325	CG31221	8.08	7.51	7.26	7.33	8.61	8.56	8.69	8.52	1.05
A_09_P045511	FBtr0078810	45467	mtd	7.98	7.62	7.59	7.42	8.45	8.35	8.48	8.60	0.82
A_09_P045516	FBtr0308614	32505	CG9240	10.65	10.65	10.61	10.82	10.35	10.38	10.33	10.36	-0.33
A_09_P045521	FBtr0074798	33002	Nup205	11.88	11.75	11.76	11.82	11.40	11.33	11.62	11.61	-0.31
A_09_P045536	FBtr0079191	33857	CG13993	12.03	11.72	11.96	11.84	11.44	11.25	11.57	11.61	-0.42
A_09_P045571	FBtr0079663	34167	emb	10.14	10.37	10.34	10.26	9.90	9.86	9.76	9.74	-0.47
A_09_P045621	FBtr0089905	43991	Pten	8.66	8.52	8.45	8.46	8.13	8.11	8.15	8.14	-0.39
A_09_P045656	FBtr0076204	39238	Mocs1	8.31	8.80	8.57	8.50	9.14	9.14	9.09	9.27	0.62
A_09_P045666	FBtr0301423	46017	l(2)01289	7.38	8.02	8.13	7.85	6.61	6.61	6.76	7.03	-1.09
A_09_P045696	FBtr0076093	39284	chrb	8.39	8.70	8.03	8.19	9.95	10.21	9.55	9.63	1.51
A_09_P045711	FBtr0073471	44072	ran	9.84	9.94	10.21	9.83	9.25	8.81	9.37	9.54	-0.71
A_09_P045716	FBtr0308085	32132	CkIIbeta	10.25	10.24	10.20	9.99	9.77	9.59	9.86	9.99	-0.37
A_09_P045721	FBtr0308085	32132	CkIIbeta	11.11	11.03	11.03	10.94	10.65	10.34	10.60	10.77	-0.44
A_09_P045761	FBtr0089126	43788	Hcf	10.21	10.35	10.31	10.44	10.16	9.97	10.15	10.07	-0.24
A_09_P045811	FBtr0080547	34803	cenG1A	6.58	6.73	6.51	6.38	8.04	7.99	7.30	7.16	1.07
A_09_P045861	FBtr0301378	42350	GluClalpha	5.82	6.18	6.02	6.01	4.76	4.85	4.88	5.48	-1.02
A_09_P045891	FBtr0086621	37184	DptB	7.47	10.09	6.46	8.36	15.22	14.12	14.92	14.87	6.69
A_09_P045961	FBtr0088021	36295	jeb	4.92	4.16	4.11	3.61	5.77	6.50	6.94	6.03	2.11
A_09_P045976	FBtr0329929	35085	Socs36E	9.82	9.42	9.27	9.12	12.79	12.75	12.21	11.52	2.91
A_09_P046026	FBtr0079310	33942	GRHR	7.94	8.54	8.55	8.00	5.27	5.86	6.70	7.34	-1.97
A_09_P046076	FBtr0301707	43936	Men-b	8.49	8.81	8.73	8.62	8.49	8.12	7.92	7.86	-0.56
A_09_P046081	FBtr0081473	44149	Acon	6.70	7.10	6.98	6.25	5.69	5.41	5.63	5.79	-1.13
A_09_P046096	FBtr0100268	3772329	Chrac-14	9.88	9.78	9.75	9.88	9.54	9.58	9.62	9.74	-0.20

A_09_P046111	FBtr0084061	49228	mod(mdg4)	7.63	7.86	7.64	7.70	6.53	6.65	6.72	6.78	-1.04
A_09_P046116	FBtr0084080	49228	mod(mdg4)	9.85	10.30	10.15	10.07	9.32	9.19	9.35	9.60	-0.73
A_09_P046156	FBtr0084075	49228	mod(mdg4)	8.51	8.60	8.49	8.62	7.95	8.08	7.98	7.94	-0.57
A_09_P046186	FBtr0075707	2768981	Trl	8.49	7.99	8.56	8.17	7.35	7.58	7.74	7.33	-0.80
A_09_P046201	NR_002544	3772130	noRNA:U3:54A	8.78	9.14	8.52	8.84	9.34	9.36	9.25	9.22	0.47
A_09_P046361	FBtr0301481	39777	MED10	11.73	11.66	11.70	11.87	10.96	11.06	11.37	11.45	-0.53
A_09_P046421	FBtr0310384	326128	Ada2a	7.27	7.43	7.43	7.44	5.67	6.21	6.52	6.62	-1.14
A_09_P046516	FBtr0074344	32682	CG4928	13.03	13.04	13.03	13.00	14.08	13.96	13.55	13.47	0.74
A_09_P046521	FBtr0076777	45588	Pdp1	9.97	9.62	9.84	9.52	10.62	10.74	10.17	10.26	0.71
A_09_P046546	FBtr0330660	33392	aop	10.85	11.04	10.55	10.42	12.43	12.47	11.53	11.58	1.29
A_09_P046581	FBtr0089649	33692	Cf2	8.33	8.94	8.63	9.01	7.90	8.12	8.08	8.23	-0.64
A_09_P046641	FBtr0300377	43828	CaMKII	12.55	12.71	12.74	12.77	11.96	11.96	12.02	12.08	-0.69
A_09_P046841	FBtr0300671	3346208	dpr3	8.07	7.99	7.73	8.02	8.33	8.65	8.49	8.41	0.52
A_09_P046941	FBtr0308699	32458	Top1	7.41	7.60	6.93	7.28	8.12	8.77	8.30	8.13	1.03
A_09_P046951	FBtr0302863	32941	CoRest	8.75	9.09	8.94	9.14	8.57	8.40	8.36	8.53	-0.52
A_09_P046966	FBtr0332561	34560	ab	11.15	10.83	10.75	10.81	11.23	11.72	11.59	11.75	0.69
A_09_P047036	FBtr0085826	48317	ttk	9.63	9.57	9.50	9.69	9.89	9.96	9.93	9.94	0.33
A_09_P047066	FBtr0339411	50246	CoVIIc	14.33	14.32	14.45	14.51	13.80	13.91	14.03	14.19	-0.42
A_09_P047151	FBtr0301089	41851	l(3)neo43	11.32	11.17	11.35	11.22	10.49	10.72	10.54	10.50	-0.70
A_09_P047156	FBtr0077157	38611	Klp64D	10.45	10.62	10.55	10.56	10.29	10.24	10.20	10.35	-0.27
A_09_P047196	FBtr0301163	34685	CG5792	9.57	9.75	9.74	9.86	10.23	10.20	10.06	9.92	0.37
A_09_P047236	FBtr0071208	31792	Nrg	10.17	10.36	10.42	10.36	11.62	11.95	11.18	10.95	1.10
A_09_P047446	FBtr0111009	32428	eag	5.29	4.44	4.06	3.81	6.33	6.74	5.94	6.56	1.99
A_09_P047646	FBtr0299519	35238	CG10186	8.28	8.11	8.13	7.96	8.84	9.17	8.97	8.70	0.80
A_09_P047836	FBtr0339331	5740784	CG11741	6.25	5.95	5.70	5.56	6.87	7.04	7.05	7.20	1.17
A_09_P047841	FBtr0299508	7354415	CG42246	8.54	7.57	7.94	7.21	9.34	9.24	9.06	8.76	1.28
A_09_P047921	FBtr0112775	37310	CG12484	6.81	6.58	6.50	6.10	7.55	8.02	7.57	7.25	1.10
A_09_P047986	FBtr0114361	49228	mod(mdg4)	7.91	8.19	8.27	8.23	7.17	7.56	7.51	7.81	-0.64
A_09_P048041	FBtr0340396	32321	NFAT	7.40	7.51	6.70	6.67	8.21	8.48	7.82	7.97	1.05
A_09_P048106	FBtr0087746	36471	CG4716	4.70	5.39	4.31	5.05	7.51	9.11	8.14	7.97	3.32
A_09_P048221	FBtr0301880	31448	Rnp4F	8.82	9.48	8.95	9.17	7.96	7.71	8.24	8.62	-0.97

A_09_P048331	FBtr0070575	44126	AlstR	4.55	4.02	4.10	3.41	5.67	6.26	5.52	5.77	**1.78**
A_09_P048366	FBtr0112710	43892	sif	8.17	8.07	7.66	7.29	8.78	9.25	8.45	9.07	**1.09**
A_09_P048381	FBtr0333892	40424	Syn1	4.89	4.88	4.74	4.40	5.70	6.10	5.62	5.95	**1.12**
A_09_P048401	FBtr0084079	49228	mod(mdg4)	11.74	11.98	11.95	11.95	11.18	11.22	11.32	11.51	**-0.60**
A_09_P048571	FBtr0082590	41531	Pros25	13.22	13.30	13.18	13.29	12.63	12.64	12.99	13.07	**-0.42**
A_09_P048596	FBtr0076138	39304	CG6084	15.88	15.46	15.78	15.40	13.44	13.42	14.20	14.56	**-1.72**
A_09_P048611	FBtr0085241	43330	ALiX	13.30	13.38	13.43	13.63	14.45	14.52	14.21	13.91	**0.84**
A_09_P048626	FBtr0306040	44259	nbs	10.02	10.18	10.11	10.15	10.88	10.86	11.00	11.25	**0.88**
A_09_P048631	FBtr0087014	36920	tef	7.22	7.43	7.29	7.45	6.83	7.06	6.97	7.07	**-0.37**
A_09_P048636	FBtr0085582	43582	Tpi	15.83	15.91	15.87	15.98	15.37	15.38	15.63	15.77	**-0.36**
A_09_P048646	FBtr0079266	33905	Sec61alpha	14.58	14.62	14.71	14.56	13.64	13.42	14.04	14.32	**-0.76**
A_09_P048661	FBtr0083718	42283	gatA	11.39	11.10	11.32	11.16	10.84	10.76	10.88	10.99	**-0.38**
A_09_P048671	FBtr0085760	43700	spn-F	9.07	9.21	9.12	9.29	10.10	10.21	9.98	10.00	**0.90**
A_09_P048686	FBtr0071335	31863	t	8.37	8.55	8.63	8.44	6.24	6.67	7.62	8.07	**-1.35**
A_09_P048722	FBtr0301810	3355106	Alg-2	12.09	11.96	11.81	12.03	11.68	11.24	11.69	11.67	**-0.40**
A_09_P048776	FBtr0340312	117366	Gpi1	8.14	8.23	7.86	8.01	7.38	7.05	7.42	7.77	**-0.65**
A_09_P048801	FBtr0084978	43163	exo84	8.43	8.67	8.49	8.39	8.18	8.25	8.17	8.30	**-0.27**
A_09_P048831	FBtr0113192	40557	CG9853	12.69	12.69	12.73	12.68	12.37	12.09	12.30	12.37	**-0.41**
A_09_P048851	FBtr0077061	59159	Lcp65Ag3	15.58	15.35	15.44	15.43	15.83	15.89	15.74	15.81	**0.37**
A_09_P048856	FBtr0083771	42314	Ino80	8.01	8.20	7.99	8.15	6.44	6.73	7.14	7.34	**-1.18**
A_09_P048871	FBtr0088623	35933	shrb	11.86	12.05	11.91	12.07	12.59	12.36	12.33	12.16	**0.39**
A_09_P048891	FBtr0073712	32245	fne	11.03	11.12	10.99	11.13	11.58	11.74	11.59	11.73	**0.59**
A_09_P048906	FBtr0081887	41025	p	10.30	10.64	10.65	10.71	9.88	9.95	10.07	10.19	**-0.55**
A_09_P048941	FBtr0306596	40461	srpk79D	9.87	10.53	10.57	10.78	9.33	9.28	9.17	9.25	**-1.18**
A_09_P049006	FBtr0301661	41550	Vha55	14.13	14.04	14.03	14.04	14.00	13.74	13.67	13.54	**-0.32**
A_09_P049026	FBtr0082136	41200	RpS29	15.84	15.66	15.74	15.74	15.83	15.88	15.92	15.91	**0.14**
A_09_P049086	FBtr0305094	39198	A2bp1	11.27	11.58	11.33	11.25	12.28	12.40	11.78	11.86	**0.72**
A_09_P049141	FBtr0330276	45840	cpo	10.03	10.10	10.07	9.84	11.01	11.06	10.72	10.89	**0.91**
A_09_P049151	FBtr0329880	36643	Spred	8.43	8.35	8.17	8.39	8.49	8.61	8.66	8.69	**0.27**
A_09_P049166	FBtr0083099	41883	Atx2	10.55	11.12	10.64	10.27	11.60	11.83	10.96	11.74	**0.89**
A_09_P049191	FBtr0079928	45248	Nckx30C	5.37	4.99	5.13	5.13	6.17	6.79	6.01	6.63	**1.24**

A_09_P049206	FBtr0330623	36238	E(Pc)	5.54	5.42	5.28	5.30	6.38	6.42	6.34	6.61	1.05
A_09_P049211	FBtr0080052	2768916	CG33303	11.97	11.95	11.84	11.89	10.78	10.56	11.34	11.59	-0.85
A_09_P049216	FBtr0304056	34274	jp	10.52	11.30	11.14	10.72	10.30	9.81	9.54	10.21	-0.95
A_09_P049256	FBtr0085438	44643	Cnx99A	12.30	12.51	12.22	12.56	11.53	11.55	11.95	12.22	-0.59
A_09_P049271	FBtr0074404	44271	xmas-2	7.88	8.02	8.07	7.92	6.95	7.68	7.33	7.60	-0.59
A_09_P049281	FBtr0331841	39284	chrb	10.73	11.21	10.66	11.05	11.99	12.23	11.72	11.61	0.97
A_09_P049296	FBtr0333754	31179	Unc-76	9.19	9.10	9.15	9.25	9.65	9.71	9.37	9.36	0.35
A_09_P049381	FBtr0071700	37453	CG10494	7.49	8.06	7.69	8.08	6.85	7.05	7.12	7.29	-0.75
A_09_P049456	FBtr0339765	50225	Prosap	10.90	11.19	10.80	10.96	11.57	12.00	11.53	11.48	0.68
A_09_P049486	FBtr0306292	43649	Npc2h	9.55	9.97	9.75	9.87	9.00	9.21	9.15	9.27	-0.62
A_09_P049516	FBtr0075394	39842	mbf1	11.50	11.52	11.50	11.48	10.50	10.62	11.09	11.38	-0.60
A_09_P049541	FBtr0075150	39999	Eip75B	8.74	7.85	8.29	7.75	8.94	9.09	9.05	9.03	0.87
A_09_P049586	FBtr0332676	44013	sls	10.12	11.13	10.86	10.37	11.91	12.37	11.72	11.79	1.33
A_09_P049646	FBtr0085826	48317	ttk	8.81	9.18	9.17	8.79	9.63	9.58	9.26	9.46	0.49
A_09_P049671	FBtr0334482	43814	bt	10.67	11.61	11.21	11.11	9.69	10.28	9.60	10.55	-1.12
A_09_P049676	FBtr0339701	32288	CG15747	9.19	9.35	9.42	9.47	8.72	9.10	8.94	8.95	-0.43
A_09_P049706	FBtr0306086	38612	CG4769	15.32	15.26	15.45	15.18	14.82	14.80	15.05	15.13	-0.35
A_09_P049741	FBtr0304998	38895	Ect4	6.39	6.92	6.28	6.11	8.59	8.62	7.64	7.76	1.73
A_09_P049746	FBtr0303818	10178961	CG42808	12.26	11.64	11.91	11.85	13.51	13.71	13.15	12.65	1.34
A_09_P049766	FBtr0335418	42350	GluClalpha	6.72	6.79	6.28	6.36	7.71	8.48	7.74	8.24	1.51
A_09_P049836	FBtr0089447	317815	CG32000	7.69	8.13	7.96	7.76	7.16	7.27	6.90	7.39	-0.71
A_09_P049896	FBtr0334482	43814	bt	14.77	15.22	14.89	14.95	14.10	14.30	14.11	14.66	-0.67
A_09_P049951	FBtr0331711	32337	Pdcd4	9.49	10.18	9.60	10.33	11.09	11.10	10.76	10.65	1.00
A_09_P049981	FBtr0080774	34924	CycE	6.14	6.13	6.82	6.50	5.05	5.44	5.43	5.79	-0.97
A_09_P050036	FBtr0086681	37116	Dp1	12.03	12.25	12.23	12.11	11.19	11.30	11.55	11.64	-0.73
A_09_P050051	FBtr0300588	41670	B52	13.96	13.82	13.88	13.88	13.46	13.41	13.69	13.64	-0.33
A_09_P050061	FBtr0076093	39284	chrb	9.66	10.48	10.07	9.80	11.34	11.72	10.77	11.13	1.24
A_09_P050071	FBtr0078212	40285	knrl	9.19	9.16	9.22	8.81	9.90	10.14	9.57	9.75	0.75
A_09_P050081	FBtr0321255	42806	Hrd3	11.50	11.46	11.61	11.57	10.87	10.76	11.07	11.04	-0.60
A_09_P050146	FBtr0076972	59223	CG18769	12.61	12.82	12.74	12.74	12.00	11.76	12.11	12.31	-0.68
A_09_P050176	FBtr0112830	43906	skd	6.71	7.05	6.24	6.57	7.36	7.61	7.40	7.08	0.72

A_09_P050196	FBtr0333955	38050	RhoGEF3	7.09	7.25	7.29	7.30	8.11	8.29	7.85	7.51	**0.71**
A_09_P050246	FBtr0089983	47220	Fur1	9.98	10.07	10.24	10.03	10.98	11.17	10.55	10.47	**0.71**
A_09_P050256	FBtr0300509	32771	mnb	8.71	8.98	8.66	8.39	9.45	9.63	9.08	9.56	**0.74**
A_09_P050406	FBtr0075909	39440	caup	10.00	10.37	10.25	10.33	9.87	9.77	9.61	9.66	**-0.51**
A_09_P050506	FBtr0343706	34853	CG15282	16.14	15.62	15.94	15.92	16.58	16.59	16.53	16.41	**0.63**
A_09_P050536	FBtr0308694	32245	fne	7.45	7.60	7.13	6.79	8.01	8.45	7.99	8.39	**0.97**
A_09_P050596	FBtr0302695	44258	ps	13.17	13.05	12.90	13.17	13.77	14.06	13.71	13.38	**0.66**
A_09_P050606	FBtr0332830	39153	CG43897	12.56	13.07	12.89	13.01	12.35	11.90	12.10	12.17	**-0.75**
A_09_P050691	FBtr0086621	37184	DptB	7.94	10.66	7.02	8.86	15.59	14.65	15.41	15.26	**6.61**
A_09_P050771	FBtr0073165	38469	scrt	8.45	8.64	8.36	8.63	9.58	9.99	9.38	9.23	**1.02**
A_09_P050861	FBtr0300504	35509	ap	7.91	7.94	7.97	7.74	6.48	6.82	6.97	7.12	**-1.04**
A_09_P050901	FBtr0301402	42596	how	8.70	8.65	8.81	8.65	8.45	8.40	8.40	8.00	**-0.39**
A_09_P050991	FBtr0301310	41289	Cap-H2	7.27	7.28	7.25	7.19	6.43	6.70	6.59	6.67	**-0.64**
A_09_P051006	FBtr0082799	47998	rin	10.17	10.53	10.59	10.37	9.91	10.08	9.81	9.86	**-0.50**
A_09_P051011	FBtr0089658	32780	Sh	8.62	8.46	7.98	8.50	9.21	9.40	9.16	9.11	**0.83**
A_09_P051051	FBtr0305152	44669	cwo	10.01	10.07	10.21	10.11	11.05	11.31	10.75	10.58	**0.82**
A_09_P051091	FBtr0339465	36129	CG7220	12.61	12.48	12.40	12.42	13.65	13.90	13.44	13.06	**1.04**
A_09_P051101	FBtr0082199	37528	Fmr1	8.90	9.02	9.12	9.09	8.62	8.76	8.50	8.69	**-0.39**
A_09_P051181	FBtr0071117	31724	Upf2	10.91	10.88	10.87	10.91	10.68	10.67	10.49	10.54	**-0.30**
A_09_P051191	FBtr0113384	43105	LpR2	9.66	9.13	9.31	9.16	10.00	9.95	9.88	9.56	**0.54**
A_09_P051221	FBtr0083999	42481	Calx	8.24	7.59	8.26	8.07	7.51	7.18	7.06	6.99	**-0.85**
A_09_P051361	FBtr0112947	30988	CG13373	7.19	7.04	6.95	6.88	6.00	5.78	6.45	6.66	**-0.79**
A_09_P051581	FBtr0083947	42435	Elongin-B	10.77	10.90	10.99	10.94	10.24	10.01	10.21	10.25	**-0.73**
A_09_P051586	FBtr0086541	37237	Elongin-C	12.69	12.73	12.83	12.88	12.45	12.64	12.59	12.44	**-0.25**
A_09_P051646	FBtr0086259	37323	Mgat1	7.42	7.89	7.45	7.86	6.09	6.13	6.82	7.30	**-1.07**
A_09_P051946	FBtr0085088	43236	Ets97D	8.47	8.51	8.46	8.46	7.68	7.93	8.01	8.31	**-0.49**
A_09_P051961	FBtr0076534	39054	Rdl	7.26	7.14	6.94	7.01	8.04	8.61	7.89	8.05	**1.06**
A_09_P051966	FBtr0309054	39054	Rdl	6.34	5.20	5.51	5.47	6.31	6.95	6.77	7.03	**1.13**
A_09_P052126	FBtr0084080	49228	mod(mdg4)	9.94	10.37	10.21	10.10	9.63	9.28	9.43	9.64	**-0.66**
A_09_P052141	FBtr0089056	35634	Cyp9b1	8.14	8.52	8.57	8.39	7.32	7.49	7.84	7.84	**-0.78**
A_09_P052146	FBtr0089055	35635	Cyp9b2	12.70	13.64	13.15	13.34	12.03	11.71	11.88	11.92	**-1.32**

A_09_P052176	FBtr0070388	31193	Cyp4ae1	6.49	6.04	6.36	5.43	7.35	7.53	7.09	6.70	1.09
A_09_P052181	FBtr0339278	35822	Cyp4e2	10.78	11.34	10.72	11.25	9.69	9.40	9.79	9.98	-1.31
A_09_P052216	FBtr0077114	38654	S6k	10.00	10.04	10.03	9.62	11.21	11.09	10.49	10.42	0.88
A_09_P052221	FBtr0083018	41785	eff	11.13	11.13	11.24	10.74	10.74	10.09	10.51	10.64	-0.57
A_09_P052256	FBtr0082434	41366	Blm	7.99	7.83	8.03	8.04	7.57	7.58	7.54	7.49	-0.43
A_09_P052276	FBtr0335153	35341	cad	5.49	5.17	5.17	5.68	6.42	6.24	6.25	6.06	0.86
A_09_P052336	FBtr0089510	48971	Atpalpha	7.03	7.44	6.98	6.86	6.10	6.31	6.08	6.33	-0.87
A_09_P052341	FBtr0072218	49297	Ca-P60A	13.12	13.85	13.20	13.50	12.03	10.50	12.03	12.37	-1.68
A_09_P052361	FBtr0089649	33692	Cf2	8.11	8.20	8.14	8.17	7.56	7.57	7.62	7.80	-0.52
A_09_P052406	FBtr0111037	45928	shi	11.36	11.54	11.58	11.53	11.99	12.19	11.87	11.92	0.49
A_09_P052436	FBtr0334482	43814	bt	8.20	9.08	8.70	8.59	6.93	7.33	7.15	7.87	-1.32
A_09_P052461	FBtr0084079	49228	mod(mdg4)	11.45	11.60	11.63	11.79	10.91	10.89	11.12	11.25	-0.58
A_09_P052471	FBtr0089585	39916	TpnC73F	15.87	15.82	15.98	15.84	15.32	15.13	15.43	15.42	-0.55
A_09_P052491	FBtr0074295	32623	Arp2	9.82	10.03	9.93	9.98	10.14	10.27	10.05	10.17	0.22
A_09_P052581	FBtr0073244	38518	CG15011	10.55	10.80	10.63	10.70	9.52	9.67	10.09	10.45	-0.74
A_09_P052626	FBtr0073233	38526	CG1316	9.19	9.54	9.27	9.31	8.72	8.15	8.67	8.94	-0.71
A_09_P052636	FBtr0300775	38530	CG1319	12.53	12.47	12.50	12.49	12.33	12.15	12.20	12.27	-0.26
A_09_P052661	FBtr0100185	38539	DopEcR	9.18	8.86	9.02	9.00	10.23	10.11	9.93	9.75	0.99
A_09_P052666	FBtr0100186	38539	DopEcR	8.22	8.34	7.95	7.57	9.46	9.36	8.50	9.07	1.08
A_09_P052676	FBtr0073293	38541	Syx17	9.95	10.46	10.23	10.48	10.90	11.01	10.69	10.61	0.52
A_09_P052756	FBtr0113143	38561	CG11357	10.53	10.72	10.72	10.65	11.03	10.93	10.93	10.95	0.30
A_09_P052841	FBtr0073352	38587	CG13705	15.21	15.36	15.07	15.24	15.53	15.51	15.45	15.35	0.24
A_09_P052846	FBtr0073353	38588	CG13704	10.96	10.37	10.62	10.43	11.82	11.79	11.63	11.25	1.03
A_09_P052871	FBtr0077179	38598	CG10672	13.29	13.15	13.13	13.17	13.64	13.80	13.72	13.81	0.56
A_09_P052896	FBtr0077164	38603	CG4603	12.25	12.50	12.51	12.66	11.17	11.08	11.63	11.94	-1.03
A_09_P052916	FBtr0077155	38613	Uev1A	12.69	12.92	12.85	12.90	12.50	12.26	12.48	12.60	-0.38
A_09_P052921	FBtr0077145	38614	membrin	12.71	12.93	12.79	13.01	11.91	11.87	12.26	12.54	-0.71
A_09_P052926	FBtr0077154	38615	CG10635	8.52	8.49	8.14	8.32	7.36	7.22	7.67	8.12	-0.77
A_09_P052946	FBtr0077149	38622	Lkr	8.71	9.22	9.13	9.05	8.68	8.29	8.37	8.20	-0.64
A_09_P052986	FBtr0077127	38635	CG10590	12.13	12.39	12.14	12.33	11.27	10.89	11.55	11.70	-0.90
A_09_P052991	FBtr0077088	38636	mthl2	8.29	8.24	8.39	8.12	7.70	7.92	7.59	7.14	-0.67

A_09_P052996	FBtr0077126	38637	Eaf6	10.11	9.93	10.17	10.12	9.23	9.20	9.54	9.67	**-0.67**
A_09_P053006	FBtr0077090	38639	lin-28	6.21	6.19	4.90	4.71	7.95	8.81	8.40	8.58	**2.94**
A_09_P053051	FBtr0331522	38653	Tektin-C	6.58	5.84	6.65	6.77	10.92	11.14	10.70	10.47	**4.35**
A_09_P053056	FBtr0077112	38655	CG5537	13.49	13.43	13.65	13.56	13.22	13.39	13.26	13.29	**-0.24**
A_09_P053081	FBtr0077081	38661	Pole2	9.26	8.94	9.05	8.99	8.22	8.04	8.49	8.68	**-0.70**
A_09_P053101	FBtr0077077	38666	CG10483	10.08	10.27	10.25	10.31	9.53	9.54	9.44	9.28	**-0.78**
A_09_P053106	FBtr0077070	38674	CG10479	7.66	8.27	7.74	8.20	7.35	7.26	7.17	7.09	**-0.75**
A_09_P053136	FBtr0077042	38684	Jon65Aii	9.84	9.48	7.93	8.61	13.61	12.45	12.88	12.20	**3.82**
A_09_P053151	FBtr0077066	38688	CG10472	15.42	15.22	14.93	14.96	16.16	16.07	16.02	15.85	**0.89**
A_09_P053176	FBtr0077048	38693	CG6610	11.02	10.97	10.94	11.06	10.40	10.56	10.60	10.79	**-0.41**
A_09_P053191	FBtr0077064	38696	CG10469	8.72	9.35	9.21	9.51	8.35	8.71	8.34	8.30	**-0.77**
A_09_P053196	FBtr0077063	38697	CG10467	10.87	11.80	11.73	12.05	10.19	10.10	10.08	10.05	**-1.51**
A_09_P053231	FBtr0076999	38719	CG7386	8.53	8.52	8.71	8.83	8.47	7.92	8.18	8.22	**-0.45**
A_09_P053236	FBtr0077015	38720	CG13298	12.95	13.06	13.03	13.19	12.91	12.66	12.78	12.87	**-0.25**
A_09_P053291	FBtr0076986	38735	CG10144	9.72	9.79	9.76	9.92	10.18	10.00	10.10	9.95	**0.26**
A_09_P053326	FBtr0076943	38749	CG8549	11.22	11.20	11.31	11.22	11.76	11.76	11.72	11.76	**0.51**
A_09_P053331	FBtr0076949	38750	CG10103	9.30	9.48	9.43	9.45	11.42	11.31	10.87	10.55	**1.62**
A_09_P053336	FBtr0076894	38754	CG14820	10.80	9.45	9.84	9.64	12.03	11.61	11.71	11.41	**1.75**
A_09_P053341	FBtr0333079	38755	tow	7.17	7.05	6.89	7.09	7.78	8.02	7.73	7.55	**0.72**
A_09_P053401	FBtr0076905	38772	CG14823	9.18	9.85	9.94	10.02	7.86	8.18	8.53	8.54	**-1.47**
A_09_P053431	FBtr0076925	38781	CG8629	14.32	14.64	14.53	14.59	14.31	14.02	14.04	13.78	**-0.48**
A_09_P053441	FBtr0076914	38783	CG8628	10.55	10.31	10.30	10.58	9.58	9.69	9.71	9.62	**-0.79**
A_09_P053481	FBtr0290001	38801	unc-13-4A	5.07	5.15	4.68	4.67	6.77	7.19	6.57	6.50	**1.86**
A_09_P053496	FBtr0076838	38807	CG8605	11.25	11.39	11.42	11.43	10.78	10.90	11.05	11.21	**-0.39**
A_09_P053516	FBtr0076881	38813	CG8596	11.51	11.82	11.59	11.67	10.99	11.39	11.12	11.12	**-0.49**
A_09_P053531	FBtr0273398	38818	pst	13.89	13.45	13.70	13.53	15.26	14.80	14.94	14.61	**1.26**
A_09_P053536	FBtr0076847	38819	sec63	13.47	13.58	13.54	13.60	13.00	12.96	13.23	13.43	**-0.39**
A_09_P053576	FBtr0332797	38835	CG8546	6.11	5.76	5.60	5.26	6.59	6.44	6.49	6.77	**0.89**
A_09_P053636	FBtr0301222	38850	frac	11.64	11.60	11.63	11.47	11.25	10.93	11.16	11.19	**-0.45**
A_09_P053651	FBtr0089850	2768948	CG33275	10.17	9.88	10.18	9.82	10.34	10.46	10.33	10.24	**0.33**
A_09_P053706	FBtr0076748	38880	CG8281	6.98	6.96	6.73	6.53	7.27	7.50	7.28	7.40	**0.57**

A_09_P053721	FBtr0076765	38887	HP4	10.81	10.91	10.94	11.03	10.11	9.96	10.45	10.75	-0.60
A_09_P053726	FBtr0076753	38888	CG8209	9.80	10.07	10.02	10.07	8.75	9.01	9.28	9.46	-0.87
A_09_P053776	FBtr0076690	38911	CG7979	9.99	10.39	10.25	10.24	9.37	9.67	9.55	9.59	-0.68
A_09_P053781	FBtr0076724	38912	ERR	9.43	9.40	9.28	9.24	10.01	9.77	9.66	9.61	0.42
A_09_P053786	FBtr0076693	38913	Atg18	11.53	11.93	11.66	11.77	12.66	12.73	12.34	12.23	0.77
A_09_P053801	FBtr0076722	38916	Ubc12	11.54	11.52	11.54	11.57	11.25	11.09	11.17	11.22	-0.36
A_09_P053851	FBtr0299841	2769008	Dscam4	4.58	4.62	3.56	3.80	7.36	7.98	7.28	7.50	3.39
A_09_P053866	FBtr0076706	38932	CG13671	10.70	10.86	10.73	10.77	10.21	10.17	10.34	10.56	-0.45
A_09_P053891	FBtr0076710	38937	CG7185	13.01	13.32	13.10	13.23	12.60	12.70	12.81	13.00	-0.39
A_09_P053926	FBtr0076676	38946	CG17352	8.62	9.52	8.92	9.73	9.95	10.48	10.15	10.35	1.03
A_09_P053951	FBtr0076610	38956	CG13667	8.68	8.21	8.35	8.18	9.59	9.35	9.44	9.47	1.11
A_09_P053966	FBtr0076661	38963	Unr	9.34	9.68	9.71	9.56	8.94	8.90	8.81	8.96	-0.67
A_09_P053976	FBtr0308608	38966	CG43163	7.87	8.57	8.09	8.45	6.72	7.50	7.40	7.75	-0.90
A_09_P054026	FBtr0076626	44679	ergic53	13.88	14.17	13.94	14.00	13.19	13.07	13.35	13.79	-0.65
A_09_P054031	FBtr0076651	38979	CG6638	12.76	12.92	12.99	12.93	11.78	11.80	12.11	12.36	-0.89
A_09_P054036	FBtr0076637	38985	CG6282	7.16	6.65	6.70	6.44	7.62	7.59	7.55	7.49	0.83
A_09_P054051	FBtr0089561	38988	Zasp66	14.80	15.18	15.04	15.14	13.85	13.43	14.01	14.26	-1.15
A_09_P054061	FBtr0076647	38989	Cdc6	9.49	9.79	9.77	9.88	8.15	8.31	8.67	9.04	-1.19
A_09_P054071	FBtr0076568	38994	Pex7	7.90	6.84	7.25	6.70	8.79	9.05	8.57	8.37	1.52
A_09_P054086	FBtr0076591	39005	CG5804	16.00	15.78	15.88	15.79	15.40	15.60	15.51	15.44	-0.38
A_09_P054131	FBtr0331519	39018	orb2	8.92	9.27	9.10	8.98	8.67	8.66	8.50	8.42	-0.51
A_09_P054161	FBtr0076516	39026	CG5026	9.00	9.40	9.45	9.57	8.81	8.97	8.81	8.82	-0.50
A_09_P054166	FBtr0076557	39027	pix	14.65	14.67	14.75	14.68	15.12	15.24	15.11	15.03	0.43
A_09_P054171	FBtr0076517	39028	Srp68	12.12	12.23	12.11	12.10	11.05	11.02	11.30	11.55	-0.91
A_09_P054191	FBtr0076519	39032	CG5068	9.05	8.91	9.08	8.93	8.60	8.50	8.55	8.58	-0.44
A_09_P054231	FBtr0076524	39044	CG4942	10.12	9.74	9.99	9.66	9.35	9.32	9.48	9.56	-0.45
A_09_P054236	FBtr0076531	39050	Dhpr	11.77	11.77	11.83	11.71	12.19	12.35	12.33	12.35	0.53
A_09_P054251	FBtr0309280	39052	nwk	8.44	7.68	7.91	7.51	9.60	9.38	9.25	9.04	1.43
A_09_P054276	FBtr0076435	39062	PGRP-LA	10.41	9.45	9.96	9.73	11.00	11.09	11.04	10.98	1.14
A_09_P054286	FBtr0089491	39063	PGRP-LC	11.15	11.60	11.36	11.58	12.41	12.71	12.22	12.05	0.92
A_09_P054291	FBtr0089492	39063	PGRP-LC	7.62	7.69	7.36	7.62	9.57	9.46	9.12	8.68	1.63

A_09_P054296	FBtr0076439	39064	PGRP-LF	9.84	9.18	9.74	10.05	12.24	12.61	11.70	10.98	**2.18**
A_09_P054301	FBtr0076501	39065	UGP	12.42	12.66	12.51	12.70	11.07	11.11	11.66	12.00	**-1.11**
A_09_P054306	FBtr0076444	39067	CG4447	11.48	11.17	11.30	11.19	10.29	10.37	10.65	10.80	**-0.76**
A_09_P054326	FBtr0076455	39081	Cpr67B	15.17	15.18	15.17	15.32	16.27	16.43	16.21	15.95	**1.01**
A_09_P054336	FBtr0076456	39083	CG3689	11.16	11.31	11.24	11.36	10.86	10.85	10.95	11.15	**-0.31**
A_09_P054391	FBtr0076463	39097	CG3552	9.77	9.78	9.91	9.83	10.25	10.43	10.34	10.41	**0.54**
A_09_P054416	FBtr0076427	39106	path	9.37	9.06	9.36	9.20	9.72	9.48	9.70	9.55	**0.37**
A_09_P054486	FBtr0076389	39125	CG18180	16.05	16.08	15.87	15.99	16.23	16.43	16.47	16.32	**0.37**
A_09_P054491	FBtr0076392	39129	CG16717	9.51	9.70	9.59	9.68	8.91	8.92	8.93	9.24	**-0.62**
A_09_P054526	FBtr0076403	39141	defl	5.53	5.40	4.91	5.02	8.39	8.58	8.59	8.65	**3.34**
A_09_P054556	FBtr0076329	39150	Ilp2	9.18	9.00	9.23	9.15	9.37	9.77	9.52	9.52	**0.41**
A_09_P054566	FBtr0076364	39160	iPLA2-VIA	10.95	11.20	11.11	11.02	10.87	10.80	10.79	10.86	**-0.24**
A_09_P054616	FBtr0303833	39179	CG42825	10.43	9.63	10.10	9.53	13.01	12.12	12.55	12.35	**2.58**
A_09_P054671	FBtr0076255	39204	CG8009	9.30	9.16	9.19	9.21	8.79	8.73	8.85	8.91	**-0.39**
A_09_P054691	FBtr0076260	39210	CG8003	12.59	12.34	12.54	12.48	11.85	12.02	12.13	12.27	**-0.42**
A_09_P054696	FBtr0076265	39213	CG11811	12.88	12.84	12.92	12.70	12.58	12.42	12.37	12.33	**-0.41**
A_09_P054721	FBtr0076273	39220	CG6409	14.27	13.82	14.07	13.85	14.65	14.59	14.52	14.43	**0.54**
A_09_P054726	FBtr0076270	39221	CG7949	9.47	8.97	9.16	8.93	8.61	8.36	8.53	8.72	**-0.58**
A_09_P054766	FBtr0076242	39233	CG6321	8.32	8.25	8.34	8.14	8.00	7.87	7.79	7.68	**-0.43**
A_09_P054781	FBtr0076204	39238	Mocs1	11.52	11.70	11.75	11.78	12.37	12.66	12.38	12.21	**0.72**
A_09_P054796	FBtr0076234	39243	CG6272	8.74	8.78	8.76	8.72	9.40	9.39	9.33	9.29	**0.60**
A_09_P054816	FBtr0076210	39250	CG7638	12.91	12.62	12.77	12.48	11.72	11.73	12.12	12.39	**-0.70**
A_09_P054821	FBtr0300784	39252	fd68A	8.36	8.22	8.10	8.09	8.82	9.04	8.70	8.87	**0.66**
A_09_P054836	FBtr0301438	39255	CG42575	9.83	9.91	9.89	9.83	10.31	10.23	10.54	10.79	**0.60**
A_09_P054851	FBtr0076218	39259	wls	9.09	9.70	9.45	9.57	8.99	8.73	9.02	9.01	**-0.52**
A_09_P054881	FBtr0076187	39265	Plod	14.83	14.48	14.69	14.41	12.97	13.29	13.87	14.39	**-0.97**
A_09_P054921	FBtr0076173	39276	CG7560	11.06	11.26	11.13	11.07	9.42	9.63	9.92	10.21	**-1.33**
A_09_P054956	FBtr0076093	39284	chrb	8.57	8.76	8.28	8.22	9.07	9.17	9.33	9.31	**0.76**
A_09_P054966	FBtr0100588	3772574	Fuca	9.02	10.04	9.58	10.00	8.78	8.57	8.69	8.68	**-0.98**
A_09_P054971	FBtr0089332	39293	Mob2	6.47	6.30	6.05	5.99	7.30	7.29	7.07	6.96	**0.96**
A_09_P054976	FBtr0076098	39294	CG7394	12.33	11.99	12.20	12.09	11.52	11.57	11.69	11.84	**-0.50**

A_09_P054981	FBtr0076100	2768978	CG7377	7.97	5.46	5.26	5.35	10.45	10.30	10.18	9.79	4.17
A_09_P054986	FBtr0076145	2768979	CG33268	8.05	4.68	4.24	4.06	9.36	9.16	9.74	9.52	4.19
A_09_P055011	FBtr0076137	39305	CG6083	9.60	9.32	9.50	9.63	8.14	7.80	8.30	8.74	-1.27
A_09_P055016	FBtr0076102	39306	PCID2	11.03	10.86	11.05	10.87	10.37	10.27	10.53	10.77	-0.47
A_09_P055101	FBtr0076028	39328	CG14131	10.41	10.50	10.37	10.36	11.71	11.67	11.37	11.03	1.03
A_09_P055116	FBtr0076084	39333	CG10361	10.30	10.93	10.81	11.17	9.17	9.27	9.30	9.47	-1.50
A_09_P055126	FBtr0076078	39336	CG5946	12.28	11.98	12.08	11.82	11.06	10.83	11.31	11.60	-0.84
A_09_P055131	FBtr0110780	39337	CG11597	6.74	7.09	7.09	7.29	7.94	8.03	7.73	7.64	0.78
A_09_P055186	FBtr0076067	39352	CG5883	11.92	11.76	11.70	12.02	11.35	10.57	10.80	10.51	-1.05
A_09_P055196	FBtr0076042	39354	CG17826	15.83	15.85	16.02	15.99	14.63	14.83	14.82	14.92	-1.12
A_09_P055221	FBtr0332816	39360	CG43896	13.69	13.76	13.85	13.90	13.58	13.70	13.55	13.61	-0.19
A_09_P055226	FBtr0332817	39360	CG43896	7.76	8.25	8.09	8.25	6.77	7.12	6.85	7.13	-1.12
A_09_P055241	FBtr0076062	39364	viaf	12.69	12.76	12.88	12.64	12.31	12.00	12.07	12.00	-0.65
A_09_P055266	FBtr0075992	39375	ssp	8.70	8.70	8.84	8.69	7.99	8.08	8.14	8.25	-0.62
A_09_P055271	FBtr0075993	39376	CG11560	9.38	9.50	9.55	9.64	8.90	8.94	8.97	9.12	-0.54
A_09_P055346	FBtr0076009	39403	CG4300	12.91	12.75	12.73	12.72	12.23	12.34	12.15	12.26	-0.53
A_09_P055376	FBtr0075985	39414	Smyd4	12.18	11.83	12.07	11.72	11.38	11.43	11.35	11.25	-0.60
A_09_P055386	FBtr0075956	39420	CG10616	10.23	10.40	10.12	10.37	9.70	9.41	9.76	10.06	-0.55
A_09_P055411	FBtr0089322	39424	CG10638	4.60	4.50	4.31	4.52	6.00	6.41	6.47	6.82	1.94
A_09_P055421	FBtr0075972	39426	CG10646	9.35	9.36	9.31	9.34	8.74	8.82	9.05	9.21	-0.39
A_09_P055431	FBtr0075960	39432	ver	8.99	9.53	9.42	9.42	10.69	10.97	10.96	11.21	1.62
A_09_P055446	FBtr0075961	39436	CG10688	12.44	12.82	12.59	12.79	11.81	11.61	11.81	11.95	-0.86
A_09_P055461	FBtr0075921	39446	CG10984	8.27	8.45	8.30	8.37	7.90	7.90	7.75	7.63	-0.55
A_09_P055471	FBtr0075949	39450	Hip1	9.58	9.64	9.47	9.34	11.05	10.58	10.44	10.17	1.05
A_09_P055516	FBtr0075935	39461	Ent3	6.41	7.04	6.43	6.41	7.76	7.94	7.34	7.46	1.06
A_09_P055521	FBtr0075936	39462	CG10943	10.40	9.72	9.68	9.43	12.81	12.19	12.17	11.65	2.40
A_09_P055526	FBtr0075868	39463	CG14120	12.80	11.40	12.03	11.63	14.78	14.71	14.36	13.81	2.45
A_09_P055571	FBtr0332074	39473	CG14117	8.45	8.52	8.57	8.47	7.81	7.89	8.01	8.11	-0.55
A_09_P055581	FBtr0075898	39475	MICAL-like	9.26	9.42	9.34	9.37	9.86	9.88	9.71	9.71	0.44
A_09_P055596	FBtr0075876	39478	ste14	8.27	8.20	8.21	8.08	8.64	8.68	8.46	8.51	0.39
A_09_P055611	FBtr0075883	39483	SRm160	8.51	8.91	8.70	8.77	7.86	8.13	8.14	8.14	-0.65

A_09_P055686	FBtr0075845	39505	flr	11.88	12.29	12.12	12.15	11.38	11.16	11.32	11.33	-0.81
A_09_P055731	FBtr0075852	39513	CG10732	12.66	12.87	13.02	13.05	12.44	12.25	12.36	12.26	-0.57
A_09_P055736	FBtr0075825	39514	CG10133	9.99	9.60	9.92	9.76	10.44	10.52	10.33	10.22	0.56
A_09_P055741	FBtr0075824	39515	CG10116	11.41	11.20	11.14	11.31	11.84	12.01	11.71	11.49	0.50
A_09_P055791	FBtr0330356	39527	bru-3	10.00	9.78	9.74	9.83	11.09	11.07	10.84	10.64	1.07
A_09_P055811	FBtr0075792	39535	CG8833	8.19	8.11	8.14	8.21	7.85	7.54	7.73	7.62	-0.48
A_09_P055841	FBtr0075767	39548	CG17361	8.04	8.33	8.12	8.36	7.35	7.65	7.54	7.90	-0.60
A_09_P055846	FBtr0075787	39549	CG17359	7.66	7.67	7.55	7.58	6.87	6.87	7.13	7.37	-0.55
A_09_P055861	FBtr0075769	39555	CG6650	9.42	9.92	9.75	9.93	8.40	8.58	8.80	9.15	-1.02
A_09_P055871	FBtr0075774	39559	CG6833	9.44	9.31	9.28	9.20	9.82	9.67	9.72	9.86	0.46
A_09_P055911	FBtr0075744	39577	CG13482	6.13	6.16	5.35	5.29	7.36	6.72	6.94	6.51	1.15
A_09_P055921	FBtr0075729	39580	CG3868	15.19	15.01	14.72	14.73	15.67	15.24	15.51	15.44	0.55
A_09_P055931	FBtr0308596	39583	bbg	11.41	11.78	11.69	11.64	12.61	12.64	12.22	12.23	0.79
A_09_P055956	FBtr0075677	39593	CG9628	8.65	8.41	8.42	8.38	9.27	9.04	9.09	9.06	0.65
A_09_P056016	FBtr0075699	39611	bmm	10.83	10.61	10.63	10.83	12.64	12.14	12.15	11.93	1.49
A_09_P056021	FBtr0075691	39612	CG13472	12.14	12.33	12.29	12.47	11.46	11.50	11.89	12.09	-0.57
A_09_P056061	FBtr0113171	39626	CG10006	8.85	9.39	9.24	9.29	8.39	8.05	8.34	8.41	-0.89
A_09_P056096	FBtr0075664	39635	CG13461	11.05	11.78	11.37	11.74	8.94	9.23	8.89	8.61	-2.57
A_09_P056116	FBtr0305979	3772382	cp309	8.61	8.75	8.66	8.71	8.07	8.19	8.32	8.21	-0.49
A_09_P056141	FBtr0075637	39646	CG13458	7.77	7.51	7.55	7.15	5.86	6.03	6.54	7.03	-1.13
A_09_P056151	FBtr0075655	39648	CG16959	10.30	8.34	9.01	7.88	10.48	10.82	10.37	10.54	1.67
A_09_P056166	FBtr0075640	39652	Pex3	10.51	10.41	10.51	10.56	10.21	10.02	10.16	10.10	-0.38
A_09_P056176	FBtr0075646	39654	Msh6	7.05	7.46	7.49	7.34	6.84	6.39	6.26	6.48	-0.84
A_09_P056191	FBtr0075645	39657	CG7011	11.61	12.15	11.81	12.17	9.91	10.26	10.93	11.39	-1.31
A_09_P056261	FBtr0075548	39670	CG7272	11.67	11.69	11.92	11.59	11.55	11.31	11.35	11.10	-0.39
A_09_P056281	FBtr0075600	39679	CG7945	10.83	11.15	10.98	11.15	10.40	10.29	10.54	10.71	-0.55
A_09_P056296	FBtr0075561	39685	CG7427	8.92	8.78	8.93	8.72	8.04	8.19	8.16	8.26	-0.68
A_09_P056321	FBtr0075565	39690	CG12304	13.76	13.56	13.59	13.61	13.28	13.23	13.37	13.54	-0.27
A_09_P056386	FBtr0075542	39720	fwe	9.07	9.32	9.26	9.17	8.98	8.51	8.90	8.87	-0.39
A_09_P056396	FBtr0075506	39724	CG12713	8.77	8.50	8.81	8.44	8.17	7.95	8.11	8.16	-0.53
A_09_P056421	FBtr0334980	39730	pHCl	5.23	4.52	5.03	4.14	6.19	6.84	5.73	6.25	1.53

A_09_P056426	FBtr0089527	39732	sff	6.92	6.56	6.32	6.36	7.28	7.62	7.40	7.41	0.89
A_09_P056441	FBtr0075511	39736	CG17032	13.23	13.11	12.97	13.04	13.58	13.79	13.92	14.15	0.77
A_09_P056451	FBtr0075512	39738	CG10516	9.87	10.01	10.13	10.22	9.41	9.56	9.64	9.59	-0.51
A_09_P056456	FBtr0075513	39739	CG17026	10.63	11.49	11.13	11.34	9.95	9.78	9.84	9.82	-1.30
A_09_P056481	FBtr0084535	192506	CG5986	10.07	10.12	10.10	10.27	10.83	10.94	10.94	11.02	0.79
A_09_P056496	FBtr0072711	192509	CG12084	11.13	11.26	11.17	11.24	12.10	12.46	11.97	11.84	0.89
A_09_P056501	FBtr0088121	36214	lambdaTry	12.84	11.59	11.64	11.09	13.81	13.75	13.40	13.06	1.71
A_09_P056566	FBtr0075348	39870	PGRP-SB1	7.10	10.03	7.77	7.82	13.25	11.80	12.99	12.92	4.56
A_09_P056616	FBtr0080389	34652	vir-1	10.80	10.68	10.73	10.64	13.91	13.89	13.25	12.52	2.68
A_09_P056656	FBtr0074756	32976	dome	10.95	11.22	11.09	11.05	12.14	12.04	11.84	11.69	0.85
A_09_P056696	FBtr0076371	2768992	Ilp5	7.96	7.79	8.21	8.17	6.47	7.33	7.27	7.20	-0.97
A_09_P056726	FBtr0332818	326184	CG32052	5.63	4.95	4.96	4.64	5.87	6.36	6.19	6.25	1.12
A_09_P056731	FBtr0072970	38344	Atg2	11.77	11.99	11.95	12.11	12.51	12.71	12.32	12.24	0.49
A_09_P056786	FBtr0083312	41995	Pak3	12.57	12.76	12.53	12.66	13.84	13.84	13.43	13.21	0.95
A_09_P056801	FBtr0110874	41839	tefu	8.62	9.18	8.87	9.15	8.34	8.30	8.29	8.41	-0.62
A_09_P056806	FBtr0079562	250710	Proct	8.19	7.93	8.06	8.28	8.69	8.92	8.76	8.57	0.62
A_09_P056811	FBtr0087946	250735	fdl	8.31	8.36	8.40	8.19	7.10	7.41	7.71	7.91	-0.78
A_09_P056971	NM_001104446	43067	CHKov1	7.71	7.99	7.78	7.97	8.05	8.32	8.15	8.27	0.33
A_09_P056976	FBtr0113352	43067	CHKov1	10.79	11.18	11.06	11.28	11.32	11.54	11.48	11.56	0.40
A_09_P057016	FBtr0080711	34894	yuri	10.56	10.02	10.52	10.20	11.43	11.38	11.13	10.62	0.82
A_09_P057046	FBtr0336969	33275	GluRIIC	10.39	10.63	10.70	10.60	10.30	10.31	10.28	10.27	-0.29
A_09_P057101	FBtr0078448	40432	CG7148	11.26	11.24	11.40	11.35	10.88	10.91	10.96	10.99	-0.38
A_09_P057106	FBtr0081183	35162	CG10650	12.63	12.53	12.44	12.39	13.69	13.39	13.32	12.95	0.84
A_09_P057136	FBtr0083996	318725	Takl1	8.40	9.13	8.32	8.47	10.74	10.46	10.28	9.92	1.77
A_09_P057171	FBtr0084047	50050	CG17278	12.64	12.08	12.27	12.15	13.29	13.19	13.11	12.82	0.82
A_09_P057186	FBtr0075560	39683	AGO2	11.79	12.28	11.98	12.24	12.90	12.57	12.66	12.67	0.63
A_09_P057256	FBtr0073106	251645	CG32276	13.95	14.36	14.16	14.31	13.37	13.48	13.65	13.93	-0.59
A_09_P057261	FBtr0075647	251690	CG32147	10.67	10.97	10.82	10.91	10.41	10.41	10.27	10.31	-0.49
A_09_P057326	FBtr0088374	246389	CG30010	9.94	10.17	9.96	10.11	9.39	9.43	9.34	9.64	-0.59
A_09_P057336	FBtr0113356	36147	CG30015	10.48	10.65	10.68	10.80	12.20	12.35	11.72	11.31	1.25
A_09_P057341	FBtr0088241	246393	CG30016	11.17	11.42	11.58	11.76	9.73	9.71	10.18	10.40	-1.48

A_09_P057376	FBtr0300922	246399	CG30026	7.14	5.75	6.77	5.85	9.70	9.27	9.02	8.25	**2.68**
A_09_P057426	FBtr0113358	36371	s-cup	6.32	5.68	6.05	5.45	6.58	6.74	6.59	6.80	**0.80**
A_09_P057496	FBtr0087715	246425	CG30059	8.05	8.39	7.95	8.14	7.02	7.26	7.26	7.46	**-0.88**
A_09_P057581	FBtr0087306	246445	Rif1	10.95	10.89	10.96	10.92	10.03	10.07	10.27	10.49	**-0.71**
A_09_P057606	FBtr0087277	246449	CG30091	11.74	12.32	11.92	12.29	12.89	12.56	12.64	12.57	**0.60**
A_09_P057656	FBtr0086928	246458	NT5E-2	9.65	10.92	10.04	10.92	8.65	9.05	8.79	8.66	**-1.59**
A_09_P057696	FBtr0310055	37134	GEFmeso	9.85	10.16	9.77	9.98	11.12	11.20	10.71	10.34	**0.91**
A_09_P057701	FBtr0113361	37119	CG30118	11.50	11.64	11.90	11.35	12.71	12.82	12.09	12.24	**0.87**
A_09_P057756	FBtr0086269	246484	CG30151	10.11	10.38	10.24	10.37	10.95	10.76	11.10	11.39	**0.77**
A_09_P057761	FBtr0086215	246487	CG30154	7.83	8.92	8.75	8.98	7.24	7.63	7.17	6.98	**-1.36**
A_09_P057781	FBtr0089761	35611	CG30159	13.09	13.28	13.28	13.28	13.08	12.86	12.90	12.86	**-0.31**
A_09_P057811	FBtr0072302	37916	Brca2	8.73	9.00	8.77	8.89	8.39	8.28	8.43	8.65	**-0.41**
A_09_P057866	FBtr0304796	246509	CG30187	9.94	10.24	10.30	10.43	8.46	8.73	9.04	9.34	**-1.34**
A_09_P057871	FBtr0302770	37643	CG42678	12.49	12.83	12.77	12.84	12.32	12.13	12.07	11.94	**-0.62**
A_09_P057876	FBtr0302770	37643	CG42678	12.63	12.98	12.94	12.91	12.36	12.23	12.14	12.04	**-0.67**
A_09_P057921	FBtr0306908	246515	CG43325	13.38	13.39	13.56	13.47	13.05	12.94	12.95	12.94	**-0.48**
A_09_P057996	FBtr0071779	37543	Oatp58Da	9.08	9.65	9.58	9.78	7.66	7.69	8.43	8.59	**-1.43**
A_09_P058046	FBtr0113367	246531	CG30288	8.80	10.50	9.84	10.67	5.15	5.91	6.69	7.00	**-3.77**
A_09_P058051	FBtr0071697	246532	CG30289	8.71	8.81	8.66	8.48	6.66	6.99	7.50	7.77	**-1.43**
A_09_P058076	FBtr0071528	37384	RIC-3	12.48	12.89	12.79	13.02	12.06	11.71	12.02	12.04	**-0.83**
A_09_P058156	FBtr0088702	246560	CG30354	6.85	6.97	7.27	7.13	6.48	6.36	6.20	6.28	**-0.73**
A_09_P058186	FBtr0273325	35832	mtt	5.76	4.69	5.28	4.64	6.12	6.93	6.43	6.44	**1.39**
A_09_P058266	FBtr0113374	246580	CG30381	8.32	8.36	8.20	8.38	7.68	7.86	7.97	8.15	**-0.40**
A_09_P058346	FBtr0071747	246596	Tango11	11.20	11.34	11.29	11.26	10.95	10.81	10.92	10.93	**-0.37**
A_09_P058356	FBtr0301852	246599	Rpi	14.26	14.55	14.57	14.67	13.30	13.61	13.82	14.03	**-0.82**
A_09_P058371	FBtr0072040	246601	CG30413	7.34	6.17	6.96	5.97	8.24	7.54	8.02	7.80	**1.29**
A_09_P058381	FBtr0072031	246602	CG30415	14.28	14.25	14.36	14.29	13.83	13.99	13.94	14.12	**-0.32**
A_09_P058396	FBtr0072263	37882	nord	7.30	7.80	7.49	7.61	8.06	8.09	7.98	8.23	**0.54**
A_09_P058446	FBtr0085991	35549	CG30431	9.15	8.98	9.06	9.12	8.14	8.45	8.37	8.55	**-0.70**
A_09_P058486	FBtr0086143	35573	Tdc1	11.18	10.06	10.83	10.44	13.12	12.99	12.85	12.59	**2.26**
A_09_P058556	FBtr0087334	36719	CG30467	10.16	10.32	10.25	10.33	9.83	9.77	9.81	9.92	**-0.43**

A_09_P058636	FBtr0089419	246648	Cyp12d1-p	10.00	11.07	10.34	11.16	8.90	9.06	9.41	9.65	**-1.38**
A_09_P058646	FBtr0088919	35704	CG30491	10.27	10.02	10.14	10.13	9.89	9.69	9.82	9.87	**-0.32**
A_09_P058671	FBtr0088926	246652	CG30496	8.68	8.59	8.62	8.53	7.86	7.96	8.25	8.47	**-0.47**
A_09_P058681	FBtr0088954	35692	CG30499	12.25	12.28	12.31	12.34	11.73	11.97	11.99	12.21	**-0.32**
A_09_P058686	FBtr0300959	35670	fa2h	10.48	9.72	10.17	9.82	11.97	11.44	11.45	11.17	**1.46**
A_09_P058691	FBtr0089003	246656	CG30503	11.24	11.11	11.22	11.34	12.08	11.97	11.78	11.68	**0.65**
A_09_P058701	FBtr0085779	43688	CG31004	13.15	12.79	12.88	12.63	14.44	14.13	14.07	13.74	**1.23**
A_09_P058781	FBtr0085579	318563	CG31030	8.48	8.21	8.48	8.26	8.81	8.95	8.87	8.87	**0.52**
A_09_P058791	FBtr0085501	43539	CG31036	7.64	6.77	7.15	6.72	8.12	8.38	8.23	7.95	**1.10**
A_09_P058816	FBtr0085470	318567	CG31041	10.39	9.11	9.86	9.81	14.47	13.96	13.67	12.77	**3.93**
A_09_P058861	FBtr0085197	326116	tau	8.16	8.57	8.45	8.57	7.25	7.06	7.18	7.17	**-1.28**
A_09_P058901	FBtr0085080	43244	CG31075	13.17	14.80	14.17	14.64	12.13	11.68	12.42	12.70	**-1.96**
A_09_P058941	FBtr0084989	43179	CG31086	9.78	8.99	8.84	8.75	10.38	10.37	9.99	9.79	**1.05**
A_09_P058946	FBtr0084855	43062	CG31087	12.11	11.67	11.85	11.82	11.65	11.07	11.23	10.95	**-0.64**
A_09_P058981	FBtr0290052	43058	CG31097	7.72	7.91	7.77	7.77	6.75	6.80	7.43	7.51	**-0.67**
A_09_P059001	FBtr0084862	326118	CG31102	9.36	9.37	9.33	9.19	11.33	11.53	11.07	10.77	**1.86**
A_09_P059036	FBtr0084791	43001	CG31109	9.36	9.38	9.49	9.56	9.73	9.78	9.80	9.87	**0.35**
A_09_P059061	FBtr0084736	42960	RabX4	8.42	8.52	8.58	8.70	10.10	10.37	9.95	9.48	**1.42**
A_09_P059081	FBtr0084723	42975	CG31121	10.31	9.98	9.93	10.08	10.58	10.90	10.97	11.19	**0.83**
A_09_P059096	FBtr0084663	326121	CG31126	11.96	11.48	11.70	11.52	10.91	10.84	11.22	11.38	**-0.58**
A_09_P059156	FBtr0083189	41921	cv-d	12.72	12.74	12.60	12.53	11.36	11.53	11.85	12.21	**-0.91**
A_09_P059166	FBtr0083137	261631	Rpb7	11.33	11.35	11.30	11.31	10.64	10.63	10.91	11.11	**-0.50**
A_09_P059176	FBtr0334519	318608	CG31157	7.19	8.88	8.26	8.45	3.04	3.23	4.74	5.63	**-4.03**
A_09_P059236	FBtr0084505	42842	LSm3	12.13	12.04	12.07	12.04	11.15	11.22	11.63	11.85	**-0.61**
A_09_P059266	FBtr0084128	318622	CG31198	15.04	14.68	14.74	14.69	15.69	15.64	15.57	15.46	**0.80**
A_09_P059281	FBtr0110837	318623	GluRIIE	9.94	10.25	10.06	10.29	9.45	9.33	9.52	9.50	**-0.69**
A_09_P059291	FBtr0305058	42455	CG42322	7.66	6.94	7.18	6.98	8.00	8.43	8.37	8.42	**1.11**
A_09_P059331	FBtr0083786	42348	Naam	8.20	7.75	7.78	7.69	9.44	9.15	8.91	8.67	**1.19**
A_09_P059336	FBtr0083353	42032	modSP	9.96	10.14	9.95	9.99	11.26	11.64	11.14	10.82	**1.21**
A_09_P059356	FBtr0304686	42269	CG42613	5.54	4.93	4.49	4.20	6.62	6.76	5.99	6.43	**1.66**
A_09_P059366	FBtr0083691	42237	CG31224	9.26	9.21	9.23	9.24	8.90	9.03	9.00	8.95	**-0.26**

A_09_P059381	FBtr0083663	318636	CG31229	8.77	8.67	8.35	8.35	7.53	7.24	7.32	8.14	-0.98
A_09_P059391	FBtr0334146	14462485	koko	9.47	9.31	9.27	9.38	8.69	8.53	8.97	9.20	-0.51
A_09_P059396	FBtr0084127	42557	CG31233	14.56	14.09	13.84	13.52	15.50	15.36	15.15	14.73	1.18
A_09_P059431	FBtr0083483	42129	CG31249	9.54	9.47	9.59	9.50	8.84	8.68	8.93	9.02	-0.66
A_09_P059481	FBtr0113401	318653	CG31269	11.09	11.65	11.72	11.78	9.96	10.23	10.00	9.98	-1.52
A_09_P059486	FBtr0082312	326130	CG31272	11.80	12.04	11.98	12.06	12.49	12.67	12.35	12.29	0.48
A_09_P059516	FBtr0299516	42632	lqfR	6.49	6.50	6.48	6.17	5.48	5.62	5.82	5.89	-0.71
A_09_P059531	FBtr0084860	318664	CG31288	12.56	11.83	12.13	11.83	13.39	13.43	13.33	13.18	1.24
A_09_P059566	FBtr0304827	41880	Rbp	8.15	7.86	7.83	7.50	8.71	8.95	8.42	8.83	0.89
A_09_P059586	FBtr0301499	3772109	Meltrin	6.55	6.15	6.33	5.98	8.25	8.26	7.86	7.56	1.73
A_09_P059616	FBtr0084985	43177	CG31324	7.94	7.23	7.58	7.00	9.79	10.27	9.58	9.67	2.39
A_09_P059686	FBtr0083480	318698	CG31360	9.04	8.91	9.07	9.07	8.12	8.03	8.38	8.62	-0.73
A_09_P059716	FBtr0301042	41437	CG31368	11.04	11.13	11.06	11.25	10.71	10.73	10.81	10.97	-0.32
A_09_P059721	FBtr0290055	43060	CG31370	7.59	7.74	7.43	7.58	9.16	9.04	8.84	8.56	1.32
A_09_P059731	FBtr0082318	318700	CG31373	11.64	12.51	12.31	12.62	10.97	10.72	10.60	10.47	-1.58
A_09_P059736	FBtr0082399	41377	sals	10.02	10.83	10.45	10.62	9.68	9.58	9.37	9.27	-1.01
A_09_P059761	FBtr0082311	41288	MED7	9.17	9.29	9.05	9.25	8.62	8.65	8.85	8.86	-0.44
A_09_P059771	FBtr0303074	318709	CG42726	7.90	8.25	7.93	8.14	7.20	7.68	7.66	7.74	-0.48
A_09_P059801	FBtr0113405	318721	CG31414	10.21	10.44	10.41	10.73	9.25	9.95	9.80	9.97	-0.70
A_09_P059831	FBtr0113407	43464	CG31427	6.61	3.35	5.69	4.53	8.79	8.92	9.71	10.24	4.37
A_09_P059836	FBtr0084137	326138	CG31431	10.51	10.33	10.44	10.24	11.63	11.51	11.30	11.09	1.00
A_09_P059841	FBtr0084830	318734	CG31436	9.80	9.63	9.50	9.60	10.83	10.67	10.68	10.69	1.08
A_09_P059856	FBtr0084845	318737	Muc96D	14.48	14.62	14.75	14.88	13.36	13.66	13.46	13.21	-1.26
A_09_P059861	FBtr0082355	326139	CG31441	8.90	8.92	8.99	8.99	8.42	8.63	8.67	8.80	-0.32
A_09_P059866	FBtr0085401	326140	CG31445	8.17	7.01	7.86	7.25	8.84	9.04	9.12	9.22	1.48
A_09_P059881	NR_048473	12798523	CR31451	10.95	10.97	11.02	10.90	11.89	11.87	11.68	11.75	0.84
A_09_P059891	FBtr0081870	318744	CG31454	9.41	8.56	8.91	8.12	10.99	10.59	10.37	9.69	1.66
A_09_P059921	FBtr0081831	40978	CG31463	11.57	10.41	10.63	10.17	13.37	13.11	12.75	12.26	2.18
A_09_P059941	FBtr0082844	261628	CG31469	7.74	8.77	8.40	8.80	5.50	5.60	5.42	4.90	-3.07
A_09_P059981	FBtr0082882	261626	CG31495	7.04	7.49	7.15	7.36	5.35	5.59	5.78	6.01	-1.58
A_09_P060026	FBtr0078937	326148	CG31523	13.15	12.33	12.75	12.26	13.45	13.30	13.36	13.43	0.77

A_09_P060051	FBtr0334965	40590	CG44098	11.63	11.80	11.85	11.68	11.26	11.18	11.41	11.57	-0.39
A_09_P060066	FBtr0308857	40583	CG43427	9.18	8.97	9.12	9.06	9.31	9.50	9.32	9.30	0.27
A_09_P060111	FBtr0078736	318794	CG31548	12.33	12.42	12.52	12.58	12.13	12.06	12.14	12.29	-0.31
A_09_P060116	FBtr0078707	40689	CG31549	12.70	12.63	12.77	12.78	12.88	12.84	12.89	12.93	0.17
A_09_P060136	FBtr0308953	40721	CG11000	7.09	6.68	6.91	6.69	7.54	7.74	7.60	7.73	0.81
A_09_P060216	NM_001032121	3772282	His2A:CG33808	7.08	6.83	7.04	7.05	6.34	6.06	5.97	5.83	-0.95
A_09_P060226	FBtr0110959	35424	CG31619	6.82	6.86	6.87	6.24	7.39	7.77	7.08	7.46	0.73
A_09_P060256	FBtr0079325	33931	CG31633	9.36	8.98	9.41	9.15	10.38	10.24	10.00	9.70	0.85
A_09_P060286	FBtr0079262	33910	CG31638	8.58	8.97	8.64	8.82	8.23	8.31	8.38	8.41	-0.42
A_09_P060346	FBtr0077388	33690	pog	8.73	8.62	8.62	8.43	9.74	10.04	9.23	9.39	1.00
A_09_P060356	FBtr0077893	33363	CG31663	10.54	10.67	10.87	10.82	9.75	9.76	9.82	9.87	-0.92
A_09_P060366	FBtr0089786	33361	wry	8.13	7.60	7.66	7.45	8.68	9.00	8.67	8.62	1.03
A_09_P060381	FBtr0081422	35348	CG31673	13.11	13.11	13.26	13.14	12.43	12.22	12.56	12.65	-0.69
A_09_P060436	FBtr0335159	33405	CG4267	6.84	6.31	6.41	6.11	8.70	8.67	8.01	7.45	1.79
A_09_P060451	FBtr0301682	33455	CG31690	6.72	7.23	6.90	7.00	7.66	7.91	7.49	7.42	0.66
A_09_P060496	FBtr0080241	261621	CG31706	8.91	8.91	8.79	9.01	9.36	9.77	9.31	9.27	0.52
A_09_P060526	FBtr0079895	318908	Apf	10.90	10.97	10.98	11.09	10.57	10.65	10.75	10.87	-0.27
A_09_P060531	FBtr0079996	318909	CG31715	11.49	11.49	11.48	11.56	11.03	11.02	11.15	11.32	-0.38
A_09_P060541	FBtr0085269	43348	CG4980	12.25	12.36	12.34	12.45	11.46	11.49	11.68	11.86	-0.73
A_09_P060561	FBtr0085254	43354	Gp93	15.88	15.88	16.02	15.83	15.11	14.91	15.51	15.75	-0.59
A_09_P060571	FBtr0085255	43356	CG5527	11.51	10.68	11.18	10.70	12.09	11.86	11.84	11.67	0.85
A_09_P060581	FBtr0085257	43358	CG4849	11.90	11.74	11.73	11.77	11.41	11.27	11.56	11.56	-0.33
A_09_P060586	FBtr0085260	43360	CG4815	4.97	5.45	5.14	5.38	3.29	3.27	2.81	3.66	-1.98
A_09_P060606	FBtr0085297	43373	Gfat2	12.65	12.86	12.95	13.19	12.35	12.32	12.48	12.49	-0.51
A_09_P060646	FBtr0085305	43388	CG9988	8.67	8.36	8.83	8.52	7.21	7.73	7.09	6.78	-1.39
A_09_P060656	FBtr0085307	43390	CG9989	8.44	8.20	7.48	6.84	9.42	9.44	9.56	9.66	1.78
A_09_P060706	FBtr0085326	43401	CG1647	8.83	9.09	9.00	8.99	8.10	8.35	8.43	8.70	-0.58
A_09_P060711	FBtr0085328	43408	CG14529	9.16	8.86	9.09	8.77	10.37	10.17	10.02	9.64	1.08
A_09_P060730	FBtr0085330	43412	CG14527	8.27	7.88	7.82	7.62	5.02	4.95	6.59	7.30	-1.93
A_09_P060811	FBtr0085416	43436	alpha-Man-Ib	10.92	11.30	11.15	11.08	9.93	10.04	10.27	10.64	-0.89
A_09_P060816	FBtr0085368	43437	CG11876	12.13	11.66	11.93	11.69	11.29	11.14	11.46	11.53	-0.50

A_09_P060836	FBtr0085414	43441	dgt6	7.39	7.97	7.85	8.08	7.45	7.04	7.01	6.97	-0.71
A_09_P060841	FBtr0085413	43443	CG14512	9.51	9.20	9.31	9.27	8.90	9.00	9.04	9.05	-0.32
A_09_P060856	FBtr0085411	43446	CG11882	9.18	10.00	9.58	9.86	8.78	9.00	8.80	8.82	-0.80
A_09_P060861	FBtr0085387	43450	CG11897	8.90	8.61	8.54	8.38	11.76	11.94	11.29	10.90	2.86
A_09_P060871	FBtr0113304	43453	CG14509	9.16	8.74	9.23	8.75	9.94	10.37	9.74	10.21	1.09
A_09_P060881	FBtr0085407	43456	Mesh1	10.33	10.31	10.37	10.42	9.17	9.34	9.62	9.98	-0.83
A_09_P060931	FBtr0085427	43475	CG2010	13.67	13.56	13.79	13.58	13.14	13.10	13.13	13.24	-0.50
A_09_P060946	FBtr0085472	43479	CG7567	13.28	13.69	13.72	13.64	13.21	12.01	12.67	11.96	-1.12
A_09_P060951	FBtr0085471	43480	CG11470	11.28	11.96	11.67	11.67	9.65	8.59	9.35	9.38	-2.41
A_09_P060961	FBtr0085447	43483	CG1907	14.33	14.51	14.52	14.51	14.05	13.98	14.10	14.20	-0.38
A_09_P060996	FBtr0085464	43492	CG7582	10.28	9.87	9.86	9.84	9.30	9.08	9.37	9.45	-0.66
A_09_P061016	FBtr0085462	43497	Obp99b	15.89	16.47	16.29	16.48	8.96	9.20	10.42	10.90	-6.41
A_09_P061021	FBtr0085456	43498	CG15506	10.64	8.85	9.80	8.95	11.39	11.71	11.34	11.19	1.85
A_09_P061046	FBtr0085476	43506	IntS11	11.83	11.66	11.75	11.74	11.95	12.07	12.00	11.99	0.26
A_09_P061081	FBtr0085533	43521	Vps16B	8.94	9.10	9.00	9.32	8.55	8.65	8.64	8.89	-0.41
A_09_P061096	FBtr0085528	326115	CG31033	10.27	10.54	10.49	10.51	10.05	9.88	9.95	10.00	-0.48
A_09_P061116	FBtr0089927	43530	CG7824	9.18	9.26	9.19	9.33	10.10	10.11	9.95	9.79	0.74
A_09_P061131	FBtr0089925	43533	CG7816	10.27	10.56	10.12	10.34	11.93	11.73	11.42	11.17	1.24
A_09_P061176	FBtr0085608	43554	CG7903	9.91	10.17	10.00	10.10	9.44	9.37	9.54	9.63	-0.56
A_09_P061241	FBtr0085559	43574	CG7943	11.80	11.80	11.96	11.81	11.70	11.70	11.52	11.53	-0.23
A_09_P061251	FBtr0085561	43576	CG7946	11.35	11.70	11.58	11.73	10.96	10.97	11.22	11.38	-0.46
A_09_P061261	FBtr0085564	43579	CG7950	9.91	9.26	9.68	9.27	10.22	10.39	10.31	10.38	0.79
A_09_P061306	FBtr0085571	43593	CG9743	13.52	12.87	13.25	13.05	14.30	14.28	14.17	14.08	1.03
A_09_P061311	FBtr0089423	43594	RpS7	16.42	16.43	16.38	16.44	16.47	16.47	16.47	16.49	0.06
A_09_P061336	FBtr0085618	43603	CG18404	5.13	4.66	4.47	4.69	7.25	6.78	6.78	6.56	2.10
A_09_P061361	NM_143534	43612	CG15533	11.96	10.69	11.27	10.45	13.70	13.00	13.12	12.80	2.06
A_09_P061371	FBtr0113310	43614	CG15537	6.07	5.43	6.03	5.31	6.67	6.86	6.63	6.92	1.06
A_09_P061381	FBtr0331358	43617	CG2224	8.49	8.68	8.54	8.64	8.32	8.23	8.23	8.27	-0.32
A_09_P061386	FBtr0085649	43618	CDase	10.02	8.98	9.43	8.84	12.07	11.87	11.38	10.83	2.22
A_09_P061391	FBtr0085650	43620	PH4alphaEFB	13.35	13.51	13.48	13.44	11.48	12.13	12.49	12.95	-1.18
A_09_P061401	FBtr0085652	43622	Jon99Fi	14.90	14.45	14.01	14.64	16.02	15.99	15.95	15.76	1.43

A_09_P061441	FBtr0085679	43636	CG9717	9.95	8.96	9.83	9.13	10.82	10.85	10.54	10.36	1.17
A_09_P061471	FBtr0335452	43646	CG11313	10.24	11.06	10.91	11.30	8.73	8.28	9.09	9.25	-2.04
A_09_P061481	FBtr0085723	43648	Npc2g	13.89	14.22	14.13	14.30	12.38	12.54	13.02	13.23	-1.34
A_09_P061491	FBtr0085703	43652	dj-1beta	14.42	14.55	14.42	14.53	13.71	13.69	13.91	14.07	-0.63
A_09_P061546	FBtr0290201	43677	CG15553	6.72	6.46	5.77	5.99	7.59	8.00	7.89	8.16	1.68
A_09_P061551	FBtr0085739	43678	CG11318	8.15	7.32	7.28	7.07	9.18	8.97	8.73	8.37	1.36
A_09_P061566	FBtr0085737	43681	CG15556	8.49	8.85	8.81	8.87	7.46	7.39	7.47	7.51	-1.30
A_09_P061596	FBtr0113312	43694	CG12054	12.02	12.35	12.27	12.31	12.95	13.21	12.84	12.59	0.66
A_09_P061636	FBtr0085785	43707	CG1607	11.20	11.24	11.13	11.19	10.94	11.03	10.89	10.92	-0.24
A_09_P061651	FBtr0085808	43713	CG11334	12.85	13.03	12.90	13.04	12.15	12.43	12.39	12.50	-0.59
A_09_P061671	FBtr0085796	43720	CG1635	11.42	10.98	11.26	11.09	10.58	10.51	10.82	10.90	-0.48
A_09_P061696	FBtr0085868	43726	CG11539	9.09	9.06	9.05	8.98	8.34	8.27	8.51	8.79	-0.57
A_09_P061701	FBtr0085819	43727	CG1792	9.64	9.63	9.78	9.77	9.19	9.34	9.37	9.35	-0.40
A_09_P061706	FBtr0085820	43728	pasha	9.96	10.07	10.04	10.12	9.76	9.69	9.63	9.74	-0.34
A_09_P061721	FBtr0085867	43731	CG11550	7.15	8.64	8.04	8.71	5.11	6.09	6.42	6.79	-2.03
A_09_P061726	FBtr0085865	43734	CstF-50	10.68	10.67	10.68	10.66	9.57	9.65	10.00	10.30	-0.79
A_09_P061741	FBtr0085837	43738	CG1896	10.46	10.65	10.63	10.74	9.76	9.88	9.95	10.13	-0.69
A_09_P061746	FBtr0300915	43742	salt	12.73	12.66	12.67	12.86	12.61	12.40	12.43	12.42	-0.27
A_09_P061751	FBtr0085860	43744	Smvt	11.71	11.79	11.80	12.00	11.23	10.40	10.96	11.03	-0.92
A_09_P061756	FBtr0085858	43746	CG2135	9.79	9.80	9.69	9.71	9.35	9.03	9.03	9.47	-0.53
A_09_P061761	FBtr0085842	43747	sip3	11.97	12.14	12.16	12.22	11.01	11.21	11.34	11.64	-0.82
A_09_P061771	FBtr0085856	43750	CG2118	11.93	12.21	12.10	12.22	11.25	11.31	11.44	11.71	-0.69
A_09_P061796	FBtr0085852	43756	CG11576	10.01	10.10	9.95	9.96	9.57	9.75	9.48	9.59	-0.41
A_09_P061801	FBtr0302181	43758	RhoGAP100F	9.18	9.11	8.70	8.82	9.72	10.12	9.55	9.94	0.88
A_09_P061831	FBtr0089116	43780	CG1674	13.17	13.91	13.46	13.74	12.28	11.93	12.44	12.86	-1.20
A_09_P061836	FBtr0308087	43780	CG1674	6.77	6.95	7.01	6.76	5.79	5.95	5.91	6.22	-0.91
A_09_P061841	FBtr0089119	43780	CG1674	8.34	8.62	8.33	8.36	5.72	5.39	6.40	6.82	-2.33
A_09_P061846	FBtr0299722	43781	RhoGAP102A	5.66	6.25	6.23	5.94	4.53	4.98	4.73	5.15	-1.17
A_09_P061851	FBtr0089139	43783	Syt7	8.72	8.42	8.36	8.46	9.06	9.18	9.14	9.30	0.68
A_09_P061871	FBtr0089111	43791	lgs	10.57	10.82	10.95	10.95	10.36	10.24	10.34	10.22	-0.53
A_09_P061876	FBtr0332906	43794	Asator	10.10	10.43	10.36	10.35	9.86	9.85	9.93	10.12	-0.37

A_09_P061886	FBtr0089079	43800	CG1909	7.48	7.04	7.07	6.90	8.35	8.42	8.17	8.01	1.12
A_09_P061896	FBtr0089103	43804	mav	11.06	11.13	11.23	11.29	10.45	10.62	10.67	10.50	-0.62
A_09_P061916	FBtr0089254	43816	MED26	10.88	11.00	10.85	10.77	10.73	10.70	10.64	10.67	-0.19
A_09_P061931	FBtr0089195	43822	CG11155	8.32	9.04	8.97	8.97	8.10	7.55	7.68	7.43	-1.14
A_09_P061936	FBtr0089194	43822	CG11155	6.04	6.69	6.36	6.31	5.54	4.79	5.29	5.69	-1.02
A_09_P061941	FBtr0089195	43822	CG11155	11.48	12.30	12.07	12.29	11.47	11.23	11.25	11.12	-0.77
A_09_P061946	FBtr0089207	43824	Cals	11.73	11.66	11.60	11.72	11.05	10.76	11.30	11.48	-0.53
A_09_P061956	FBtr0089222	43831	CG11077	10.59	10.44	10.55	10.54	11.35	11.14	11.17	11.14	0.67
A_09_P061961	FBtr0334625	43835	CORL	4.81	5.02	4.90	4.93	6.81	6.69	6.19	5.75	1.45
A_09_P061966	FBtr0300885	43842	CG11148	12.62	12.74	12.59	12.80	13.05	13.06	13.00	12.82	0.29
A_09_P061976	FBtr0089252	43844	Sox102F	5.55	5.75	5.43	5.60	4.09	4.70	4.27	3.93	-1.33
A_09_P061996	FBtr0309316	3354988	CG17163	7.68	8.23	7.93	8.04	6.87	7.31	7.23	7.55	-0.73
A_09_P062001	FBtr0113730	3354996	CG17168	11.25	11.40	11.27	11.29	10.94	10.94	11.00	11.07	-0.31
A_09_P062036	FBtr0113704	3355094	CG12567	10.41	10.47	10.41	10.49	10.33	10.05	10.08	10.10	-0.30
A_09_P062056	FBtr0111192	3355158	CG13865	9.92	9.73	10.02	9.53	9.44	9.03	9.34	9.42	-0.49
A_09_P062081	FBtr0086071	49893	CG17508	11.49	11.67	11.42	11.66	12.25	11.82	11.96	11.94	0.43
A_09_P062176	FBtr0111252	3355127	CG17494	11.35	11.43	11.39	11.48	10.99	10.81	11.08	11.17	-0.40
A_09_P062201	FBtr0113768	3354992	CG2893	8.72	9.01	8.82	8.99	8.07	8.15	8.24	8.50	-0.64
A_09_P062301	FBtr0074988	40092	rept	12.24	11.97	12.11	12.19	11.65	11.46	11.84	11.97	-0.40
A_09_P062306	FBtr0091746	3772427	primo-2	10.51	10.41	10.33	10.36	9.37	8.94	9.53	9.67	-1.02
A_09_P062311	FBtr0082226	53439	pont	12.81	12.71	12.75	12.74	12.36	12.28	12.44	12.56	-0.34
A_09_P062326	FBtr0071089	31698	p115	12.74	12.88	12.82	12.90	11.86	11.94	12.37	12.70	-0.61
A_09_P062331	FBtr0074812	53448	meso18E	8.81	8.83	9.05	8.83	7.78	8.21	8.09	8.27	-0.79
A_09_P062371	FBtr0077469	53550	lectin-24Db	8.29	9.16	8.52	9.12	6.76	7.07	7.43	7.79	-1.51
A_09_P062406	FBtr0085877	53554	krz	10.72	10.94	11.02	10.97	9.52	9.64	9.49	9.58	-1.35
A_09_P062486	FBtr0077084	38628	bc10	6.87	7.20	7.03	6.94	6.32	6.58	6.36	6.48	-0.57
A_09_P062496	FBtr0082378	53502	Ugt86Di	4.75	3.91	3.31	NA	7.48	7.18	7.66	7.99	3.59
A_09_P062521	FBtr0082337	53508	Ugt86Dc	6.44	4.30	4.89	4.33	8.32	7.82	8.24	8.15	3.14
A_09_P062526	FBtr0082338	53510	Ugt86Da	12.01	11.25	11.43	11.12	12.58	12.40	12.35	12.03	0.88
A_09_P062531	FBtr0080912	53511	Ugt36Bc	13.95	13.66	13.75	13.51	13.19	13.21	13.21	13.30	-0.49
A_09_P062546	FBtr0081246	35236	Top3alpha	10.02	10.20	10.15	10.22	10.79	10.73	10.47	10.42	0.45

A_09_P062551	FBtr0083273	53437	Sulf1	6.66	6.23	6.30	5.62	7.35	7.20	7.12	6.78	0.91
A_09_P062556	FBtr0086531	53442	Spt5	11.71	11.92	11.82	11.88	11.41	11.37	11.57	11.70	-0.32
A_09_P062571	FBtr0072627	53472	Aplip1	7.56	7.71	7.57	7.58	7.84	7.89	7.83	8.01	0.29
A_09_P062601	FBtr0089899	53444	SC35	13.38	13.33	13.27	13.37	13.07	12.88	13.06	13.23	-0.28
A_09_P062606	FBtr0076729	53438	RecQ4	9.29	9.16	9.12	9.10	8.55	8.61	8.75	9.02	-0.43
A_09_P062621	FBtr0086086	35486	sxc	9.84	9.72	10.03	9.77	9.41	9.27	9.35	9.21	-0.53
A_09_P062626	FBtr0299779	39621	Ocho	5.71	6.20	5.66	5.75	4.46	4.49	4.71	5.09	-1.14
A_09_P062636	FBtr0077118	38649	Myt1	9.79	9.93	10.00	9.93	9.28	9.10	9.27	9.56	-0.61
A_09_P062641	FBtr0079475	53515	Myo28B1	8.65	8.34	8.29	8.11	9.93	9.30	9.32	9.29	1.11
A_09_P062646	FBtr0079476	53515	Myo28B1	8.40	7.84	8.08	7.61	9.96	9.67	9.28	8.87	1.46
A_09_P062656	FBtr0072983	53577	Jafrac2	11.72	11.81	11.67	11.58	10.99	10.62	11.13	11.42	-0.65
A_09_P062686	FBtr0075050	40034	GNBP1	10.74	11.34	11.04	11.32	10.28	10.45	10.43	10.64	-0.66
A_09_P062691	FBtr0089075	43799	Ephrin	7.09	7.38	7.25	7.04	6.68	6.67	6.46	6.41	-0.63
A_09_P062711	FBtr0070442	31245	Seipin	11.75	11.57	11.60	11.51	10.74	10.81	10.79	10.82	-0.82
A_09_P062726	FBtr0070165	31038	MED22	10.69	10.80	10.69	10.92	10.41	10.53	10.47	10.64	-0.26
A_09_P062731	FBtr0070167	31042	TRAM	14.41	14.61	14.66	14.57	13.19	13.50	13.78	13.99	-0.95
A_09_P062751	FBtr0331303	31037	CG3711	8.67	8.91	8.98	8.78	8.48	8.20	8.59	8.46	-0.40
A_09_P062766	FBtr0070170	31044	mus81	6.61	6.79	6.79	6.38	5.27	5.54	5.95	6.09	-0.93
A_09_P062771	FBtr0070187	31045	CG3703	7.29	7.13	7.01	6.99	7.85	8.27	7.72	7.66	0.77
A_09_P062781	FBtr0070185	31047	CG3690	11.30	12.08	11.83	12.00	11.25	10.97	10.81	10.46	-0.93
A_09_P062846	FBtr0070251	31061	CG11384	6.37	5.24	5.20	3.41	8.15	7.75	7.69	7.51	2.72
A_09_P062912	FBtr0339401	31235	Vha36-3	9.73	9.93	9.90	10.02	9.70	9.43	9.44	9.29	-0.43
A_09_P062951	FBtr0070405	31222	mRpL14	11.49	11.06	11.27	10.98	10.51	10.38	10.61	10.73	-0.64
A_09_P062991	FBtr0070181	31056	CG3655	10.98	10.65	10.73	10.38	11.46	11.48	11.24	11.13	0.64
A_09_P062996	FBtr0070179	31055	CG14629	12.21	12.47	12.06	12.17	10.72	11.14	11.48	12.00	-0.89
A_09_P063001	FBtr0086734	53579	Dip3	8.56	8.66	8.49	8.56	9.11	9.26	8.98	8.83	0.47
A_09_P063011	FBtr0071302	53580	Dip1	8.16	8.07	8.23	8.18	7.18	7.52	7.78	7.97	-0.55
A_09_P063016	FBtr0076413	39136	SH3PX1	11.86	11.98	11.94	11.99	12.41	12.33	12.22	12.05	0.31
A_09_P063021	FBtr0087667	36495	cid	6.58	6.81	6.53	6.76	5.50	5.47	5.97	6.24	-0.88
A_09_P063036	FBtr0344095	41587	grsm	12.29	12.81	12.30	12.46	11.12	11.00	11.66	11.98	-1.03
A_09_P063041	FBtr0082690	41585	Spc25	8.64	9.19	9.05	9.34	8.70	8.23	8.37	8.32	-0.65

A_09_P063046	FBtr0080105	34465	CG17104	13.72	12.21	13.31	12.64	14.76	14.92	14.40	13.81	1.51
A_09_P063061	FBtr0087118	53425	Alk	7.50	7.14	7.02	6.96	8.31	8.54	8.20	8.08	1.13
A_09_P063091	FBtr0075352	39862	zetaCOP	11.20	11.17	11.26	11.24	10.47	10.18	10.85	11.04	-0.58
A_09_P063111	FBtr0111273	3355145	CG12547	10.18	10.41	10.31	10.36	9.88	9.64	9.80	9.87	-0.52
A_09_P063146	FBtr0082006	50007	CG11985	10.13	9.80	9.92	10.07	9.26	9.25	9.48	9.52	-0.60
A_09_P063156	FBtr0082735	50024	CG11686	12.19	11.89	11.80	11.74	12.91	12.65	12.66	12.78	0.85
A_09_P063171	FBtr0083838	50044	CG17193	7.18	6.58	6.63	5.99	7.53	7.88	7.56	7.62	1.05
A_09_P063181	FBtr0083951	50048	CG15922	11.33	11.15	11.27	11.27	10.45	10.62	10.66	10.86	-0.61
A_09_P063246	FBtr0085435	50096	Spase12	13.77	14.00	13.86	14.04	12.50	12.83	13.20	13.64	-0.87
A_09_P063306	FBtr0077323	50123	CG15456	8.55	8.26	8.40	8.49	7.77	7.96	8.02	8.21	-0.43
A_09_P063406	FBtr0077440	50178	CG15434	6.91	6.79	7.27	7.15	6.20	5.01	5.92	5.91	-1.27
A_09_P063416	FBtr0077817	50190	Nplp4	13.57	14.51	14.71	14.71	10.45	12.06	12.47	12.61	-2.48
A_09_P063421	FBtr0077863	50191	CG15353	14.92	15.71	15.52	15.66	13.00	14.03	13.82	14.11	-1.71
A_09_P063431	FBtr0077973	50196	CG5011	10.70	9.84	9.95	9.77	12.27	12.12	11.82	11.41	1.84
A_09_P063441	FBtr0086238	2768858	dpr	6.15	4.86	4.53	5.14	5.97	6.80	6.70	6.81	1.40
A_09_P063446	FBtr0086475	50202	CG15126	12.29	12.07	12.10	12.14	13.97	13.65	13.68	13.20	1.48
A_09_P063471	FBtr0086666	50208	CG16836	6.21	3.39	2.86	3.31	10.60	10.87	9.88	9.05	6.16
A_09_P063476	FBtr0086665	50209	IM3	9.31	5.23	6.26	8.82	13.75	13.83	13.01	11.66	5.66
A_09_P063496	FBtr0087581	50224	CG13018	10.58	10.30	10.49	10.55	9.45	9.91	9.91	9.92	-0.68
A_09_P063506	FBtr0087739	50227	CG17059	11.15	11.34	11.23	11.44	10.74	10.89	10.93	11.10	-0.37
A_09_P063541	FBtr0088396	50246	CoVIIc	13.93	13.88	13.86	14.01	13.29	13.39	13.48	13.61	-0.48
A_09_P063571	FBtr0299563	50259	CG14104	7.24	7.21	7.17	7.18	6.29	6.36	6.63	6.62	-0.73
A_09_P063616	FBtr0113333	2768954	CG12355	8.96	8.21	8.36	8.09	9.76	9.86	9.35	8.92	1.07
A_09_P063656	FBtr0113334	50290	CG14132	11.80	11.59	11.63	11.82	12.45	12.85	12.73	13.07	1.06
A_09_P063661	FBtr0076353	50296	dpr6	6.09	6.00	5.54	5.69	6.55	7.32	6.68	6.74	0.99
A_09_P063671	FBtr0076592	50301	CG13306	7.15	6.74	6.87	6.25	5.37	5.55	5.81	6.21	-1.02
A_09_P063676	FBtr0076699	50305	CG8012	10.67	11.42	11.16	11.26	6.94	6.74	8.93	9.55	-3.09
A_09_P063681	FBtr0332760	8674111	CG42445	9.00	8.97	9.00	9.18	8.11	8.39	8.50	8.65	-0.62
A_09_P063686	FBtr0076918	50310	CG8620	9.88	8.83	9.64	8.83	13.57	14.03	13.40	13.00	4.20
A_09_P063731	FBtr0300214	50363	ksh	8.85	9.11	9.00	9.15	8.23	8.61	8.35	8.64	-0.57
A_09_P063751	FBtr0070390	50373	CG17776	12.36	12.39	12.52	12.52	11.70	12.05	11.93	11.96	-0.54

A_09_P063826	FBtr0071343	50405	CG9034	14.86	14.69	14.96	14.79	14.07	14.21	14.43	14.60	-0.49
A_09_P063856	FBtr0079203	50424	Muc26B	15.01	14.61	14.90	14.86	15.23	15.41	15.42	15.27	0.49
A_09_P063861	FBtr0079403	50428	CG13779	14.39	14.47	14.38	14.43	13.46	13.70	13.98	14.27	-0.57
A_09_P063916	FBtr0080959	50459	CG6115	12.96	13.23	13.20	13.24	13.81	13.77	13.51	13.34	0.45
A_09_P063931	FBtr0081102	50467	CG17325	14.66	14.38	14.52	14.49	16.07	16.08	15.92	15.57	1.40
A_09_P063961	FBtr0111248	3355129	CG17715	9.77	9.75	9.78	9.73	9.26	9.02	9.23	9.45	-0.52
A_09_P063976	FBtr0076168	39270	scyl	7.71	8.47	8.28	7.86	9.64	9.64	8.90	8.77	1.16
A_09_P063991	FBtr0078318	40336	park	8.98	9.09	8.97	8.98	7.91	8.18	8.27	8.46	-0.80
A_09_P064011	FBtr0299534	32561	mmd	6.46	6.11	5.66	5.98	6.49	7.04	7.05	7.02	0.85
A_09_P064021	FBtr0073160	45574	ida	9.82	9.80	9.81	9.80	9.05	8.66	9.14	9.40	-0.75
A_09_P064036	FBtr0075580	39716	comm2	9.86	9.05	8.92	8.74	10.01	9.92	10.20	10.33	0.97
A_09_P064046	FBtr0100641	38427	armi	10.35	10.68	10.53	10.76	10.06	9.67	9.95	9.97	-0.67
A_09_P064056	FBtr0088115	53433	Vhl	9.22	9.56	9.48	9.57	8.81	9.04	9.01	9.15	-0.46
A_09_P064061	FBtr0081254	35248	Tep4	14.67	14.65	14.66	14.65	14.95	15.00	14.91	14.97	0.30
A_09_P064101	FBtr0078693	117332	Rheb	12.85	12.91	12.80	12.97	13.76	13.34	13.49	13.47	0.63
A_09_P064126	FBtr0333333	32278	HDAC4	6.68	6.55	6.41	6.35	8.19	8.48	7.82	7.44	1.48
A_09_P064281	FBtr0305795	36484	AttC	11.28	12.61	10.99	11.34	16.45	16.11	16.37	16.29	4.75
A_09_P064286	FBtr0087438	36637	AttB	11.67	12.74	11.42	12.54	16.19	15.76	16.03	15.92	3.88
A_09_P064321	FBtr0301768	64877	cpx	10.61	10.28	10.52	10.26	11.00	10.60	11.05	11.08	0.51
A_09_P064366	FBtr0080763	34930	Ku80	9.13	8.87	9.06	8.95	10.13	10.16	10.17	10.39	1.21
A_09_P064371	FBtr0071249	31808	Hexo2	11.93	12.50	12.27	12.48	11.53	11.64	11.46	11.37	-0.80
A_09_P064376	FBtr0073237	38528	Hexo1	14.11	14.08	13.84	13.99	14.51	14.54	14.51	14.58	0.53
A_09_P064391	FBtr0301640	40644	7B2	10.88	10.58	10.78	10.74	11.39	11.36	11.44	11.57	0.70
A_09_P064396	FBtr0072902	38294	yellow-g	3.39	3.26	2.86	3.24	5.83	6.51	5.83	5.50	2.73
A_09_P064401	FBtr0082723	41595	yellow-f	9.31	10.76	9.31	9.55	11.41	11.32	11.03	10.74	1.40
A_09_P064406	FBtr0082757	41653	yellow-e	9.39	10.56	9.91	10.47	8.17	8.44	8.15	8.00	-1.89
A_09_P064411	FBtr0072011	37703	yellow-d	12.57	12.19	12.19	12.54	13.38	13.62	13.19	13.00	0.92
A_09_P064416	FBtr0080699	34879	yellow-c	12.40	12.06	12.11	12.11	13.71	13.81	13.29	12.78	1.23
A_09_P064441	FBtr0081214	35215	Pax	15.48	15.62	15.97	15.68	13.83	13.80	14.22	14.53	-1.59
A_09_P064446	FBtr0100449	35215	Pax	9.53	9.26	9.50	9.40	8.97	8.85	8.91	8.70	-0.57
A_09_P064451	FBtr0086126	59261	CG3267	11.72	11.98	11.99	11.98	11.20	11.10	11.25	11.42	-0.68

A_09_P064461	FBtr0086177	59177	Tsp42Eb	13.09	12.95	12.58	12.85	15.13	14.59	14.88	14.65	**1.95**
A_09_P064471	FBtr0082330	41318	Adk3	12.91	12.70	12.67	12.84	12.26	12.47	12.35	12.43	**-0.40**
A_09_P064481	FBtr0114516	59140	CG18744	9.41	8.41	8.56	8.49	11.07	10.87	10.47	9.97	**1.88**
A_09_P064486	FBtr0081777	59141	CG18745	7.98	7.85	7.68	7.39	9.67	9.38	9.11	8.28	**1.38**
A_09_P064501	FBtr0113345	59144	CG18748	8.67	8.45	8.23	8.24	10.50	10.11	9.82	9.46	**1.58**
A_09_P064521	FBtr0076735	59151	mRpL36	12.49	12.04	12.30	12.09	11.63	11.70	11.82	11.85	**-0.48**
A_09_P064526	FBtr0077060	59158	Cpr65Au	9.43	10.30	9.76	10.26	8.34	8.92	8.12	8.10	**-1.57**
A_09_P064566	FBtr0075098	59172	Capr	10.78	11.07	10.88	11.10	10.56	10.67	10.48	10.43	**-0.42**
A_09_P064571	FBtr0088935	59173	CG18812	11.59	11.55	11.52	11.46	12.47	12.44	12.09	11.73	**0.65**
A_09_P064576	FBtr0307504	59175	CG18814	10.01	9.57	9.84	9.73	8.13	8.27	8.79	9.14	**-1.21**
A_09_P064581	FBtr0076103	59176	CG18815	13.36	13.26	13.35	13.13	12.94	12.72	12.96	13.13	**-0.34**
A_09_P064609	FBtr0075313	59215	CG32164	12.37	12.26	12.36	12.31	12.83	12.80	12.59	12.52	**0.36**
A_09_P064631	FBtr0076970	59223	CG18769	6.85	6.76	6.35	6.42	7.57	7.33	7.19	7.25	**0.74**
A_09_P064691	FBtr0085459	59246	CG18731	10.98	11.19	10.88	11.22	10.17	10.36	10.50	10.83	**-0.60**
A_09_P064701	FBtr0075747	39569	Sox21b	8.08	7.94	7.90	7.74	8.28	8.81	8.64	8.63	**0.67**
A_09_P064711	FBtr0299515	42632	lqfR	11.45	11.85	11.43	11.90	11.20	10.85	11.13	11.24	**-0.55**
A_09_P064726	FBtr0089141	43782	Nfl	9.58	9.54	9.69	9.58	7.68	7.64	8.32	8.91	**-1.46**
A_09_P064741	FBtr0072866	117365	HBS1	11.06	11.11	11.18	11.14	11.33	11.46	11.37	11.48	**0.29**
A_09_P064746	FBtr0073642	32166	Chrac-16	9.64	10.26	9.99	10.29	8.94	8.95	9.08	9.15	**-1.01**
A_09_P064751	FBtr0100268	3772329	Chrac-14	10.28	10.18	10.23	10.36	9.95	10.07	10.07	10.07	**-0.22**
A_09_P064756	FBtr0113347	117368	prt	7.28	6.48	7.06	6.51	8.34	8.33	8.18	7.99	**1.38**
A_09_P064766	FBtr0084103	42525	AP-2sigma	11.42	11.35	11.32	11.51	10.60	10.37	10.86	11.12	**-0.66**
A_09_P064781	FBtr0082627	117369	desat1	13.62	14.04	14.03	13.76	13.23	13.19	13.14	13.33	**-0.64**
A_09_P064816	FBtr0071317	31874	ric8a	7.97	8.25	8.03	8.03	7.32	7.56	7.58	7.88	**-0.49**
A_09_P064826	FBtr0071372	326234	l(1)G0320	12.23	12.64	12.51	12.29	11.33	11.11	11.48	12.06	**-0.92**
A_09_P064841	FBtr0071405	31907	l(1)G0232	10.45	10.68	10.49	10.49	11.48	11.53	11.15	11.13	**0.79**
A_09_P064846	FBtr0071438	31950	l(1)G0230	15.30	15.32	15.23	15.29	15.06	15.02	15.20	15.20	**-0.17**
A_09_P064871	FBtr0301374	43997	jbug	11.13	10.90	10.91	10.90	11.68	11.90	11.55	11.43	**0.68**
A_09_P064891	FBtr0082639	41547	hug	8.74	8.58	8.79	8.68	9.14	9.86	9.38	9.45	**0.76**
A_09_P064896	FBtr0080852	34961	heix	10.56	10.69	10.57	10.48	9.68	9.28	9.99	10.29	**-0.76**
A_09_P064911	FBtr0083304	42008	decay	13.13	13.27	13.02	13.36	14.22	14.26	13.88	13.52	**0.78**

A_09_P064936	FBtr0100023	45233	capt	14.85	15.10	15.08	15.03	14.88	14.63	14.69	14.76	-0.28
A_09_P064946	FBtr0079688	34191	CG17834	10.40	10.67	10.56	10.65	11.38	11.23	11.01	10.84	0.54
A_09_P064961	FBtr0077717	33469	Taf10	7.35	7.23	7.25	7.27	6.71	6.44	6.84	7.00	-0.53
A_09_P064971	FBtr0081578	40876	Syt4	8.94	8.79	8.78	8.72	9.32	9.05	9.25	9.42	0.45
A_09_P065026	FBtr0075722	47746	Lk	7.62	7.55	7.75	7.57	8.25	8.41	7.96	8.14	0.57
A_09_P065046	FBtr0083938	44483	KaiRIA	10.04	10.27	10.25	10.11	10.04	9.50	9.69	9.54	-0.48
A_09_P065051	FBtr0087139	36819	Jhl-26	12.30	12.41	12.11	12.54	13.61	13.76	13.23	12.75	1.00
A_09_P065066	FBtr0078305	53573	Ilk	10.88	11.07	10.88	10.84	10.77	10.34	10.45	10.52	-0.40
A_09_P065076	FBtr0112935	39156	I-2	11.48	11.59	11.61	11.62	11.45	11.34	11.37	11.35	-0.20
A_09_P065106	FBtr0079297	33932	CG11070	10.46	10.46	10.56	10.50	10.12	10.14	10.14	10.36	-0.31
A_09_P065111	FBtr0084009	42500	rtet	9.84	10.28	9.87	10.10	7.56	8.05	8.95	9.55	-1.50
A_09_P065131	FBtr0088554	35963	CG8801	14.43	14.42	14.44	14.43	15.46	15.47	15.37	15.22	0.95
A_09_P065136	FBtr0070799	31481	CG4119	11.16	11.29	11.19	11.36	11.02	10.95	10.99	11.07	-0.25
A_09_P065161	FBtr0077949	33276	CG4341	8.64	8.45	8.39	8.53	9.59	10.00	9.43	9.32	1.08
A_09_P065181	FBtr0112937	41677	f-cup	9.89	10.39	10.23	10.49	11.11	10.85	10.72	10.61	0.57
A_09_P065196	FBtr0070610	31341	CG2930	14.52	14.16	14.16	13.99	14.99	14.99	14.70	14.57	0.60
A_09_P065201	FBtr0086886	37012	CG6424	10.36	10.84	10.82	10.78	11.55	11.64	11.35	11.18	0.73
A_09_P065206	FBtr0082643	41552	CG18616	10.79	10.88	10.96	10.90	11.10	11.12	11.08	11.06	0.21
A_09_P065221	FBtr0112940	37707	CG3530	10.24	10.21	10.26	10.34	10.83	10.78	10.71	10.65	0.48
A_09_P065246	FBtr0080761	34928	CG3793	10.97	11.13	11.23	11.08	10.29	10.15	10.43	10.67	-0.72
A_09_P065251	FBtr0080546	34803	cenG1A	7.27	7.20	6.99	6.90	8.19	8.49	7.75	7.62	0.92
A_09_P065256	FBtr0080746	34897	UK114	14.23	14.24	14.20	14.39	13.76	14.15	13.90	13.95	-0.32
A_09_P065281	FBtr0080714	34890	ZnT35C	11.32	11.63	11.61	11.82	8.67	8.90	10.22	10.56	-2.01
A_09_P065336	FBtr0080641	34837	mTTF	10.25	10.16	10.20	10.15	9.87	9.64	9.87	10.02	-0.34
A_09_P065351	FBtr0080550	34801	CG7953	10.70	11.46	11.05	11.02	9.16	8.85	9.25	9.40	-1.89
A_09_P065361	FBtr0080728	34874	l(2)35Be	10.84	10.91	10.83	10.89	10.32	10.12	10.50	10.71	-0.45
A_09_P065366	FBtr0080691	318935	CG31775	10.85	10.71	10.47	10.65	11.04	11.39	11.35	11.67	0.69
A_09_P065381	FBtr0343662	34779	CG9008	11.85	10.97	11.56	11.11	12.63	12.52	12.26	11.89	0.95
A_09_P065386	FBtr0080532	34778	TM9SF4	11.85	12.11	11.91	12.19	11.40	11.24	11.55	11.70	-0.54
A_09_P065421	FBtr0072187	37842	gammaSnap	11.13	11.20	11.04	11.19	11.36	11.27	11.38	11.48	0.23
A_09_P065426	FBtr0079368	33967	x16	10.52	10.76	10.72	10.83	10.04	10.01	10.20	10.41	-0.54

A_09_P065441	FBtr0088778	44269	sut2	8.40	8.22	8.30	8.29	8.80	8.93	8.78	8.80	**0.53**
A_09_P065491	FBtr0077220	33120	Ics	12.36	13.07	12.45	13.24	14.71	15.02	14.65	14.62	**1.97**
A_09_P065511	FBtr0080808	34939	beat-Ib	7.42	7.15	6.85	6.59	8.07	8.32	8.00	8.18	**1.14**
A_09_P065576	FBtr0085374	43442	Vha100-1	10.04	10.32	10.01	10.27	9.74	9.35	9.61	9.59	**-0.59**
A_09_P065586	FBtr0076052	37066	Sema-5c	10.32	10.12	10.29	10.10	9.83	9.87	10.06	10.01	**-0.27**
A_09_P065596	FBtr0084452	42805	Tbp-1	13.22	13.45	13.16	13.25	12.33	11.92	12.62	12.84	**-0.84**
A_09_P065601	FBtr0305495	31567	Rpt4	12.94	13.11	12.87	12.96	11.94	11.93	12.47	12.76	**-0.70**
A_09_P065606	FBtr0073436	32047	Rpt3	12.71	12.93	12.75	12.73	12.07	12.21	12.44	12.65	**-0.44**
A_09_P065616	FBtr0084294	42641	Rpn7	12.90	13.01	12.92	12.86	11.88	11.43	12.27	12.56	**-0.89**
A_09_P065621	FBtr0087475	36638	Rpn6	11.81	12.31	11.77	12.10	11.17	10.84	11.32	11.66	**-0.75**
A_09_P065626	FBtr0078629	40717	Rpn5	11.51	11.70	11.34	11.42	10.44	10.33	10.91	11.29	**-0.75**
A_09_P065641	FBtr0075308	39845	Rpn12	12.55	12.61	12.45	12.39	11.38	11.16	11.97	12.31	**-0.79**
A_09_P065646	FBtr0079027	33738	Rpn11	11.76	11.88	11.73	11.64	10.90	10.64	11.29	11.50	**-0.67**
A_09_P065651	FBtr0074893	40174	Rpn1	11.99	12.26	11.93	11.98	11.14	10.89	11.43	11.66	**-0.76**
A_09_P065661	FBtr0113743	3354918	RpL15	15.97	15.88	15.81	15.90	16.12	16.04	16.09	16.04	**0.18**
A_09_P065671	FBtr0080225	34550	RfC38	11.18	11.23	11.25	11.23	10.34	10.17	10.59	10.91	**-0.72**
A_09_P065701	FBtr0081969	41089	Mst85C	9.64	10.12	9.87	10.05	8.92	9.16	9.21	9.36	**-0.76**
A_09_P065706	FBtr0084935	43130	Lnk	10.02	10.38	10.24	10.36	11.54	11.69	11.12	10.79	**1.03**
A_09_P065716	FBtr0301386	37528	Fmr1	10.04	10.24	10.14	10.18	9.37	9.42	9.49	9.63	**-0.67**
A_09_P065776	FBtr0079646	49077	CSN8	11.71	12.02	11.87	12.03	11.35	11.44	11.59	11.66	**-0.40**
A_09_P065781	FBtr0088742	35816	CSN7	12.76	12.93	12.89	13.05	12.47	12.55	12.68	12.77	**-0.29**
A_09_P065786	FBtr0084265	42661	CSN6	11.87	11.92	11.88	12.00	11.44	11.36	11.46	11.53	**-0.47**
A_09_P065836	FBtr0080656	34853	CG15282	11.89	10.58	10.82	10.89	13.13	13.75	13.11	12.96	**2.19**
A_09_P065861	FBtr0303797	34951	CG42817	9.07	8.68	9.27	8.62	8.15	8.29	8.00	8.08	**-0.78**
A_09_P065906	FBtr0080606	34823	CG18095	8.03	8.44	8.12	8.08	7.03	5.77	7.08	7.42	**-1.34**
A_09_P065916	FBtr0080594	34826	nAcRalpha-34E	6.76	6.46	6.50	6.25	7.04	7.49	7.48	7.61	**0.91**
A_09_P065921	FBtr0080594	34826	nAcRalpha-34E	8.30	7.96	7.95	7.93	9.09	9.56	9.24	9.21	**1.24**
A_09_P065936	FBtr0080702	34886	CG15270	7.38	6.98	7.31	6.99	7.76	8.13	8.00	7.94	**0.79**
A_09_P065966	FBtr0343105	34889	CG4168	5.81	5.18	5.50	4.84	6.05	6.42	6.35	6.38	**0.97**
A_09_P065976	FBtr0080735	34907	CG4161	5.57	5.90	5.89	6.61	4.54	3.97	4.12	2.78	**-2.14**
A_09_P066061	FBtr0080624	34835	CG33090	11.31	11.14	11.20	11.04	11.68	11.46	11.51	11.40	**0.34**

A_09_P066101	FBtr0080536	34784	CG16863	7.61	7.58	7.78	7.69	7.05	7.35	7.39	7.28	-0.40
A_09_P066116	FBtr0080586	34815	nimB5	10.68	10.96	10.97	11.04	9.17	9.74	10.02	10.30	-1.11
A_09_P066156	FBtr0080754	34919	CG7631	12.81	11.45	11.70	11.20	13.93	14.00	13.56	13.21	1.88
A_09_P066181	FBtr0080785	34913	CG15255	13.14	11.98	12.41	12.15	14.13	14.03	13.73	13.29	1.37
A_09_P066206	FBtr0086955	36961	mthl3	9.16	9.28	9.11	9.11	9.85	9.39	9.70	9.80	0.52
A_09_P066216	FBtr0077168	38595	Aats-ala-m	11.10	10.90	10.97	10.94	10.51	10.65	10.60	10.64	-0.38
A_09_P066266	FBtr0077043	44785	tan	9.31	9.60	9.58	9.65	8.87	8.96	9.18	9.20	-0.48
A_09_P066281	FBtr0083141	49804	Spn88Ea	12.77	11.89	12.37	11.89	13.40	13.37	13.18	13.02	1.01
A_09_P066286	FBtr0086204	49805	Spn42Da	11.76	11.95	11.85	11.82	12.15	11.93	12.10	12.16	0.24
A_09_P066316	FBtr0083436	42091	sds22	11.57	11.59	11.49	11.63	11.19	10.86	11.22	11.29	-0.43
A_09_P066371	FBtr0086832	36997	icln	9.45	9.39	9.34	9.30	8.45	8.39	8.78	9.15	-0.68
A_09_P066416	FBtr0074947	40141	asf1	10.96	10.74	10.86	10.85	10.16	10.12	10.32	10.48	-0.58
A_09_P066421	FBtr0078031	33268	aru	11.74	11.82	11.59	11.91	12.88	13.26	12.69	12.46	1.06
A_09_P066431	FBtr0076734	44496	Uba2	12.14	12.43	12.33	12.47	11.93	11.79	11.93	12.11	-0.40
A_09_P066441	FBtr0076897	44498	Surf1	9.81	9.54	9.62	9.54	7.24	7.63	7.59	7.83	-2.05
A_09_P066446	FBtr0077134	44001	Sucb	14.86	14.91	14.92	14.88	14.09	13.99	14.27	14.53	-0.67
A_09_P066456	FBtr0077083	44002	Sras	8.91	8.84	9.03	8.69	9.54	9.52	9.57	9.31	0.62
A_09_P066461	FBtr0079733	44275	SoxN	9.59	9.63	9.28	9.51	9.73	10.02	9.89	9.96	0.40
A_09_P066501	FBtr0078383	44131	Mkrn1	9.09	9.19	9.08	9.12	8.74	8.72	8.66	8.68	-0.42
A_09_P066516	FBtr0085110	43936	Men-b	14.04	14.52	14.49	14.59	13.29	13.08	13.27	13.40	-1.15
A_09_P066556	FBtr0079872	47762	FKBP59	13.26	13.23	13.30	13.32	12.56	12.50	12.88	13.14	-0.51
A_09_P066561	FBtr0100513	3772101	Ext2	8.51	8.37	8.43	8.34	7.52	7.61	7.93	8.12	-0.61
A_09_P066576	FBtr0070963	44007	COQ7	12.24	12.03	12.13	12.02	12.42	12.33	12.29	12.25	0.22
A_09_P066581	FBtr0084554	42874	CHORD	12.42	12.55	12.52	12.58	10.75	10.84	11.73	12.19	-1.14
A_09_P066586	FBtr0112946	31678	CHES-1-like	7.15	6.87	6.76	6.24	8.77	8.98	8.09	7.72	1.63
A_09_P066596	FBtr0086181	35613	Tsp42Ed	13.01	12.82	12.75	12.88	13.59	13.57	13.28	13.12	0.53
A_09_P066606	FBtr0082593	41532	Aos1	11.75	11.97	11.74	11.98	11.07	10.96	11.34	11.62	-0.61
A_09_P066611	FBtr0072680	38161	312	10.77	10.49	10.80	10.68	10.09	9.99	10.18	10.19	-0.57
A_09_P066626	FBtr0070076	30988	CG13373	9.26	9.24	9.32	9.29	8.62	8.73	8.90	9.14	-0.43
A_09_P066631	FBtr0112947	30988	CG13373	9.10	9.10	9.15	9.17	8.50	8.66	8.80	8.92	-0.41
A_09_P066741	FBtr0070313	31148	CG14806	10.58	10.40	10.41	10.49	10.22	10.21	10.14	10.27	-0.26

A_09_P066771	FBtr0070564	31276	CG14419	14.45	14.47	14.51	14.60	14.88	15.00	14.95	14.95	**0.44**
A_09_P066806	FBtr0070558	31289	CG3603	11.10	11.51	11.42	11.56	10.01	10.02	10.39	10.78	**-1.10**
A_09_P066851	FBtr0070591	31317	CG10804	6.69	6.14	6.26	5.76	6.83	7.38	6.88	7.03	**0.82**
A_09_P066886	FBtr0070597	31335	HIP-R	12.98	13.58	13.48	13.42	12.75	12.90	12.69	12.80	**-0.58**
A_09_P066911	FBtr0070613	31348	CG2941	7.84	7.84	8.01	7.75	7.32	7.16	7.48	7.61	**-0.47**
A_09_P066916	FBtr0070616	31349	Vap-33-1	13.05	13.46	13.31	13.50	12.70	12.48	12.79	12.89	**-0.61**
A_09_P066926	FBtr0070625	31351	CG6428	9.44	9.42	9.68	9.51	10.28	10.50	10.02	9.83	**0.65**
A_09_P066941	FBtr0070671	31355	CG6379	9.88	10.09	10.06	10.04	9.67	9.75	9.67	9.79	**-0.30**
A_09_P066991	FBtr0070652	31377	CG3626	10.60	11.00	10.92	10.89	10.07	10.01	10.17	10.33	**-0.70**
A_09_P067001	FBtr0070674	31380	CG3556	8.22	8.17	8.27	8.09	8.70	8.74	8.51	8.47	**0.42**
A_09_P067021	FBtr0070677	31385	CG15912	9.40	9.37	9.50	9.32	8.44	8.40	8.63	8.87	**-0.81**
A_09_P067036	FBtr0070698	31388	CG11444	12.66	12.60	12.68	12.67	12.17	12.32	12.37	12.41	**-0.33**
A_09_P067206	FBtr0301950	31456	SK	5.67	5.10	4.82	4.74	6.45	6.84	6.24	6.46	**1.42**
A_09_P067216	FBtr0070797	31457	NAAT1	7.72	7.47	7.45	7.54	9.22	8.92	8.62	8.31	**1.22**
A_09_P067236	FBtr0070777	31461	CG15784	14.04	14.61	14.70	14.42	15.72	15.84	15.72	15.86	**1.34**
A_09_P067241	FBtr0070778	31463	SPR	7.19	6.84	6.79	6.11	8.31	8.05	7.61	7.54	**1.14**
A_09_P067246	FBtr0339782	31464	CG3239	8.82	8.52	8.59	8.33	9.87	10.35	9.78	9.57	**1.33**
A_09_P067271	FBtr0300466	8674066	CG42492	9.41	8.89	8.94	8.85	9.80	9.70	9.85	9.89	**0.79**
A_09_P067291	FBtr0070851	31480	Sirt4	11.20	11.11	11.14	11.02	10.64	10.54	10.67	10.72	**-0.47**
A_09_P067306	FBtr0070847	31487	CG3160	8.99	9.35	9.07	9.15	8.74	8.59	8.71	8.81	**-0.43**
A_09_P067331	FBtr0070840	31499	lin-52	8.99	9.03	9.02	9.05	8.39	8.68	8.82	8.70	**-0.37**
A_09_P067376	FBtr0070814	31509	CG12239	8.91	8.59	8.76	8.70	9.74	9.68	9.46	9.51	**0.85**
A_09_P067416	FBtr0070830	31517	CG3033	11.83	12.03	11.99	12.07	11.64	11.74	11.64	11.65	**-0.31**
A_09_P067421	FBtr0070828	31519	CG3016	11.35	11.67	11.55	11.61	11.05	11.06	11.11	11.25	**-0.43**
A_09_P067446	FBtr0070827	31524	CG3011	14.25	14.49	14.49	14.48	13.80	14.04	14.01	14.25	**-0.40**
A_09_P067451	FBtr0070858	31525	CG3726	8.24	7.79	7.84	7.91	10.13	10.54	9.80	9.36	**2.01**
A_09_P067461	FBtr0070861	31527	CG6041	10.87	10.74	10.95	10.77	11.85	11.63	11.49	11.04	**0.67**
A_09_P067466	FBtr0070863	31528	CG6048	9.59	9.49	9.28	9.07	11.11	10.63	10.53	10.13	**1.24**
A_09_P067471	FBtr0070864	31529	CG6067	9.44	10.73	10.01	10.41	8.55	8.96	8.73	8.95	**-1.35**
A_09_P067486	FBtr0070867	31536	CG5941	12.30	12.36	12.44	12.44	11.85	11.96	11.97	12.18	**-0.40**
A_09_P067496	FBtr0336972	31538	CG5921	8.23	8.25	8.28	8.09	9.97	9.78	9.34	8.92	**1.29**

A_09_P067516	FBtr0070886	31542	CG4660	6.94	6.70	6.62	6.48	7.18	7.57	7.36	7.44	0.70
A_09_P067561	FBtr0089869	31562	CG3566	13.03	12.94	12.94	13.00	12.32	12.46	12.57	12.76	-0.45
A_09_P067611	FBtr0070924	31578	CG3446	11.09	10.99	11.21	11.16	10.30	10.11	10.37	10.29	-0.84
A_09_P067666	FBtr0070993	31597	CG3226	12.37	12.51	12.44	12.61	10.89	11.12	11.80	12.24	-0.97
A_09_P067671	FBtr0070992	31600	CG3224	11.60	11.23	11.43	11.29	12.32	12.18	12.04	11.88	0.72
A_09_P067681	FBtr0070990	31604	CG3192	14.08	14.15	14.14	14.19	13.73	13.52	13.75	13.93	-0.41
A_09_P067696	FBtr0070989	31608	CG3184	9.52	9.34	9.46	9.47	9.87	10.09	9.79	9.64	0.40
A_09_P067711	FBtr0070959	31611	CG14441	6.02	5.99	5.27	5.24	6.93	6.66	6.35	6.65	1.02
A_09_P067781	FBtr0071041	31630	Cht11	8.46	8.98	8.72	8.82	8.43	8.26	8.24	8.35	-0.42
A_09_P067791	FBtr0071040	31632	CG14434	9.97	10.12	10.11	10.15	9.66	9.83	9.82	9.98	-0.27
A_09_P067846	FBtr0071020	31656	CG4615	9.34	10.06	9.90	10.05	8.54	8.82	8.79	9.00	-1.05
A_09_P067941	FBtr0071076	31683	Pdp	12.30	12.60	12.59	12.73	11.95	11.99	11.93	11.96	-0.60
A_09_P067946	FBtr0071072	31684	Rab39	8.94	9.26	9.21	9.28	8.74	8.79	8.76	8.72	-0.42
A_09_P067991	FBtr0071143	31695	CG10932	14.03	14.28	14.09	14.32	13.25	13.13	13.39	13.76	-0.80
A_09_P068001	FBtr0071141	31697	CG18624	13.79	13.76	13.97	13.93	13.15	13.06	13.25	13.31	-0.67
A_09_P068081	FBtr0071116	31726	CG2254	13.66	13.85	13.52	13.52	12.53	12.58	12.86	13.25	-0.83
A_09_P068151	FBtr0071157	31743	CG10959	8.30	8.50	8.51	8.54	7.83	7.94	8.02	8.31	-0.44
A_09_P068201	FBtr0071183	31761	sni	12.39	12.34	12.51	12.38	12.02	12.20	12.19	12.27	-0.23
A_09_P068216	FBtr0071178	31765	CG15343	8.13	8.44	8.49	8.46	6.85	7.41	7.33	7.34	-1.15
A_09_P068221	FBtr0071171	31766	CG1636	7.08	7.19	7.22	6.89	6.02	6.46	6.23	6.84	-0.71
A_09_P068231	FBtr0071174	31771	CG10555	8.26	8.84	8.66	8.68	7.58	8.04	8.01	8.29	-0.63
A_09_P068256	FBtr0071242	31779	CG15347	14.15	13.59	13.88	13.57	14.97	14.91	14.66	14.17	0.88
A_09_P068276	FBtr0071232	31796	CG12111	3.75	3.44	3.09	3.89	5.99	6.28	5.73	5.10	2.23
A_09_P068296	FBtr0071228	31801	Caf1-180	8.45	8.41	8.37	8.46	7.54	7.71	7.86	7.95	-0.65
A_09_P068306	FBtr0071222	31804	CG11284	13.16	13.25	13.22	13.24	13.13	13.10	13.02	12.94	-0.17
A_09_P068311	FBtr0071225	31805	Ppt1	9.44	9.73	9.73	9.75	9.37	8.88	8.98	8.96	-0.62
A_09_P068341	FBtr0071268	31817	CG1885	9.35	9.40	9.58	9.52	7.99	8.19	8.50	8.63	-1.14
A_09_P068386	FBtr0071356	31830	CG10970	4.47	4.03	4.67	4.80	6.94	7.05	6.23	5.72	1.99
A_09_P068406	FBtr0071284	31834	HP1b	10.20	10.38	10.49	10.41	9.71	9.80	9.93	10.01	-0.51
A_09_P068416	FBtr0071289	31838	CG7033	13.52	13.41	13.51	13.41	13.15	13.07	13.15	13.18	-0.32
A_09_P068421	FBtr0071352	31839	CG7766	10.87	11.50	11.33	11.55	10.23	10.39	10.34	10.72	-0.89

A_09_P068426	FBtr0071293	31841	CG7039	9.10	9.15	9.08	9.26	8.81	8.92	8.84	8.93	-0.27
A_09_P068446	FBtr0071348	31845	fh	9.10	8.97	9.17	9.00	8.54	8.65	8.77	8.93	-0.34
A_09_P068471	FBtr0071304	31854	CG12057	11.84	12.74	11.52	12.92	13.57	13.83	13.41	13.07	1.22
A_09_P068491	FBtr0071336	31859	CG12119	10.12	11.13	10.75	11.05	8.22	8.32	8.70	8.90	-2.23
A_09_P068521	FBtr0071334	31866	CG12121	12.49	12.69	12.58	12.73	12.05	12.04	12.11	12.32	-0.49
A_09_P068541	FBtr0071327	31880	Cfp1	9.68	9.78	9.80	9.85	10.13	10.28	10.05	10.01	0.34
A_09_P068551	FBtr0071360	31897	RpS28b	15.85	15.89	15.83	15.93	15.97	15.99	16.00	16.04	0.12
A_09_P068566	FBtr0071368	31903	Lst8	11.36	11.46	11.47	11.40	11.59	11.77	11.73	11.67	0.27
A_09_P068591	FBtr0089384	31914	CG1354	14.55	14.51	14.56	14.51	14.16	14.07	14.29	14.47	-0.29
A_09_P068606	FBtr0071389	31921	CG9686	10.27	10.17	10.31	10.23	10.91	11.28	10.87	10.92	0.75
A_09_P068616	FBtr0071392	31923	CG9691	13.66	13.44	13.73	13.60	14.04	14.18	14.15	14.07	0.51
A_09_P068706	FBtr0083623	42165	CG7218	9.55	9.91	9.80	10.03	9.44	9.25	9.26	9.43	-0.48
A_09_P068711	FBtr0100619	3771951	Prx5	14.73	14.77	14.79	14.83	14.21	14.19	14.39	14.62	-0.43
A_09_P068716	FBtr0083619	42171	cdm	10.70	10.98	10.99	10.92	10.35	10.52	10.36	10.30	-0.51
A_09_P068756	FBtr0083610	42181	CG7183	8.32	8.29	8.31	8.16	8.49	8.48	8.51	8.61	0.25
A_09_P068771	FBtr0083608	42184	CG7168	11.19	11.46	11.45	11.51	10.59	10.78	10.79	10.88	-0.65
A_09_P068786	FBtr0083569	42188	CG18598	5.44	5.89	5.70	6.37	4.01	4.46	4.31	4.58	-1.51
A_09_P068791	FBtr0083605	42189	CG12320	8.97	8.87	8.88	8.90	8.62	8.75	8.59	8.63	-0.26
A_09_P068826	FBtr0083594	42201	CG18600	11.53	10.70	11.08	10.79	11.85	11.82	11.62	11.55	0.69
A_09_P068836	FBtr0083589	42203	PKD	8.62	8.93	8.70	8.93	8.98	9.32	9.39	9.60	0.53
A_09_P068851	FBtr0083586	42208	WRNexo	9.41	9.67	9.65	9.62	8.40	8.02	8.67	8.97	-1.07
A_09_P068856	FBtr0083577	42209	Nup43	11.06	10.91	10.93	10.93	10.71	10.66	10.67	10.77	-0.25
A_09_P068881	FBtr0083655	42218	CG12333	9.61	9.95	9.77	9.88	9.44	9.31	9.26	9.39	-0.45
A_09_P068901	FBtr0083632	42230	CG7694	12.15	12.40	12.20	12.42	11.63	11.45	11.77	12.04	-0.57
A_09_P068911	FBtr0083639	42232	CG14304	10.07	10.22	10.09	10.46	9.64	9.95	9.80	9.82	-0.41
A_09_P068946	FBtr0083668	42245	CG7708	9.33	9.04	9.06	8.68	9.78	10.00	9.49	9.89	0.76
A_09_P068956	FBtr0100288	3346166	CG14300	10.56	10.85	10.80	11.36	9.49	9.55	9.49	9.41	-1.41
A_09_P068961	FBtr0083686	42250	CG7714	13.78	13.82	13.80	13.90	12.50	12.18	12.30	12.40	-1.48
A_09_P069016	FBtr0083725	42264	CG14292	15.02	14.87	15.18	15.13	13.87	14.68	14.38	14.50	-0.69
A_09_P069021	FBtr0083698	42265	endoA	8.97	8.67	8.79	8.91	9.01	9.10	9.10	9.15	0.26
A_09_P069036	FBtr0083700	42267	Xrp1	13.81	14.20	14.32	14.11	15.40	15.52	15.10	14.89	1.12

A_09_P069046	FBtr0083722	42271	CG12269	6.24	6.70	6.71	6.82	3.42	4.76	4.67	4.47	-2.29
A_09_P069051	FBtr0083721	42272	Smu1	10.78	10.69	10.71	10.77	9.90	10.03	10.32	10.45	-0.56
A_09_P069056	FBtr0083720	42274	snRNP-U1-C	12.15	12.10	12.09	12.27	11.63	11.75	11.82	11.94	-0.37
A_09_P069071	FBtr0083711	42281	CG14285	12.77	13.06	13.01	13.07	11.27	11.32	11.73	12.12	-1.37
A_09_P069076	FBtr0083714	42286	CG6013	11.12	11.20	11.22	11.17	10.77	10.66	10.82	11.08	-0.35
A_09_P069086	FBtr0083754	42290	mRpL55	12.42	12.17	12.33	12.20	11.51	11.85	11.81	12.12	-0.46
A_09_P069091	FBtr0083729	42292	CG6040	11.48	11.06	10.91	11.05	11.61	11.79	11.77	11.95	0.65
A_09_P069096	FBtr0083730	42293	Cyp12a5	11.29	12.11	11.84	12.25	10.92	10.77	10.75	10.49	-1.14
A_09_P069101	FBtr0083731	42294	Cyp12a4	13.26	13.42	13.19	13.33	14.62	14.27	14.38	14.23	1.07
A_09_P069106	FBtr0083751	42296	CG5835	12.33	11.84	12.21	11.85	13.90	13.55	13.27	12.82	1.32
A_09_P069116	FBtr0083751	42296	CG5835	12.17	11.75	12.03	11.59	13.71	13.42	13.10	12.62	1.33
A_09_P069136	FBtr0083746	42302	CG5555	10.53	10.71	10.63	10.67	10.41	10.15	10.26	10.34	-0.35
A_09_P069156	FBtr0301693	42310	unc79	8.62	8.26	8.30	8.19	9.08	9.31	9.12	9.37	0.88
A_09_P069176	NM_142545	42318	CG3734	11.35	9.96	10.39	9.82	12.25	11.91	11.88	11.69	1.55
A_09_P069181	FBtr0083756	42319	CG18493	13.82	13.39	13.59	13.73	14.47	14.16	14.14	13.97	0.56
A_09_P069236	FBtr0273217	42336	CG17751	11.49	12.60	12.32	12.79	4.50	6.96	9.27	9.98	-4.62
A_09_P069241	FBtr0083779	42337	CG17752	11.68	12.29	12.10	12.49	10.16	10.73	10.95	11.37	-1.34
A_09_P069246	FBtr0083780	42338	CG16727	8.36	10.17	9.05	9.83	4.93	5.69	6.52	7.56	-3.18
A_09_P069256	FBtr0083805	42340	CG16718	10.51	10.83	10.81	10.75	9.96	9.80	9.60	9.47	-1.02
A_09_P069261	FBtr0083781	42342	Nup58	11.76	11.78	11.84	11.87	11.57	11.42	11.62	11.72	-0.23
A_09_P069266	FBtr0083803	42343	CG6195	12.75	12.64	12.73	12.63	12.19	12.29	12.39	12.48	-0.35
A_09_P069286	FBtr0083797	42352	CG11659	11.41	11.72	11.78	12.15	9.14	8.86	9.93	10.31	-2.20
A_09_P069301	FBtr0083791	42355	CG11453	11.95	11.39	11.47	11.31	12.66	12.64	12.34	12.10	0.91
A_09_P069311	FBtr0083814	42357	CG4662	10.92	11.11	11.07	11.02	10.16	9.91	10.10	10.23	-0.93
A_09_P069331	FBtr0083816	42361	CG4686	10.11	10.10	10.28	10.38	9.80	9.61	9.80	9.83	-0.45
A_09_P069341	FBtr0083858	42363	CG17186	8.82	8.99	9.05	8.92	8.55	8.67	8.71	8.72	-0.28
A_09_P069346	FBtr0083817	42364	Arc42	12.60	12.84	12.77	12.87	11.88	11.89	12.25	12.43	-0.66
A_09_P069351	FBtr0301117	42368	CG4733	10.41	10.53	10.63	10.43	9.98	9.93	9.89	10.00	-0.55
A_09_P069391	FBtr0083850	42376	CG4462	10.92	11.55	11.31	11.49	10.28	10.51	10.37	10.41	-0.92
A_09_P069406	FBtr0083823	42381	CG4783	9.24	9.00	9.28	9.19	11.78	11.85	11.24	10.56	2.18
A_09_P069431	FBtr0083829	42389	psidin	12.32	12.16	12.43	12.25	11.79	11.90	11.84	12.02	-0.40

A_09_P069471	FBtr0083836	42396	CG4390	12.11	12.35	12.13	12.40	11.54	11.25	11.56	11.81	**-0.71**
A_09_P069486	FBtr0083866	42400	CG5023	14.87	14.95	15.08	15.05	13.71	13.64	13.90	13.94	**-1.19**
A_09_P069506	FBtr0083901	42409	CG4367	12.85	12.55	12.48	12.71	14.76	15.05	14.44	13.96	**1.91**
A_09_P069511	FBtr0083900	42410	CG4362	14.44	14.15	13.26	14.03	16.79	16.49	16.49	16.05	**2.48**
A_09_P069531	FBtr0113252	42413	CG4360	10.25	10.11	10.14	10.11	9.82	10.00	9.98	9.97	**-0.21**
A_09_P069536	FBtr0083882	42414	Sirt2	11.47	11.36	11.45	11.44	11.02	11.05	11.15	11.28	**-0.31**
A_09_P069551	FBtr0083894	42421	CG4335	11.85	12.07	11.99	12.14	10.97	11.10	11.43	11.69	**-0.72**
A_09_P069581	FBtr0083950	42432	CG10877	11.42	11.54	11.61	11.60	12.96	12.68	12.52	12.23	**1.05**
A_09_P069596	FBtr0273271	42436	Srp14	13.02	12.93	13.07	13.12	12.23	12.45	12.63	12.83	**-0.50**
A_09_P069606	FBtr0083911	42439	Srp72	12.93	12.97	12.88	12.87	11.62	11.55	12.01	12.33	**-1.04**
A_09_P069666	FBtr0083995	2768674	Fancd2	7.69	8.02	7.65	8.00	6.73	6.91	7.00	7.06	**-0.91**
A_09_P069676	FBtr0083993	42463	CG17271	12.09	12.57	12.40	12.62	10.54	10.75	11.56	12.05	**-1.19**
A_09_P069681	FBtr0110823	42463	CG17271	13.40	13.56	13.41	13.52	13.28	13.32	13.17	13.11	**-0.25**
A_09_P069741	FBtr0084048	42482	CG10827	13.02	12.68	12.39	12.43	14.96	14.99	14.55	13.89	**1.97**
A_09_P069776	FBtr0084036	42496	RhoGAP93B	8.39	8.51	8.49	8.31	7.79	7.98	7.97	8.09	**-0.47**
A_09_P069786	FBtr0084008	42498	CG5745	9.79	10.00	9.86	9.93	9.48	9.43	9.57	9.64	**-0.37**
A_09_P069791	FBtr0084034	42499	sec15	8.56	8.55	8.62	8.60	7.79	7.92	8.05	8.10	**-0.62**
A_09_P069801	FBtr0333930	42505	CG5793	13.40	13.60	13.53	13.70	12.19	12.27	12.48	12.82	**-1.12**
A_09_P069816	FBtr0084029	42508	CG7009	10.29	10.18	10.32	10.24	9.80	9.93	9.84	9.91	**-0.39**
A_09_P069831	FBtr0084028	42512	CG10824	10.36	10.31	10.25	10.23	10.02	10.13	10.09	10.05	**-0.21**
A_09_P069841	FBtr0333928	42514	Snmp1	5.46	5.87	5.46	5.66	4.12	4.49	3.30	4.30	**-1.57**
A_09_P069846	FBtr0084049	42516	CG5862	13.60	13.74	13.59	13.80	13.11	13.27	13.32	13.39	**-0.41**
A_09_P069851	FBtr0084108	42517	CG3353	11.34	10.85	11.05	10.92	11.89	12.00	11.56	11.47	**0.69**
A_09_P069856	FBtr0084050	42518	Oga	11.33	11.50	11.45	11.45	10.76	10.81	10.96	11.24	**-0.49**
A_09_P069861	FBtr0084107	42519	CG3337	11.61	11.49	11.60	11.51	11.10	10.92	11.00	11.03	**-0.54**
A_09_P069871	FBtr0339193	42522	CG5892	8.06	8.18	7.28	7.83	9.97	9.34	9.42	9.00	**1.59**
A_09_P069896	FBtr0084101	42528	CG3301	12.65	13.76	13.43	13.70	10.33	11.08	10.80	11.11	**-2.55**
A_09_P069906	FBtr0084098	42530	SIFR	5.46	5.11	4.86	4.69	6.48	7.09	6.41	6.38	**1.56**
A_09_P069956	FBtr0084113	42552	CG15497	10.19	4.37	8.69	5.35	11.09	11.26	10.75	10.23	**3.69**
A_09_P069961	FBtr0084125	42556	CG5849	9.97	9.54	9.69	9.48	10.99	10.34	10.48	10.21	**0.83**
A_09_P069966	FBtr0344211	42558	CG42335	9.24	7.94	8.55	8.11	12.72	12.35	12.14	11.35	**3.68**

A_09_P069971	FBtr0084131	42560	burs	8.01	6.24	7.19	6.45	10.94	10.77	10.27	9.45	3.39
A_09_P070011	FBtr0084151	42580	dnd	9.17	9.26	9.34	9.46	7.64	7.89	7.82	8.27	-1.40
A_09_P070036	FBtr0084190	42586	CG6439	11.66	11.73	11.78	11.70	10.95	10.50	10.81	10.84	-0.94
A_09_P070041	FBtr0084171	42588	mRpL35	11.85	11.37	11.58	11.42	10.88	10.77	11.02	11.06	-0.62
A_09_P070061	FBtr0084183	42593	CG6015	11.03	10.96	11.06	11.15	11.26	11.21	11.20	11.22	0.17
A_09_P070066	FBtr0084176	42594	BG4	8.40	8.54	8.45	8.51	8.00	8.00	7.93	7.82	-0.54
A_09_P070106	FBtr0084204	42610	CG13862	9.76	9.47	9.35	9.55	10.09	10.59	10.19	10.15	0.72
A_09_P070121	FBtr0084240	42613	CG5386	7.68	7.58	7.87	7.48	6.02	6.65	6.51	7.15	-1.07
A_09_P070136	FBtr0084239	42616	PSR	12.21	12.14	12.25	12.18	12.45	12.51	12.42	12.34	0.24
A_09_P070141	FBtr0084211	42617	CG7071	11.72	11.58	11.64	11.61	11.39	11.38	11.31	11.38	-0.28
A_09_P070151	FBtr0084236	42619	CG5380	9.36	9.29	9.26	9.30	8.43	8.71	8.58	8.84	-0.66
A_09_P070161	FBtr0084216	42622	CG18596	10.31	10.40	10.85	10.43	11.10	11.28	11.12	10.82	0.58
A_09_P070171	FBtr0084219	42626	CG7059	5.85	6.25	6.11	6.55	8.55	8.64	7.86	7.18	1.87
A_09_P070206	FBtr0084297	42635	pinta	7.90	8.04	7.56	7.72	8.74	8.60	8.54	8.41	0.77
A_09_P070226	FBtr0084255	42644	Pebp1	16.05	16.14	16.03	16.09	15.85	15.78	15.77	15.66	-0.31
A_09_P070231	FBtr0084293	42645	CG5377	11.92	12.14	12.16	12.18	10.49	10.58	11.02	11.41	-1.23
A_09_P070236	FBtr0334612	42646	Nrx-1	10.23	10.14	10.16	10.07	10.84	11.21	10.75	10.94	0.79
A_09_P070246	FBtr0084292	42648	CG5376	11.21	11.50	11.25	11.51	10.12	9.89	10.34	10.64	-1.12
A_09_P070266	FBtr0084289	42653	CG5346	12.21	12.13	12.18	12.28	13.62	13.67	13.12	12.73	1.09
A_09_P070331	FBtr0084334	42679	wfs1	8.85	8.82	9.03	8.96	9.23	9.32	9.36	9.38	0.41
A_09_P070356	FBtr0084305	42684	CG6972	11.37	10.80	11.25	10.74	9.67	9.70	9.99	9.99	-1.21
A_09_P070371	FBtr0084326	42690	CG4813	9.21	9.04	9.23	9.09	8.75	8.92	8.89	8.95	-0.27
A_09_P070391	FBtr0084308	42694	CG6985	7.54	7.91	7.73	7.92	8.24	8.05	8.09	8.06	0.33
A_09_P070401	FBtr0084309	42696	HP1c	10.69	10.81	10.64	10.87	10.05	9.83	10.31	10.50	-0.58
A_09_P070406	FBtr0084322	42697	CG17141	9.36	9.05	9.07	9.01	8.37	8.75	8.50	8.56	-0.58
A_09_P070411	FBtr0084319	42699	CG4725	11.14	12.03	11.56	11.77	8.09	8.78	9.92	10.60	-2.28
A_09_P070416	FBtr0084318	42700	CG4723	8.29	9.34	8.75	9.00	5.29	6.52	7.00	7.42	-2.29
A_09_P070421	FBtr0084317	42701	CG4721	11.13	11.74	11.53	11.57	10.33	9.83	10.43	10.83	-1.14
A_09_P070436	FBtr0084312	42703	CG7029	11.89	12.21	12.13	12.26	13.04	13.05	12.61	12.41	0.66
A_09_P070461	FBtr0084340	42709	CG17244	12.04	12.63	12.35	12.69	11.05	11.21	11.47	11.64	-1.09
A_09_P070496	FBtr0084345	42720	CG13837	4.39	4.24	4.29	3.91	5.14	5.40	5.48	5.64	1.21

A_09_P070501	FBtr0084361	42721	CG17121	10.29	10.60	10.40	10.66	11.58	11.64	11.18	10.93	0.85
A_09_P070506	FBtr0084360	2768677	p53	8.68	8.28	8.37	8.39	8.80	9.09	8.73	8.82	0.43
A_09_P070511	FBtr0084357	42723	CG17119	11.31	11.16	10.97	11.14	12.30	12.10	11.79	11.43	0.76
A_09_P070521	FBtr0084349	42728	CG6726	14.07	14.39	14.33	14.49	13.89	13.56	13.83	13.95	-0.51
A_09_P070531	FBtr0084351	42730	CG17109	12.68	12.16	12.41	12.23	14.24	13.62	13.67	13.31	1.34
A_09_P070536	FBtr0084352	42731	CG6733	12.56	12.68	12.86	13.04	11.38	11.15	11.92	12.31	-1.09
A_09_P070541	FBtr0084353	42732	CG6738	9.82	10.04	9.85	10.06	6.44	6.46	7.81	8.32	-2.69
A_09_P070551	FBtr0084407	42734	Rassf	8.24	8.21	8.12	8.08	7.95	7.98	7.89	7.99	-0.21
A_09_P070556	FBtr0084368	42735	cenB1A	8.20	8.30	8.00	8.20	8.69	8.80	8.69	8.76	0.56
A_09_P070581	FBtr0110801	42747	CG4467	7.67	7.77	7.70	7.74	8.20	8.31	8.15	8.12	0.48
A_09_P070601	FBtr0084376	42751	CG13827	10.41	10.31	10.32	10.26	9.32	9.56	9.54	9.89	-0.75
A_09_P070621	FBtr0084431	42762	CG4408	10.51	10.61	10.50	10.53	7.98	7.81	9.59	10.20	-1.65
A_09_P070681	FBtr0084419	42775	CG16732	9.73	9.15	9.37	8.98	7.16	7.48	7.46	7.63	-1.87
A_09_P070696	FBtr0084433	42778	beat-IV	7.34	7.13	6.87	6.59	8.27	8.57	7.67	8.29	1.22
A_09_P070701	FBtr0084486	42780	CG10182	11.29	11.44	10.88	11.27	12.95	12.62	12.46	12.02	1.29
A_09_P070706	FBtr0304611	42781	CG16723	3.63	3.68	3.26	3.40	6.03	5.43	5.67	5.63	2.19
A_09_P070716	FBtr0084481	42783	CG10184	13.59	13.76	13.69	13.84	12.37	12.41	12.99	13.30	-0.95
A_09_P070721	FBtr0084434	42787	CG13822	11.29	11.76	11.61	11.90	10.86	10.83	10.84	10.88	-0.78
A_09_P070726	FBtr0084435	42788	CG10157	7.40	8.44	6.61	6.87	10.01	10.10	9.66	9.48	2.48
A_09_P070731	FBtr0303150	42790	CG16710	7.67	7.54	7.44	7.49	6.42	7.24	7.00	7.11	-0.59
A_09_P070751	FBtr0084445	42798	CG10300	8.42	8.95	8.28	8.48	10.03	9.69	9.52	9.33	1.11
A_09_P070756	FBtr0334768	42800	CG10232	8.79	6.18	7.73	5.98	10.21	10.09	9.65	9.23	2.63
A_09_P070766	FBtr0084468	42804	RanBP3	10.20	10.75	10.60	10.61	9.80	9.73	9.69	9.70	-0.81
A_09_P070771	FBtr0084453	42807	Plip	11.99	11.71	11.46	11.46	12.03	12.04	12.10	12.25	0.45
A_09_P070776	FBtr0084466	42808	CG10219	11.43	11.72	11.50	11.84	10.56	10.40	10.49	10.66	-1.09
A_09_P070786	FBtr0084455	42810	Lsd-1	13.17	13.13	13.21	13.10	10.90	10.84	12.20	12.66	-1.50
A_09_P070801	FBtr0084462	42813	tst	10.21	10.17	10.28	10.35	9.71	9.87	9.84	10.04	-0.39
A_09_P070811	FBtr0084460	42816	Nup98-96	10.35	10.37	10.51	10.46	10.79	11.07	10.78	10.66	0.40
A_09_P070831	FBtr0084522	42826	CG13601	11.72	11.44	11.56	11.43	11.21	11.27	11.27	11.34	-0.26
A_09_P070841	FBtr0084493	42829	CG13599	7.92	8.41	8.23	8.58	7.75	7.66	7.66	7.75	-0.58
A_09_P070866	FBtr0084510	42838	CG13603	10.28	10.65	10.47	10.66	11.42	11.36	11.16	10.92	0.70

A_09_P070901	FBtr0343101	42851	CG6000	8.16	7.71	7.98	7.64	9.32	9.27	9.61	9.84	1.64
A_09_P070916	FBtr0300705	42858	CG13605	9.82	10.13	9.88	10.06	9.66	9.70	9.62	9.60	-0.33
A_09_P070921	FBtr0084585	42859	CG13607	11.31	11.93	11.73	11.83	10.15	10.28	10.19	10.36	-1.45
A_09_P070926	FBtr0339244	42860	Rootletin	9.50	11.03	10.39	11.30	7.62	7.91	7.44	7.52	-2.94
A_09_P070941	FBtr0084549	42865	Kal1	9.71	11.38	10.76	10.96	6.49	6.94	6.88	7.02	-3.87
A_09_P070946	FBtr0084550	42867	CG6178	13.05	13.05	12.99	12.98	12.34	12.17	12.34	12.50	-0.68
A_09_P070986	FBtr0084574	42875	CG5515	13.13	13.14	13.07	13.14	12.66	12.80	12.91	13.05	-0.26
A_09_P070991	FBtr0084573	42876	CG5524	9.33	9.54	9.49	9.58	9.88	9.68	9.67	9.72	0.25
A_09_P070996	FBtr0084555	42877	CG6204	9.44	9.50	9.42	9.37	9.78	9.76	9.63	9.70	0.29
A_09_P071011	FBtr0084560	42883	CG13609	6.18	6.23	6.36	6.17	5.02	5.30	5.49	5.79	-0.84
A_09_P071016	FBtr0305044	42885	Spase22-23	8.27	8.84	8.37	8.72	7.13	7.36	7.73	8.01	-0.99
A_09_P071021	FBtr0084637	42888	CG5706	13.74	13.60	13.58	13.53	13.08	13.06	13.27	13.42	-0.41
A_09_P071036	FBtr0084602	42894	CG6364	9.84	9.78	9.73	9.89	9.38	9.47	9.53	9.60	-0.31
A_09_P071061	FBtr0084630	42903	CG5746	9.18	9.22	9.35	9.40	8.76	9.10	8.97	9.08	-0.31
A_09_P071076	FBtr0084628	42906	CG18528	10.90	11.10	10.88	10.89	10.21	10.29	10.33	10.49	-0.61
A_09_P071151	FBtr0084644	42925	CG6607	11.15	11.47	11.39	11.49	10.80	10.91	10.88	10.99	-0.48
A_09_P071156	FBtr0084679	42926	CG13623	10.34	10.10	10.13	10.20	9.54	9.60	9.60	9.70	-0.58
A_09_P071176	FBtr0084675	42930	CG13624	7.00	7.42	6.75	6.28	8.60	8.91	7.86	8.09	1.50
A_09_P071186	FBtr0084670	42933	Syx18	11.59	11.73	11.71	11.74	10.62	10.54	11.09	11.35	-0.79
A_09_P071191	FBtr0084657	42934	atl	12.37	12.42	12.25	12.50	12.13	12.11	12.18	12.11	-0.25
A_09_P071196	FBtr0084667	42935	CG5794	6.20	6.69	6.47	6.76	5.53	5.02	5.06	5.11	-1.35
A_09_P071221	FBtr0084745	42943	CG13630	13.14	12.89	13.23	13.02	12.79	12.66	12.79	12.68	-0.34
A_09_P071281	FBtr0084705	42964	nct	9.16	9.37	9.21	9.20	8.74	8.75	8.85	8.90	-0.42
A_09_P071306	FBtr0334575	42972	CG3744	9.38	9.44	9.52	9.52	8.83	8.78	9.03	9.27	-0.49
A_09_P071346	FBtr0084749	42987	Nmnat	9.68	9.62	9.49	9.63	9.92	10.27	9.90	9.93	0.40
A_09_P071351	FBtr0084750	42987	Nmnat	9.06	8.78	8.99	8.93	9.43	9.18	9.19	9.17	0.30
A_09_P071366	FBtr0084755	42991	beta4GalT7	11.56	11.41	11.58	11.60	11.08	11.10	11.21	11.14	-0.40
A_09_P071371	FBtr0084797	42992	CG11781	11.27	10.93	11.05	10.98	10.44	10.36	10.62	10.89	-0.48
A_09_P071381	FBtr0084795	42995	CG6422	10.45	10.71	10.53	10.57	10.25	10.20	10.26	10.33	-0.30
A_09_P071391	FBtr0084761	42999	CG11790	12.66	13.07	12.94	13.02	12.21	12.19	12.48	12.65	-0.54
A_09_P071431	FBtr0113286	43014	CG13650	7.02	6.88	6.68	6.89	7.47	7.29	7.41	7.56	0.57

A_09_P071481	FBtr0084808	43031	CG11851	10.28	10.36	10.29	10.22	9.75	9.33	9.76	9.97	-0.59
A_09_P071491	FBtr0084870	43034	CG10420	10.29	10.46	10.45	10.19	8.44	8.60	9.53	10.16	-1.16
A_09_P071511	FBtr0084868	43039	RpS27	15.24	15.19	15.33	15.22	15.54	15.57	15.59	15.50	0.31
A_09_P071526	FBtr0084814	43042	CG11857	12.96	13.14	13.09	13.06	12.35	12.18	12.58	12.90	-0.56
A_09_P071561	FBtr0114505	43051	CG10513	14.07	14.14	14.19	14.24	13.05	12.76	13.42	13.74	-0.92
A_09_P071566	FBtr0084864	43052	CG10514	13.86	14.21	14.23	14.24	11.91	11.62	12.98	13.37	-1.66
A_09_P071571	FBtr0089645	43053	CG11892	13.03	13.54	13.46	13.72	10.51	10.46	11.62	11.98	-2.30
A_09_P071581	FBtr0084827	43056	CG11893	11.31	11.21	11.62	11.02	4.41	4.18	7.87	8.62	-5.02
A_09_P071586	FBtr0084828	43059	CG13659	9.99	9.97	9.98	9.87	11.43	11.02	11.26	11.15	1.26
A_09_P071601	FBtr0084853	43064	CG10553	10.29	10.34	10.36	10.41	6.77	7.41	8.22	8.85	-2.54
A_09_P071606	FBtr0084852	43065	CG10560	11.94	12.22	12.15	12.48	9.26	9.21	10.76	11.31	-2.07
A_09_P071621	FBtr0084848	43069	CG10669	6.57	6.74	6.65	6.65	5.73	6.17	6.00	6.28	-0.61
A_09_P071636	FBtr0084846	43074	alrm	9.49	9.21	9.37	9.14	9.84	9.84	9.78	9.78	0.51
A_09_P071641	FBtr0084835	43077	Vps33B	11.04	11.07	11.22	11.19	10.86	10.72	10.89	10.88	-0.29
A_09_P071666	FBtr0084878	43083	CG5112	13.30	13.37	13.42	13.35	12.15	12.56	12.57	12.93	-0.81
A_09_P071706	FBtr0084893	43091	Npl4	12.78	13.10	12.99	13.09	12.55	12.35	12.60	12.77	-0.42
A_09_P071711	FBtr0084891	43092	Ssadh	12.62	12.75	12.63	12.73	12.41	12.22	12.45	12.58	-0.26
A_09_P071731	FBtr0084934	43099	CG4730	7.39	7.70	7.67	8.08	7.23	7.01	7.01	7.04	-0.64
A_09_P071746	FBtr0113289	43102	CG5028	12.29	12.72	12.68	12.75	12.20	11.86	12.02	12.21	-0.53
A_09_P071756	FBtr0084929	43104	CLS	11.84	12.00	11.90	11.98	11.49	11.18	11.33	11.29	-0.61
A_09_P071811	FBtr0084907	43125	CG5886	12.71	12.89	12.88	12.84	11.13	10.93	11.46	11.95	-1.47
A_09_P071846	FBtr0290140	43136	CG42235	10.01	11.25	10.67	11.22	8.24	8.40	8.93	9.18	-2.10
A_09_P071851	FBtr0290139	43136	CG42235	8.41	9.80	9.11	9.59	7.08	7.33	7.34	7.43	-1.93
A_09_P071891	FBtr0084975	43166	Sld5	10.46	10.27	10.48	10.41	9.97	10.12	9.98	10.04	-0.38
A_09_P071901	FBtr0084974	43168	CG14550	10.44	10.34	10.30	10.43	9.88	9.98	10.08	10.31	-0.31
A_09_P071971	FBtr0085012	43192	CG5447	10.59	10.80	10.71	10.60	10.37	10.32	10.42	10.28	-0.32
A_09_P071976	FBtr0330153	43194	CG14237	9.13	9.27	8.82	9.88	11.69	11.71	11.41	10.89	2.15
A_09_P071986	FBtr0085015	43196	CG5455	11.07	11.19	11.08	10.90	9.98	10.01	9.96	10.06	-1.05
A_09_P072036	FBtr0085031	43207	TwdlN	15.82	12.50	14.95	13.92	16.32	16.33	16.16	15.62	1.81
A_09_P072081	FBtr0085039	43217	CG5484	12.21	12.31	12.13	12.35	11.19	11.16	11.73	12.01	-0.73
A_09_P072086	FBtr0085046	43218	CG6420	10.04	10.13	10.21	10.25	9.99	9.99	9.97	9.89	-0.20

A_09_P072096	FBtr0085044	43220	CG6403	13.77	13.73	13.90	13.78	14.92	14.47	14.63	14.46	**0.83**
A_09_P072141	FBtr0085073	43243	CG14253	11.42	11.38	11.53	11.51	12.32	12.41	12.05	11.71	**0.66**
A_09_P072146	FBtr0085075	43245	TwdlC	6.78	NA	5.98	NA	3.11	3.47	4.16	4.60	**-2.54**
A_09_P072156	FBtr0085077	43247	CG6295	14.11	14.88	14.26	14.82	13.61	13.34	13.41	13.37	**-1.09**
A_09_P072166	FBtr0085152	43249	CG17191	9.98	10.95	9.87	10.74	8.26	7.84	8.27	8.22	**-2.24**
A_09_P072211	FBtr0085104	43261	CG14259	7.47	8.65	8.10	8.76	5.49	6.64	5.87	6.15	**-2.21**
A_09_P072226	FBtr0085133	43264	CG6074	8.54	9.44	8.93	9.14	6.80	6.16	7.59	7.90	**-1.90**
A_09_P072231	FBtr0085132	43265	gb	9.05	9.73	9.71	9.76	8.88	8.90	8.59	8.53	**-0.84**
A_09_P072296	FBtr0085158	43289	CG5934	11.11	11.18	11.29	11.30	10.74	10.67	10.79	10.92	**-0.44**
A_09_P072311	FBtr0085173	43293	bigmax	10.98	11.17	10.87	11.14	12.36	12.40	11.79	11.45	**0.96**
A_09_P072346	FBtr0339319	43304	Cyp6a18	9.24	9.95	9.92	10.35	7.38	7.23	7.94	8.23	**-2.17**
A_09_P072376	FBtr0085202	43314	CG5639	12.26	11.01	11.30	11.04	12.33	12.43	12.73	13.01	**1.22**
A_09_P072381	FBtr0301390	43315	dsd	7.51	8.08	7.83	7.37	8.94	9.13	8.21	8.64	**1.03**
A_09_P072396	FBtr0085199	43318	CG5611	9.79	9.92	9.86	9.89	9.69	9.53	9.58	9.68	**-0.24**
A_09_P072401	FBtr0085192	43319	Mtl	8.14	8.46	8.57	8.32	8.15	7.73	7.70	7.83	**-0.52**
A_09_P072411	FBtr0085215	43325	CG5590	15.31	15.42	15.53	15.45	14.40	14.62	14.93	15.18	**-0.65**
A_09_P072416	FBtr0085216	43326	CG12883	9.46	9.65	9.59	9.71	8.94	8.99	8.92	8.64	**-0.73**
A_09_P072421	FBtr0306924	43329	CG43320	8.04	8.07	8.01	8.08	7.80	7.89	7.79	7.89	**-0.21**
A_09_P072461	FBtr0085270	43347	CG12259	9.98	9.85	9.79	9.92	9.05	8.80	9.12	9.30	**-0.82**
A_09_P072481	FBtr0081829	40976	CG11671	7.95	7.19	7.51	6.63	10.80	10.64	10.25	9.57	**2.99**
A_09_P072486	FBtr0081865	40977	CG11672	12.05	12.21	12.34	12.46	11.40	11.08	11.23	11.30	**-1.01**
A_09_P072531	FBtr0081834	40987	eIF4AIII	12.67	12.67	12.65	12.69	12.08	11.76	12.25	12.50	**-0.53**
A_09_P072561	FBtr0081841	40996	DppIII	10.90	11.23	10.98	11.15	9.33	8.54	9.86	10.23	**-1.57**
A_09_P072596	FBtr0081900	41007	Or85b	7.17	6.88	6.87	6.78	5.93	6.30	6.38	6.55	**-0.63**
A_09_P072626	FBtr0081893	41019	Cyp313b1	6.55	7.15	6.63	6.95	8.01	8.42	7.75	7.48	**1.09**
A_09_P072641	FBtr0081880	41026	CG8032	10.10	10.10	10.17	10.04	9.58	9.60	9.60	9.70	**-0.48**
A_09_P072661	FBtr0081902	41030	CG8043	11.12	10.72	11.05	11.10	10.62	10.21	10.60	10.73	**-0.46**
A_09_P072681	FBtr0081905	41035	CG8116	9.30	9.35	9.14	8.96	8.87	8.79	8.47	8.76	**-0.46**
A_09_P072686	FBtr0081907	41036	CG11760	10.95	10.68	10.79	10.78	10.21	10.15	10.21	10.13	**-0.62**
A_09_P072706	FBtr0301194	41040	CG8159	8.71	8.90	8.70	8.80	7.76	8.28	8.26	8.58	**-0.56**
A_09_P072746	FBtr0081942	41048	CG13318	9.23	8.89	9.23	9.06	9.97	10.42	9.90	9.64	**0.88**

A_09_P072761	FBtr0081918	41054	Tcp-1eta	14.33	14.17	14.22	14.09	13.55	13.77	13.94	13.99	-0.39
A_09_P072771	FBtr0081919	41056	CG8359	7.66	7.51	7.52	7.65	6.88	7.05	7.06	7.10	-0.56
A_09_P072781	FBtr0081932	41058	CG9821	10.67	11.19	10.67	10.75	12.59	12.40	11.80	11.76	1.32
A_09_P072786	FBtr0081930	41059	CG9836	12.48	12.57	12.54	12.59	12.85	12.95	12.77	12.75	0.29
A_09_P072791	FBtr0081922	41060	CG8379	10.34	10.47	10.57	10.42	10.28	10.15	10.17	10.30	-0.22
A_09_P072816	FBtr0081958	41070	CAHbeta	10.58	10.10	10.49	10.26	9.46	9.88	9.68	9.85	-0.64
A_09_P072836	FBtr0081962	41078	CG11980	13.24	12.94	12.95	13.00	12.50	12.41	12.66	12.85	-0.43
A_09_P072906	FBtr0302133	41100	CG16734	10.00	9.89	9.91	9.94	9.22	8.95	9.49	9.72	-0.59
A_09_P072916	FBtr0081984	41102	CG9740	10.71	10.64	10.66	10.72	9.62	9.62	10.10	10.35	-0.76
A_09_P072921	FBtr0081977	41103	CG8436	10.28	10.40	10.30	10.38	9.65	9.63	9.71	9.91	-0.61
A_09_P072936	FBtr0081979	41107	Vps16A	11.52	11.77	11.70	11.68	11.38	11.34	11.33	11.47	-0.29
A_09_P072956	FBtr0082057	41111	CG16749	14.90	14.39	14.10	14.13	15.65	15.53	15.45	15.05	1.04
A_09_P072966	FBtr0082019	41113	CG8121	9.10	9.13	9.09	9.20	9.26	9.35	9.34	9.27	0.17
A_09_P072976	FBtr0082026	41117	CG8129	9.61	10.64	10.14	10.67	9.23	9.12	8.99	9.06	-1.17
A_09_P073001	FBtr0082030	41123	CG8135	11.11	11.41	11.41	11.38	10.69	10.86	10.92	11.02	-0.46
A_09_P073016	FBtr0082044	41132	GstZ1	10.24	10.11	10.31	10.20	8.05	8.01	8.89	9.26	-1.66
A_09_P073031	FBtr0082061	41137	CG8149	11.55	11.36	11.50	11.46	10.94	10.94	11.00	11.15	-0.46
A_09_P073036	FBtr0082123	41138	rump	12.22	12.41	12.37	12.45	11.94	11.75	12.07	12.21	-0.37
A_09_P073066	FBtr0082067	41149	CG8199	12.96	13.15	13.08	13.03	12.01	11.96	12.20	12.51	-0.89
A_09_P073086	FBtr0082112	41155	CG16790	7.84	7.94	7.81	7.79	8.25	8.31	8.06	8.20	0.36
A_09_P073091	FBtr0082111	41156	CG9396	12.23	12.24	12.53	12.33	12.15	11.70	11.99	11.95	-0.38
A_09_P073101	FBtr0082072	41159	CG8273	8.36	8.29	8.37	8.39	7.26	7.17	7.37	7.63	-1.00
A_09_P073106	FBtr0082073	41160	CG8301	7.60	6.56	7.18	6.68	8.04	8.32	7.95	7.91	1.05
A_09_P073111	FBtr0082074	41161	P58IPK	13.41	13.55	13.50	13.40	12.18	12.02	12.86	13.25	-0.88
A_09_P073116	FBtr0100537	41162	bocksbeutel	10.51	10.28	10.46	10.39	10.76	10.80	10.84	10.68	0.36
A_09_P073131	FBtr0082077	41165	CG8319	10.13	10.21	10.22	10.33	8.95	9.29	9.10	9.09	-1.11
A_09_P073136	FBtr0082079	41167	SpdS	14.94	14.58	14.84	14.63	13.95	14.23	14.28	14.50	-0.51
A_09_P073151	FBtr0082081	41172	CG8358	5.62	5.72	5.82	5.53	6.72	7.81	7.17	7.17	1.54
A_09_P073156	FBtr0082082	41173	CG16817	13.40	13.35	13.57	13.38	13.06	12.98	12.98	13.09	-0.40
A_09_P073166	FBtr0082084	41176	CG18542	10.42	10.62	10.61	10.51	9.79	9.89	10.11	10.34	-0.51
A_09_P073191	FBtr0113210	41185	Pnn	10.11	10.10	10.19	10.11	10.00	9.79	9.94	10.00	-0.19

A_09_P073201	FBtr0082166	41189	CG16908	9.50	9.60	9.54	9.54	9.76	9.79	9.85	9.87	0.27
A_09_P073211	FBtr0082130	41191	CG8412	12.39	12.69	12.66	12.81	11.10	11.54	11.81	11.89	-1.05
A_09_P073216	FBtr0082131	41192	CG8417	13.46	13.52	13.52	13.58	12.20	12.23	12.63	12.92	-1.02
A_09_P073236	FBtr0110789	41198	Whamy	10.87	10.55	10.63	10.52	12.98	12.58	12.35	11.74	1.77
A_09_P073271	FBtr0082140	41205	CG8507	11.57	11.62	11.52	11.60	10.78	10.55	10.80	11.13	-0.76
A_09_P073296	FBtr0082143	41210	CG8534	9.77	11.06	10.87	10.94	5.58	6.71	7.35	8.11	-3.72
A_09_P073311	FBtr0082153	41213	CG9459	8.38	8.68	8.49	8.41	5.72	6.39	7.17	7.73	-1.74
A_09_P073316	FBtr0082152	41214	CG9458	10.34	10.84	10.79	10.63	8.80	9.23	9.26	9.46	-1.46
A_09_P073321	FBtr0082151	41215	Teh1	6.77	6.14	6.13	5.83	7.32	7.81	7.41	7.51	1.30
A_09_P073331	FBtr0082150	41219	Art4	9.16	9.03	9.03	9.15	8.49	8.39	8.48	8.60	-0.60
A_09_P073346	FBtr0082205	41227	CG11722	11.88	11.45	11.63	11.49	11.12	11.07	11.22	11.36	-0.42
A_09_P073356	FBtr0082204	41229	CG12811	12.82	13.07	12.84	12.96	11.79	11.81	12.18	12.39	-0.88
A_09_P073366	FBtr0082203	41231	Fancl	10.53	10.62	10.45	10.59	11.40	11.31	11.05	10.84	0.60
A_09_P073386	FBtr0082179	41238	CG3940	13.13	13.62	13.51	13.46	12.97	12.27	12.63	12.58	-0.82
A_09_P073436	FBtr0082225	41253	CG3999	12.34	12.31	12.34	12.21	9.87	10.35	10.99	11.42	-1.64
A_09_P073466	FBtr0082230	41261	CG12818	10.23	10.34	10.49	10.38	9.76	9.74	9.96	10.09	-0.47
A_09_P073471	FBtr0082231	41262	sle	9.86	10.18	10.14	10.25	9.83	9.65	9.62	9.64	-0.42
A_09_P073491	FBtr0082260	41268	CG6325	8.03	8.19	8.38	8.35	7.99	7.68	7.71	7.66	-0.48
A_09_P073516	FBtr0113215	41275	CG14688	11.49	11.66	11.52	11.64	9.81	10.18	10.38	10.71	-1.31
A_09_P073551	FBtr0082314	41287	CG14691	6.59	6.70	6.66	7.09	5.10	5.61	5.52	5.46	-1.34
A_09_P073656	FBtr0082335	41314	CG14695	7.44	7.90	8.22	7.93	10.37	10.10	9.84	9.44	2.06
A_09_P073696	FBtr0082341	41337	CG18577	7.70	7.10	7.27	6.61	9.36	8.94	8.65	8.12	1.60
A_09_P073706	FBtr0082372	41340	SdhC	15.12	15.00	15.19	15.03	14.55	14.43	14.71	14.81	-0.46
A_09_P073741	FBtr0082352	41349	CG17726	11.26	11.18	11.25	11.25	10.86	11.14	10.97	10.99	-0.25
A_09_P073746	FBtr0082366	41350	CG10703	11.66	11.60	11.56	11.65	11.39	11.25	11.43	11.44	-0.24
A_09_P073766	FBtr0082354	41354	CG17721	8.85	9.09	8.83	9.08	8.25	8.07	8.42	8.62	-0.62
A_09_P073781	FBtr0082361	41359	CG17734	14.11	14.25	14.22	14.28	13.75	13.82	13.52	13.35	-0.61
A_09_P073786	FBtr0082358	41360	CG5214	14.72	14.78	14.87	14.83	14.57	14.34	14.51	14.67	-0.28
A_09_P073826	FBtr0082403	41372	RpL24-like	14.02	13.81	14.04	13.92	14.93	15.23	14.81	14.62	0.95
A_09_P073831	FBtr0082390	41373	CG5276	10.30	10.43	10.48	10.66	10.05	10.01	9.95	10.01	-0.46
A_09_P073841	FBtr0082391	41375	CG5281	8.28	7.85	8.01	8.10	8.57	8.52	8.54	8.49	0.47

A_09_P073871	FBtr0100464	3772221	sea	14.07	13.94	13.96	13.87	13.61	13.46	13.67	13.79	-0.33
A_09_P073876	FBtr0100320	3772232	fabp	15.86	15.88	15.67	15.83	15.12	15.25	15.39	15.45	-0.51
A_09_P073891	FBtr0082414	41390	wkd	11.46	11.64	11.50	11.52	12.25	12.32	12.04	11.86	0.59
A_09_P073926	FBtr0299729	41398	Csk	10.61	10.63	10.62	10.68	11.53	11.63	11.49	10.88	0.75
A_09_P073971	FBtr0082437	41410	CG6908	11.47	11.75	11.52	11.52	11.27	11.28	11.06	10.85	-0.45
A_09_P073996	FBtr0082429	41416	CG12594	7.84	7.40	7.63	7.38	8.11	8.23	8.16	8.08	0.58
A_09_P074001	FBtr0082430	41417	CG14721	10.45	10.28	10.31	10.43	9.92	9.92	9.85	9.93	-0.46
A_09_P074011	FBtr0082432	41420	CG6923	9.68	9.94	9.92	9.89	9.28	9.39	9.48	9.72	-0.39
A_09_P074016	FBtr0110876	41423	l(3)neo38	7.68	8.64	8.14	8.62	7.30	7.46	7.24	7.19	-0.97
A_09_P074021	FBtr0082531	41425	CG17360	7.93	8.23	7.83	8.04	8.37	8.46	8.33	8.34	0.37
A_09_P074076	FBtr0113223	41447	CG12213	8.91	9.16	9.14	9.15	8.69	8.45	8.68	8.83	-0.43
A_09_P074081	FBtr0082484	41448	CG18347	11.43	11.61	11.54	11.65	11.29	11.04	10.99	10.82	-0.52
A_09_P074111	FBtr0082504	41454	CG3397	9.70	11.36	10.13	11.18	7.59	8.08	7.46	7.06	-3.04
A_09_P074126	FBtr0082557	41458	KLHL18	9.85	10.41	10.14	10.41	9.50	9.34	9.61	9.77	-0.65
A_09_P074131	FBtr0113224	41459	CG3532	9.76	9.90	9.95	10.00	9.59	9.58	9.56	9.62	-0.31
A_09_P074136	FBtr0082555	41460	CG3313	8.19	8.48	8.41	8.60	7.17	7.36	7.47	7.62	-1.02
A_09_P074191	FBtr0082625	41477	CG4830	15.01	15.20	15.41	14.68	14.57	14.17	14.38	14.49	-0.67
A_09_P074196	FBtr0082624	41479	CG4848	9.90	10.06	9.96	10.12	9.76	9.66	9.72	9.83	-0.27
A_09_P074201	FBtr0082623	41480	CG4860	11.87	12.20	12.09	12.12	11.22	11.35	11.58	11.88	-0.56
A_09_P074311	FBtr0082600	41518	lig3	9.28	9.35	9.39	9.30	10.22	10.28	10.09	10.13	0.85
A_09_P074321	FBtr0082583	41521	CG5167	13.66	13.81	13.69	13.80	13.06	13.09	13.30	13.50	-0.50
A_09_P074331	FBtr0082596	41525	Scgbeta	9.53	9.11	9.30	9.17	9.70	9.59	9.77	9.78	0.43
A_09_P074386	FBtr0082681	41541	CG5961	9.73	9.55	9.63	9.60	10.60	10.68	10.22	10.03	0.75
A_09_P074411	FBtr0082641	41551	Snx3	13.06	13.43	13.31	13.45	12.69	12.82	12.88	12.99	-0.47
A_09_P074451	FBtr0082651	41561	CG6188	13.65	12.92	13.51	13.41	14.78	14.68	14.51	14.20	1.17
A_09_P074466	FBtr0113226	41568	NijC	7.85	8.37	7.75	8.29	6.77	7.28	6.70	6.28	-1.31
A_09_P074476	FBtr0113228	41568	NijC	9.32	10.06	9.49	9.95	8.81	9.17	8.63	8.35	-0.97
A_09_P074481	FBtr0082656	41570	CG12279	12.72	12.82	12.83	12.89	11.97	11.96	12.16	12.29	-0.72
A_09_P074486	FBtr0082657	41573	CG5724	9.97	9.77	9.87	9.36	8.50	8.23	8.76	8.93	-1.14
A_09_P074491	FBtr0302379	41574	CG5999	9.47	10.57	10.04	9.99	8.06	8.08	8.59	8.84	-1.63
A_09_P074521	FBtr0082684	41583	beat-Vb	6.34	5.77	5.64	5.47	6.35	6.83	6.89	6.71	0.89

A_09_P074546	FBtr0082693	41591	Paip2	10.39	10.84	10.47	10.47	12.23	12.68	11.57	11.76	1.51
A_09_P074586	FBtr0082719	41601	CG8031	11.22	11.54	11.15	11.39	11.07	10.92	10.92	10.95	-0.36
A_09_P074591	FBtr0082717	41603	CG12360	9.58	9.78	9.78	9.97	9.38	8.90	9.13	9.15	-0.64
A_09_P074611	FBtr0082715	41610	CG7966	13.76	14.02	13.84	14.03	12.88	12.78	12.93	13.21	-0.96
A_09_P074691	FBtr0082775	41633	wntD	7.47	6.73	7.02	6.93	9.60	9.87	8.92	8.10	2.09
A_09_P074696	FBtr0290037	41634	CG8773	14.26	13.70	13.56	13.36	15.61	15.58	15.38	15.04	1.68
A_09_P074701	FBtr0082740	41635	CG8774	13.98	13.79	14.06	13.81	14.88	14.73	14.54	14.16	0.67
A_09_P074706	FBtr0082773	41638	CG8795	6.06	5.57	5.55	4.94	6.91	7.20	6.76	6.73	1.37
A_09_P074741	FBtr0082762	41648	CCHa2	10.89	11.10	10.86	11.04	10.58	10.38	10.58	10.56	-0.45
A_09_P074761	FBtr0302576	41652	yellow-e2	10.45	10.98	10.51	10.92	9.41	9.89	9.05	8.80	-1.42
A_09_P074841	FBtr0082845	41679	Adgf-D	10.95	11.54	11.11	11.43	11.64	12.08	11.93	12.07	0.67
A_09_P074861	FBtr0082826	41687	Cht5	11.82	9.59	10.50	10.15	14.17	13.61	13.30	12.42	2.86
A_09_P074866	FBtr0082828	41688	CG9297	14.46	14.97	14.86	15.00	14.11	14.01	14.32	14.54	-0.58
A_09_P074931	FBtr0113232	41711	CCHa1	8.04	7.79	7.66	7.71	8.79	9.27	8.64	8.51	1.00
A_09_P074966	FBtr0082891	41719	twf	10.95	11.35	11.13	11.21	10.55	10.28	10.43	10.52	-0.72
A_09_P074991	FBtr0082902	41727	CG9649	8.08	7.94	8.18	8.30	10.06	10.02	9.49	8.99	1.52
A_09_P074996	FBtr0082897	41730	CG9624	5.65	7.09	6.73	7.12	4.07	4.97	3.86	4.47	-2.31
A_09_P075021	FBtr0082921	41738	CG12207	8.94	8.88	8.74	8.71	9.94	9.93	9.50	9.12	0.81
A_09_P075026	FBtr0082924	41739	CG3259	5.85	5.48	5.30	5.32	7.85	7.98	7.26	6.93	2.02
A_09_P075046	FBtr0082987	41756	HtrA2	7.75	7.54	7.67	7.58	7.05	6.65	7.02	7.16	-0.67
A_09_P075091	FBtr0082957	41765	CG14852	12.95	13.68	13.45	13.24	15.03	14.90	14.50	13.87	1.25
A_09_P075121	FBtr0082966	41777	CG3505	12.54	13.08	12.68	12.99	12.10	12.19	12.25	12.20	-0.64
A_09_P075191	FBtr0083000	41800	CG3731	15.23	15.23	15.24	15.23	15.02	15.01	15.09	15.13	-0.17
A_09_P075196	FBtr0083009	41802	CG7265	11.76	11.73	11.82	11.84	11.38	11.58	11.58	11.62	-0.25
A_09_P075216	FBtr0290022	41806	VhaPPA1-2	9.86	9.98	9.96	9.98	9.83	9.19	9.31	9.17	-0.57
A_09_P075226	FBtr0083004	41809	CG3837	6.83	7.21	6.85	6.98	6.04	5.70	5.90	6.33	-0.97
A_09_P075241	FBtr0089376	2768670	dpr9	7.29	6.76	6.69	6.22	8.05	8.58	7.97	7.93	1.39
A_09_P075266	FBtr0083025	41821	CG3984	9.51	9.73	9.34	9.32	7.54	7.68	8.21	8.75	-1.43
A_09_P075276	FBtr0083036	41823	CG6904	13.07	13.38	13.22	13.43	12.09	11.83	12.48	12.68	-1.01
A_09_P075281	FBtr0083031	41824	Mf	16.15	16.39	16.35	16.41	15.56	15.56	15.59	15.67	-0.73
A_09_P075291	FBtr0083034	41824	Mf	14.66	14.94	14.70	15.20	13.55	13.38	13.83	13.72	-1.25

A_09_P075326	FBtr0083049	41832	CG4210	10.83	10.64	10.77	10.71	11.08	11.02	11.32	11.41	0.47
A_09_P075361	FBtr0305613	41842	CG42404	8.06	8.38	8.08	8.07	8.89	9.39	8.64	8.80	0.79
A_09_P075371	FBtr0290018	41847	CG4334	10.09	11.01	10.02	10.70	8.43	8.80	9.02	9.43	-1.53
A_09_P075376	FBtr0083066	41849	CG4338	9.59	9.36	9.48	9.41	10.75	11.02	10.75	10.75	1.36
A_09_P075391	FBtr0083122	41857	CG6236	11.59	11.91	11.87	11.86	11.47	11.45	11.32	11.42	-0.39
A_09_P075426	FBtr0083091	41869	CG5044	13.66	13.90	13.75	13.83	12.60	12.40	12.61	12.69	-1.21
A_09_P075486	FBtr0083147	41891	CG5205	10.46	10.71	10.61	10.56	11.29	11.40	11.50	11.73	0.89
A_09_P075496	FBtr0334152	41893	CG44014	12.20	11.31	11.68	11.40	14.91	14.48	14.48	14.02	2.83
A_09_P075506	FBtr0083154	41894	CG18522	14.19	13.81	13.96	13.81	14.47	14.58	14.50	14.57	0.59
A_09_P075531	FBtr0083164	41900	CG5399	12.78	12.58	12.52	12.77	14.67	13.93	14.13	13.47	1.39
A_09_P075576	FBtr0083174	41910	Acyp2	9.81	9.97	9.93	10.10	9.45	9.50	9.51	9.57	-0.44
A_09_P075621	FBtr0083188	41925	Hmt-1	11.81	11.77	11.80	11.70	12.02	12.03	11.88	11.92	0.19
A_09_P075636	FBtr0083200	41931	CG3303	9.64	9.91	9.76	9.87	10.39	10.19	10.56	10.77	0.68
A_09_P075651	FBtr0083203	41937	blp	11.71	11.34	11.50	11.35	11.08	11.09	10.89	10.89	-0.48
A_09_P075656	FBtr0083240	41939	CG4287	11.87	12.05	11.93	12.07	11.61	11.59	11.72	11.83	-0.29
A_09_P075661	FBtr0083212	41940	CG5516	10.97	11.05	11.12	11.12	10.26	10.42	10.55	10.80	-0.56
A_09_P075666	FBtr0083213	41941	Rbf2	7.06	7.10	7.03	6.86	5.98	5.51	6.46	6.51	-0.90
A_09_P075671	FBtr0083218	41945	GATAe	8.66	7.97	8.19	7.68	10.01	9.50	9.37	8.92	1.33
A_09_P075691	FBtr0083223	41951	CG5013	8.77	8.77	8.82	8.70	7.50	7.75	7.91	8.18	-0.93
A_09_P075721	FBtr0083253	41963	CG8925	9.31	9.13	9.42	9.05	10.60	10.61	10.18	9.95	1.10
A_09_P075731	FBtr0083278	41967	CG6126	12.15	12.38	12.27	12.24	11.74	11.53	11.38	11.32	-0.77
A_09_P075781	FBtr0083323	8674030	CG17931	10.26	10.27	10.10	10.13	9.47	9.40	9.69	10.09	-0.53
A_09_P075816	FBtr0083314	41993	CG14894	11.62	11.77	11.77	11.80	11.39	11.32	11.38	11.41	-0.37
A_09_P075831	FBtr0083291	41997	CG14883	9.48	9.30	9.37	9.39	9.21	9.00	9.19	9.15	-0.25
A_09_P075866	FBtr0083307	42005	Der-2	12.24	12.47	12.33	12.35	11.79	11.53	11.86	12.03	-0.54
A_09_P075891	FBtr0336775	7354466	CG42342	7.38	7.46	7.28	7.06	7.79	8.04	8.02	8.03	0.67
A_09_P075921	FBtr0083329	42021	CG17562	9.07	10.81	9.94	10.41	5.40	6.37	7.60	8.21	-3.17
A_09_P075926	FBtr0083330	42022	CG17560	10.60	11.53	11.65	11.45	3.43	6.69	9.40	10.18	-3.88
A_09_P075931	FBtr0300865	42023	CG14893	9.29	10.68	10.44	10.73	3.59	5.17	4.32	4.47	-5.90
A_09_P075941	FBtr0083336	42027	CG10326	10.08	10.21	10.15	10.34	10.04	9.89	9.97	9.77	-0.28
A_09_P075951	FBtr0083354	42031	CG14907	9.31	8.76	9.20	8.86	10.90	10.88	10.55	10.08	1.57

A_09_P075986	FBtr0083372	42050	CG8907	12.71	12.32	12.48	12.19	13.70	13.37	13.23	13.03	**0.91**
A_09_P076011	FBtr0083391	42056	CG5220	11.17	10.91	10.95	10.97	11.32	11.21	11.32	11.48	**0.33**
A_09_P076036	FBtr0083398	42063	kuk	10.38	11.30	11.00	11.30	10.46	10.13	9.95	9.77	**-0.92**
A_09_P076051	FBtr0083418	42067	CG17477	8.64	8.98	9.24	9.16	7.38	7.94	7.53	7.14	**-1.50**
A_09_P076056	FBtr0083417	42069	CG17475	11.84	12.24	12.23	12.54	11.31	11.18	10.89	10.48	**-1.25**
A_09_P076101	FBtr0083410	42079	CG5292	10.45	10.45	10.48	10.54	10.10	10.17	10.19	10.23	**-0.31**
A_09_P076116	FBtr0083453	42086	beat-IIa	8.25	7.93	8.06	7.76	9.19	9.20	8.94	8.86	**1.05**
A_09_P076161	FBtr0083446	42098	CG14332	7.35	7.77	6.91	7.50	6.01	6.05	6.27	6.77	**-1.11**
A_09_P076186	FBtr0083455	42106	CG5840	11.81	11.87	11.81	11.93	11.98	12.08	12.00	12.11	**0.19**
A_09_P076191	FBtr0083503	42109	Prx3	14.07	13.88	13.93	13.87	12.30	12.17	12.65	12.88	**-1.44**
A_09_P076236	FBtr0083470	42122	AttD	4.31	5.92	6.05	5.09	11.21	9.41	10.51	10.26	**5.00**
A_09_P076281	FBtr0083512	42135	TyrRII	7.31	7.21	6.97	7.15	5.41	5.34	5.94	6.35	**-1.40**
A_09_P076321	FBtr0075207	39959	Jon74E	8.81	9.01	8.24	8.29	8.12	6.91	7.43	7.11	**-1.19**
A_09_P076336	FBtr0083947	42435	Elongin-B	12.96	12.94	13.04	13.07	12.50	12.45	12.61	12.62	**-0.46**
A_09_P076341	FBtr0112904	43839	eIF4G	14.01	14.15	14.13	14.15	13.61	13.52	13.72	13.91	**-0.42**
A_09_P076361	FBtr0307279	31329	Parg	9.32	9.60	9.64	9.64	8.73	8.96	9.02	9.23	**-0.56**
A_09_P076371	FBtr0074998	40111	Chd3	6.53	6.60	6.69	6.76	6.41	5.77	5.92	5.80	**-0.67**
A_09_P076386	FBtr0085897	35419	Ac3	8.72	8.47	8.74	8.54	9.03	8.95	9.18	9.21	**0.47**
A_09_P076421	FBtr0072141	37804	Tal	15.81	15.70	15.73	15.63	14.28	14.72	15.18	15.53	**-0.79**
A_09_P076426	FBtr0076526	39048	Tequila	13.28	13.53	13.45	13.44	13.71	13.64	13.67	13.59	**0.23**
A_09_P076461	FBtr0070317	31157	Ocrl	10.43	10.51	10.64	10.56	10.37	10.16	10.39	10.28	**-0.24**
A_09_P076466	FBtr0070315	31153	mip130	10.48	10.79	10.75	10.70	10.03	10.50	10.12	10.17	**-0.47**
A_09_P076481	FBtr0070351	31156	eIF2B-epsilon	8.58	8.63	8.51	8.47	8.19	8.13	8.29	8.41	**-0.29**
A_09_P076506	FBtr0070366	31143	Pgam5	12.40	12.40	12.37	12.40	11.85	11.78	12.05	12.23	**-0.41**
A_09_P076516	FBtr0332579	31150	mRpL16	12.48	12.18	12.41	12.19	11.88	11.93	11.93	12.08	**-0.36**
A_09_P076526	FBtr0070321	31160	CG3587	10.82	10.71	10.79	10.74	10.96	11.18	11.06	11.06	**0.30**
A_09_P076551	FBtr0332962	31217	CG2865	10.56	9.71	10.30	9.99	11.82	12.05	11.49	11.19	**1.49**
A_09_P076591	FBtr0070088	46717	arg	12.47	12.64	12.78	12.81	11.36	11.73	11.87	12.23	**-0.88**
A_09_P076601	FBtr0070093	30995	CG17896	15.73	15.62	15.74	15.68	15.46	15.44	15.40	15.35	**-0.28**
A_09_P076611	FBtr0070426	45706	Cyp4d14	9.20	8.89	8.88	8.77	8.60	8.32	8.18	8.09	**-0.64**
A_09_P076616	FBtr0070425	31195	Nmd3	11.38	10.75	11.12	10.80	12.09	12.12	11.61	11.52	**0.82**

A_09_P076621	FBtr0070347	31163	CG4406	11.97	12.29	12.12	12.20	11.81	11.68	11.80	12.00	**-0.33**
A_09_P076676	FBtr0088126	44724	san	11.73	11.38	11.56	11.49	10.95	10.75	11.01	11.18	**-0.57**
A_09_P076691	FBtr0073029	44098	rasp	8.44	8.70	8.46	8.57	8.08	7.97	8.18	8.28	**-0.42**
A_09_P076706	FBtr0304804	44432	ird5	7.70	7.97	7.92	7.82	8.50	8.51	8.28	8.23	**0.53**
A_09_P076711	FBtr0080180	34504	ial	11.09	11.72	11.47	11.80	9.25	10.65	10.69	10.69	**-1.20**
A_09_P076716	FBtr0081288	44433	Hs2st	9.69	9.83	9.81	10.15	8.62	8.72	8.93	9.04	**-1.04**
A_09_P076736	FBtr0074441	32721	Fim	11.42	11.35	11.53	11.08	12.46	12.61	11.98	11.94	**0.91**
A_09_P076756	FBtr0077531	49638	drm	10.46	10.30	10.49	10.29	11.10	11.13	10.86	10.64	**0.55**
A_09_P076796	FBtr0072645	43974	trio	7.36	7.18	7.23	7.20	8.08	8.28	8.05	8.11	**0.89**
A_09_P076801	FBtr0072649	43974	trio	11.04	11.03	10.94	11.00	11.97	11.51	11.58	11.42	**0.62**
A_09_P076831	FBtr0100356	45040	Spn43Ab	13.52	13.74	13.67	13.73	14.35	14.14	14.17	14.18	**0.54**
A_09_P076846	FBtr0077980	43978	Plap	12.28	12.55	12.41	12.57	11.67	11.79	11.98	12.14	**-0.56**
A_09_P076866	FBtr0082941	41747	NK7.1	9.02	9.53	9.29	9.28	10.54	10.82	9.96	9.99	**1.05**
A_09_P076876	FBtr0331353	42253	Mekk1	7.31	7.58	6.86	7.22	8.06	8.12	7.78	7.79	**0.70**
A_09_P076881	FBtr0082162	44728	MED6	9.24	9.14	9.18	9.20	9.12	8.96	8.91	8.95	**-0.20**
A_09_P076886	FBtr0070762	31449	Mcm3	10.92	11.36	10.97	11.29	10.36	10.49	10.48	10.59	**-0.66**
A_09_P076901	FBtr0070211	31080	Tsp2A	11.05	10.67	10.92	10.65	12.18	11.73	11.70	11.43	**0.94**
A_09_P076941	FBtr0083575	42199	Dlc90F	8.98	9.02	9.03	8.84	8.49	8.38	8.32	8.41	**-0.57**
A_09_P076951	FBtr0084409	44437	sec13	9.47	9.64	9.56	9.50	8.58	8.84	8.94	9.34	**-0.62**
A_09_P076961	FBtr0076835	38795	Neos	9.54	9.61	9.68	9.83	9.36	9.14	9.30	9.35	**-0.38**
A_09_P076986	FBtr0089258	44250	Cpsf160	10.40	10.37	10.36	10.39	10.04	10.11	10.12	10.26	**-0.25**
A_09_P077036	FBtr0072520	252479	DIP2	10.39	10.68	10.64	10.74	10.21	10.07	10.32	10.20	**-0.41**
A_09_P077046	FBtr0089113	43775	Crk	11.65	11.78	11.66	11.76	11.51	11.34	11.34	11.43	**-0.31**
A_09_P077056	FBtr0084253	42642	AP-50	11.67	11.75	11.70	11.70	11.11	10.93	11.35	11.52	**-0.48**
A_09_P077066	FBtr0088214	36125	stan	9.70	10.18	9.98	10.09	9.31	9.52	9.54	9.67	**-0.48**
A_09_P077086	FBtr0074881	40160	Kap-alpha1	9.20	9.10	9.15	8.95	8.94	8.64	8.64	8.65	**-0.38**
A_09_P077101	FBtr0081756	40934	Taf7	10.44	10.51	10.48	10.53	8.76	8.78	9.43	9.86	**-1.28**
A_09_P077106	FBtr0081697	40816	agt	7.05	6.85	6.78	7.00	9.16	9.35	9.28	9.28	**2.34**
A_09_P077116	FBtr0077679	33499	Ts	8.29	8.45	8.51	8.35	7.22	7.37	7.57	7.92	**-0.88**
A_09_P077126	FBtr0112906	36040	TER94	13.16	13.19	13.21	13.19	12.20	11.83	12.70	12.86	**-0.79**
A_09_P077146	FBtr0110936	43982	Oamb	5.88	5.56	5.34	5.03	7.00	7.42	6.43	6.64	**1.42**

A_09_P077156	FBtr0072483	38029	NitFhit	11.19	11.16	11.08	11.28	11.55	11.56	11.50	11.38	**0.32**
A_09_P077161	FBtr0077682	33495	NTPase	12.73	12.71	12.65	12.67	12.43	12.34	12.39	12.50	**-0.27**
A_09_P077181	FBtr0335418	42350	GluClalpha	4.23	4.25	2.91	3.02	5.01	5.64	5.50	5.46	**1.80**
A_09_P077196	FBtr0070488	31267	CG2712	8.48	8.44	8.49	8.37	8.12	8.21	8.13	8.34	**-0.24**
A_09_P077216	FBtr0100476	31001	CG4293	11.30	11.63	11.41	11.84	9.47	10.12	10.66	11.01	**-1.23**
A_09_P077226	FBtr0070225	31090	CG11448	9.79	9.91	9.89	10.01	10.84	10.88	10.51	10.19	**0.70**
A_09_P077246	FBtr0070068	30979	CG3777	12.73	12.44	12.70	12.25	11.26	11.64	11.75	12.10	**-0.84**
A_09_P077261	NM_130662	31254	CG2662	6.40	6.23	6.52	6.34	5.20	5.77	5.89	5.90	**-0.68**
A_09_P077266	FBtr0070501	31255	Csat	11.18	11.39	11.06	11.47	10.29	10.13	10.50	10.71	**-0.87**
A_09_P077271	FBtr0273279	31257	CG2680	12.03	11.98	12.21	11.97	10.82	11.15	11.54	11.87	**-0.70**
A_09_P077276	FBtr0070479	31256	eIF2B-beta	10.80	10.90	10.90	10.72	10.15	10.03	10.37	10.62	**-0.54**
A_09_P077291	AF069781	44441	Bem46	8.23	8.57	8.23	8.44	7.81	8.04	7.92	8.03	**-0.42**
A_09_P077351	FBtr0076203	45739	SuUR	9.56	9.36	9.57	9.26	9.05	9.01	9.10	9.03	**-0.39**
A_09_P077356	FBtr0088865	44108	Optix	7.59	8.05	7.98	8.13	7.33	7.43	7.30	7.35	**-0.59**
A_09_P077361	FBtr0080017	34419	Ip259	14.61	14.74	14.69	14.65	14.91	14.81	14.85	15.01	**0.22**
A_09_P077386	FBtr0070257	31102	CG14780	9.12	9.19	9.33	9.12	8.72	8.74	8.53	8.63	**-0.54**
A_09_P077436	FBtr0085465	43490	Bub3	10.62	10.90	10.67	10.86	9.89	9.87	10.09	10.24	**-0.74**
A_09_P077451	FBtr0088824	35773	slv	10.38	10.43	10.45	10.54	9.85	9.86	10.14	10.33	**-0.41**
A_09_P077461	FBtr0072639	44254	bab2	9.74	9.81	9.51	9.60	10.70	10.80	10.46	10.31	**0.91**
A_09_P077476	FBtr0083456	43912	SF1	11.90	12.02	12.07	12.15	11.34	11.48	11.68	11.70	**-0.49**
A_09_P077491	FBtr0072430	37963	Lcp9	13.98	15.13	15.02	15.21	12.37	13.38	12.12	10.92	**-2.63**
A_09_P077521	FBtr0079309	33942	GRHR	11.11	11.70	11.86	11.84	9.29	9.64	10.42	10.94	**-1.56**
A_09_P077536	FBtr0070693	31395	CG3081	7.36	7.15	7.10	7.03	6.82	6.63	6.65	6.87	**-0.42**
A_09_P077556	FBtr0070147	31025	CG13360	9.99	10.44	9.94	10.45	7.29	7.72	7.12	7.33	**-2.84**
A_09_P077621	FBtr0070121	31013	CG13367	9.96	10.00	9.98	9.99	10.55	10.31	10.20	10.22	**0.34**
A_09_P077626	FBtr0070116	47718	CG17829	9.13	9.20	9.13	9.07	8.87	8.73	8.78	8.70	**-0.37**
A_09_P077631	FBtr0070124	31016	skpA	13.51	13.80	13.62	13.79	13.33	13.25	13.28	13.47	**-0.35**
A_09_P077636	FBtr0308651	31014	Roc1a	9.74	9.94	9.58	9.76	9.26	9.11	9.23	9.37	**-0.51**
A_09_P077661	FBtr0301061	31278	CG3588	15.08	15.19	15.12	15.35	15.83	16.19	15.93	15.87	**0.77**
A_09_P077721	FBtr0078134	33199	CG11592	9.86	10.51	10.21	10.23	9.90	8.65	8.90	8.29	**-1.27**
A_09_P077731	FBtr0087814	36418	CG3814	13.18	12.62	12.64	12.48	13.92	13.54	13.58	13.44	**0.89**

A_09_P077771	FBtr0300559	43787	PMCA	11.90	11.88	12.18	11.96	11.43	11.10	11.35	11.32	-0.68
A_09_P077786	FBtr0072761	38203	CG13920	12.42	12.43	12.60	12.67	11.63	11.49	11.95	12.33	-0.68
A_09_P077816	FBtr0089247	43841	unc-13	8.26	8.50	8.39	8.58	8.81	9.17	8.85	8.70	0.45
A_09_P077831	FBtr0089224	43832	plexA	10.70	11.12	11.06	11.18	10.60	10.56	10.60	10.49	-0.45
A_09_P077846	FBtr0333875	2768974	ind	6.23	6.48	5.99	5.96	5.24	4.27	5.13	5.36	-1.16
A_09_P077851	FBtr0079306	33944	homer	6.86	7.21	7.18	6.89	6.34	6.00	6.34	6.29	-0.79
A_09_P077856	FBtr0084377	42753	cdc16	7.87	7.97	7.84	8.00	6.72	6.71	7.07	7.64	-0.88
A_09_P077901	FBtr0074190	32529	Paf-AHalpha	8.76	9.06	8.82	9.01	8.17	8.10	8.37	8.56	-0.62
A_09_P077916	FBtr0070952	31603	Mcm6	11.16	11.33	11.15	11.44	10.55	10.56	10.78	10.89	-0.57
A_09_P077941	FBtr0087036	36886	IntS8	9.45	9.49	9.29	9.46	9.04	9.07	9.12	9.30	-0.29
A_09_P077951	FBtr0087071	36896	CG8910	7.11	6.82	6.79	6.28	7.63	7.98	7.72	7.77	1.03
A_09_P077976	FBtr0070386	31190	CG3621	11.84	11.96	11.94	11.96	11.42	11.19	11.48	11.52	-0.52
A_09_P077991	FBtr0076343	39165	CalpB	11.29	11.33	11.32	11.17	12.98	12.85	12.64	12.32	1.42
A_09_P078001	FBtr0071821	37555	wrapper	8.04	8.24	8.19	8.30	7.40	7.89	7.63	7.55	-0.57
A_09_P078006	FBtr0082245	41248	Timp	11.25	11.79	11.54	11.88	13.25	12.86	12.69	12.49	1.21
A_09_P078011	FBtr0089329	35671	Inos	14.14	14.08	14.02	14.08	13.49	13.43	13.68	13.68	-0.51
A_09_P078016	FBtr0089084	43803	Eph	10.14	10.18	10.39	10.42	9.73	9.47	9.87	9.67	-0.60
A_09_P078026	FBtr0070930	31565	Top3beta	12.44	12.52	12.49	12.55	12.79	13.21	12.82	12.82	0.41
A_09_P078046	FBtr0083231	41955	Mhcl	10.21	11.03	10.85	11.05	9.75	9.73	9.63	9.84	-1.05
A_09_P078051	FBtr0110974	41955	Mhcl	8.77	8.59	8.69	8.62	9.32	9.28	9.18	9.22	0.58
A_09_P078101	FBtr0070643	31363	tyf	9.34	9.66	9.61	9.65	8.88	9.26	9.10	9.29	-0.43
A_09_P078126	FBtr0070364	31147	CG14818	13.39	13.76	13.62	13.79	13.16	13.18	13.07	13.15	-0.50
A_09_P078131	FBtr0070365	31145	CG14817	12.59	12.43	12.36	12.47	11.78	11.79	11.89	12.20	-0.55
A_09_P078151	FBtr0070182	31052	CDC45L	9.69	9.62	9.61	9.55	9.11	9.26	9.23	9.35	-0.38
A_09_P078166	FBtr0304948	47729	CG16833	8.91	8.84	8.67	8.65	8.01	8.24	8.40	8.62	-0.45
A_09_P078176	FBtr0071358	31890	BCL7-like	8.59	8.77	8.48	8.72	7.80	7.89	8.01	8.33	-0.63
A_09_P078236	FBtr0078290	40327	siz	9.84	9.85	9.75	9.78	10.52	10.60	10.29	10.09	0.57
A_09_P078306	FBtr0089094	43811	myo	10.04	10.31	10.22	10.23	9.59	9.42	9.47	9.47	-0.72
A_09_P078326	FBtr0309849	35478	gus	11.42	11.52	11.50	11.55	11.22	11.12	11.23	11.24	-0.29
A_09_P078391	FBtr0082375	41333	Ugt35b	7.89	9.46	9.29	9.74	5.03	6.45	6.71	7.22	-2.74
A_09_P078396	FBtr0082374	41334	Ugt35a	12.22	12.16	11.95	12.09	12.78	12.55	12.52	12.45	0.47

A_09_P078401	FBtr0089553	37035	UbcD10	12.08	12.38	12.23	12.39	11.78	11.94	11.82	11.92	**-0.40**
A_09_P078406	FBtr0084546	42862	Tsc1	10.04	10.03	10.17	10.14	9.69	9.95	9.82	9.78	**-0.29**
A_09_P078416	FBtr0077429	33638	Traf4	7.49	7.83	7.56	7.75	8.94	9.20	8.76	8.69	**1.24**
A_09_P078431	FBtr0077735	33468	Taf10b	11.88	11.91	11.95	12.05	11.29	11.34	11.53	11.65	**-0.50**
A_09_P078451	FBtr0087384	36706	SRPK	12.01	12.27	12.18	12.26	11.57	11.73	11.79	11.93	**-0.43**
A_09_P078471	FBtr0074096	32531	Rhp	8.95	8.84	8.64	8.50	9.10	9.33	9.00	9.20	**0.43**
A_09_P078576	FBtr0085320	43384	Noa36	12.96	13.07	13.11	13.14	12.60	12.74	12.81	12.99	**-0.28**
A_09_P078596	FBtr0076347	39173	Nc	9.70	9.55	9.46	9.44	11.26	11.08	10.83	10.45	**1.37**
A_09_P078616	FBtr0078876	40598	Hus1-like	7.92	7.45	7.57	7.13	8.62	8.55	8.52	8.57	**1.05**
A_09_P078621	FBtr0075773	39557	Hsc70Cb	12.58	12.79	12.66	12.77	11.79	11.62	12.00	12.25	**-0.79**
A_09_P078636	FBtr0074775	32946	Grip84	10.23	10.23	10.20	10.40	9.98	9.85	10.01	10.14	**-0.27**
A_09_P078656	FBtr0301316	33247	Eaat2	7.81	7.75	7.66	7.70	8.05	8.29	8.05	8.08	**0.39**
A_09_P078661	FBtr0079801	34251	Eaat1	9.87	10.08	10.10	10.06	9.35	9.11	9.07	8.80	**-0.95**
A_09_P078666	FBtr0083082	44451	ear	8.12	8.28	8.32	8.30	7.23	7.58	7.46	7.49	**-0.81**
A_09_P078696	FBtr0081699	40812	CG1315	9.73	8.96	8.90	8.65	10.05	9.92	9.87	9.86	**0.87**
A_09_P078706	FBtr0081687	40815	twr	14.68	14.86	14.80	14.73	13.34	13.37	13.91	14.39	**-1.01**
A_09_P078711	FBtr0072915	48613	CG5704	10.05	9.72	9.87	9.73	10.25	10.26	10.29	10.35	**0.45**
A_09_P078766	FBtr0085553	43565	Axn	10.29	10.53	10.61	10.57	10.26	10.31	10.15	10.24	**-0.26**
A_09_P078801	FBtr0075019	40093	nes	11.50	11.33	11.84	11.43	11.12	10.90	10.91	10.88	**-0.58**
A_09_P078806	FBtr0083114	41870	ldlCp	7.54	7.88	7.45	7.62	8.20	8.48	8.49	8.66	**0.84**
A_09_P078841	FBtr0079703	34176	fu12	11.13	11.33	11.18	10.98	10.13	10.08	10.50	10.75	**-0.79**
A_09_P078851	FBtr0088850	35747	drosha	9.55	9.77	9.67	9.67	9.41	9.43	9.39	9.48	**-0.24**
A_09_P078861	FBtr0083113	41872	CG6171	10.08	10.16	10.29	10.35	10.90	11.19	11.01	11.08	**0.83**
A_09_P078866	FBtr0075605	39671	CG7857	9.78	9.74	9.87	9.65	9.04	8.94	9.17	9.48	**-0.60**
A_09_P078921	FBtr0089121	43785	Rad23	12.15	12.27	12.04	12.24	11.88	11.61	11.85	11.93	**-0.36**
A_09_P078926	FBtr0077747	33440	Rad1	9.27	9.01	9.22	9.07	8.66	8.69	8.77	8.82	**-0.41**
A_09_P078941	FBtr0089106	43796	Thd1	9.04	9.05	8.98	9.07	8.70	8.56	8.47	8.25	**-0.54**
A_09_P078956	FBtr0114454	31140	MED18	9.95	9.99	10.04	10.19	9.21	9.45	9.65	9.68	**-0.55**
A_09_P078971	FBtr0070245	31072	CG11403	8.62	9.24	9.12	9.31	8.50	8.55	8.46	8.33	**-0.61**
A_09_P078976	FBtr0070342	31167	CG4325	6.87	8.61	7.61	8.57	5.15	5.22	5.24	4.98	**-2.77**
A_09_P078996	FBtr0083292	42000	CSN5	9.66	9.63	9.64	9.72	10.16	9.89	10.20	10.26	**0.47**

A_09_P079001	FBtr0088859	35759	CSN4	11.12	11.30	11.31	11.35	11.04	10.91	11.03	10.96	-0.28
A_09_P079011	FBtr0075074	40063	CSN1b	12.11	12.11	12.05	12.09	11.41	11.25	11.64	11.78	-0.57
A_09_P079051	FBtr0072948	38331	Aats-pro	10.45	10.18	10.29	10.12	9.60	9.50	9.79	10.07	-0.52
A_09_P079081	FBtr0075633	39644	Aats-gly	14.18	14.16	14.20	14.15	13.68	13.70	13.81	14.12	-0.34
A_09_P079091	FBtr0087234	36784	Aats-cys	12.59	12.42	12.53	12.53	12.18	12.01	12.26	12.42	-0.30
A_09_P079096	FBtr0074178	32539	Aats-arg	12.78	12.66	12.69	12.61	12.12	11.95	12.26	12.38	-0.51
A_09_P079106	FBtr0083283	41983	Manf	11.65	11.91	11.65	11.71	10.08	10.13	10.97	11.41	-1.08
A_09_P079111	FBtr0100406	3885567	Dyrk3	10.10	10.43	10.27	10.41	9.62	9.80	9.75	9.86	-0.54
A_09_P079121	FBtr0071034	33027	Inx7	11.39	10.66	10.97	10.70	12.34	11.83	11.83	11.47	0.94
A_09_P079141	FBtr0083328	42018	npf	7.61	6.72	7.12	6.77	8.35	8.33	8.27	8.37	1.27
A_09_P079146	FBtr0072504	38047	miple	9.11	9.07	8.86	9.02	9.45	9.12	9.35	9.50	0.34
A_09_P079171	FBtr0071129	31712	l(1)G0193	11.86	12.46	12.11	12.39	13.03	13.18	12.79	12.75	0.73
A_09_P079206	FBtr0071280	31811	l(1)G0020	9.61	8.98	9.15	9.11	9.89	10.14	9.77	9.72	0.67
A_09_P079216	FBtr0074616	32850	Rip11	10.52	10.86	10.52	10.77	11.60	11.78	11.42	11.26	0.85
A_09_P079256	FBtr0080590	44117	bgm	14.93	14.59	14.74	14.52	15.17	14.88	15.04	15.10	0.35
A_09_P079261	FBtr0087828	36383	Amph	11.48	11.28	11.31	11.21	10.82	10.47	10.85	10.95	-0.55
A_09_P079306	FBtr0083125	41850	MRG15	11.63	11.89	11.69	11.82	11.14	10.98	11.29	11.56	-0.51
A_09_P079341	FBtr0087983	36299	CG8378	9.59	9.70	9.54	9.57	9.08	9.06	9.09	9.02	-0.54
A_09_P079346	FBtr0079261	33908	epsilonCOP	12.90	12.96	12.83	13.00	12.13	11.91	12.52	12.77	-0.59
A_09_P079351	FBtr0078722	40710	Madm	8.62	8.98	8.92	9.00	8.17	8.17	8.40	8.35	-0.61
A_09_P079356	FBtr0073431	32042	CG2061	8.65	9.01	8.95	9.15	8.34	8.24	8.40	8.48	-0.57
A_09_P079366	FBtr0075363	39850	spd-2	8.38	9.12	8.91	9.09	8.24	8.13	8.24	8.24	-0.67
A_09_P079371	FBtr0079268	33903	mmy	13.54	13.49	13.58	13.38	13.09	13.36	12.85	12.64	-0.51
A_09_P079391	FBtr0086958	36959	EDTP	11.07	10.95	10.88	10.96	11.55	11.45	11.45	11.42	0.50
A_09_P079401	FBtr0084890	43095	tankyrase	11.34	11.39	11.38	11.22	11.81	11.72	11.58	11.64	0.36
A_09_P079411	FBtr0084477	42793	CG10254	10.54	10.84	10.92	10.90	9.59	9.73	10.10	10.28	-0.87
A_09_P079426	FBtr0079523	34069	CG7115	11.31	11.60	11.57	11.58	11.05	10.54	10.76	10.82	-0.72
A_09_P079441	FBtr0082172	41226	CG3909	12.10	12.09	12.11	12.11	11.38	11.24	11.60	11.83	-0.59
A_09_P079446	FBtr0088245	36146	CG7686	12.43	12.61	12.60	12.70	13.59	13.82	13.38	13.21	0.91
A_09_P079451	FBtr0084347	42726	Ublcp1	10.75	11.13	10.83	11.01	10.33	10.31	10.50	10.70	-0.47
A_09_P079491	FBtr0073828	32350	CG11092	11.73	11.55	11.79	11.81	11.19	11.05	11.38	11.41	-0.46

A_09_P079501	FBtr0084739	42956	CG5807	10.19	10.64	10.51	10.52	9.92	9.73	9.97	10.13	-0.53
A_09_P079521	FBtr0085630	43607	CG2217	5.80	5.65	4.67	5.75	9.23	8.69	8.31	7.29	2.91
A_09_P079571	FBtr0074344	32682	CG4928	15.11	14.98	15.24	14.93	15.80	15.95	15.68	15.52	0.67
A_09_P079611	FBtr0078238	40250	CG5498	10.18	10.43	10.26	10.43	11.33	11.46	11.17	10.99	0.91
A_09_P079621	FBtr0080054	34403	Cand1	10.32	10.47	10.42	10.52	10.26	10.07	10.20	10.25	-0.24
A_09_P079641	FBtr0089548	37028	CG5009	12.24	12.60	12.33	12.42	12.03	12.00	12.00	12.15	-0.35
A_09_P079646	FBtr0085106	43266	CG5815	10.74	10.95	10.87	10.93	10.21	10.07	10.31	10.65	-0.56
A_09_P079651	FBtr0084168	42561	GABA-B-R2	7.64	7.22	7.35	7.30	7.93	8.48	8.21	8.06	0.79
A_09_P079666	FBtr0088458	36020	CG1516	14.96	15.00	15.10	14.92	14.06	13.81	14.30	14.51	-0.82
A_09_P079676	FBtr0080228	34546	CG6230	10.76	10.66	10.73	10.73	10.08	10.01	10.34	10.59	-0.47
A_09_P079691	FBtr0088712	35866	CG8740	13.62	13.45	13.64	13.47	14.43	14.62	14.28	14.00	0.78
A_09_P079696	FBtr0080449	34728	CG5867	11.56	12.57	12.33	12.62	11.21	11.02	10.23	9.28	-1.84
A_09_P079701	FBtr0072528	38032	CG7028	10.34	10.35	10.38	10.33	9.97	9.65	9.95	10.06	-0.44
A_09_P079706	FBtr0070061	49953	CG14476	13.89	14.12	13.96	13.93	13.00	12.81	13.46	13.70	-0.73
A_09_P079711	FBtr0088495	36005	CG1688	6.71	6.11	6.31	5.32	7.96	8.28	7.60	7.74	1.79
A_09_P079716	FBtr0072436	37960	GstE12	11.61	11.94	11.71	11.85	11.52	11.28	11.31	11.22	-0.44
A_09_P079816	FBtr0077457	33648	morgue	10.49	10.80	10.58	10.65	10.24	10.07	10.19	10.31	-0.43
A_09_P079826	FBtr0080030	34437	LM408	13.80	13.18	13.07	13.01	14.57	14.54	14.46	14.33	1.21
A_09_P079841	FBtr0084738	42957	CG5808	9.19	9.19	9.09	9.24	8.78	8.94	8.94	9.07	-0.24
A_09_P079851	FBtr0085845	43751	Acf1	9.47	9.54	9.43	9.58	9.18	9.10	9.10	9.10	-0.38
A_09_P079861	FBtr0085681	43630	jdp	8.26	8.12	8.08	8.43	8.90	8.94	8.95	8.65	0.64
A_09_P079896	FBtr0340243	34175	U26	8.32	8.49	8.37	8.40	8.09	8.07	8.08	8.15	-0.30
A_09_P079906	FBtr0083717	42285	Prp18	8.84	8.80	8.69	8.80	8.98	9.03	8.90	9.13	0.23
A_09_P079911	FBtr0083712	42282	NP15.6	15.36	15.29	15.45	15.35	15.06	15.02	15.19	15.28	-0.23
A_09_P079916	FBtr0072478	38026	Mtch	13.33	13.43	13.45	13.49	12.94	12.85	13.02	13.09	-0.45
A_09_P079956	FBtr0086681	37116	Dp1	14.18	14.35	14.23	14.20	13.55	13.59	13.92	14.10	-0.45
A_09_P079966	FBtr0083660	42239	CstF-64	9.61	9.33	9.45	9.40	8.91	8.89	8.99	9.21	-0.44
A_09_P079976	FBtr0076001	39390	CAH2	12.37	11.63	11.84	11.73	14.56	14.76	14.10	13.31	2.29
A_09_P079986	FBtr0071223	31806	Ogg1	9.26	9.05	9.19	9.06	8.86	8.96	8.95	8.84	-0.24
A_09_P079991	FBtr0084912	43127	Tsp96F	10.06	10.36	10.48	10.31	8.84	8.67	8.85	8.94	-1.48
A_09_P080036	FBtr0080585	34813	nimB1	5.12	5.62	5.07	5.67	7.22	7.17	6.53	6.30	1.44

A_09_P080056	FBtr0078412	40349	ppl	13.16	13.50	13.45	13.55	11.83	11.88	12.36	12.67	-1.23
A_09_P090045	FBtr0290240	326158	CG31759	9.24	9.03	9.18	8.81	8.56	8.67	8.50	8.76	-0.44
A_09_P090070	NR_037746	10178964	CR42862	15.34	15.61	15.49	15.51	15.66	15.80	15.79	15.69	0.25
A_09_P090110	FBtr0073300	38545	nAcRbeta-64B	7.04	6.90	6.31	6.52	7.45	7.95	7.46	7.37	0.87
A_09_P090170	NM_141807	41360	CG5214	14.55	14.63	14.70	14.65	14.42	14.25	14.42	14.51	-0.23
A_09_P090175	FBtr0087831	36384	Galpha49B	8.26	8.81	8.33	8.63	7.69	7.89	7.79	7.83	-0.71
A_09_P090210	FBtr0331605	31310	dm	11.19	11.42	11.33	10.86	12.69	12.96	12.02	12.13	1.25
A_09_P090230	FBtr0114582	41217	Glut4EF	7.21	6.19	6.60	5.99	8.08	8.55	7.88	7.48	1.50
A_09_P090265	FBtr0073416	32009	Imp	11.42	11.46	11.64	11.28	12.06	12.48	11.84	12.08	0.66
A_09_P090320	FBtr0081931	41058	CG9821	9.86	10.13	9.97	9.99	11.35	11.35	10.79	10.40	0.99
A_09_P090395	FBtr0304955	36384	Galpha49B	8.49	8.99	8.55	8.74	9.44	9.33	9.10	9.19	0.58
A_09_P090575	FBtr0074545	32797	Frq1	8.56	8.10	8.02	8.07	9.35	9.42	9.21	9.18	1.10
A_09_P090660	U92431	43573	RpL32	15.88	15.83	15.87	15.92	16.02	16.10	16.11	16.09	0.20
A_09_P090675	FBtr0089510	48971	Atpalpha	14.40	14.55	14.56	14.48	13.96	13.92	14.07	14.11	-0.48
A_09_P090740	Y10016	38982	Nelf-E	9.11	9.23	9.22	9.22	8.49	7.95	8.49	8.64	-0.81
A_09_P090755	FBtr0082660	47173	Men	13.37	13.35	13.39	13.40	12.50	12.57	12.88	13.07	-0.62
A_09_P090985	FBtr0300447	42453	cdc2c	7.61	8.00	7.84	7.94	6.55	7.20	7.36	7.33	-0.74
A_09_P090990	FBtr0085733	43671	Aph-4	13.89	14.03	13.84	13.85	12.54	13.04	12.88	13.22	-0.99
A_09_P091085	FBtr0300584	8674087	CG42494	10.60	9.92	10.35	10.21	11.24	11.18	11.13	10.84	0.83
A_09_P091195	FBtr0100485	33386	GlyP	15.45	15.67	15.73	15.68	14.88	14.69	15.19	15.38	-0.60
A_09_P091200	AF080266	31781	Sptr	9.89	10.28	10.03	10.33	8.35	8.57	9.16	9.67	-1.19
A_09_P091210	FBtr0083304	42008	decay	12.87	12.98	12.67	12.94	13.94	13.97	13.60	13.15	0.80
A_09_P091290	FBtr0082660	47173	Men	14.55	14.46	14.59	14.51	13.75	14.04	14.15	14.32	-0.47
A_09_P091315	AF203725	40349	ppl	9.98	10.30	10.27	10.34	8.59	8.67	9.23	9.39	-1.25
A_09_P091405	NR_002489	3771936	pRNA:Psi28S-25	8.11	8.07	8.03	7.59	7.63	7.39	7.18	7.27	-0.58
A_09_P100015	FBtr0333306	31967	Psf3	8.09	8.19	8.05	8.08	7.56	7.64	7.78	7.95	-0.37
A_09_P100055	FBtr0112487	5740331	CG34291	13.38	13.06	13.32	13.43	13.49	14.00	13.87	13.90	0.52
A_09_P100060	FBtr0112480	5740234	CG34284	10.73	10.92	11.00	11.24	10.26	10.48	10.21	10.18	-0.69
A_09_P100090	FBtr0305215	36678	chn	8.06	8.19	7.75	8.18	7.65	7.68	7.44	7.53	-0.47
A_09_P100120	FBtr0071697	246532	CG30289	9.06	9.05	9.19	8.89	7.19	7.28	7.72	8.11	-1.47
A_09_P100190	FBtr0074238	32595	Cyp1	14.31	14.42	14.63	14.47	13.89	13.89	14.04	14.19	-0.45

A_09_P100340	FBtr0112391	5740481	CG34198	8.42	8.04	8.27	8.27	10.41	10.10	9.92	9.43	1.71
A_09_P100355	FBtr0071779	37543	Oatp58Da	9.42	10.22	10.04	10.25	8.36	8.43	8.94	9.39	-1.20
A_09_P100505	FBtr0086177	59177	Tsp42Eb	13.77	13.60	13.20	13.47	15.41	15.16	15.17	15.09	1.70
A_09_P100560	FBtr0070598	31335	HIP-R	12.88	13.23	12.92	13.16	12.02	12.04	12.25	12.37	-0.88
A_09_P100705	FBtr0081818	40950	CG2747	10.14	10.02	10.30	10.04	9.60	9.77	9.83	9.73	-0.39
A_09_P101025	FBtr0289990	36233	CG30022	11.59	11.52	11.65	11.63	11.76	11.74	11.74	11.79	0.16
A_09_P101040	FBtr0299881	3355150	AGO3	7.84	7.29	7.44	7.06	8.67	8.33	8.15	8.09	0.90
A_09_P101215	FBtr0075174	317900	edin	3.44	4.30	3.10	3.42	14.68	13.83	14.16	13.78	10.55
A_09_P101305	FBtr0290140	43136	CG42235	12.13	13.37	12.87	13.37	10.78	10.73	11.18	11.44	-1.90
A_09_P101325	FBtr0082565	41490	CG17738	14.68	15.09	14.97	15.45	16.62	16.87	16.45	15.56	1.33
A_09_P101380	FBtr0086186	246489	CG30157	8.24	8.15	8.11	8.19	7.47	7.28	7.77	8.05	-0.53
A_09_P101390	FBtr0079243	33874	CG9486	12.04	11.72	11.60	11.83	12.45	12.26	12.11	12.10	0.43
A_09_P101400	FBtr0303763	33230	rempA	6.66	7.01	6.68	7.03	7.27	7.64	7.33	7.43	0.57
A_09_P101415	FBtr0078052	318893	CG33127	13.53	12.88	12.89	12.69	14.31	14.03	13.82	13.58	0.93
A_09_P101425	CO189689	3771936	bRNA:Psi28S-25	7.32	7.39	7.25	7.11	5.96	6.45	5.90	6.20	-1.14
A_09_P101525	FBtr0078624	40714	jagn	11.85	11.73	11.87	11.89	11.20	10.98	11.34	11.34	-0.62
A_09_P101545	FBtr0075744	39577	CG13482	10.01	9.33	9.57	9.60	13.44	12.83	12.53	11.90	3.05
A_09_P101725	FBtr0112424	5740811	CG34231	7.99	7.86	8.06	8.25	6.51	6.97	7.26	7.56	-0.96
A_09_P101730	FBtr0112422	5740848	CG34229	10.22	10.11	10.22	10.22	9.72	9.82	9.89	9.98	-0.34
A_09_P101790	FBtr0112356	5740685	CG34165	12.73	11.30	12.09	11.84	14.65	14.94	14.29	13.39	2.32
A_09_P101850	FBtr0075960	39432	ver	6.61	6.98	6.56	6.27	9.18	9.41	9.40	9.64	2.80
A_09_P101890	FBtr0075483	39776	PDCD-5	12.56	12.52	12.48	12.59	11.66	11.84	11.95	12.13	-0.64
A_09_P102140	FBtr0081151	35180	CG17343	9.32	9.59	9.48	9.58	9.00	8.97	9.09	9.05	-0.46
A_09_P102165	FBtr0075868	39463	CG14120	12.71	11.39	11.91	11.55	14.93	14.95	14.61	14.17	2.78
A_09_P102255	FBtr0111126	3355131	cta	9.19	9.32	9.31	9.47	9.01	8.70	8.94	8.95	-0.42
A_09_P102260	FBtr0308291	3355118	CG40005	8.22	8.29	8.34	8.25	7.64	7.46	7.75	7.71	-0.64
A_09_P102430	FBtr0332953	31976	nocte	10.99	11.38	11.26	11.18	10.49	10.73	10.83	10.96	-0.45
A_09_P102610	FBtr0301072	40912	CG34127	7.30	7.23	7.04	6.41	8.32	8.49	7.91	7.90	1.16
A_09_P102630	FBtr0113226	41568	NijC	7.45	8.18	7.44	8.17	6.58	6.85	6.29	6.28	-1.31
A_09_P102720	FBtr0332336	44205	spen	8.56	8.58	8.10	8.54	9.20	9.17	9.04	8.82	0.61
A_09_P102745	FBtr0304570	49090	Rya-r44F	13.68	13.89	13.93	13.85	12.85	12.95	13.04	13.35	-0.79

A_09_P102815	FBtr0339463	36125	stan	9.51	9.84	9.78	9.90	9.07	9.32	9.38	9.49	-0.44
A_09_P102885	FBtr0307325	32158	cac	9.41	9.06	9.01	9.37	10.11	10.28	10.28	10.49	1.08
A_09_P102920	FBtr0305979	3772382	cp309	11.01	11.19	11.11	11.19	10.80	10.92	10.79	10.90	-0.27
A_09_P102975	FBtr0273382	42256	CG14299	10.17	10.73	10.50	10.69	11.06	11.08	10.91	10.80	0.44
A_09_P103020	FBtr0303274	35442	CG42748	10.28	10.43	10.20	10.36	10.78	10.50	10.70	10.78	0.37
A_09_P103075	FBtr0336766	43404	spg	7.48	7.44	7.30	7.15	8.26	8.38	7.92	7.71	0.72
A_09_P103160	FBtr0084295	42637	CG12499	10.84	10.50	10.54	10.52	11.08	11.34	10.96	10.99	0.49
A_09_P103260	FBtr0305182	31363	tyf	9.30	9.50	9.33	9.61	8.20	9.15	8.76	8.98	-0.66
A_09_P103280	FBtr0304601	39117	CG3280	8.49	8.42	8.33	8.58	9.04	9.53	9.12	8.91	0.69
A_09_P103425	FBtr0075064	40055	Ugt	11.37	11.75	11.49	11.64	10.00	9.94	10.74	11.19	-1.10
A_09_P103430	FBtr0073245	38516	ago	11.34	11.63	11.58	11.62	10.97	11.02	11.00	10.95	-0.56
A_09_P103480	FBtr0300351	41469	CG14741	8.07	7.77	7.98	7.82	8.10	8.20	8.24	8.19	0.27
A_09_P103570	FBtr0073457	32080	hop	9.75	9.75	9.75	9.83	9.22	9.35	9.53	9.33	-0.41
A_09_P103625	FBtr0332320	35225	Top2	11.67	12.21	11.98	12.18	11.59	11.22	11.52	11.60	-0.52
A_09_P103805	FBtr0089290	34831	smi35A	9.04	8.34	8.62	8.37	9.23	9.32	9.36	9.15	0.67
A_09_P103835	FBtr0334088	40439	CG14562	8.53	8.69	8.73	8.44	7.63	7.88	8.26	8.33	-0.57
A_09_P103880	FBtr0074693	32910	Ulp1	8.37	8.21	8.60	8.55	7.96	8.07	7.90	7.86	-0.48
A_09_P103920	FBtr0072466	38017	Pdk1	11.82	11.95	11.99	11.87	13.17	13.34	12.88	12.62	1.10
A_09_P103970	FBtr0301420	46017	l(2)01289	14.59	14.70	14.73	14.73	13.17	12.71	13.48	13.76	-1.41
A_09_P104170	FBtr0082290	41311	Fdh	11.76	11.84	11.75	11.87	10.77	10.57	10.83	10.94	-1.03
A_09_P104175	FBtr0089052	45908	nec	12.29	12.37	12.39	12.38	13.15	13.09	12.86	12.50	0.54
A_09_P104200	FBtr0290323	45339	net	7.60	7.66	7.58	7.30	6.25	5.93	6.62	7.10	-1.06
A_09_P104205	FBtr0071610	45280	cv-2	9.51	9.52	9.26	9.33	10.70	11.02	10.67	10.51	1.32
A_09_P104235	AF225902	44724	san	11.84	11.63	11.72	11.67	11.14	10.96	11.33	11.40	-0.51
A_09_P104310	FBtr0081306	44038	Hf	9.95	10.39	10.25	10.24	8.70	9.23	9.17	9.46	-1.07
A_09_P104330	FBtr0333605	43951	e(r)	9.45	9.36	9.08	9.36	8.31	8.67	8.77	8.93	-0.65
A_09_P104375	FBtr0334546	43670	CanA1	11.36	11.84	11.69	11.83	10.24	9.97	10.26	10.45	-1.45
A_09_P104425	FBtr0086182	35609	vimar	10.07	10.28	10.09	10.20	9.93	9.87	10.03	9.97	-0.21
A_09_P104450	FBtr0087699	36496	bbc	11.44	11.43	11.48	11.32	11.23	11.09	11.20	11.14	-0.25
A_09_P104455	FBtr0084377	42753	cdc16	9.37	9.47	9.25	9.40	8.46	8.21	8.75	9.01	-0.76
A_09_P104575	FBtr0080546	34803	cenG1A	9.64	9.71	9.59	9.59	10.98	10.92	10.48	10.13	1.00

A_09_P104655	FBtr0070762	31449	Mcm3	10.06	10.35	10.19	10.51	9.57	9.51	9.72	9.71	-0.65
A_09_P104705	AF172995	40201	gig	8.49	8.46	8.52	8.51	8.89	8.84	8.69	8.67	0.28
A_09_P104770	FBtr0081145	35173	Acn	11.32	11.53	11.44	11.55	11.03	11.01	11.11	11.26	-0.36
A_09_P104795	FBtr0079799	34250	Cks30A	7.47	8.00	7.79	8.03	7.39	6.97	6.74	7.00	-0.79
A_09_P104850	FBtr0330407	31127	a6	9.19	9.32	9.32	9.40	8.83	9.09	8.75	8.86	-0.42
A_09_P104955	FBtr0112888	42824	sba	7.94	7.85	7.96	7.89	8.73	8.60	8.54	8.41	0.66
A_09_P104980	FBtr0074202	32515	CG11679	12.25	12.13	12.20	12.25	11.46	11.50	11.73	11.98	-0.54
A_09_P105075	FBtr0088959	35686	Orc1	8.16	8.45	8.30	8.31	6.91	6.96	7.55	7.75	-1.01
A_09_P105210	FBtr0084639	42919	nAcRalpha-96Ab	7.02	6.71	7.00	6.49	7.15	7.40	7.48	7.53	0.58
A_09_P105245	FBtr0339154	42561	GABA-B-R2	9.69	9.47	9.49	9.67	9.80	10.05	9.98	10.18	0.42
A_09_P105315	FBtr0083503	42109	Prx3	13.96	13.81	13.82	13.88	12.29	12.21	12.66	12.97	-1.34
A_09_P105375	FBtr0112789	41286	Takr86C	4.43	4.40	4.57	3.71	5.68	6.25	6.06	5.91	1.70
A_09_P105385	FBtr0082101	41169	Fst	14.82	14.75	14.84	14.76	15.47	15.35	15.25	14.96	0.47
A_09_P105460	FBtr0078492	40457	Ddx1	11.40	11.56	11.38	11.57	10.78	10.39	10.78	11.00	-0.74
A_09_P105590	FBtr0073375	38590	Con	7.19	7.14	6.90	6.77	7.99	8.15	7.63	7.54	0.83
A_09_P105615	FBtr0072101	37744	l(2)efl	15.76	16.52	16.40	16.43	14.34	14.43	14.67	14.89	-1.69
A_09_P105750	FBtr0082866	41703	Orc2	8.50	8.55	8.58	8.73	7.81	7.60	7.93	8.09	-0.73
A_09_P105755	FBtr0333867	39102	Uch-L3	12.24	12.52	12.49	12.57	11.79	11.72	12.00	12.23	-0.52
A_09_P105765	FBtr0302557	38836	Tsp66A	7.49	7.59	7.35	7.60	6.62	6.77	6.70	7.18	-0.69
A_09_P105865	FBtr0077198	33114	40421	9.16	9.74	9.40	9.61	8.64	8.73	8.89	9.11	-0.64
A_09_P106065	FBtr0076497	39071	Hsp67Bc	10.84	11.87	11.32	11.66	12.86	12.80	12.61	12.24	1.21
A_09_P106075	FBtr0077221	33118	Hlc	7.62	7.84	7.91	7.51	8.33	8.87	8.26	8.41	0.75
A_09_P106310	FBtr0071188	31746	Traf6	10.12	10.13	10.05	10.28	9.52	9.66	9.93	9.77	-0.42
A_09_P106615	FBtr0089685	40675	CRMP	11.99	12.06	11.82	11.99	10.47	10.52	10.82	11.26	-1.20
A_09_P106680	FBtr0334593	41913	alpha-Man-IIb	9.47	9.31	9.14	9.19	9.83	10.04	9.79	9.93	0.62
A_09_P106905	FBtr0075605	39671	CG7857	10.46	10.43	10.56	10.34	9.91	9.76	10.03	10.20	-0.47
A_09_P106920	AJ242796	39173	Nc	9.32	9.23	9.18	9.14	11.06	10.85	10.54	10.30	1.47
A_09_P106930	FBtr0110936	43982	Oamb	5.41	4.98	4.57	4.54	6.24	6.60	6.40	6.09	1.46
A_09_P107000	FBtr0301765	2768677	p53	8.85	8.48	8.31	8.46	9.02	9.17	8.88	8.96	0.48
A_09_P107185	FBtr0089075	43799	Ephrin	8.72	9.14	9.03	9.07	8.41	8.30	8.33	8.39	-0.63
A_09_P107190	FBtr0079027	33738	Rpn11	13.19	13.31	13.21	13.21	12.35	12.50	12.87	13.04	-0.54

A_09_P107240	FBtr0088184	36195	TpnC47D	16.43	16.46	16.45	16.46	16.15	16.04	16.23	16.19	-0.29
A_09_P107450	FBtr0070351	31156	eIF2B-epsilon	10.66	10.60	10.69	10.63	10.45	10.40	10.42	10.46	-0.21
A_09_P107455	FBtr0070479	31256	eIF2B-beta	9.06	8.87	8.85	8.77	8.18	8.28	8.53	8.67	-0.48
A_09_P107525	FBtr0076053	39368	rols	8.68	8.83	9.09	8.70	9.42	9.92	9.34	9.33	0.68
A_09_P107545	FBtr0075958	44118	vih	10.84	10.77	10.66	10.84	10.33	10.07	10.36	10.55	-0.45
A_09_P107630	FBtr0084748	42984	CG11771	12.93	12.76	12.85	12.62	12.59	12.47	12.46	12.44	-0.30
A_09_P107765	FBtr0305992	39377	yps	12.07	11.88	11.95	11.95	11.79	11.41	11.64	11.66	-0.34
A_09_P107810	FBtr0112830	43906	skd	8.44	8.95	8.59	8.81	9.26	9.51	9.28	9.15	0.61
A_09_P107910	FBtr0088834	35733	CG1882	11.66	11.49	11.63	11.55	13.23	13.38	13.01	12.78	1.51
A_09_P108115	FBtr0113478	32696	Arpc3B	11.80	11.90	11.82	11.83	12.34	12.35	12.21	12.16	0.43
A_09_P108165	FBtr0072132	37791	DNA-ligI	10.02	10.00	9.94	10.01	9.24	9.16	9.52	9.66	-0.60
A_09_P108305	FBtr0081841	40996	DppIII	14.78	14.79	14.81	14.77	13.55	13.38	13.98	14.36	-0.97
A_09_P108475	FBtr0079869	34300	IP3K1	10.27	9.83	10.08	9.95	11.07	11.11	10.76	10.61	0.86
A_09_P108505	FBtr0300615	34939	beat-Ib	7.32	6.83	6.83	6.21	7.85	8.16	7.93	7.98	1.18
A_09_P108545	FBtr0088567	35924	tsu	11.60	11.49	11.54	11.58	11.08	10.94	11.26	11.42	-0.37
A_09_P108731	FBtr0076439	39064	PGRP-LF	8.16	7.65	7.84	8.03	10.61	10.87	10.03	9.41	2.31
A_09_P108950	FBtr0331368	326116	tau	10.02	10.41	10.43	10.42	9.48	9.23	9.45	9.44	-0.92
A_09_P109000	FBtr0081758	45894	lds	8.25	8.55	8.56	8.56	8.21	7.96	8.06	7.99	-0.43
A_09_P109040	FBtr0299516	42632	lqfR	6.79	6.86	6.77	7.01	6.29	6.48	6.14	6.02	-0.62
A_09_P109080	FBtr0082656	41570	CG12279	11.04	11.15	11.25	11.19	10.09	9.94	10.30	10.33	-0.99
A_09_P109110	FBtr0331979	36415	CG17574	9.63	9.72	9.44	9.70	10.72	10.36	10.20	9.95	0.69
A_09_P109230	FBtr0083049	41832	CG4210	10.61	10.46	10.52	10.55	11.03	10.87	11.04	11.08	0.47
A_09_P109315	FBtr0339164	42709	CG17244	12.54	13.24	12.86	13.15	11.64	11.87	11.95	12.14	-1.05
A_09_P109340	FBtr0073642	32166	Chrac-16	7.76	8.34	7.92	8.26	7.35	7.60	7.41	7.29	-0.66
A_09_P109360	FBtr0301287	35211	fon	7.99	8.65	8.00	8.28	6.68	6.16	6.64	6.68	-1.69
A_09_P109455	FBtr0332706	39243	CG6272	9.90	10.04	10.12	10.17	10.64	10.67	10.61	10.66	0.58
A_09_P109470	FBtr0087739	50227	CG17059	11.66	11.83	11.79	11.95	11.34	11.48	11.52	11.68	-0.30
A_09_P109610	FBtr0340328	50209	IM3	6.79	5.62	4.65	4.27	9.75	10.39	9.28	8.39	4.12
A_09_P110060	FBtr0082311	41288	MED7	11.55	11.59	11.50	11.73	11.19	11.23	11.27	11.43	-0.31
A_09_P110140	FBtr0086625	251984	Jheh1	12.77	13.10	12.87	12.97	12.59	12.35	12.45	12.45	-0.47
A_09_P110320	FBtr0076995	38709	Lcp65Aa	10.24	10.71	10.64	10.68	9.73	9.86	9.36	8.75	-1.14

A_09_P110365	FBtr0299711	3346206	CG42313	6.77	6.26	6.19	5.82	7.64	7.84	7.54	7.17	1.29
A_09_P110425	FBtr0299851	39696	comm3	10.21	10.50	10.40	10.34	11.67	11.68	11.22	10.75	0.97
A_09_P110630	FBtr0342602	34630	Pex19	10.68	10.77	10.64	10.60	10.02	10.09	10.09	9.87	-0.65
A_09_P110685	FBtr0087448	36654	Ercc1	7.43	7.48	7.61	7.54	7.04	7.23	7.18	7.20	-0.35
A_09_P110773	FBtr0080590	44117	bgm	13.91	13.65	13.80	13.71	14.32	14.06	14.14	14.15	0.40
A_09_P110805	FBtr0086728	37102	CG15067	8.34	4.37	6.13	4.43	13.73	13.51	12.90	11.72	7.15
A_09_P110855	FBtr0336486	42030	CG14906	10.51	10.44	10.48	10.46	12.23	12.72	12.21	12.06	1.83
A_09_P110935	FBtr0073379	32013	CG17333	12.15	12.10	12.12	12.24	11.36	11.46	11.51	11.77	-0.63
A_09_P110975	FBtr0078210	40282	CG4858	11.68	11.60	11.64	11.73	11.81	11.88	11.93	11.93	0.23
A_09_P111005	FBtr0088163	36217	etaTry	12.20	11.97	12.00	12.04	12.74	12.73	12.46	12.23	0.48
A_09_P111030	FBtr0077218	33123	CG10918	11.88	13.35	12.89	13.77	13.95	14.66	14.20	14.21	1.28
A_09_P111075	FBtr0305317	246553	CG30345	9.88	9.38	9.37	9.29	10.64	10.79	10.90	10.99	1.35
A_09_P111115	FBtr0071286	318161	CG32708	11.85	11.67	11.81	11.73	11.23	11.12	11.31	11.48	-0.48
A_09_P111140	FBtr0084860	318664	CG31288	12.14	11.37	11.68	11.42	12.94	12.91	12.80	12.61	1.16
A_09_P111170	FBtr0330650	33195	CG17078	10.08	10.32	10.18	10.23	9.50	9.30	9.54	9.82	-0.66
A_09_P111230	FBtr0321259	34436	CG5322	11.49	10.45	10.58	10.42	12.23	12.01	11.96	11.80	1.26
A_09_P111240	FBtr0302854	34638	Phae2	11.34	12.08	11.87	12.04	13.86	12.93	13.32	12.99	1.44
A_09_P111265	FBtr0081048	35087	CG15155	11.40	12.74	12.26	12.97	7.57	8.21	9.30	10.09	-3.55
A_09_P111270	FBtr0081068	35105	CG10178	10.14	11.24	10.54	11.09	7.64	8.35	8.86	9.44	-2.18
A_09_P111325	FBtr0088031	36259	CG13192	11.11	11.13	11.19	11.03	10.90	10.61	10.70	10.65	-0.40
A_09_P111470	FBtr0074913	40181	CG14182	9.19	9.45	9.33	9.39	7.86	8.17	8.77	8.87	-0.92
A_09_P111490	FBtr0304704	40938	CG10445	6.00	6.25	6.25	5.93	6.87	7.32	6.94	7.25	0.99
A_09_P111505	FBtr0082112	41155	CG16790	8.89	8.80	9.05	8.89	9.22	9.28	9.10	9.14	0.28
A_09_P111550	FBtr0300570	41917	Arpc3A	11.76	11.77	11.74	11.75	11.99	11.91	11.92	11.96	0.19
A_09_P111575	FBtr0083783	42344	CG31219	3.74	NA	2.96	NA	6.02	6.37	5.92	6.04	2.74
A_09_P111585	FBtr0084107	42519	CG3337	11.65	11.57	11.66	11.60	11.17	11.02	11.09	11.13	-0.52
A_09_P111610	FBtr0335233	43062	CG31087	13.28	13.09	13.16	13.22	12.87	12.47	12.47	12.33	-0.65
A_09_P111625	FBtr0085104	43261	CG14259	6.19	7.05	6.64	7.08	3.97	5.26	4.58	4.12	-2.26
A_09_P111685	FBtr0074325	318094	TwdlX	14.20	14.42	14.60	14.30	13.44	13.87	13.74	14.06	-0.60
A_09_P111805	FBtr0075348	39870	PGRP-SB1	8.35	11.10	8.80	9.45	13.75	12.35	13.52	13.48	3.85
A_09_P111880	FBtr0335452	43646	CG11313	8.61	9.51	9.18	9.64	6.71	6.11	6.93	7.34	-2.46

A_09_P112015	FBtr0077180	38600	CG10673	10.80	10.86	10.86	10.91	11.13	11.10	10.99	10.99	0.19
A_09_P112060	FBtr0301117	42368	CG4733	11.02	11.20	11.25	11.11	10.71	10.69	10.52	10.69	-0.49
A_09_P112070	FBtr0085216	43326	CG12883	10.25	10.60	10.48	10.56	9.67	9.72	9.34	9.24	-0.98
A_09_P112155	FBtr0302362	3346221	lectin-37Db	7.36	7.05	6.90	6.95	8.86	8.57	8.90	9.01	1.77
A_09_P112280	FBtr0070863	31528	CG6048	11.41	11.44	11.20	11.31	13.09	12.65	12.45	12.08	1.23
A_09_P112305	FBtr0071178	31765	CG15343	8.69	9.29	8.92	9.26	7.82	8.26	7.94	7.95	-1.05
A_09_P112360	FBtr0077451	318825	mRpL27	13.17	12.92	13.02	13.00	12.62	12.66	12.80	12.90	-0.28
A_09_P112610	FBtr0079830	34269	CG17855	9.84	9.78	9.61	9.56	11.16	10.78	10.63	10.15	0.98
A_09_P112670	FBtr0080606	34823	CG18095	6.89	7.00	6.78	6.41	5.99	5.29	5.74	5.99	-1.02
A_09_P112680	FBtr0080754	34919	CG7631	12.77	11.46	11.61	11.15	13.87	13.91	13.50	13.14	1.86
A_09_P112685	FBtr0080908	35009	CG17928	11.48	11.60	11.36	11.50	11.26	11.22	11.00	10.94	-0.38
A_09_P112715	FBtr0081256	35251	CG13078	3.75	4.53	3.69	4.99	8.62	7.77	7.59	7.11	3.53
A_09_P112855	FBtr0330053	37173	CG15082	6.71	6.57	6.72	6.21	6.96	7.82	7.34	7.40	0.83
A_09_P112880	FBtr0302932	37271	Obp56h	11.30	11.11	11.27	11.34	11.64	11.59	11.52	11.35	0.27
A_09_P112910	FBtr0342900	37459	CG15673	10.29	9.49	9.89	9.84	11.56	11.84	11.21	10.50	1.40
A_09_P112955	FBtr0072878	38290	CG13807	11.88	11.95	11.91	12.02	11.48	11.57	11.67	11.84	-0.30
A_09_P113005	FBtr0331579	5740442	CG34356	6.12	5.02	5.59	4.30	6.99	7.68	7.28	7.48	2.10
A_09_P113210	FBtr0081603	40866	CG14606	9.34	9.44	9.54	9.40	11.38	11.31	10.86	10.46	1.57
A_09_P113330	FBtr0083599	42195	CG14312	7.93	7.48	7.50	7.66	8.01	8.16	8.18	8.13	0.48
A_09_P113400	FBtr0084821	43050	CG11878	8.59	8.77	8.97	8.75	9.69	9.37	9.31	9.17	0.61
A_09_P113410	FBtr0290052	43058	CG31097	9.48	9.46	9.40	9.67	8.50	8.47	8.87	9.00	-0.79
A_09_P113425	FBtr0084939	43133	CG5948	7.99	7.99	7.81	7.86	7.16	7.12	7.41	7.55	-0.60
A_09_P113430	FBtr0290139	43136	CG42235	9.51	10.98	10.23	10.76	9.03	8.87	8.89	8.73	-1.49
A_09_P113510	FBtr0082574	48340	GstD7	13.83	13.79	13.86	13.78	12.81	13.47	13.27	13.41	-0.57
A_09_P113545	FBtr0300214	50363	ksh	11.19	11.15	11.14	11.37	10.37	10.71	10.63	10.79	-0.59
A_09_P113550	FBtr0070390	50373	CG17776	12.78	12.69	12.89	12.97	12.20	12.61	12.40	12.42	-0.42
A_09_P113560	FBtr0082378	53502	Ugt86Di	5.61	4.92	4.09	4.05	9.07	8.88	9.24	9.61	4.53
A_09_P113575	FBtr0114516	59140	CG18744	9.55	8.65	8.80	8.57	11.17	11.03	10.57	10.10	1.83
A_09_P113650	FBtr0074494	32774	CG12985	4.83	3.57	4.48	4.39	5.91	5.94	5.73	5.57	1.47
A_09_P113755	FBtr0310521	35599	Spn42Db	7.39	8.21	7.96	8.54	5.31	5.81	6.37	6.95	-1.92
A_09_P113840	FBtr0299805	37122	CG5189	5.57	5.81	5.59	5.44	7.15	7.54	6.79	6.60	1.42

A_09_P113875	FBtr0072916	38274	CG15879	10.14	10.12	10.30	10.31	9.46	9.58	9.65	9.65	-0.63
A_09_P113950	FBtr0290228	39993	CG16775	10.94	8.98	10.45	9.73	13.47	12.47	13.01	12.70	2.89
A_09_P113990	FBtr0076440	317835	CG32040	5.91	4.98	4.79	4.96	6.95	7.59	7.02	6.80	1.94
A_09_P114000	FBtr0082335	41314	CG14695	8.08	8.32	8.60	8.47	11.05	10.97	10.55	10.13	2.31
A_09_P114195	FBtr0081399	35330	Cen	10.19	10.19	10.33	10.23	9.88	9.86	9.98	10.02	-0.30
A_09_P114240	FBtr0100394	40170	Mi-2	8.13	8.30	8.16	8.10	7.53	7.48	7.79	8.02	-0.47
A_09_P114270	FBtr0083797	42352	CG11659	12.97	13.25	13.52	13.78	10.97	10.97	11.71	12.17	-1.92
A_09_P114475	FBtr0308892	3354890	CG40498	7.63	7.54	7.55	7.53	7.87	8.01	8.12	7.84	0.40
A_09_P114595	FBtr0088432	36047	Def	5.55	7.28	5.80	5.20	10.17	10.37	10.34	10.34	4.35
A_09_P114685	FBtr0072487	38034	CG16940	11.03	10.97	11.10	10.96	10.69	10.66	10.86	10.83	-0.25
A_09_P114780	FBtr0304846	41549	Octbeta2R	6.21	6.65	6.38	5.79	3.48	3.59	4.37	4.37	-2.31
A_09_P114810	FBtr0085328	43408	CG14529	10.44	10.14	10.27	10.11	11.29	11.13	10.93	10.55	0.74
A_09_P114850	FBtr0299517	5740876	CG34452	8.37	7.72	8.30	8.00	5.64	5.86	6.19	6.53	-2.05
A_09_P114860	FBtr0300097	39315	pallidin	9.36	9.43	9.32	9.47	9.91	9.85	9.55	9.63	0.34
A_09_P114905	FBtr0082006	50007	CG11985	10.54	10.39	10.39	10.55	9.57	9.80	9.91	10.15	-0.61
A_09_P114925	FBtr0089123	318246	CG32850	6.51	6.59	5.56	6.06	7.24	7.77	7.16	7.02	1.12
A_09_P114960	FBtr0080543	34794	Orc5	7.43	7.64	7.19	7.44	6.99	6.98	6.73	7.05	-0.49
A_09_P115025	FBtr0079351	33978	CG10399	11.20	11.42	11.36	11.50	10.42	10.38	10.62	10.77	-0.82
A_09_P115120	FBtr0079640	34111	CG8498	11.62	11.54	11.67	11.70	10.85	11.00	11.30	11.38	-0.50
A_09_P115185	FBtr0081192	35151	Nedd8	12.53	12.64	12.65	12.79	11.85	11.86	12.06	12.32	-0.63
A_09_P115420	FBtr0088728	35848	beta3GalTII	8.94	8.95	8.88	8.99	8.56	8.71	8.57	8.82	-0.27
A_09_P115450	FBtr0331940	36215	kappaTry	12.65	12.03	12.01	11.91	13.14	13.33	12.97	12.67	0.88
A_09_P115475	FBtr0087581	50224	CG13018	10.63	10.32	10.62	10.50	9.65	9.91	9.95	9.95	-0.66
A_09_P115610	FBtr0070677	31385	CG15912	9.38	9.32	9.39	9.25	8.62	8.62	8.78	8.88	-0.61
A_09_P115785	FBtr0302434	38325	CG16986	7.68	7.46	7.32	7.18	7.79	7.79	7.96	8.04	0.48
A_09_P115815	FBtr0076748	38880	CG8281	9.70	9.86	9.60	9.95	10.44	10.98	10.37	10.28	0.74
A_09_P115860	FBtr0077064	38696	CG10469	8.70	9.08	8.98	9.03	8.29	8.58	8.30	8.20	-0.60
A_09_P115900	FBtr0075490	39771	CG5157	8.44	8.05	7.70	7.92	10.17	10.11	9.68	9.26	1.78
A_09_P115920	FBtr0299563	50259	CG14104	8.66	8.87	8.72	8.92	8.33	8.27	8.35	8.44	-0.45
A_09_P116090	FBtr0084815	43044	CG11858	12.28	12.18	12.44	12.30	11.65	11.77	11.79	11.84	-0.54
A_09_P116150	FBtr0083996	318725	Takl1	8.73	9.37	8.70	8.96	11.10	10.95	10.66	10.19	1.79

A_09_P116190	FBtr0085510	318624	CG31202	5.48	6.53	5.80	6.07	4.55	4.28	3.24	3.45	-2.09
A_09_P116205	FBtr0337033	43726	CG11539	9.11	9.06	8.98	8.91	8.56	8.36	8.64	8.89	-0.40
A_09_P116270	FBtr0086061	35505	scaf	13.48	13.00	13.41	13.30	14.16	14.23	14.10	14.02	0.83
A_09_P116365	FBtr0307497	35424	CG31619	8.57	8.38	8.52	8.34	8.81	9.02	8.98	9.07	0.52
A_09_P116500	FBtr0301100	31344	CG42541	5.83	4.86	4.61	4.58	6.06	6.41	6.28	6.49	1.34
A_09_P116515	FBtr0084273	42672	loco	3.75	3.56	3.97	2.52	5.40	5.64	5.08	5.13	1.86
A_09_P116535	FBtr0073220	38504	tipE	7.02	6.65	6.89	6.54	7.33	7.50	7.72	7.43	0.72
A_09_P116585	FBtr0085826	48317	ttk	8.84	9.06	8.93	8.96	9.15	9.38	9.31	9.30	0.34
A_09_P116615	FBtr0335418	42350	GluClalpha	6.93	7.05	6.20	5.98	7.83	8.42	7.69	8.33	1.53
A_09_P116640	FBtr0072733	38135	Klp61F	6.91	7.53	7.09	7.10	6.26	6.32	6.12	5.82	-1.03
A_09_P116660	FBtr0301390	43315	dsd	10.18	10.19	10.17	10.18	10.92	10.99	10.85	10.73	0.69
A_09_P116790	FBtr0071228	31801	Caf1-180	10.19	10.49	10.43	10.53	9.88	9.85	10.08	10.18	-0.41
A_09_P116830	FBtr0304833	40515	nrm	11.53	11.65	11.57	11.74	10.93	10.66	10.64	10.70	-0.89
A_09_P116840	FBtr0070914	31589	dx	9.13	9.43	9.23	9.49	8.34	8.33	8.64	8.57	-0.85
A_09_P116880	FBtr0084674	42930	CG13624	8.84	8.81	8.52	8.65	10.17	10.49	9.80	9.57	1.30
A_09_P116890	FBtr0089963	41852	Tm1	11.62	11.32	11.68	11.48	11.27	10.94	11.16	10.68	-0.51
A_09_P116945	FBtr0075646	39654	Msh6	7.31	7.38	7.57	7.60	6.77	6.29	6.43	6.44	-0.98
A_09_P116965	FBtr0091512	40858	gfzf	11.99	11.84	11.95	11.85	11.53	11.47	11.49	11.54	-0.40
A_09_P116990	FBtr0100430	46068	Acsl	11.47	11.08	11.26	11.02	10.79	10.85	10.77	10.70	-0.43
A_09_P116995	FBtr0085876	43765	Map205	10.02	10.62	10.48	10.33	9.44	9.48	9.17	9.04	-1.08
A_09_P117021	FBtr0301416	33277	IA-2	8.14	8.13	7.96	7.44	9.46	9.25	8.99	9.19	1.31
A_09_P117150	FBtr0078358	40385	Cdk12	9.28	9.42	9.38	9.36	8.80	8.58	8.79	8.89	-0.60
A_09_P117226	FBtr0077350	33132	CG14619	10.26	10.60	10.61	10.38	9.81	9.82	9.79	9.86	-0.64
A_09_P117240	FBtr0076268	39217	tna	9.98	10.41	10.01	10.28	10.71	11.04	10.66	10.50	0.56
A_09_P117250	FBtr0302710	39111	CG42673	8.04	7.80	8.05	7.65	8.94	9.05	8.59	8.34	0.84
A_09_P117310	FBtr0334699	53437	Sulf1	9.00	8.68	8.79	8.48	9.88	9.70	9.47	9.17	0.82
A_09_P117410	FBtr0303107	38206	CG7971	10.53	11.03	10.76	11.01	11.10	11.39	11.28	11.23	0.42
A_09_P117470	FBtr0075208	39957	Edc3	9.16	9.48	8.98	9.44	9.87	10.02	9.80	9.60	0.56
A_09_P117490	FBtr0082535	44672	Lk6	14.01	14.38	14.16	14.36	14.95	14.93	14.68	14.53	0.54
A_09_P117535	FBtr0080231	34541	Ge-1	9.41	9.61	9.49	9.34	8.97	9.15	9.15	9.21	-0.34
A_09_P117635	FBtr0081788	44910	unc-45	12.71	12.69	12.74	12.57	11.89	12.01	12.09	12.30	-0.61

A_09_P117665	FBtr0340232	44851	raw	10.96	10.59	10.58	10.47	12.40	12.67	11.94	11.28	1.42
A_09_P117685	FBtr0074920	40171	Su(Tpl)	12.98	13.09	12.98	13.21	14.28	14.16	13.98	13.80	0.99
A_09_P117795	FBtr0332107	38045	Tudor-SN	13.86	13.85	13.73	13.85	13.04	12.91	13.23	13.38	-0.68
A_09_P117875	FBtr0331514	39018	orb2	6.28	6.19	5.98	6.10	6.57	7.09	6.67	6.79	0.64
A_09_P117990	FBtr0076777	45588	Pdp1	7.92	7.65	7.55	7.19	9.77	9.73	9.04	8.76	1.74
A_09_P118015	FBtr0334319	45826	bel	10.14	10.07	10.28	10.12	9.67	9.37	9.21	9.11	-0.81
A_09_P118040	FBtr0305152	44669	cwo	10.28	10.26	10.32	10.39	11.06	11.13	10.89	10.49	0.58
A_09_P118095	FBtr0309319	40739	Rm62	13.75	13.91	13.67	13.93	14.61	14.52	14.35	14.06	0.57
A_09_P118135	FBtr0074893	40174	Rpn1	13.23	13.49	13.28	13.26	12.33	12.23	12.80	13.06	-0.71
A_09_P118215	FBtr0333754	31179	Unc-76	8.53	8.60	8.41	8.45	9.05	9.10	8.91	8.69	0.44
A_09_P118405	FBtr0305342	31538	CG5921	9.45	9.30	9.10	9.04	11.33	10.91	10.64	10.36	1.59
A_09_P118420	FBtr0340532	37232	CalpA	11.71	11.69	11.75	11.72	12.26	12.37	12.12	11.84	0.43
A_09_P118455	FBtr0100469	33260	CG3662	13.70	14.16	13.91	14.13	14.92	14.80	14.60	14.51	0.73
A_09_P118505	FBtr0088401	36048	magu	9.69	10.53	10.19	10.51	8.97	8.59	9.06	9.26	-1.26
A_09_P118575	FBtr0078256	40302	CG3680	10.45	10.97	10.83	10.87	9.85	9.80	10.13	10.37	-0.74
A_09_P118615	FBtr0340670	31493	CG32758	6.88	6.73	6.63	6.74	7.41	7.37	7.37	7.05	0.55
A_09_P118660	FBtr0075344	39878	CG9674	7.81	7.80	7.54	7.75	8.46	8.30	8.47	7.94	0.57
A_09_P118680	FBtr0082107	41158	Kap-alpha3	8.56	8.29	8.32	8.24	7.97	8.10	8.04	8.10	-0.30
A_09_P118695	FBtr0073620	32122	FucT6	10.53	10.67	10.64	10.64	10.23	10.35	10.23	10.42	-0.31
A_09_P118720	FBtr0079889	34328	Dref	9.75	9.85	9.81	9.93	9.59	9.51	9.52	9.52	-0.30
A_09_P118725	FBtr0070952	31603	Mcm6	11.50	11.83	11.59	11.80	11.23	11.03	11.26	11.40	-0.45
A_09_P118760	FBtr0089945	35343	vari	10.40	10.73	10.36	10.64	11.39	11.58	11.12	11.06	0.76
A_09_P118810	FBtr0089195	43822	CG11155	8.98	9.70	9.42	9.60	8.80	8.52	8.55	8.33	-0.87
A_09_P118910	FBtr0084845	318737	Muc96D	13.69	14.05	13.98	14.42	12.98	13.28	13.02	12.91	-0.99
A_09_P118970	FBtr0332249	36342	CG33012	10.07	10.48	10.58	10.44	9.37	8.40	8.88	8.78	-1.53
A_09_P119000	FBtr0071911	37618	CycB	9.32	10.10	9.93	9.89	8.75	8.78	8.51	8.48	-1.18
A_09_P119025	FBtr0330216	32425	Flo-2	9.68	9.83	9.95	10.03	9.62	9.56	9.51	9.46	-0.33
A_09_P119070	FBtr0333598	39759	ClC-c	9.63	9.94	9.78	9.86	9.50	9.25	9.29	9.35	-0.46
A_09_P119075	FBtr0075746	39570	D	8.80	8.64	8.63	8.72	9.13	9.55	9.19	9.15	0.56
A_09_P119090	FBtr0086942	36981	eIF3-S9	14.75	14.83	14.72	14.66	14.92	14.90	14.96	14.99	0.21
A_09_P119095	FBtr0085441	45285	Dr	7.60	8.21	7.81	8.15	6.76	7.18	7.10	7.23	-0.87

A_09_P119105	FBtr0303909	45248	Nckx30C	7.67	7.09	7.39	6.80	7.84	8.27	8.13	7.97	**0.82**
A_09_P119205	FBtr0091470	3346141	CG33521	8.80	8.87	8.68	8.75	7.69	7.25	7.58	7.69	**-1.22**
A_09_P119220	FBtr0332271	33291	CG4577	8.52	8.30	8.52	8.27	9.16	9.21	8.99	8.93	**0.67**
A_09_P119290	FBtr0077173	38605	shep	13.16	13.45	13.28	13.32	13.96	14.28	13.79	13.80	**0.66**
A_09_P119340	FBtr0071600	37435	CG9485	13.12	13.74	13.57	13.64	11.55	12.05	12.47	12.92	**-1.27**
A_09_P119420	FBtr0309233	50003	CG43462	3.88	3.95	4.61	3.82	5.07	5.79	5.74	6.02	**1.59**
A_09_P119470	FBtr0073651	32199	Lsp1alpha	14.91	15.88	15.53	15.74	3.15	5.24	3.80	NA	**-11.45**
A_09_P119475	FBtr0330698	34038	Slob	7.55	6.89	7.17	6.68	8.25	7.91	7.94	7.52	**0.84**
A_09_P119495	FBtr0081006	35047	dl	10.91	10.94	10.62	10.85	12.46	12.72	12.00	11.51	**1.34**
A_09_P119650	FBtr0334901	40602	CG14657	8.55	8.75	8.76	8.78	8.14	8.27	8.41	8.40	**-0.41**
A_09_P119655	FBtr0084786	43005	OstStt3	13.23	13.21	13.00	13.21	12.14	12.09	12.80	12.92	**-0.67**
A_09_P119815	FBtr0081931	41058	CG9821	12.09	12.05	12.04	12.18	13.42	13.31	12.92	12.70	**1.00**
A_09_P119845	FBtr0072989	38364	CG1271	10.54	10.13	10.30	10.11	11.26	11.16	10.98	10.79	**0.78**
A_09_P119875	FBtr0301409	32009	Imp	11.84	11.75	11.80	11.96	12.49	12.85	12.54	12.52	**0.76**
A_09_P119905	FBtr0083281	41959	CG5903	15.10	15.08	15.13	15.13	15.22	15.31	15.21	15.19	**0.12**
A_09_P119995	FBtr0071562	37355	insc	7.01	6.93	6.91	6.48	8.34	8.66	7.93	7.66	**1.31**
A_09_P120065	FBtr0076678	318833	CG33057	9.51	9.61	9.59	9.75	10.15	10.45	10.13	10.15	**0.61**
A_09_P120150	FBtr0303933	33343	chinmo	11.52	11.39	11.49	11.53	12.08	12.33	12.23	12.07	**0.69**
A_09_P120160	FBtr0075202	39962	Eip74EF	10.72	10.85	11.14	10.79	9.15	9.29	9.60	9.52	**-1.49**
A_09_P120170	FBtr0303151	59145	CG18754	4.52	4.54	3.96	4.58	6.20	6.49	5.87	5.85	**1.70**
A_09_P120650	FBtr0299709	40620	cno	9.05	8.88	9.10	8.93	9.14	9.33	9.46	9.31	**0.32**
A_09_P120705	FBtr0073396	32035	CG1552	7.06	6.91	7.05	6.75	8.49	8.92	8.10	7.73	**1.37**
A_09_P120845	FBtr0088310	36107	Caf1-105	8.46	8.36	8.49	8.59	7.84	7.95	7.95	8.05	**-0.53**
A_09_P120937	FBtr0074820	40207	Ssk	9.13	8.77	8.90	8.53	10.25	9.65	9.65	9.50	**0.93**
A_09_P120980	FBtr0075898	39475	MICAL-like	11.57	11.72	11.65	11.68	11.92	11.87	11.82	11.85	**0.21**
A_09_P121180	FBtr0302307	42208	WRNexo	9.59	9.64	9.72	9.73	8.56	8.26	8.84	9.25	**-0.94**
A_09_P121300	FBtr0079082	33760	CG14024	7.70	8.14	8.13	7.63	8.75	9.14	8.36	8.73	**0.84**
A_09_P121465	FBtr0079423	33994	Pvf2	7.16	8.12	7.53	7.60	8.58	8.47	8.61	8.71	**0.99**
A_09_P121700	FBtr0301316	33247	Eaat2	7.59	7.48	7.24	7.53	7.80	7.96	8.21	7.88	**0.50**
A_09_P121790	FBtr0089322	39424	CG10638	5.81	5.45	5.61	5.75	6.93	7.30	7.42	7.60	**1.66**
A_09_P121910	FBtr0085860	43744	Smvt	13.07	13.02	13.12	13.18	12.14	11.59	12.02	11.90	**-1.18**

A_09_P122110	FBtr0329990	38381	CG14962	8.86	8.88	8.74	8.87	8.38	8.37	8.38	8.46	-0.44
A_09_P122365	FBtr0344300	42728	CG6726	8.34	8.91	8.71	9.08	7.86	7.85	8.11	8.29	-0.73
A_09_P122565	FBtr0081905	41035	CG8116	12.23	12.42	12.25	12.33	12.00	12.07	11.80	11.89	-0.37
A_09_P123065	FBtr0085733	43671	Aph-4	13.41	13.40	13.32	13.10	12.33	12.91	12.70	12.92	-0.59
A_09_P123245	FBtr0333948	38895	Ect4	13.59	13.74	13.87	13.76	14.79	14.59	14.46	14.04	0.73
A_09_P123325	FBtr0077032	38667	CG32407	11.53	11.90	11.47	11.38	10.54	10.27	10.29	10.39	-1.20
A_09_P123330	FBtr0303884	318996	CG31875	7.08	7.09	7.26	7.23	6.99	6.69	6.69	6.58	-0.43
A_09_P123470	FBtr0080994	35060	CG6412	12.79	12.66	12.68	12.72	12.48	12.45	12.47	12.53	-0.23
A_09_P123580	FBtr0083566	42186	14-3-3epsilon	14.78	14.72	14.68	14.84	14.24	14.21	14.42	14.25	-0.48
A_09_P123745	FBtr0076395	39133	UbcD4	12.14	12.06	12.12	12.11	11.97	11.58	11.79	11.92	-0.29
A_09_P123760	FBtr0087005	36927	GstS1	13.74	14.02	13.93	14.12	13.38	13.46	13.54	13.68	-0.44
A_09_P123825	FBtr0113289	43102	CG5028	10.62	10.08	10.52	10.28	9.21	9.07	9.65	9.55	-1.00
A_09_P123980	FBtr0082865	41701	Ipp	7.89	8.28	8.17	8.32	7.58	7.46	7.44	7.72	-0.61
A_09_P124020	FBtr0074895	40177	Grasp65	12.70	13.18	12.95	13.19	12.11	12.08	12.36	12.53	-0.73
A_09_P124180	FBtr0073257	50170	Teh3	7.31	6.91	6.93	6.51	7.28	7.85	7.62	7.74	0.71
A_09_P124235	FBtr0332830	39153	CG43897	13.60	14.00	13.81	14.09	12.92	12.72	12.96	12.99	-0.98
A_09_P124335	FBtr0076604	38943	CG7083	9.74	10.03	9.86	10.00	11.16	10.96	10.76	10.31	0.89
A_09_P124465	FBtr0089561	38988	Zasp66	14.63	15.04	14.75	14.93	13.80	13.42	13.81	14.11	-1.05
A_09_P124582	FBtr0089612	2768886	Sdic4	11.02	11.06	11.08	11.24	10.87	10.61	10.76	10.72	-0.36
A_09_P124635	FBtr0088811	46027	kermit	10.40	10.07	10.22	10.06	11.39	11.10	11.02	10.79	0.89
A_09_P124660	FBtr0084853	43064	CG10553	8.72	8.79	8.77	8.78	5.48	6.16	7.04	7.55	-2.21
A_09_P124670	FBtr0073436	32047	Rpt3	12.24	12.52	12.22	12.27	11.37	11.56	11.79	12.12	-0.60
A_09_P124680	FBtr0078082	33226	lwr	6.49	6.23	6.41	5.89	4.80	4.69	5.18	5.86	-1.12
A_09_P124785	FBtr0304846	41549	Octbeta2R	9.76	10.18	10.10	9.88	8.00	8.27	8.45	8.71	-1.62
A_09_P124805	FBtr0077671	33510	CG17224	8.54	8.89	8.62	8.62	7.05	6.71	7.63	7.68	-1.40
A_09_P124880	FBtr0070764	44317	CanB	8.15	7.99	8.19	7.99	8.54	8.64	8.52	8.51	0.47
A_09_P125070	FBtr0305331	44131	Mkrn1	8.93	9.00	8.86	8.97	8.57	8.53	8.55	8.51	-0.40
A_09_P125215	FBtr0079780	34225	alien	12.20	12.28	12.16	12.30	11.66	11.22	11.80	12.06	-0.55
A_09_P125350	FBtr0344157	41738	CG12207	11.07	11.05	11.01	10.99	12.24	12.12	11.71	11.37	0.83
A_09_P125395	FBtr0273398	38818	pst	11.35	11.11	11.07	10.87	13.11	12.29	12.45	12.10	1.39
A_09_P125455	FBtr0344343	41170	Dh44	7.41	7.18	7.63	7.20	7.89	7.98	8.09	7.95	0.62

A_09_P125580	FBtr0078721	40708	CG1236	12.45	13.06	12.58	12.95	11.63	11.64	12.03	12.39	**-0.84**
A_09_P125865	FBtr0076897	44498	Surf1	11.59	11.40	11.54	11.38	8.23	8.49	9.23	9.58	**-2.60**
A_09_P125945	FBtr0078131	33202	Hop	13.11	13.22	13.14	13.12	11.99	11.98	12.49	12.89	**-0.81**
A_09_P126200	FBtr0072252	37862	CG3257	7.70	7.93	7.53	7.67	8.26	8.62	8.19	8.46	**0.67**
A_09_P126335	FBtr0111300	3355011	CG17683	7.31	7.40	7.13	7.24	6.69	6.92	6.92	6.99	**-0.39**
A_09_P126435	FBtr0082230	41261	CG12818	10.17	10.01	10.24	10.17	9.65	9.55	9.70	9.69	**-0.50**
A_09_P126440	FBtr0083358	42020	CG10340	13.63	13.44	13.56	13.46	13.06	13.01	13.18	13.32	**-0.38**
A_09_P126550	FBtr0089224	43832	plexA	11.02	11.08	11.13	11.24	10.72	10.54	10.69	10.52	**-0.50**
A_09_P126715	FBtr0074821	40207	Ssk	6.99	6.15	5.81	5.80	7.82	7.23	7.25	7.37	**1.23**
A_09_P126775	FBtr0344131	43323	Mlc1	14.22	14.40	14.42	14.56	13.72	13.14	13.58	13.51	**-0.91**
A_09_P126830	FBtr0082082	41173	CG16817	14.18	14.15	14.00	14.17	13.75	13.73	13.87	13.94	**-0.30**
A_09_P126835	FBtr0080785	34913	CG15255	13.00	11.91	12.33	12.05	13.92	13.90	13.60	13.21	**1.33**
A_09_P126840	FBtr0343671	34799	CG8997	10.94	11.09	10.41	10.81	10.00	9.86	9.88	10.01	**-0.87**
A_09_P126860	BT126208	38684	Jon65Aii	14.63	14.80	14.24	14.80	16.08	15.83	15.90	15.72	**1.26**
A_09_P126880	FBtr0070867	31536	CG5941	10.59	10.96	10.77	10.90	10.42	10.56	10.35	10.48	**-0.35**
A_09_P126900	FBtr0072676	38154	mtacp1	13.79	13.81	13.83	13.69	13.00	13.04	13.20	13.44	**-0.61**
A_09_P126940	FBtr0070797	31457	NAAT1	10.77	10.43	10.58	10.67	12.32	12.21	11.83	11.35	**1.32**
A_09_P127535	FBtr0081831	40978	CG31463	11.67	10.54	10.66	10.34	13.44	13.15	12.82	12.31	**2.13**
A_09_P127595	FBtr0082537	41461	Spt3	8.72	8.59	8.86	8.68	8.38	8.46	8.44	8.37	**-0.30**
A_09_P127640	FBtr0083780	42338	CG16727	10.60	11.89	11.29	11.92	7.86	8.42	9.16	9.52	**-2.69**
A_09_P127720	FBtr0085173	43293	bigmax	9.73	9.94	9.57	9.97	11.18	11.28	10.62	10.26	**1.03**
A_09_P127765	FBtr0081777	59141	CG18745	10.78	10.93	10.70	10.75	12.70	12.54	12.07	11.49	**1.41**
A_09_P127935	FBtr0344217	43362	CG34362	9.13	8.87	8.85	9.01	9.87	10.15	9.86	9.76	**0.95**
A_09_P128070	FBtr0074631	32865	CG6891	8.21	8.72	8.39	8.42	7.68	7.46	7.60	7.48	**-0.88**
A_09_P128265	FBtr0340145	30996	CG17778	4.46	3.68	4.40	3.52	5.41	6.03	5.77	5.55	**1.67**
A_09_P128290	FBtr0081331	250736	bwa	8.61	9.08	8.52	8.72	9.84	9.61	9.41	9.26	**0.80**
A_09_P128345	FBtr0332060	38693	CG6610	10.75	10.70	10.61	10.80	10.14	10.34	10.32	10.53	**-0.38**
A_09_P128460	FBtr0082773	41638	CG8795	8.56	8.00	8.41	8.00	9.74	10.06	9.40	9.11	**1.34**
A_09_P128475	FBtr0088477	36012	CG1663	7.74	7.57	7.52	7.64	7.10	6.94	7.01	7.30	**-0.53**
A_09_P128490	FBtr0072155	37818	CG3065	9.83	10.27	10.06	10.23	9.49	9.51	9.63	9.78	**-0.49**
A_09_P128600	FBtr0073233	38526	CG1316	11.50	11.73	11.65	11.83	11.17	10.97	11.22	11.23	**-0.53**

A_09_P128650	FBtr0075657	39643	CG6945	9.01	9.19	9.28	9.40	8.63	8.31	8.52	8.42	-0.75
A_09_P128775	FBtr0343338	37853	Mlp60A	6.08	6.00	6.06	5.78	6.74	6.60	6.53	6.62	0.64
A_09_P128810	FBtr0289975	40481	CG14456	10.73	10.53	10.72	10.74	11.18	11.51	11.32	11.19	0.62
A_09_P128920	FBtr0290221	39909	Ilp8	7.85	6.44	7.08	6.19	9.58	9.69	9.73	9.96	2.85
A_09_P128945	FBtr0113171	39626	CG10006	9.63	10.28	10.17	10.18	9.26	9.02	9.29	9.43	-0.81
A_09_P128985	FBtr0075918	39444	CG32109	8.22	8.27	8.01	8.03	8.44	8.45	8.69	8.80	0.47
A_09_P129060	FBtr0333887	38997	CG6511	9.43	9.58	9.44	9.67	7.42	7.49	8.83	9.31	-1.27
A_09_P129065	FBtr0076568	38994	Pex7	10.49	9.64	10.23	9.78	11.48	11.70	11.42	11.15	1.41
A_09_P129135	FBtr0077037	38677	CG32406	7.25	6.90	7.21	6.49	8.24	8.42	7.97	7.86	1.16
A_09_P129205	FBtr0342888	37429	Sgf29	9.49	9.48	9.35	9.50	9.68	9.57	9.66	9.75	0.21
A_09_P129240	FBtr0333445	38329	CG1143	13.73	13.92	13.95	14.02	14.22	14.44	14.31	14.37	0.43
A_09_P129245	FBtr0072968	38310	CG16762	10.40	10.14	10.39	10.13	13.24	12.25	12.59	12.18	2.30
A_09_P129310	FBtr0072625	38120	CG13912	11.46	11.43	11.47	11.67	10.30	10.58	10.84	11.35	-0.74
A_09_P129390	FBtr0087199	246451	CG30093	5.37	3.45	5.08	5.28	6.70	7.26	6.52	6.25	1.89
A_09_P129395	FBtr0087404	36682	CG8090	9.87	9.80	9.61	9.89	9.11	8.60	9.24	9.51	-0.68
A_09_P129400	FBtr0301735	36620	CG12868	11.17	11.33	11.38	11.39	13.66	13.44	12.96	12.45	1.81
A_09_P129405	FBtr0087517	36603	CG17385	11.46	11.54	11.60	11.46	11.24	11.30	11.18	10.96	-0.35
A_09_P129425	FBtr0087922	36353	Cpr49Ag	14.52	13.77	13.93	13.83	14.93	15.08	14.79	14.61	0.84
A_09_P129455	FBtr0088375	36022	Ntmt	9.29	9.30	9.43	9.33	9.24	9.19	9.19	9.09	-0.16
A_09_P129465	FBtr0085867	43731	CG11550	7.10	8.66	8.05	8.68	5.19	6.17	6.55	6.82	-1.94
A_09_P129473	FBtr0113315	43721	CG1638	8.05	7.70	7.86	7.83	7.13	7.05	7.36	7.65	-0.56
A_09_P129545	FBtr0085133	43264	CG6074	11.70	12.61	12.21	12.51	10.27	9.96	10.65	11.00	-1.79
A_09_P129565	FBtr0290328	43213	beat-VII	6.37	5.60	5.55	5.24	7.08	7.64	7.02	7.17	1.54
A_09_P129650	FBtr0083900	42410	CG4362	13.87	13.63	12.86	13.63	15.83	15.54	15.56	15.31	2.06
A_09_P129800	FBtr0329899	5740131	CG34383	9.74	10.38	9.62	10.22	10.34	10.93	10.73	10.97	0.75
A_09_P129855	FBtr0110808	4379854	CG34114	7.61	7.11	7.12	6.81	8.52	8.87	8.26	8.30	1.33
A_09_P129880	FBtr0082204	41229	CG12811	13.55	13.70	13.60	13.69	12.63	12.75	12.92	13.28	-0.74
A_09_P129885	FBtr0082150	41219	Art4	9.06	9.17	9.01	9.09	8.39	8.35	8.50	8.71	-0.60
A_09_P129895	FBtr0082167	41187	CG12948	10.56	10.67	10.77	10.88	10.26	10.30	10.20	10.13	-0.49
A_09_P130005	FBtr0301835	32928	gfA	7.13	7.14	6.69	6.51	7.63	8.45	7.71	8.10	1.10
A_09_P130075	FBtr0074334	32670	CG13005	7.48	7.42	7.22	7.07	6.01	6.55	6.68	6.91	-0.76

A_09_P130095	FBtr0074240	32592	CG9914	14.50	14.76	14.58	14.45	13.43	13.37	13.86	13.93	-0.93
A_09_P130110	FBtr0074153	32563	CG9170	9.11	9.55	9.34	9.45	9.72	10.20	9.80	9.80	0.52
A_09_P130375	FBtr0081262	35257	CG10262	8.14	8.46	8.47	8.40	8.10	7.82	7.99	8.06	-0.37
A_09_P130540	FBtr0079605	34146	CG14273	12.58	12.81	12.73	12.68	11.97	11.54	12.16	12.51	-0.66
A_09_P130765	FBtr0113182	40288	cmpy	7.74	7.52	7.47	7.13	8.09	8.14	8.23	8.34	0.74
A_09_P130780	FBtr0082651	41561	CG6188	13.83	13.26	13.60	13.68	14.83	14.73	14.50	14.42	1.03
A_09_P130840	FBtr0305495	31567	Rpt4	14.08	14.33	14.05	14.13	13.22	13.24	13.69	14.00	-0.61
A_09_P130855	FBtr0070211	31080	Tsp2A	10.58	10.29	10.28	10.32	11.50	11.46	11.24	10.87	0.90
A_09_P130890	FBtr0070346	31165	usp	9.86	10.22	10.26	10.16	9.49	9.60	9.74	9.90	-0.45
A_09_P130980	FBtr0070495	31268	crm	7.61	7.72	7.62	7.57	8.03	8.02	7.94	7.72	0.30
A_09_P130995	FBtr0070847	31487	CG3160	9.13	9.35	9.10	9.07	8.77	8.53	8.74	8.78	-0.46
A_09_P131140	FBtr0073570	32149	SelG	12.35	12.36	12.40	12.42	12.51	12.46	12.46	12.47	0.10
A_09_P131200	FBtr0074135	32567	exd	11.36	11.61	11.71	11.59	11.25	11.24	11.17	11.07	-0.38
A_09_P131250	FBtr0340632	32754	CG42684	11.32	11.81	11.50	11.71	12.34	12.53	12.18	12.22	0.73
A_09_P131385	FBtr0077835	33399	mio	10.32	10.56	10.45	10.57	11.27	11.31	11.00	10.75	0.61
A_09_P131445	FBtr0335136	33654	RpL27A	15.92	15.92	15.77	15.91	16.07	16.05	15.99	16.02	0.15
A_09_P131640	FBtr0079842	34282	GlcAT-S	9.71	9.75	9.65	9.61	10.37	10.37	10.04	10.00	0.52
A_09_P131760	FBtr0080930	35016	Cas	12.20	12.44	12.28	12.60	11.65	11.46	11.86	12.13	-0.61
A_09_P131900	FBtr0088782	35781	CG18316	9.75	9.67	9.70	9.82	10.38	10.35	10.26	10.13	0.54
A_09_P132155	FBtr0300127	36664	Cyp6a20	9.70	8.76	9.19	8.62	11.26	10.73	10.47	9.97	1.54
A_09_P132165	FBtr0087369	36692	scb	10.18	10.52	10.39	10.32	11.44	11.27	11.03	10.65	0.74
A_09_P132170	FBtr0087374	36697	mus210	9.48	9.72	9.67	9.73	10.70	10.70	10.50	10.37	0.92
A_09_P132285	FBtr0086566	37185	sano	8.97	9.06	8.94	8.64	10.72	10.93	10.32	10.21	1.64
A_09_P132325	FBtr0071671	37477	CG10307	6.80	7.41	7.14	7.46	5.91	6.08	6.34	6.68	-0.95
A_09_P132345	FBtr0071905	37638	Gmer	10.48	10.35	10.42	10.50	9.81	9.50	9.98	10.15	-0.58
A_09_P132440	FBtr0072308	37924	uri	9.79	9.83	9.76	9.79	9.41	9.45	9.59	9.70	-0.26
A_09_P132610	FBtr0331788	38715	CG7376	6.92	7.13	7.12	6.91	6.57	6.73	6.67	6.59	-0.38
A_09_P132695	FBtr0076519	39032	CG5068	9.40	9.33	9.45	9.39	9.10	9.04	9.16	9.04	-0.30
A_09_P132780	FBtr0075913	39443	Ptp69D	9.99	10.06	10.16	10.12	9.74	9.29	9.59	9.56	-0.54
A_09_P132835	FBtr0075495	39763	IntS9	10.26	10.41	10.22	10.36	9.65	9.45	9.90	10.07	-0.54
A_09_P132850	FBtr0075308	39845	Rpn12	12.76	12.86	12.71	12.80	11.73	11.76	12.32	12.64	-0.67

A_09_P133020	FBtr0334904	40598	Hus1-like	7.69	7.35	7.39	7.04	8.41	8.32	8.27	8.31	0.96
A_09_P133135	FBtr0081930	41059	CG9836	12.73	12.79	12.77	12.83	13.12	13.10	13.01	12.98	0.27
A_09_P133340	FBtr0083218	41945	GATAe	10.68	10.11	10.32	9.92	11.99	11.62	11.49	11.07	1.29
A_09_P133625	FBtr0085132	43265	gb	10.18	10.56	10.39	10.33	9.50	9.42	9.10	9.09	-1.09
A_09_P133915	FBtr0081687	40815	twr	15.10	15.22	15.21	15.14	13.89	13.97	14.52	14.92	-0.84
A_09_P133950	FBtr0306102	2768940	Pp2A-29B	13.81	13.94	13.88	13.96	13.74	13.76	13.83	13.69	-0.15
A_09_P133980	FBtr0332760	8674111	CG42445	8.95	9.02	9.05	9.23	8.12	8.30	8.60	8.71	-0.63
A_09_P134115	FBtr0076707	38934	Sbp2	9.39	9.84	9.39	9.77	10.15	10.35	10.10	10.11	0.58
A_09_P134205	FBtr0089500	37746	Pal2	9.58	9.68	9.51	9.50	10.24	10.07	10.07	9.94	0.51
A_09_P134230	FBtr0074210	32584	Pros28.1	12.37	12.76	12.55	12.66	11.55	11.61	11.89	12.24	-0.76
A_09_P134240	FBtr0072064	37733	Pi3K59F	10.22	10.58	10.40	10.38	11.18	11.14	10.96	10.95	0.66
A_09_P134290	FBtr0084744	42947	Ast	9.80	9.65	9.62	9.82	10.20	10.44	10.25	9.86	0.46
A_09_P134315	FBtr0112710	43892	sif	7.46	6.79	7.07	6.56	8.38	8.76	8.54	8.47	1.57
A_09_P134350	FBtr0333472	53580	Dip1	8.52	8.46	8.58	8.60	7.74	8.03	8.06	8.31	-0.50
A_09_P134565	FBtr0334401	33638	Traf4	9.24	9.47	9.28	9.35	10.62	10.92	10.49	10.29	1.24
A_09_P134800	FBtr0339509	43833	toy	7.91	8.31	8.07	8.09	10.24	10.38	9.93	9.48	1.91
A_09_P134910	FBtr0076938	38760	sgl	12.54	12.14	12.16	12.17	13.77	13.53	13.35	13.14	1.19
A_09_P134915	FBtr0075021	40093	nes	12.07	12.09	12.40	12.08	11.81	11.71	11.75	11.80	-0.39
A_09_P134980	FBtr0074921	40168	Rab8	10.35	10.39	10.16	10.44	10.72	10.60	10.71	10.63	0.33
A_09_P134995	FBtr0075141	39989	CG7441	11.23	10.86	11.15	11.03	10.53	10.25	10.41	10.42	-0.66
A_09_P135025	FBtr0080845	34969	cact	10.62	10.19	10.27	10.17	12.01	11.87	11.43	10.84	1.23
A_09_P135126	FBtr0084079	49228	mod(mdg4)	11.20	11.27	11.32	11.38	10.49	10.68	10.81	10.87	-0.58
A_09_P135190	FBtr0300649	35963	CG8801	8.68	8.65	8.63	8.35	9.60	9.48	9.27	9.11	0.79
A_09_P135205	FBtr0308570	32975	et	10.18	10.75	10.73	10.58	9.40	9.83	9.92	9.94	-0.78
A_09_P135325	FBtr0085330	43412	CG14527	12.07	11.86	11.90	11.82	10.07	10.12	11.15	11.57	-1.18
A_09_P135350	FBtr0085438	44643	Cnx99A	11.40	11.37	11.24	11.47	10.07	10.17	10.63	10.84	-0.94
A_09_P135485	FBtr0089931	35940	ltd	11.29	11.30	11.14	11.20	11.75	11.73	11.51	11.42	0.37
A_09_P135620	FBtr0077157	38611	Klp64D	11.19	11.27	11.27	11.36	10.92	11.04	11.02	10.97	-0.29
A_09_P135760	FBtr0072973	38346	pgant6	12.52	12.94	12.88	13.06	12.24	12.13	12.34	12.51	-0.54
A_09_P135840	FBtr0308750	34565	mre11	9.09	9.17	9.08	9.29	10.86	10.84	10.86	10.82	1.68
A_09_P135855	FBtr0076301	326186	scramb1	10.96	10.91	11.18	10.98	11.71	11.54	11.42	11.19	0.46

A_09_P136080	FBtr0070906	31579	kdn	13.02	13.11	13.29	13.19	12.83	12.98	12.71	12.36	**-0.43**
A_09_P136085	FBtr0085298	43385	Hrb98DE	13.55	13.51	13.33	13.55	12.81	12.73	12.91	13.28	**-0.55**
A_09_P136145	FBtr0079444	34021	CG5261	14.09	14.13	14.08	14.06	13.29	13.24	13.58	13.87	**-0.60**
A_09_P136212	FBtr0087853	36408	Dgkepsilon	9.17	9.34	9.25	9.34	8.84	8.75	8.90	9.12	**-0.37**
A_09_P136235	FBtr0075358	39858	Mo25	11.53	11.55	11.47	11.68	10.75	11.22	11.12	11.08	**-0.51**
A_09_P136340	FBtr0078629	40717	Rpn5	12.45	12.57	12.38	12.39	11.39	11.25	11.94	12.07	**-0.78**
A_09_P136370	FBtr0086145	35569	Hsepi	6.87	7.23	7.16	6.99	5.99	6.04	6.46	6.31	**-0.87**
A_09_P136535	FBtr0083730	42293	Cyp12a5	11.10	11.83	11.70	12.02	10.81	10.64	10.56	10.26	**-1.10**
A_09_P136610	FBtr0079038	33721	Rtnl1	14.71	14.84	14.77	14.73	14.15	13.93	14.31	14.35	**-0.58**
A_09_P136615	FBtr0087185	36808	fidipidine	10.69	10.69	10.74	10.91	10.42	10.29	10.57	10.59	**-0.29**
A_09_P136665	FBtr0084080	49228	mod(mdg4)	10.76	11.28	11.13	11.29	10.37	10.45	10.48	10.64	**-0.63**
A_09_P136745	FBtr0339278	35822	Cyp4e2	13.16	13.62	13.25	13.60	12.37	12.17	12.52	12.69	**-0.97**
A_09_P136770	FBtr0332328	33967	x16	9.84	10.19	10.06	10.09	9.49	9.49	9.68	9.83	**-0.42**
A_09_P136850	FBtr0087100	36850	CG4945	10.02	10.04	9.99	10.05	9.29	9.33	9.43	9.61	**-0.61**
A_09_P136891	FBtr0081987	41095	D1	12.07	12.32	12.06	12.49	11.63	11.34	11.77	11.67	**-0.63**
A_09_P136970	FBtr0087516	36606	phyl	7.03	6.81	6.55	6.96	7.28	8.01	7.69	7.45	**0.77**
A_09_P137085	FBtr0075492	44010	Pgm	15.02	15.26	15.33	15.38	14.76	14.56	14.89	14.91	**-0.47**
A_09_P137150	FBtr0071019	31657	CG4617	7.85	8.01	8.10	8.07	7.55	7.82	7.61	7.59	**-0.37**
A_09_P137260	FBtr0075058	40048	Cat	14.54	15.12	15.05	15.09	13.88	13.90	14.06	14.18	**-0.95**
A_09_P137310	FBtr0085559	43574	CG7943	11.89	11.79	11.88	11.72	11.62	11.65	11.46	11.52	**-0.26**
A_09_P137325	FBtr0073727	32269	CG12096	11.12	11.25	11.19	11.24	10.21	10.25	10.67	10.98	**-0.67**
A_09_P137400	FBtr0339498	33131	CG14614	10.04	10.19	10.00	10.13	9.76	9.83	9.96	9.95	**-0.22**
A_09_P137410	FBtr0082718	41603	CG12360	6.37	7.07	6.74	6.79	4.74	5.73	5.31	5.97	**-1.31**
A_09_P137450	FBtr0084190	42586	CG6439	11.96	12.09	12.14	12.02	10.78	10.33	10.75	10.85	**-1.37**
A_09_P137505	FBtr0079925	34291	Cyp4e3	6.08	6.84	6.27	6.99	10.40	10.54	9.56	8.40	**3.18**
A_09_P137570	FBtr0073299	38545	nAcRbeta-64B	8.94	8.48	8.72	8.47	9.27	9.43	9.35	9.48	**0.73**
A_09_P137765	FBtr0301773	39063	PGRP-LC	6.95	7.16	6.67	6.60	9.04	8.98	8.36	8.15	**1.79**
A_09_P138025	FBtr0343382	34089	CG7224	12.89	13.17	13.17	13.22	14.89	15.42	14.77	14.11	**1.69**
A_09_P138060	FBtr0343742	2768917	CG33301	11.41	11.52	11.60	11.73	10.94	11.15	10.81	10.77	**-0.65**
A_09_P138065	FBtr0079489	34066	r2d2	9.57	9.51	9.43	9.57	8.75	8.38	8.84	9.04	**-0.77**
A_09_P138290	FBtr0084988	318682	CG31323	8.01	7.15	6.99	6.86	8.59	8.72	8.33	7.86	**1.12**

A_09_P138405	FBtr0075171	39978	NUCB1	13.25	13.63	13.52	13.57	12.58	12.46	12.89	13.09	-0.74
A_09_P138430	FBtr0089194	43822	CG11155	8.51	9.36	9.17	9.31	8.21	8.06	7.91	7.76	-1.10
A_09_P138455	FBtr0302528	2768840	CG33461	7.69	7.83	7.74	8.13	7.05	7.41	7.15	7.40	-0.59
A_09_P138595	FBtr0075294	39903	CG6664	10.01	10.58	10.31	10.35	10.72	10.83	10.76	10.80	0.47
A_09_P138685	FBtr0082960	41771	CG14853	6.83	6.83	6.84	6.39	7.94	8.11	7.72	7.60	1.12
A_09_P138690	FBtr0085192	43319	Mtl	8.21	8.30	8.13	8.04	7.70	7.40	7.57	7.73	-0.57
A_09_P138895	FBtr0082052	41118	Fps85D	9.84	9.88	9.52	9.66	10.09	10.44	10.25	10.01	0.47
A_09_P138980	FBtr0077670	33512	alpha4GT1	9.29	9.66	9.41	9.22	8.78	8.89	8.82	8.90	-0.54
A_09_P138990	FBtr0335509	34341	CG44153	5.53	3.94	4.88	4.48	6.10	6.28	5.74	6.09	1.34
A_09_P139025	FBtr0081109	44183	ScpX	12.80	13.31	13.12	13.33	12.33	12.30	12.38	12.45	-0.78
A_09_P139055	FBtr0088805	35797	CG14757	8.10	8.25	8.38	8.63	8.10	7.37	7.36	7.07	-0.86
A_09_P139155	FBtr0076062	39364	viaf	13.45	13.46	13.71	13.41	13.12	12.94	12.94	12.98	-0.51
A_09_P139310	FBtr0088831	47186	mus205	8.71	8.98	8.71	8.93	9.95	10.18	9.83	9.89	1.13
A_09_P139420	FBtr0082030	41123	CG8135	11.24	11.39	11.53	11.48	10.72	11.04	10.96	11.14	-0.45
A_09_P139475	FBtr0084901	43102	CG5028	12.28	12.45	12.43	12.08	12.99	12.78	12.78	12.97	0.57
A_09_P139610	FBtr0075883	39483	SRm160	10.56	10.76	10.70	10.81	10.21	10.06	10.27	10.33	-0.49
A_09_P139620	FBtr0076720	317996	CG32364	4.64	5.31	4.76	4.93	7.21	7.59	7.32	6.99	2.37
A_09_P139850	FBtr0332813	40434	P5CDh1	9.34	9.70	9.58	9.68	8.25	8.03	8.43	8.51	-1.27
A_09_P139890	FBtr0342718	246485	CG30152	11.90	11.92	12.01	11.99	12.02	12.21	12.12	12.23	0.19
A_09_P140075	FBtr0078508	40468	laza	10.03	10.17	10.19	10.10	11.79	12.06	11.33	10.97	1.41
A_09_P140165	FBtr0303766	37514	CG11474	10.10	10.21	10.00	10.02	8.76	8.63	9.17	9.43	-1.09
A_09_P140170	FBtr0331936	36159	CG7712	12.25	12.35	12.38	12.45	11.87	11.97	11.98	12.15	-0.36
A_09_P140200	FBtr0300561	39454	Atg1	10.78	10.72	10.66	10.60	12.15	12.11	11.60	11.26	1.09
A_09_P140270	FBtr0070987	31610	CG14440	7.31	7.21	7.06	7.30	6.61	6.53	6.66	6.91	-0.54
A_09_P140415	FBtr0070311	31144	Vps26	11.72	11.97	11.95	11.87	11.60	11.53	11.66	11.46	-0.32
A_09_P140470	FBtr0083174	41910	Acyp2	9.49	9.62	9.57	9.67	9.00	8.94	9.08	9.09	-0.56
A_09_P140795	FBtr0301950	31456	SK	6.52	5.45	5.50	5.79	7.45	8.10	7.26	7.19	1.69
A_09_P141360	FBtr0085088	43236	Ets97D	8.60	8.75	8.64	8.62	8.11	8.20	8.33	8.42	-0.39
A_09_P141365	FBtr0085595	43569	janA	12.68	12.78	12.85	12.90	12.10	12.02	12.39	12.57	-0.53
A_09_P141395	FBtr0072233	37780	wibg	11.72	11.56	11.67	11.56	10.99	11.23	11.24	11.35	-0.42
A_09_P141495	FBtr0078917	40553	Cont	11.98	12.24	12.04	12.11	12.65	12.65	12.45	12.28	0.41

A_09_P141755	FBtr0070778	31463	SPR	7.23	6.80	6.70	6.14	8.24	8.04	7.72	7.56	1.17
A_09_P142065	FBtr0084532	42844	Ime4	9.89	10.13	9.98	10.20	9.38	9.32	9.45	9.69	-0.59
A_09_P142140	FBtr0110874	41839	tefu	9.26	9.72	9.47	9.83	8.80	9.00	8.85	9.04	-0.65
A_09_P142265	FBtr0089715	42757	pnt	7.45	6.93	6.90	6.95	7.74	8.04	7.67	7.65	0.72
A_09_P142282	FBtr0083632	42230	CG7694	12.21	12.48	12.31	12.60	11.82	11.87	11.89	12.14	-0.47
A_09_P142455	FBtr0305708	40346	CG9391	11.69	11.63	11.71	11.69	11.34	11.26	11.43	11.58	-0.28
A_09_P142710	FBtr0340293	32095	Spase25	13.65	14.18	13.91	14.13	12.54	12.51	13.18	13.68	-0.99
A_09_P143015	FBtr0072441	37950	ST6Gal	8.12	7.51	7.42	7.54	8.34	8.84	8.49	8.82	0.97
A_09_P143135	FBtr0334681	41200	RpS29	15.54	15.56	15.46	15.52	15.71	15.72	15.76	15.71	0.20
A_09_P143255	FBtr0078754	40671	CG14671	11.94	11.84	11.88	11.86	11.42	11.49	11.50	11.63	-0.37
A_09_P143355	FBtr0089077	43799	Ephrin	9.44	9.83	9.61	9.77	9.05	8.87	8.97	8.94	-0.70
A_09_P143485	FBtr0075780	39560	CG13484	9.58	9.47	9.64	9.61	8.62	8.71	8.83	8.96	-0.80
A_09_P143530	FBtr0077718	33471	CG2862	9.67	9.55	9.81	9.63	9.10	9.25	9.21	9.29	-0.46
A_09_P143571	FBtr0079636	34119	Su(var)205	12.35	12.57	12.70	12.65	12.17	11.72	12.08	12.11	-0.55
A_09_P143625	FBtr0073471	44072	ran	13.84	13.88	13.93	13.84	13.22	13.05	13.45	13.53	-0.56
A_09_P143635	FBtr0307341	43179	CG31086	16.09	15.65	15.95	15.60	16.20	16.24	16.15	16.15	0.36
A_09_P143640	FBtr0310559	44380	fok	9.44	9.17	9.04	9.21	10.16	10.22	9.96	9.57	0.76
A_09_P143645	FBtr0100369	36589	Ih	9.54	9.09	8.73	9.20	9.82	10.24	9.89	9.65	0.76
A_09_P143700	FBtr0302156	34896	Cul-3	9.97	10.28	10.01	10.11	9.62	9.64	9.76	9.81	-0.38
A_09_P143705	FBtr0303790	40793	gpp	5.99	5.97	5.75	5.72	6.94	7.09	6.56	6.44	0.90
A_09_P143820	FBtr0329905	33528	CG12400	8.99	8.97	9.00	8.92	8.17	8.25	8.08	8.05	-0.83
A_09_P143855	FBtr0089145	43774	CG31998	8.43	8.40	8.56	8.37	8.06	7.96	8.02	7.67	-0.51
A_09_P143935	FBtr0304998	38895	Ect4	6.31	6.82	5.97	5.98	8.48	8.55	7.54	8.01	1.88
A_09_P143950	FBtr0075294	39903	CG6664	11.22	11.78	11.65	11.58	10.89	10.98	10.94	10.95	-0.62
A_09_P143985	FBtr0070616	31349	Vap-33-1	11.79	12.14	11.95	11.88	11.39	11.32	11.42	11.61	-0.51
A_09_P144020	FBtr0304867	37129	CG43066	7.73	7.22	7.11	7.49	7.98	7.99	8.05	8.13	0.65
A_09_P144030	FBtr0100070	3885570	CG34015	9.73	9.45	9.65	9.54	8.87	8.76	8.99	9.03	-0.68
A_09_P144040	FBtr0082136	41200	RpS29	15.94	15.82	15.88	15.88	16.04	16.01	16.10	15.98	0.16
A_09_P144115	FBtr0084121	42549	InR	6.61	5.36	5.39	5.36	8.46	8.50	7.83	7.58	2.41
A_09_P144195	FBtr0310632	38493	Rop	10.93	10.90	10.93	10.89	10.62	10.19	10.41	10.49	-0.49
A_09_P144210	FBtr0100109	3885601	CG34054	8.68	8.13	8.28	8.31	9.37	9.38	9.09	9.14	0.89

A_09_P144230	FBtr0303157	36205	CG42336	10.48	10.69	10.55	10.50	10.20	9.77	9.90	9.86	-0.63
A_09_P144340	FBtr0333464	40390	CoVIII	14.96	14.94	15.02	14.94	14.59	14.46	14.65	14.88	-0.32
A_09_P144345	FBtr0334319	45826	bel	12.71	12.60	12.71	12.28	12.20	11.80	11.98	11.93	-0.60
A_09_P144445	FBtr0079146	33824	Gpdh	13.56	13.33	13.25	13.34	12.73	12.33	12.66	12.68	-0.77
A_09_P144555	FBtr0110801	42747	CG4467	7.62	7.67	7.57	7.69	8.10	8.42	8.23	8.10	0.57
A_09_P144590	FBtr0076915	38784	Dbi	15.41	15.26	15.27	15.26	15.77	15.66	15.63	15.55	0.35
A_09_P144705	FBtr0343121	33850	CG9135	9.24	9.83	9.83	9.89	9.01	8.74	8.97	9.16	-0.73
A_09_P144735	FBtr0333324	48971	Atpalpha	9.70	9.01	9.09	9.24	10.06	10.11	10.07	9.90	0.78
A_09_P144790	FBtr0079146	33824	Gpdh	13.78	13.57	13.56	13.68	13.38	13.05	13.19	13.21	-0.44
A_09_P144810	FBtr0305605	32136	CG43154	7.73	7.97	7.19	7.59	8.54	8.59	8.38	8.17	0.80
A_09_P144815	FBtr0333261	32083	dlg1	10.92	11.26	10.84	11.24	10.40	10.39	10.33	10.43	-0.68
A_09_P144825	FBtr0077440	50178	CG15434	6.00	5.62	5.88	5.87	5.30	3.88	5.02	4.61	-1.14
A_09_P144915	FBtr0076614	38961	Cbl	7.63	7.52	6.86	7.38	5.83	6.42	6.06	6.12	-1.24
A_09_P144930	FBtr0081932	41058	CG9821	14.67	14.87	14.75	14.81	16.08	16.23	15.88	15.43	1.13
A_09_P144995	FBtr0308806	43841	unc-13	8.50	8.79	8.72	8.88	9.18	9.45	9.10	8.99	0.46
A_09_P145010	FBtr0073249	38513	ImpL2	13.31	13.18	13.19	13.10	14.85	14.71	14.51	14.52	1.45
A_09_P145075	FBtr0289998	43300	side	7.98	7.67	7.57	7.49	8.28	8.44	8.41	8.54	0.74
A_09_P145120	FBtr0113769	3354992	CG2893	6.85	6.83	6.70	6.75	5.19	5.53	5.94	6.16	-1.08
A_09_P145160	NR_004369	5740367	CR40677	11.79	13.22	11.05	12.51	14.13	14.59	13.50	13.88	1.88
A_09_P145200	FBtr0339250	42843	KrT95D	10.12	10.20	10.11	10.26	10.77	10.72	10.54	10.34	0.42
A_09_P145230	FBtr0301666	32552	CG8909	7.63	7.44	7.63	7.17	7.68	8.16	8.12	8.19	0.57
A_09_P145285	FBtr0330164	43127	Tsp96F	9.04	9.04	9.15	9.04	8.51	8.03	8.41	7.95	-0.84
A_09_P145300	FBtr0077531	49638	drm	10.08	9.99	10.18	9.92	10.88	10.88	10.56	10.24	0.60
A_09_P145315	FBtr0078318	40336	park	9.50	9.79	9.69	9.78	8.70	8.93	8.90	9.29	-0.74
A_09_P145385	FBtr0089348	44548	lola	10.00	10.40	10.21	10.43	9.91	10.00	9.77	9.83	-0.38
A_09_P145390	FBtr0074839	40232	polo	9.12	10.19	9.75	10.06	8.70	8.40	8.39	8.22	-1.36
A_09_P145400	FBtr0100058	3885611	nimB3	13.45	14.20	13.99	14.31	12.72	13.05	12.94	12.91	-1.08
A_09_P145420	FBtr0112506	5740536	CG34310	10.49	10.15	10.37	10.13	9.50	9.49	9.53	9.68	-0.73
A_09_P145425	FBtr0332879	38231	alpha-Spec	12.19	12.13	11.73	12.36	12.61	12.67	12.84	12.41	0.53
A_09_P145445	FBtr0333416	34189	Acer	8.48	8.58	8.70	8.49	6.77	7.04	7.37	7.97	-1.27
A_09_P145555	FBtr0303902	192507	CG4747	11.09	10.86	11.09	11.21	10.60	10.65	10.53	10.57	-0.47

A_09_P145580	FBtr0305095	39198	A2bp1	7.04	6.99	6.79	6.76	7.57	7.69	7.37	7.25	**0.58**
A_09_P145600	FBtr0331202	33427	VGlut	9.38	8.91	9.06	8.89	9.46	9.47	9.59	9.61	**0.47**
A_09_P145680	NM_165795	36129	CG7220	8.24	8.24	7.76	7.96	9.77	9.59	9.16	8.72	**1.26**
A_09_P145710	FBtr0080023	34427	KdelR	10.97	11.10	10.91	11.37	10.02	9.93	10.48	10.55	**-0.84**
A_09_P145715	FBtr0076539	39049	bol	6.93	6.65	6.75	6.23	3.13	3.18	4.73	5.47	**-2.51**
A_09_P145730	FBtr0071771	37536	Vps35	11.75	11.99	11.88	11.95	11.26	11.06	11.35	11.51	**-0.60**
A_09_P145780	FBtr0304114	34453	Trim9	5.40	4.94	5.32	5.29	6.43	6.43	6.32	6.03	**1.06**
A_09_P145855	FBtr0306814	39999	Eip75B	6.93	7.13	6.38	6.55	7.83	7.95	7.73	7.78	**1.07**
A_09_P145860	FBtr0339833	31766	CG1636	10.12	10.20	10.04	10.22	9.33	9.37	9.61	10.00	**-0.57**
A_09_P145930	FBtr0331254	3772180	Sxl	9.38	9.30	9.50	9.20	9.69	9.73	9.62	9.53	**0.30**
A_09_P145955	FBtr0308309	32468	cngl	9.11	8.76	9.02	8.74	10.13	10.49	10.07	10.04	**1.27**
A_09_P146006	FBtr0333346	33944	homer	7.59	7.96	7.83	7.95	7.31	6.80	7.05	7.04	**-0.78**
A_09_P146035	FBtr0343662	34779	CG9008	10.76	10.01	10.60	10.27	11.46	11.58	11.21	10.85	**0.87**
A_09_P146075	FBtr0088007	36321	RpS11	11.21	11.35	11.40	11.36	11.17	10.97	11.12	11.12	**-0.24**
A_09_P146250	FBtr0080558	34811	CG16885	15.89	16.03	16.07	16.13	15.46	15.81	15.46	15.09	**-0.57**
A_09_P146265	FBtr0075784	39552	ptip	7.47	7.78	7.61	7.69	6.93	7.42	7.12	7.27	**-0.45**
A_09_P146365	FBtr0084507	42843	KrT95D	6.16	6.28	5.87	5.78	7.15	7.14	6.65	6.69	**0.89**
A_09_P146382	FBtr0335408	43694	CG12054	8.08	8.39	7.99	8.05	7.28	7.82	7.24	7.09	**-0.77**
A_09_P146430	FBtr0302697	39268	CG42671	9.43	10.20	9.51	10.03	8.92	9.15	8.47	8.40	**-1.06**
A_09_P146540	FBtr0114583	41217	Glut4EF	9.85	8.91	9.47	8.72	10.56	11.07	10.50	10.33	**1.38**
A_09_P146560	FBtr0085582	43582	Tpi	6.18	6.35	6.36	6.41	4.25	4.30	4.19	5.21	**-1.84**
A_09_P146625	FBtr0089647	33692	Cf2	8.03	8.51	8.22	8.63	7.57	7.75	7.61	7.83	**-0.66**
A_09_P146640	FBtr0089194	43822	CG11155	9.00	9.46	9.28	9.26	8.51	8.06	8.08	8.16	**-1.05**
A_09_P146685	FBtr0344383	41783	kibra	9.50	9.25	9.23	9.06	10.43	10.86	10.17	10.10	**1.13**
A_09_P146770	FBtr0339557	33128	CG14621	8.07	8.55	8.05	8.38	6.96	7.05	7.20	7.64	**-1.05**
A_09_P146800	FBtr0072242	37767	Ssrp	12.45	12.51	12.49	12.67	12.97	13.08	13.26	13.49	**0.67**
A_09_P146880	FBtr0075365	39847	Syx8	10.50	10.67	10.58	10.63	11.41	11.53	11.08	11.05	**0.67**
A_09_P146900	FBtr0339401	31235	Vha36-3	10.68	10.78	10.86	10.92	10.69	10.50	10.47	10.29	**-0.32**
A_09_P146925	FBtr0339975	43241	Tsp97E	10.57	10.42	10.39	10.46	10.31	10.27	10.29	10.22	**-0.19**
A_09_P146930	FBtr0074838	40231	CG6597	10.32	10.49	10.44	10.55	9.85	10.06	9.97	10.05	**-0.47**
A_09_P147045	FBtr0074258	32573	Nipsnap	13.45	13.98	13.74	13.93	13.02	12.74	13.01	13.39	**-0.73**

A_09_P147050	FBtr0084103	42525	AP-2sigma	11.05	11.00	10.83	11.03	10.43	10.51	10.56	10.77	**-0.41**
A_09_P147070	FBtr0086102	35473	TpnC41C	7.06	7.53	7.19	7.41	4.53	5.17	5.85	6.33	**-1.83**
A_09_P147227	FBtr0308363	38485	CG14989	7.03	6.87	6.51	6.71	7.35	7.30	7.55	7.63	**0.68**
A_09_P147350	FBtr0088548	35971	ced-6	12.22	12.29	12.01	12.18	13.01	13.10	12.69	12.55	**0.66**
A_09_P147455	FBtr0074238	32595	Cyp1	14.26	14.29	14.19	14.46	13.58	13.81	14.09	14.05	**-0.41**
A_09_P147745	FBtr0299658	34665	bun	11.76	11.72	11.55	11.79	13.26	13.61	12.89	12.40	**1.33**
A_09_P147865	FBtr0302509	39950	CG7580	15.30	15.32	15.58	15.34	14.78	14.81	14.99	15.10	**-0.47**
A_09_P148000	FBtr0300502	46078	vlc	9.32	9.48	9.42	9.47	8.85	8.76	9.02	9.05	**-0.50**
A_09_P148150	FBtr0083810	42339	CG6231	8.78	8.54	8.41	8.28	8.78	9.43	9.25	9.42	**0.72**
A_09_P148180	FBtr0086085	35486	sxc	11.11	11.15	11.20	11.28	10.94	10.66	10.85	10.76	**-0.38**
A_09_P148200	FBtr0077717	33469	Taf10	12.15	12.36	12.34	12.47	11.67	11.94	12.02	12.11	**-0.39**
A_09_P148205	NM_143178	43125	CG5886	12.35	12.52	12.43	12.48	10.66	10.48	11.04	11.51	**-1.52**
A_09_P148580	FBtr0112946	31678	CHES-1-like	9.39	8.93	8.98	9.03	10.85	10.92	10.39	9.76	**1.40**
A_09_P148645	FBtr0299709	40620	cno	8.65	8.69	8.47	8.54	8.95	8.85	9.01	9.00	**0.37**
A_09_P148655	FBtr0075077	40056	CG3961	11.29	12.65	12.08	12.56	10.32	9.90	9.82	9.68	**-2.21**
A_09_P148740	FBtr0343568	31381	rb	9.49	9.78	9.61	9.71	8.99	9.00	9.12	9.14	**-0.59**
A_09_P148800	FBtr0070182	31052	CDC45L	10.45	10.35	10.43	10.26	9.81	10.06	10.03	10.14	**-0.36**
A_09_P148805	FBtr0088591	35946	Cyp4p2	6.74	6.87	5.92	4.97	7.86	8.26	7.65	7.23	**1.63**
A_09_P148995	FBtr0084692	42946	asp	8.17	8.82	8.87	9.14	7.81	7.69	7.57	7.50	**-1.10**
A_09_P149160	FBtr0084255	42644	Pebp1	16.06	16.12	16.04	16.04	15.87	15.78	15.75	15.63	**-0.31**
A_09_P149195	NM_080427	47219	RpS14b	13.79	13.80	13.68	13.90	14.21	14.21	14.08	13.91	**0.31**
A_09_P149215	FBtr0073415	32009	Imp	10.32	10.26	10.26	9.97	10.69	10.75	10.46	10.72	**0.45**
A_09_P149290	FBtr0339241	43220	CG6403	13.84	13.74	13.94	13.81	14.91	14.54	14.64	14.52	**0.82**
A_09_P149300	FBtr0087445	36648	CG10200	14.82	14.72	14.73	14.96	15.21	15.53	15.42	15.35	**0.57**
A_09_P149345	FBtr0080917	44887	mdy	11.69	11.35	11.33	11.31	11.01	10.49	11.07	10.90	**-0.55**
A_09_P149410	FBtr0080022	34427	KdelR	11.43	11.94	11.64	11.82	11.02	11.08	10.98	11.45	**-0.57**
A_09_P149520	FBtr0078860	40581	Karybeta3	13.82	13.93	13.87	13.99	14.43	14.44	14.18	14.07	**0.38**
A_09_P149595	AF223381	39136	SH3PX1	11.66	11.73	11.69	11.78	12.20	12.16	12.02	11.84	**0.34**
A_09_P149635	FBtr0088058	36270	ERp60	9.15	9.29	9.38	9.08	7.48	6.83	8.09	8.62	**-1.47**
A_09_P149660	FBtr0089414	42127	alt	11.62	11.82	11.97	11.70	10.91	11.16	11.40	11.48	**-0.54**
A_09_P149680	FBtr0089172	43770	Ank	12.02	12.19	12.35	12.34	12.00	11.76	11.91	11.72	**-0.38**

A_09_P149685	FBtr0076593	39002	Prm	16.01	16.17	16.45	16.33	15.65	15.39	15.83	15.79	**-0.57**
A_09_P149780	FBtr0301416	33277	IA-2	11.78	11.36	11.12	11.46	12.60	12.92	12.67	12.82	**1.32**
A_09_P150000	FBtr0331932	33214	RpLP1	10.09	9.97	10.31	9.93	9.53	9.58	9.71	9.84	**-0.41**
A_09_P150015	FBtr0340394	37144	CG5482	12.44	12.63	12.56	12.66	12.12	11.95	12.20	12.29	**-0.44**
A_09_P150035	FBtr0082930	41743	pr-set7	7.19	7.06	7.27	6.85	6.12	6.64	6.64	6.69	**-0.57**
A_09_P150055	FBtr0088377	36024	CG12744	8.83	9.00	8.81	9.05	8.38	8.24	8.47	8.74	**-0.47**
A_09_P150230	FBtr0333089	34658	Elf	7.90	8.03	7.82	7.60	7.32	7.47	7.53	7.46	**-0.39**
A_09_P150245	FBtr0308954	38963	Unr	10.73	11.24	10.84	11.12	10.10	10.36	10.33	10.50	**-0.66**
A_09_P150255	FBtr0302785	39889	Lmpt	13.36	13.79	13.62	13.81	13.29	13.01	13.05	13.06	**-0.54**
A_09_P150275	FBtr0083018	41785	eff	11.64	11.71	11.65	11.47	11.16	10.45	11.04	11.19	**-0.66**
A_09_P150315	FBtr0340424	32434	CG5548	7.66	7.76	7.72	7.57	6.60	7.45	6.98	6.87	**-0.70**
A_09_P150365	FBtr0082947	41737	trx	6.55	6.92	6.27	6.57	7.32	7.16	7.09	7.25	**0.63**
A_09_P150455	FBtr0085693	43616	aralar1	13.79	13.92	13.98	13.81	13.04	13.09	13.01	12.94	**-0.85**
A_09_P150480	FBtr0083018	41785	eff	9.65	9.60	9.54	9.23	9.07	8.42	8.82	8.93	**-0.69**
A_09_P150515	FBtr0332420	53433	Vhl	9.45	9.89	9.79	9.80	9.05	9.26	9.19	9.41	**-0.51**
A_09_P150735	FBtr0333925	43177	CG31324	8.64	7.91	8.21	7.68	9.33	9.80	9.59	9.36	**1.41**
A_09_P150870	FBtr0080641	34837	mTTF	11.78	11.68	11.77	11.59	11.45	11.42	11.56	11.53	**-0.21**
A_09_P151080	FBtr0070100	30984	pcl	10.22	9.63	10.00	9.94	12.60	12.00	11.82	10.99	**1.90**
A_09_P151100	FBtr0331686	42018	npf	7.59	6.88	7.08	6.76	8.40	8.42	8.22	8.16	**1.22**
A_09_P151125	FBtr0301151	34059	CG7179	9.75	6.99	8.52	7.09	10.03	10.32	10.67	11.01	**2.42**
A_09_P151205	FBtr0087744	36467	GstE14	6.98	7.63	7.22	7.66	6.05	5.31	6.26	6.41	**-1.36**
A_09_P151640	FBtr0290033	35889	CG8247	7.99	8.21	7.96	7.90	6.99	7.03	7.53	7.78	**-0.68**
A_09_P152205	FBtr0085739	43678	CG11318	6.41	5.38	5.60	4.85	7.54	7.70	6.81	6.88	**1.67**
A_09_P152280	FBtr0306162	35055	CG5110	6.71	6.72	6.50	6.67	6.04	6.06	6.03	6.29	**-0.55**
A_09_P153025	FBtr0070371	45250	deltaCOP	12.72	12.80	12.53	12.85	11.96	11.95	12.41	12.52	**-0.51**
A_09_P153030	FBtr0080536	34784	CG16863	7.81	8.05	8.17	8.22	7.61	7.66	7.65	7.84	**-0.37**
A_09_P153140	FBtr0079012	33739	qtc	9.36	9.81	9.49	9.43	9.84	9.99	9.80	9.98	**0.38**
A_09_P153210	FBtr0074902	40186	Nca	11.09	11.37	11.18	11.40	11.06	10.88	10.92	10.98	**-0.30**
A_09_P153220	FBtr0074129	32554	Lcch3	7.21	6.85	6.96	6.72	7.48	7.75	7.55	7.61	**0.66**
A_09_P153295	FBtr0089512	48971	Atpalpha	9.08	8.64	8.67	8.66	9.62	9.72	9.36	9.52	**0.79**
A_09_P153320	FBtr0343403	34065	Herp	10.61	10.83	10.80	10.57	10.01	9.94	10.07	10.17	**-0.66**

A_09_P153410	EL877096	32434	CG5548	14.07	14.04	14.11	14.22	13.49	13.76	13.78	13.71	-0.43
A_09_P153445	FBtr0089475	41247	Syn	6.68	6.63	6.39	5.97	7.97	7.96	8.36	8.42	1.76
A_09_P153455	AF073179	33386	GlyP	12.04	12.61	12.15	12.59	11.38	10.93	11.46	11.87	-0.94
A_09_P153500	FBtr0308617	32585	eas	11.56	11.07	11.35	11.00	12.47	11.98	11.87	11.78	0.78
A_09_P153540	FBtr0075753	39529	Hml	12.20	12.63	12.31	12.82	11.84	11.57	11.80	11.81	-0.74
A_09_P153830	FBtr0339757	36486	IM10	7.34	5.29	3.60	3.21	12.17	12.18	11.50	10.62	6.76
A_09_P153840	FBtr0088058	36270	ERp60	15.15	15.16	15.14	15.17	13.86	13.85	14.57	14.94	-0.85
A_09_P153855	FBtr0084214	42620	PyK	15.98	16.03	15.99	16.04	15.26	15.32	15.64	15.86	-0.49
A_09_P153930	FBtr0113827	3354930	CG40191	9.95	10.12	9.77	10.02	9.22	8.68	9.28	9.47	-0.80
A_09_P154010	FBtr0307504	59175	CG18814	10.15	9.78	9.96	9.87	8.49	8.75	9.13	9.52	-0.97
A_09_P154070	FBtr0074643	32881	CG7288	10.75	10.80	10.74	10.85	10.57	10.29	10.48	10.52	-0.32
A_09_P154345	FBtr0343875	45830	bur	10.64	10.72	10.73	10.80	10.33	10.38	10.36	10.26	-0.39
A_09_P154620	FBtr0333139	38467	CG14984	12.86	13.00	12.80	12.99	13.56	13.57	13.24	13.37	0.52
A_09_P154825	FBtr0334065	39625	mnd	11.43	10.81	11.09	10.87	9.35	9.83	10.13	10.50	-1.10
A_09_P160070	FBtr0301436	41616	2mit	8.12	7.90	7.98	8.08	9.21	9.53	9.11	9.00	1.19
A_09_P160195	FBtr0302886	318840	CG33062	6.11	6.57	6.54	6.65	5.25	5.30	5.68	5.79	-0.96
A_09_P160440	FBtr0299849	39630	Sytbeta	7.28	6.60	6.85	6.36	7.49	7.47	7.59	7.48	0.74
A_09_P160565	FBtr0303445	10178782	CG31808	10.66	10.33	10.54	10.37	11.19	10.99	10.94	10.90	0.53
A_09_P160600	FBtr0299863	7354472	CG42340	6.07	5.60	5.57	5.55	6.76	7.53	6.79	6.80	1.27
A_09_P160620	FBtr0071616	37431	CG9752	7.58	7.68	7.54	7.42	5.72	5.43	6.54	6.95	-1.39
A_09_P160680	FBtr0329846	35277	sick	6.81	6.84	6.43	6.38	8.05	8.08	7.88	7.99	1.39
A_09_P160715	FBtr0076093	39284	chrb	6.09	6.59	6.22	5.64	7.30	7.50	6.72	7.07	1.01
A_09_P160800	FBtr0085384	43447	Pglym78	10.50	10.81	10.88	10.93	8.44	8.40	9.38	9.65	-1.81
A_09_P160810	FBtr0273450	35031	beat-IIIb	6.52	5.26	5.76	5.49	7.02	7.20	6.88	6.63	1.17
A_09_P160845	FBtr0307181	318224	CG32815	8.27	8.57	8.29	8.57	8.75	8.99	8.72	8.77	0.38
A_09_P160915	FBtr0333484	46015	tutl	9.02	9.18	9.19	8.98	9.89	9.99	9.60	9.86	0.74
A_09_P160940	FBtr0307030	33968	Hrb27C	9.98	10.24	10.08	10.16	9.86	9.78	9.70	9.87	-0.32
A_09_P161375	FBtr0084845	318737	Muc96D	8.21	8.59	8.42	8.57	7.87	7.91	7.80	7.73	-0.62
A_09_P161480	FBtr0301748	31713	CG42593	9.23	9.26	8.95	9.16	8.70	8.80	8.90	8.94	-0.32
A_09_P161705	FBtr0340656	43892	sif	5.29	4.35	4.73	3.66	5.73	5.91	5.56	5.88	1.26
A_09_P161750	FBtr0112738	5740179	CG34437	8.50	8.99	8.67	8.97	6.76	6.96	7.67	7.98	-1.44

A_09_P161810	FBtr0333602	31826	rdgA	7.77	7.92	7.70	7.76	8.50	8.63	8.44	8.17	0.65
A_09_P161975	FBtr0303231	5740165	CG34427	8.29	8.08	8.51	8.25	6.08	5.98	7.63	7.86	-1.39
A_09_P162065	FBtr0331841	39284	chrb	9.33	9.65	9.07	9.34	10.62	10.91	10.16	10.00	1.08
A_09_P162115	FBtr0074110	32544	Gbeta13F	9.85	10.02	9.63	9.65	9.39	9.03	9.26	9.39	-0.52
A_09_P162170	FBtr0332394	32923	CG8028	7.28	7.52	7.83	7.47	6.04	6.73	6.64	7.13	-0.89
A_09_P162175	FBtr0112873	42567	CASK	8.48	8.87	8.56	8.81	7.38	8.16	7.77	8.26	-0.79
A_09_P162185	FBtr0332962	31217	CG2865	9.28	8.02	8.00	7.70	10.26	10.35	9.89	9.57	1.77
A_09_P162235	FBtr0332934	31948	CG43902	5.79	5.51	5.43	4.74	6.27	6.89	6.43	6.52	1.16
A_09_P162430	FBtr0080720	34885	vig	11.38	11.27	11.30	10.80	10.70	10.77	10.43	10.67	-0.54
A_09_P162650	FBtr0309853	41236	CG5361	6.50	7.89	7.12	7.80	5.59	5.95	5.82	5.78	-1.54
A_09_P162715	FBtr0081932	41058	CG9821	9.63	9.57	8.90	9.37	11.25	11.22	10.77	10.51	1.57
A_09_P162870	FBtr0301327	42567	CASK	8.23	8.14	7.70	8.20	8.62	8.87	8.68	8.64	0.64
A_09_P162955	FBtr0073415	32009	Imp	12.25	11.98	11.92	11.95	12.63	12.79	12.63	12.72	0.67
A_09_P162980	FBtr0088218	36129	CG7220	11.50	11.22	11.25	11.31	12.47	12.48	12.18	11.80	0.91
A_09_P163160	FBtr0333267	38103	CG32479	10.29	10.46	10.18	10.32	9.79	9.73	9.92	10.15	-0.41
A_09_P163180	FBtr0300959	35670	fa2h	7.52	6.43	7.02	6.33	8.66	7.99	8.02	7.74	1.28
A_09_P163350	FBtr0076416	39132	CG6767	9.87	9.62	9.60	9.90	9.17	9.30	9.34	9.13	-0.51
A_09_P163655	FBtr0111188	3355162	CG40486	7.01	7.67	7.40	8.03	6.00	5.46	6.33	6.68	-1.41
A_09_P163840	FBtr0308812	40051	CG14074	6.62	6.57	6.50	6.29	5.86	5.74	5.92	6.05	-0.60
A_09_P163900	FBtr0084828	43059	CG13659	4.88	4.56	4.32	4.08	6.26	5.72	6.03	6.29	1.62
A_09_P164185	FBtr0335005	43531	CG15514	8.71	8.97	9.02	8.73	8.43	8.26	8.32	8.47	-0.49
A_09_P164220	FBtr0112777	37962	egg	6.87	7.86	7.22	7.74	4.70	5.67	5.15	4.97	-2.30
A_09_P164225	FBtr0339716	59246	CG18731	11.49	11.55	11.44	11.58	10.53	10.84	11.09	11.29	-0.58
A_09_P164245	FBtr0075361	39854	CG4098	6.81	6.77	6.42	6.33	4.93	4.63	4.39	4.85	-1.88
A_09_P164265	FBtr0334599	42900	Dis3	11.93	11.58	11.87	11.68	12.06	12.23	12.04	12.09	0.34
A_09_P164285	FBtr0086502	37244	TBCB	9.17	9.34	9.42	9.38	8.80	8.44	8.81	8.87	-0.60
A_09_P164330	FBtr0080108	34474	CG6415	11.76	12.26	11.96	12.05	9.39	9.64	10.37	10.79	-1.96
A_09_P164335	FBtr0340026	31578	CG3446	14.74	14.64	14.77	14.73	14.05	14.12	14.36	14.48	-0.46
A_09_P164340	FBtr0333672	43829	ATPsyn-beta	15.37	15.15	15.41	15.03	14.60	13.83	14.70	14.83	-0.75
A_09_P164345	FBtr0080052	2768916	CG33303	11.16	11.32	11.04	11.01	10.07	9.40	10.42	10.91	-0.93
A_09_P164390	FBtr0081143	48805	Catsup	10.17	10.56	10.26	10.30	9.37	8.89	9.61	9.89	-0.88

A_09_P164395	FBtr0083052	41836	Caf1	7.72	7.83	7.69	7.47	6.74	6.01	6.81	7.26	-0.97
A_09_P164400	FBtr0086156	35590	SdhB	11.68	11.70	12.18	11.77	11.25	11.09	11.30	11.33	-0.59
A_09_P164405	FBtr0089724	31006	mod(r)	8.15	8.44	8.44	8.38	7.85	7.79	7.83	7.82	-0.53
A_09_P164415	FBtr0084852	43065	CG10560	11.09	11.23	11.35	11.49	8.55	7.96	9.56	10.14	-2.23
A_09_P164420	FBtr0111273	3355145	CG12547	8.01	8.50	8.59	8.06	7.65	7.21	7.30	7.49	-0.88
A_09_P164430	FBtr0087536	36581	CG8503	8.20	7.88	8.38	7.13	6.61	6.13	6.51	6.69	-1.42
A_09_P164450	FBtr0070828	31519	CG3016	7.48	7.65	7.67	7.16	6.85	6.35	6.61	6.72	-0.86
A_09_P164465	FBtr0083009	41802	CG7265	9.90	9.86	9.97	9.87	9.55	9.39	9.46	9.59	-0.41
A_09_P164515	FBtr0086771	37084	CG5721	9.15	9.37	9.25	9.30	8.91	8.72	8.80	9.06	-0.40
A_09_P164520	FBtr0085680	43635	Rpt6R	7.27	7.71	7.40	7.61	7.14	5.93	6.65	6.84	-0.86
A_09_P164530	FBtr0113259	42510	CG5802	9.81	9.91	9.96	9.60	8.79	8.37	9.08	9.44	-0.90
A_09_P164590	FBtr0334535	42000	CSN5	10.82	10.99	10.94	10.88	10.52	10.00	10.37	10.45	-0.57
A_09_P164595	FBtr0074961	40145	Oat	14.23	14.86	14.53	14.83	11.46	11.46	12.74	13.26	-2.38
A_09_P164610	FBtr0070026	33017	CG9581	8.58	8.78	8.56	8.46	8.10	7.53	8.01	8.32	-0.60
A_09_P164660	FBtr0070690	31399	Torsin	9.28	9.16	9.21	9.08	8.21	7.93	8.49	8.62	-0.87
A_09_P164700	FBtr0082044	41132	GstZ1	9.58	9.44	9.68	9.52	7.28	7.21	8.25	8.71	-1.69
A_09_P164705	FBtr0080025	34430	TfIIB	10.00	9.98	10.31	10.07	9.66	9.10	9.59	9.54	-0.62
A_09_P164735	FBtr0080051	34411	cdc2	10.74	11.39	11.01	11.55	10.48	9.84	10.24	10.26	-0.97
A_09_P164765	FBtr0088053	36277	PI31	8.54	8.80	8.74	8.56	8.14	7.76	8.17	8.26	-0.58
A_09_P164825	FBtr0082504	41454	CG3397	7.26	8.45	7.45	7.99	3.64	4.88	3.56	3.86	-3.80
A_09_P164870	FBtr0088593	35948	Cyp4p3	10.06	10.10	9.97	9.98	9.55	9.35	9.43	9.53	-0.56
A_09_P164980	FBtr0071223	31806	Ogg1	8.56	8.31	8.30	8.45	7.38	7.63	7.78	8.05	-0.70
A_09_P165020	FBtr0073143	38429	CG14971	7.28	7.92	7.78	7.60	6.43	6.56	6.43	6.58	-1.15
A_09_P165055	FBtr0082140	41205	CG8507	10.80	10.89	10.96	10.75	9.74	9.37	10.08	10.29	-0.98
A_09_P165105	FBtr0100323	3772232	fabp	13.14	12.83	13.06	12.79	12.39	12.12	12.55	12.65	-0.53
A_09_P165225	FBtr0075972	39426	CG10646	7.31	7.01	7.19	6.88	5.58	6.01	6.14	6.68	-1.00
A_09_P165255	FBtr0084265	42661	CSN6	10.07	9.97	10.13	9.91	9.37	8.99	9.39	9.43	-0.73
A_09_P165285	FBtr0083707	42273	dnk	11.58	11.30	11.42	11.43	10.69	10.88	10.99	11.24	-0.48
A_09_P165350	FBtr0331989	5740629	CG43795	6.00	5.91	5.76	5.30	6.73	6.95	6.68	6.87	1.06
A_09_P165460	FBtr0086078	35475	CG3107	7.13	7.11	7.38	6.72	6.05	6.05	5.71	6.42	-1.03
A_09_P165470	FBtr0078712	40696	MED27	8.52	8.62	8.71	8.58	8.34	7.68	8.20	8.32	-0.47

A_09_P165710	FBtr0334094	46260	l(3)neo18	13.96	13.94	14.10	14.09	13.55	13.45	13.59	13.71	**-0.44**
A_09_P165940	FBtr0074178	32539	Aats-arg	9.40	8.94	8.83	8.66	8.03	6.98	7.83	8.45	**-1.13**
A_09_P166085	FBtr0083078	41853	Tm2	13.19	13.44	13.53	13.45	12.25	11.20	12.31	12.37	**-1.37**
A_09_P166110	FBtr0079937	34338	Pen	8.62	9.43	9.04	9.50	8.21	7.57	7.87	7.91	**-1.26**
A_09_P166120	FBtr0334716	42460	Syp	5.66	6.17	6.58	6.97	4.33	3.12	3.22	3.57	**-2.79**
A_09_P166190	FBtr0300575	36778	Asph	9.37	9.74	9.51	9.46	7.68	7.05	8.05	8.54	**-1.69**
A_09_P166275	FBtr0330098	39135	CG16711	6.91	7.41	7.39	7.36	5.95	6.29	6.14	6.59	**-1.03**
A_09_P166410	FBtr0075992	39375	ssp	8.91	8.95	9.14	8.85	8.19	8.12	8.16	8.22	**-0.79**
A_09_P166540	FBtr0085582	43582	Tpi	12.60	13.13	13.08	13.16	11.26	10.86	11.66	12.00	**-1.54**
A_09_P166580	FBtr0088153	45680	Tapdelta	12.85	12.84	12.87	12.73	11.70	11.53	12.31	12.58	**-0.79**
A_09_P166780	FBtr0078377	40395	CG7172	12.19	12.05	12.29	12.21	11.54	11.38	11.53	11.68	**-0.65**
A_09_P166825	FBtr0082103	41166	Crc	13.48	13.58	13.57	13.40	12.38	11.98	12.81	13.14	**-0.93**
A_09_P166835	FBtr0076649	38984	CG5989	8.99	9.03	8.83	8.72	8.37	8.25	8.44	8.47	**-0.51**
A_09_P166935	FBtr0334547	43676	CG11317	6.58	6.56	6.43	6.00	7.24	7.98	7.24	7.25	**1.03**
A_09_P166965	FBtr0330645	33174	CG31974	13.80	12.97	13.36	12.98	14.07	13.66	14.05	14.13	**0.70**
A_09_P167115	FBtr0074352	2768882	CG9125	7.68	7.80	7.82	7.55	7.19	7.20	7.26	7.37	**-0.46**
A_09_P167145	FBtr0084672	42930	CG13624	7.50	8.20	7.91	7.70	9.42	9.46	8.84	8.56	**1.24**
A_09_P167170	FBtr0072145	37807	CG2970	10.17	9.96	10.17	9.95	9.62	9.11	9.65	9.86	**-0.50**
A_09_P167195	FBtr0077683	33495	NTPase	13.46	13.40	13.41	13.37	13.16	13.14	13.16	13.23	**-0.23**
A_09_P167235	FBtr0305989	37798	Nap1	13.37	13.64	13.81	13.64	14.33	14.07	14.16	13.84	**0.49**
A_09_P167250	FBtr0086669	37106	GstE1	11.40	11.50	11.45	11.34	12.00	11.73	11.90	12.04	**0.50**
A_09_P167320	FBtr0110850	31899	CG32699	11.58	11.42	11.43	11.31	10.42	10.48	10.92	11.25	**-0.67**
A_09_P167490	FBtr0333501	38109	Glut1	6.03	5.52	5.88	5.28	6.34	6.65	6.76	6.58	**0.91**
A_09_P167575	FBtr0334567	5740131	CG34383	9.60	9.81	9.50	9.82	10.26	10.41	10.51	10.76	**0.80**
A_09_P167580	FBtr0071293	31841	CG7039	8.21	8.26	8.39	8.31	7.65	7.72	7.93	8.02	**-0.46**
A_09_P167635	FBtr0078748	40680	exba	9.96	10.31	10.21	9.88	9.64	8.37	8.68	9.07	**-1.15**
A_09_P167720	FBtr0082780	41625	Ace	5.36	5.80	4.92	4.28	6.21	6.55	6.05	6.65	**1.28**
A_09_P167765	FBtr0306610	41225	Mical	11.62	11.72	11.77	11.65	11.32	11.44	11.41	11.38	**-0.30**
A_09_P167780	FBtr0331960	48311	Pkc53E	8.46	7.89	8.08	7.81	8.61	8.54	8.72	8.90	**0.63**
A_09_P167810	FBtr0301287	35211	fon	14.72	15.10	15.06	14.92	13.47	13.37	13.84	13.99	**-1.28**
A_09_P167985	FBtr0086238	2768858	dpr	6.53	5.80	5.76	6.05	7.14	7.67	7.11	7.17	**1.24**

A_09_P167995	FBtr0332138	38916	Ubc12	12.33	12.24	12.34	12.30	11.92	11.78	11.95	12.08	-0.37
A_09_P168005	FBtr0330299	43435	CG11873	7.78	8.06	7.84	7.90	8.42	8.89	8.54	8.46	0.68
A_09_P168015	FBtr0071747	246596	Tango11	8.23	8.31	8.27	7.96	7.57	7.00	7.48	7.66	-0.77
A_09_P168040	FBtr0331649	33396	CG15385	7.28	7.85	8.06	8.15	5.96	5.87	6.35	6.67	-1.62
A_09_P168160	FBtr0334511	41599	CG7518	9.96	10.23	10.02	10.25	9.90	9.80	9.87	9.68	-0.30
A_09_P168655	FBtr0344274	40660	ksr	9.04	9.38	9.20	9.51	8.79	8.47	8.79	8.81	-0.57
A_09_P168735	FBtr0333238	34921	CG4587	5.28	5.06	5.01	4.29	6.14	6.24	5.78	6.32	1.21
A_09_P168745	FBtr0081445	35359	sky	7.11	7.53	7.50	7.24	6.68	6.73	6.36	6.49	-0.78
A_09_P168845	FBtr0089338	41087	Rel	9.82	9.70	9.57	9.94	13.03	12.58	12.14	11.25	2.49
A_09_P168875	FBtr0340159	31738	Smox	11.45	11.26	11.10	11.42	12.59	12.52	12.26	11.57	0.93
A_09_P168900	FBtr0072218	49297	Ca-P60A	7.92	8.53	8.08	7.95	6.67	6.13	6.71	6.69	-1.57
A_09_P168925	FBtr0087828	36383	Amph	10.70	10.65	10.79	10.58	10.17	10.12	10.04	10.11	-0.57
A_09_P168970	FBtr0082627	117369	desat1	13.62	13.92	13.59	13.63	13.46	13.26	13.29	13.38	-0.34
A_09_P169030	FBtr0089332	39293	Mob2	6.73	6.48	6.45	6.12	7.68	7.94	7.67	7.44	1.24
A_09_P169130	FBtr0072355	37901	CG4563	9.58	10.11	10.16	9.21	8.17	7.17	8.08	8.13	-1.88
A_09_P169210	FBtr0330416	39533	dysc	8.27	8.01	8.02	7.79	9.18	9.67	8.88	9.17	1.20
A_09_P169495	FBtr0339422	36033	Pal1	12.80	13.32	12.97	13.10	12.35	12.46	12.57	12.60	-0.55
A_09_P169535	FBtr0076534	39054	Rdl	7.91	7.31	7.33	7.18	9.11	9.23	9.15	9.30	1.77
A_09_P169640	FBtr0340412	326226	CG32647	6.93	5.97	6.74	5.90	7.72	8.33	7.60	7.64	1.44
A_09_P169790	FBtr0332413	33721	Rtnl1	12.22	12.68	12.55	12.49	11.78	11.67	11.74	11.80	-0.74
A_09_P169900	FBtr0091491	3346192	Vmat	8.56	8.08	8.27	8.04	8.76	9.13	9.03	9.03	0.75
A_09_P169995	FBtr0332422	36199	CG9062	9.02	8.86	9.25	9.22	8.82	8.50	8.77	8.50	-0.44
A_09_P170060	FBtr0089925	43533	CG7816	8.87	8.80	8.46	8.57	10.12	9.91	9.68	9.47	1.12
A_09_P170115	FBtr0083308	42004	CG14898	10.94	10.54	10.81	10.68	9.85	10.32	10.18	10.32	-0.57
A_09_P170120	FBtr0072848	44013	sls	8.91	10.03	9.75	9.51	8.45	7.84	7.91	8.57	-1.36
A_09_P170265	FBtr0310326	36002	sqa	10.01	9.89	9.99	9.81	10.57	10.43	10.31	10.06	0.42
A_09_P170375	FBtr0301061	31278	CG3588	11.98	12.37	11.98	12.35	13.09	13.35	12.88	12.83	0.87
A_09_P170665	FBtr0332119	2769001	zormin	12.22	12.46	12.29	12.43	13.04	12.79	12.87	12.75	0.51
A_09_P170695	FBtr0306644	41824	Mf	14.14	14.81	14.84	15.11	13.52	13.41	13.45	13.51	-1.26
A_09_P170700	FBtr0301340	43814	bt	14.51	14.87	15.19	15.01	13.64	13.71	13.99	14.16	-1.02
A_09_P170740	FBtr0307323	32158	cac	9.56	9.60	9.15	9.28	10.32	10.39	10.38	10.70	1.05

A_09_P170885	FBtr0334610	42646	Nrx-1	10.12	10.07	10.13	10.01	10.80	11.18	10.67	10.81	**0.78**
A_09_P170925	FBtr0334946	40700	CG1347	9.94	10.20	9.91	10.15	11.04	11.25	10.83	10.40	**0.83**
A_09_P170935	FBtr0334305	53560	cic	9.40	9.61	9.23	9.68	10.83	10.89	10.43	10.10	**1.08**
A_09_P171025	FBtr0087414	44129	hbs	9.86	9.77	9.69	9.72	9.93	10.24	10.06	10.08	**0.32**
A_09_P171075	FBtr0088776	35771	lig	10.46	10.48	10.62	10.52	9.79	9.83	10.00	9.90	**-0.64**
A_09_P171125	FBtr0300466	8674066	CG42492	9.86	9.47	9.54	9.42	10.34	10.26	10.33	10.43	**0.76**
A_09_P171285	FBtr0310320	37086	CG14499	5.17	4.04	4.71	4.03	10.00	9.20	10.06	10.14	**5.37**
A_09_P171490	FBtr0333869	3354941	CG40178	9.13	9.02	8.88	8.78	9.78	9.25	9.52	9.49	**0.56**
A_09_P171545	FBtr0080829	34962	CG17328	7.97	7.96	8.05	7.87	7.22	7.33	7.56	7.70	**-0.51**
A_09_P171590	FBtr0332409	33552	CG34394	8.00	8.39	7.87	8.20	6.37	6.55	6.81	7.44	**-1.32**
A_09_P171630	FBtr0302526	7354408	CG42364	4.86	4.55	4.31	4.12	6.30	6.62	6.16	5.92	**1.79**
A_09_P172025	FBtr0290065	39693	RhoGAP71E	9.35	9.29	9.01	9.07	9.97	10.02	9.64	9.44	**0.59**
A_09_P172040	FBtr0339764	50225	Prosap	10.11	10.34	10.05	10.26	10.61	10.68	10.60	10.49	**0.41**
A_09_P172045	FBtr0305003	41995	Pak3	11.36	11.17	10.96	11.12	12.33	12.19	11.93	11.73	**0.89**
A_09_P172560	NR_047771	12798118	CR43264	10.92	11.21	11.07	11.26	10.60	10.05	10.17	10.43	**-0.80**
A_09_P173100	FBtr0079493	34065	Herp	7.08	7.45	7.33	7.03	6.08	5.56	6.24	6.80	**-1.05**
A_09_P173135	FBtr0332638	39430	tral	8.53	8.93	8.66	8.86	7.84	8.35	8.16	8.24	**-0.60**
A_09_P173140	FBtr0340026	31578	CG3446	12.81	12.86	12.95	12.78	12.00	11.62	12.19	12.44	**-0.79**
A_09_P173305	FBtr0333577	39747	Hip14	8.25	8.38	8.31	8.23	8.95	9.02	8.73	8.58	**0.53**
A_09_P173395	FBtr0332961	32017	CG2186	8.41	8.22	8.30	8.19	7.72	7.98	7.95	7.97	**-0.38**
A_09_P173405	FBtr0072956	44030	msn	9.52	9.80	9.54	9.72	10.63	10.82	10.39	10.44	**0.93**
A_09_P173485	FBtr0081203	35219	CG13085	6.51	6.62	6.46	6.21	6.07	5.82	5.77	5.85	**-0.58**
A_09_P173505	FBtr0075171	39978	NUCB1	14.19	14.44	14.36	14.29	13.64	13.52	13.87	14.16	**-0.52**
A_09_P173910	FBtr0075453	39768	SsRbeta	10.92	11.10	11.05	10.82	9.74	9.21	10.31	10.65	**-1.00**
A_09_P173920	FBtr0073610	32133	Hsc70-3	8.29	8.72	8.56	8.04	6.51	6.89	7.19	7.87	**-1.29**
A_09_P174020	FBtr0310672	41938	Sap47	8.95	9.09	9.01	9.04	9.43	9.52	9.18	9.27	**0.33**
A_09_P174055	FBtr0303852	10178965	CG42833	8.22	8.57	8.50	8.72	7.62	8.10	7.69	7.69	**-0.73**
A_09_P174100	FBtr0082655	41569	Past1	13.37	13.18	13.21	13.37	12.91	12.90	13.01	13.01	**-0.32**
A_09_P174640	FBtr0085224	43334	Ets98B	8.74	9.02	8.59	9.07	9.23	9.86	9.52	9.39	**0.64**
A_09_P174710	FBtr0309042	44013	sls	9.82	10.25	10.03	10.17	9.48	9.63	9.53	9.52	**-0.53**
A_09_P174760	FBtr0306742	41747	NK7.1	7.10	7.14	6.54	6.94	7.39	7.65	7.50	7.28	**0.53**

A_09_P174985	FBtr0303871	34242	tai	10.05	9.86	9.96	9.86	10.35	10.61	10.41	10.22	0.46
A_09_P175325	FBtr0308814	36743	tun	8.46	8.55	8.34	8.56	9.01	8.86	9.03	9.02	0.50
A_09_P175430	FBtr0339135	42935	CG5794	8.37	8.24	8.15	8.35	7.78	7.95	8.13	7.83	-0.36
A_09_P175465	FBtr0306169	34264	Gdi	10.27	10.47	10.13	10.48	9.96	9.67	10.07	10.06	-0.40
A_09_P175470	FBtr0331605	31310	dm	9.53	9.37	9.66	8.95	10.69	10.62	10.02	10.01	0.96
A_09_P175480	FBtr0083959	42460	Syp	8.43	10.10	9.02	9.61	7.23	7.35	6.91	7.25	-2.11
A_09_P175515	FBtr0087950	36361	CG8830	11.12	11.10	10.91	11.12	10.86	10.74	10.94	10.79	-0.23
A_09_P175525	FBtr0072602	38089	CG13896	7.85	8.08	8.04	7.92	7.55	7.32	7.67	7.73	-0.40
A_09_P175550	FBtr0079774	34241	CG9586	7.93	7.94	7.61	7.90	7.09	6.73	7.03	7.30	-0.81
A_09_P175635	FBtr0304955	36384	Galpha49B	7.36	7.37	7.23	7.13	7.67	7.92	7.55	7.60	0.41
A_09_P175650	FBtr0331858	39049	bol	5.40	5.05	5.28	4.35	6.45	6.99	6.40	6.53	1.57
A_09_P175660	FBtr0301216	8674119	CG42550	7.93	8.20	7.96	8.38	6.74	7.15	7.18	7.32	-1.02
A_09_P175850	FBtr0084912	43127	Tsp96F	7.22	7.59	7.36	7.14	5.79	5.93	5.73	5.90	-1.49
A_09_P175940	FBtr0333836	39054	Rdl	8.25	7.75	7.64	7.70	8.93	9.17	8.90	9.09	1.18
A_09_P175960	FBtr0070558	31289	CG3603	8.32	8.40	8.44	8.30	6.98	6.59	7.49	7.79	-1.16
A_09_P176095	FBtr0302692	44258	ps	8.06	8.14	8.15	7.67	8.85	9.16	8.47	8.55	0.76
A_09_P176120	FBtr0332594	37379	CG10543	7.55	7.36	7.34	7.34	7.96	8.24	7.99	8.03	0.66
A_09_P176125	FBtr0333289	38605	shep	9.28	9.89	9.51	9.78	10.50	10.58	10.05	10.05	0.68
A_09_P176140	FBtr0084121	42549	InR	7.84	7.03	7.05	7.02	9.92	10.16	9.54	9.11	2.45
A_09_P176155	FBtr0331841	39284	chrb	8.13	8.45	7.84	8.10	9.15	9.37	8.89	8.84	0.93
A_09_P176190	FBtr0308691	43781	RhoGAP102A	10.77	11.36	11.22	11.25	9.51	9.61	9.93	10.13	-1.36
A_09_P176230	FBtr0335495	34284	Pka-C1	13.35	13.58	13.33	13.32	13.08	13.11	12.81	12.99	-0.39
A_09_P176245	FBtr0333836	39054	Rdl	6.30	5.53	5.37	4.65	7.43	7.71	7.14	7.75	2.04
A_09_P176620	NM_001273747	12798035	CG43144	4.58	4.30	4.06	3.78	5.12	5.97	5.52	5.66	1.39
A_09_P176945	FBtr0306578	38196	n-syb	11.36	10.91	11.26	11.00	11.50	11.58	11.71	11.73	0.50
A_09_P177000	FBtr0080493	34707	DnaJ-H	7.81	8.54	7.96	8.17	7.37	6.93	7.18	7.49	-0.88
A_09_P177160	FBtr0076093	39284	chrb	5.02	5.66	4.97	5.01	6.28	6.66	6.01	6.23	1.13
A_09_P177325	FBtr0089116	43780	CG1674	11.50	12.04	11.73	11.81	9.57	8.69	9.62	10.25	-2.24
A_09_P177350	FBtr0333447	38331	Aats-pro	10.58	10.44	10.56	10.47	9.33	9.55	9.86	10.26	-0.76
A_09_P177575	FBtr0087437	36636	AttA	10.62	11.66	10.40	11.20	15.67	14.88	15.38	14.89	4.23
A_09_P177590	FBtr0075076	40059	CG3902	9.62	10.00	10.06	9.93	8.91	7.77	8.44	8.37	-1.53

A_09_P177615	FBtr0085744	43673	dco	6.58	6.91	6.76	6.66	5.70	4.96	5.56	6.23	-1.11
A_09_P177635	FBtr0088837	35733	CG1882	6.94	6.74	6.61	6.06	8.29	8.09	7.82	7.63	1.37
A_09_P178010	FBtr0088416	36059	14-3-3zeta	12.90	12.92	12.98	12.97	11.34	10.66	11.39	11.76	-1.65
A_09_P178125	FBtr0082358	41360	CG5214	14.92	15.00	15.03	14.96	14.75	14.64	14.79	14.85	-0.22
A_09_P178170	FBtr0290111	36589	Ih	8.95	8.41	8.52	8.65	9.41	9.98	9.68	9.70	1.06
A_09_P178390	FBtr0303687	32619	para	8.60	7.79	8.04	7.78	8.90	9.00	8.86	9.16	0.93
A_09_P178510	FBtr0302382	41144	by	7.29	6.52	6.62	6.31	10.64	10.85	10.23	9.63	3.66
A_09_P178620	FBtr0290235	31524	CG3011	7.30	7.71	7.46	7.19	5.76	5.87	6.23	6.90	-1.23
A_09_P178790	FBtr0075991	39374	l(3)j2D3	9.34	9.32	9.26	9.01	8.71	8.54	8.76	8.91	-0.50
A_09_P178830	FBtr0088981	35681	CG12736	10.58	10.57	10.77	10.36	10.35	9.92	10.12	10.19	-0.42
A_09_P178980	FBtr0302651	39900	Exn	10.44	10.13	10.32	10.19	11.26	11.43	10.91	10.66	0.79
A_09_P179165	FBtr0302695	44258	ps	13.18	13.09	12.98	13.19	13.71	14.05	13.63	13.44	0.60
A_09_P179295	FBtr0078769	40654	RpL35A	15.67	15.75	15.71	15.76	15.85	15.84	15.90	15.91	0.15
A_09_P179440	FBtr0087568	36583	Hsc70-5	8.83	8.59	8.47	7.92	7.89	6.84	7.40	7.74	-0.99
A_09_P179590	FBtr0343983	32475	CG6299	11.47	11.15	11.37	11.19	12.04	11.79	11.78	11.56	0.50
A_09_P179706	NM_165126	34925	l(2)35Di	8.52	8.34	8.41	8.49	7.21	7.34	7.55	7.69	-0.99
A_09_P179745	FBtr0083147	41891	CG5205	8.37	8.25	8.33	8.21	9.09	9.32	9.34	9.48	1.02
A_09_P179810	FBtr0331297	7354472	CG42340	5.67	5.71	5.48	5.43	6.65	7.35	6.50	6.71	1.23
A_09_P180025	FBtr0077368	33131	CG14614	6.24	6.38	5.93	5.98	4.66	5.35	5.23	5.60	-0.92
A_09_P180030	FBtr0085808	43713	CG11334	10.40	10.62	10.36	10.52	9.49	9.38	9.53	9.89	-0.90
A_09_P180100	FBtr0071681	37470	CG9865	8.58	8.64	8.60	8.37	7.56	7.28	7.50	7.67	-1.05
A_09_P180195	FBtr0303690	32619	para	6.25	6.00	5.76	5.52	6.75	7.34	6.80	7.12	1.12
A_09_P180200	FBtr0300709	34049	CG13795	8.64	9.42	9.10	9.50	6.82	7.26	7.47	7.85	-1.81
A_09_P180285	FBtr0075648	39651	Pdi	9.79	10.24	9.87	9.86	8.35	7.62	8.85	9.13	-1.45
A_09_P180350	FBtr0333719	32548	MSBP	9.36	9.65	9.39	9.25	8.92	8.28	8.79	9.02	-0.66
A_09_P180380	FBtr0071610	45280	cv-2	3.67	4.12	4.04	3.47	5.23	5.84	4.96	5.92	1.66
A_09_P180505	FBtr0070342	31167	CG4325	6.65	8.55	7.46	8.34	4.54	4.99	5.15	4.35	-3.00
A_09_P180905	FBtr0087871	36400	CG8768	8.66	8.55	8.51	8.40	8.07	7.85	8.15	8.19	-0.47
A_09_P181080	FBtr0310674	45398	Aldh-III	7.65	7.52	7.33	6.99	6.80	6.38	6.83	6.85	-0.66
A_09_P181435	FBtr0300174	41890	Trissin	6.20	5.42	5.44	5.42	6.48	6.79	6.63	6.72	1.04
A_09_P181475	FBtr0299768	42455	CG42322	7.95	7.67	7.55	7.45	8.43	8.89	8.82	8.89	1.10

A_09_P181495	FBtr0302917	246443	CG30082	7.54	8.20	7.66	7.88	6.37	6.82	6.45	6.55	-1.27
A_09_P181715	FBtr0075245	39930	CG6479	11.54	11.25	11.41	11.27	10.60	10.25	10.82	11.11	-0.67
A_09_P182025	FBtr0301900	37384	RIC-3	5.97	6.31	6.00	5.73	4.69	4.57	4.61	5.41	-1.18
A_09_P182035	FBtr0300369	35916	CG13742	6.72	7.05	6.57	6.60	7.16	7.95	7.88	8.07	1.03
A_09_P182350	FBtr0087059	36908	ste24a	9.44	9.27	9.21	9.10	8.54	7.76	8.45	8.83	-0.86
A_09_P182395	FBtr0076972	59223	CG18769	8.47	8.80	8.82	8.51	6.96	5.95	7.14	7.43	-1.78
A_09_P182445	FBtr0080213	34565	mre11	5.15	5.37	4.32	4.34	6.62	6.27	6.16	6.67	1.64
A_09_P182500	FBtr0086639	37166	CG15093	10.18	10.35	10.56	10.40	9.61	9.22	9.41	9.36	-0.97
A_09_P182515	NR_048352	12798076	CR43301	6.69	5.76	5.90	5.82	9.70	9.59	9.00	8.18	3.07
A_09_P182545	FBtr0070652	31377	CG3626	9.35	10.28	10.02	10.16	9.01	9.12	8.72	8.89	-1.02
A_09_P182905	FBtr0071559	50126	IM4	8.54	3.19	3.22	3.90	13.53	12.93	12.61	11.06	7.82
A_09_P183055	FBtr0304750	35768	CG14762	9.04	8.92	8.83	9.05	10.10	9.98	10.03	10.05	1.08
A_09_P183115	FBtr0081519	35408	nrv3	11.89	11.68	11.89	11.53	12.41	12.27	12.38	12.49	0.64
A_09_P183160	FBtr0343587	32120	PhKgamma	9.73	10.35	10.14	10.10	8.30	8.10	8.44	9.00	-1.62
A_09_P183215	FBtr0307598	5740544	futsch	8.51	8.58	8.89	8.50	9.77	9.83	9.19	9.33	0.91
A_09_P183365	FBtr0071289	31838	CG7033	11.64	11.64	11.79	11.45	10.83	10.36	10.89	11.13	-0.82
A_09_P183375	FBtr0073434	32045	Hsp60	8.65	8.25	8.44	7.81	7.33	6.84	7.42	7.65	-0.98
A_09_P183425	FBtr0305586	35336	Fs(2)Ket	11.82	12.22	11.94	12.25	11.26	11.41	11.48	11.67	-0.61
A_09_P183600	FBtr0076093	39284	chrb	9.43	10.28	9.91	9.52	11.05	11.50	10.59	10.99	1.25
A_09_P183665	FBtr0309247	45234	Ggamma30A	9.08	9.48	9.43	9.47	8.77	8.70	8.77	8.74	-0.62
A_09_P183705	FBtr0301698	39065	UGP	10.01	10.34	10.02	10.13	8.71	8.53	9.17	9.49	-1.15
A_09_P183750	FBtr0074909	40188	CG7433	9.83	9.87	10.14	9.81	8.13	7.20	8.38	8.75	-1.79
A_09_P183785	FBtr0074902	40186	Nca	9.86	10.24	9.98	10.05	9.84	9.54	9.63	9.67	-0.36
A_09_P183805	FBtr0080715	34890	ZnT35C	8.10	8.49	8.53	8.40	4.88	4.49	6.99	7.46	-2.42
A_09_P183900	FBtr0082627	117369	desat1	10.69	11.05	10.97	10.57	10.29	9.75	9.94	9.90	-0.85
A_09_P184010	FBtr0077121	38645	CG10576	11.49	11.51	11.51	11.26	11.12	10.86	10.99	11.18	-0.41
A_09_P184420	FBtr0343657	34778	TM9SF4	7.26	7.52	7.21	6.94	6.36	5.90	6.41	6.70	-0.88
A_09_P184430	FBtr0305356	41256	CG43143	9.58	9.60	9.32	9.34	10.53	10.31	10.16	10.19	0.84
A_09_P184505	FBtr0075082	40049	Indy	8.70	8.67	8.84	8.19	8.10	7.01	7.65	7.47	-1.04
A_09_P184540	FBtr0333267	38103	CG32479	10.32	10.54	10.18	10.27	9.69	9.56	9.80	10.20	-0.52
A_09_P184550	FBtr0343798	32928	gfA	6.34	5.97	6.01	5.52	6.44	6.81	6.67	6.98	0.76

A_09_P184735	FBtr0073562	32132	CkIIbeta	7.06	7.04	6.79	6.71	6.37	6.19	6.36	6.19	**-0.63**
A_09_P184775	FBtr0076752	38885	RNaseX25	10.14	10.38	10.27	10.29	9.55	9.44	9.70	9.62	**-0.69**
A_09_P184780	FBtr0340623	32741	CG8289	7.10	7.38	7.08	7.13	6.30	5.58	6.42	6.79	**-0.90**
A_09_P184805	FBtr0332578	35194	Aats-asn	12.70	12.73	12.63	12.70	12.56	12.45	12.53	12.52	**-0.17**
A_09_P184865	FBtr0070408	31217	CG2865	9.78	9.03	9.36	8.78	10.98	11.53	10.67	10.59	**1.71**
A_09_P184920	AF189278	40141	asf1	8.06	8.33	8.07	8.27	7.25	7.90	7.29	7.79	**-0.63**
A_09_P185115	FBtr0080558	34811	CG16885	9.48	9.96	9.82	9.84	8.84	9.20	8.57	8.16	**-1.08**
A_09_P185120	FBtr0308363	38485	CG14989	7.80	7.63	7.36	7.71	8.09	8.21	8.33	8.39	**0.63**
A_09_P185150	FBtr0073046	38399	CG11505	8.77	9.09	9.09	8.92	8.38	8.41	8.32	8.43	**-0.58**
A_09_P185205	FBtr0332858	43838	mGluRA	5.05	4.55	4.58	4.20	6.02	6.13	5.42	6.24	**1.36**
A_09_P185210	FBtr0334320	41020	Mkk4	8.26	7.90	7.74	8.03	8.89	8.80	8.63	8.39	**0.69**
A_09_P185240	FBtr0329931	34192	Sema-1a	6.93	6.99	6.45	6.75	7.58	7.79	7.45	7.70	**0.86**
A_09_P185405	FBtr0300336	49805	Spn42Da	11.18	11.41	11.29	11.36	11.84	11.48	11.64	11.59	**0.33**
A_09_P185580	FBtr0308639	38438	CG32264	6.35	6.22	6.27	6.18	6.91	6.91	7.04	7.31	**0.79**
A_09_P185630	FBtr0309056	36634	CG12858	8.97	8.60	8.66	8.55	9.17	9.03	9.35	9.23	**0.50**
A_09_P185635	FBtr0303558	31907	l(1)G0232	8.20	8.39	7.75	7.94	9.23	9.25	8.86	8.80	**0.96**
A_09_P185680	FBtr0303125	38863	Ank2	8.37	8.32	8.56	8.05	9.18	9.38	8.90	8.88	**0.76**
A_09_P185725	FBtr0330352	39527	bru-3	5.94	6.31	5.82	5.62	7.01	6.81	6.47	6.65	**0.81**
A_09_P185750	FBtr0335174	33443	CG3214	13.39	13.40	13.40	13.57	12.79	12.83	12.99	13.17	**-0.50**
A_09_P185780	FBtr0075580	39716	comm2	9.64	8.88	8.84	8.67	9.70	9.82	9.97	10.09	**0.89**
A_09_P185850	FBtr0079571	318841	Bsg	7.58	7.44	7.50	6.98	8.40	8.53	7.97	8.21	**0.90**
A_09_P185930	FBtr0087074	36889	Psi	9.35	9.74	9.47	9.34	8.83	9.02	8.88	9.25	**-0.48**
A_09_P186155	FBtr0072552	38070	hipk	11.55	11.80	11.20	11.86	12.45	12.62	12.23	12.00	**0.72**
A_09_P186220	FBtr0082752	41646	Droj2	8.21	8.02	8.05	7.81	7.18	6.63	7.42	7.69	**-0.80**
A_09_P186280	FBtr0304667	7354424	CG42394	11.27	11.06	11.08	11.23	11.83	11.68	11.86	11.96	**0.67**
A_09_P186320	FBtr0301763	31792	Nrg	5.86	5.23	4.98	4.63	6.34	6.46	6.42	6.32	**1.21**
A_09_P186340	FBtr0300523	34665	bun	12.89	12.87	12.78	13.03	14.44	14.47	14.12	13.62	**1.27**
A_09_P186380	FBtr0078748	40680	exba	13.06	13.13	13.15	12.84	12.30	11.87	12.48	12.87	**-0.67**
A_09_P186565	FBtr0072218	49297	Ca-P60A	10.06	10.80	10.15	10.38	8.91	7.58	8.65	9.07	**-1.79**
A_09_P186735	FBtr0078272	40301	CG3634	9.57	9.78	9.67	9.85	9.20	9.11	9.32	9.44	**-0.45**
A_09_P186900	FBtr0077000	38721	Trn	11.05	11.09	10.93	11.00	10.47	10.46	10.74	10.92	**-0.37**

A_09_P186945	FBtr0072090	37760	CG11300	12.32	13.16	13.06	13.14	7.82	9.51	11.22	11.93	-2.80
A_09_P187150	FBtr0306386	34557	Ca-beta	8.22	8.12	7.75	7.90	7.31	7.36	7.62	7.69	-0.50
A_09_P187275	FBtr0331716	32340	rdgB	6.63	6.72	6.27	5.74	7.42	7.74	7.17	7.29	1.07
A_09_P187465	FBtr0070959	31611	CG14441	4.77	4.85	5.11	3.78	6.49	7.53	6.51	6.34	2.09
A_09_P187515	FBtr0305605	32136	CG43154	9.26	9.51	8.90	9.42	10.22	10.54	10.24	9.95	0.96
A_09_P187665	FBtr0307597	5740544	futsch	9.32	9.08	9.04	9.41	9.49	10.03	9.86	9.76	0.57
A_09_P187670	FBtr0299551	38788	Dscam2	7.13	6.86	6.41	6.69	7.14	7.51	7.55	7.59	0.67
A_09_P187775	FBtr0334758	33369	Der-1	9.72	9.75	9.62	9.50	8.41	8.08	9.03	9.45	-0.90
A_09_P187830	FBtr0088936	59173	CG18812	6.62	5.79	5.94	5.97	8.32	8.14	7.43	7.07	1.66
A_09_P187840	FBtr0301784	5740185	Ucrh	14.25	14.08	13.98	14.12	13.20	13.06	13.48	13.67	-0.75
A_09_P187865	FBtr0343858	35265	fbp	11.71	11.91	11.92	11.89	11.41	11.55	11.51	11.62	-0.34
A_09_P187930	FBtr0301324	31591	CG34417	9.56	10.29	9.96	9.85	9.00	8.64	8.78	9.05	-1.05
A_09_P188045	FBtr0307323	32158	cac	5.91	5.52	5.51	5.19	6.24	6.40	6.32	6.55	0.85
A_09_P188085	FBtr0080905	35007	Mhc	15.59	15.84	15.82	15.87	15.46	15.43	15.45	15.61	-0.29
A_09_P188110	FBtr0335005	43531	CG15514	8.88	9.21	9.10	8.93	8.60	8.56	8.50	8.56	-0.47
A_09_P188125	FBtr0078533	40495	SPoCk	7.82	7.68	8.01	7.65	6.94	7.23	7.17	7.28	-0.64
A_09_P188245	NR_073882	14462595	CR43650	7.16	7.45	7.36	6.69	5.48	5.75	5.78	6.65	-1.25
A_09_P188260	FBtr0329944	35004	CG17912	8.30	8.23	8.51	8.47	7.99	7.81	8.08	7.92	-0.43
A_09_P188300	FBtr0309096	42894	CG6364	10.27	10.44	10.37	10.44	10.06	9.99	10.02	9.99	-0.37
A_09_P188305	FBtr0089094	43811	myo	6.18	6.41	6.26	5.72	5.40	5.21	4.70	5.44	-0.95
A_09_P188425	FBtr0070707	31405	ctp	8.67	8.97	8.61	8.77	9.10	9.55	9.13	9.32	0.52
A_09_P188450	FBtr0331921	36862	gprs	6.96	6.29	6.43	6.19	7.04	7.40	7.39	7.45	0.85
A_09_P188460	FBtr0331841	39284	chrb	9.55	9.69	9.14	9.52	10.58	10.73	10.36	10.17	0.98
A_09_P188465	FBtr0344327	42237	CG31224	8.35	8.49	8.50	8.33	7.90	8.20	8.12	7.98	-0.37
A_09_P188495	FBtr0304798	35955	Drep-2	7.77	7.67	7.08	7.34	8.17	8.61	8.44	8.48	0.96
A_09_P188600	FBtr0331237	34070	Rack1	7.62	7.30	7.26	6.96	7.81	7.91	7.74	7.98	0.58
A_09_P188780	FBtr0078484	40450	CG7130	8.17	9.19	8.92	9.12	7.45	7.57	7.61	7.36	-1.35
A_09_P188830	NR_047791	12797959	CR43497	6.42	5.81	6.14	5.52	7.21	7.72	6.98	7.24	1.32
A_09_P188835	FBtr0113004	32888	CG7378	9.28	9.63	9.43	9.61	8.53	8.36	8.28	7.94	-1.21
A_09_P188895	FBtr0306087	42964	nct	8.35	8.36	8.28	8.58	7.98	7.99	8.06	7.94	-0.40
A_09_P188985	FBtr0303908	45248	Nckx30C	6.89	6.75	6.66	5.92	7.31	7.61	7.12	7.81	0.91

A_09_P189105	FBtr0088554	35963	CG8801	7.67	7.65	7.45	7.06	8.49	7.92	8.25	8.12	**0.74**
A_09_P189150	FBtr0076504	39091	Nf-YA	9.05	9.35	9.20	9.20	8.27	8.57	8.71	8.66	**-0.65**
A_09_P189205	FBtr0304871	43775	Crk	11.57	11.78	11.50	11.64	11.18	10.99	11.10	11.25	**-0.49**
A_09_P189290	FBtr0331858	39049	bol	5.34	4.87	5.13	4.31	6.44	6.94	6.21	6.60	**1.63**
A_09_P189326	NR_004028	5740447	CR40597	14.54	14.55	14.51	14.12	14.08	13.21	13.41	13.68	**-0.83**
A_09_P189330	FBtr0343160	33923	CG11319	8.22	7.58	7.80	7.65	8.54	8.41	8.69	8.53	**0.73**
A_09_P189385	FBtr0333474	45775	mei-P26	6.18	6.02	5.44	5.73	6.69	7.16	7.06	6.88	**1.10**
A_09_P189395	FBtr0307598	5740544	futsch	8.94	8.98	9.28	8.79	10.02	10.23	9.52	9.74	**0.88**
A_09_P189475	FBtr0085995	35554	Pld	9.40	9.29	9.29	9.27	10.21	9.96	9.87	9.61	**0.60**
A_09_P189485	FBtr0071079	31678	CHES-1-like	9.07	9.01	8.96	8.71	10.90	10.91	10.14	9.69	**1.47**
A_09_P189565	FBtr0305584	35336	Fs(2)Ket	10.01	10.23	10.10	9.96	9.48	9.67	9.38	9.70	**-0.51**
A_09_P189740	FBtr0083312	41995	Pak3	12.69	12.80	12.76	12.80	14.01	13.92	13.50	13.33	**0.93**
A_09_P189750	FBtr0310669	36560	Rcd1	11.51	11.72	11.62	11.72	11.94	11.89	11.84	11.83	**0.23**
A_09_P189955	FBtr0100351	35260	CG10462	10.13	10.34	10.28	10.44	10.66	10.73	10.57	10.50	**0.32**
A_09_P189960	FBtr0333193	41688	CG9297	14.22	14.80	14.64	14.82	13.79	13.68	13.88	14.24	**-0.73**
A_09_P190035	FBtr0331602	31456	SK	9.73	9.67	9.56	9.50	10.23	10.59	10.07	10.19	**0.65**
A_09_P190045	FBtr0070345	31166	Actn	14.14	14.55	14.81	14.58	13.29	13.13	13.38	13.47	**-1.20**
A_09_P190060	FBtr0329846	35277	sick	7.99	7.92	7.97	7.67	9.27	9.37	9.27	9.06	**1.35**
A_09_P190075	FBtr0076353	50296	dpr6	6.45	6.61	5.81	6.39	6.91	7.42	6.90	7.07	**0.76**
A_09_P190116	FBtr0089179	43766	plexB	9.64	9.83	9.58	9.95	9.17	9.22	9.27	9.55	**-0.44**
A_09_P190140	FBtr0300123	42356	bnl	8.47	8.11	8.46	8.11	9.59	9.18	8.85	8.88	**0.83**
A_09_P190285	NR_002478	3771817	pncr008:3L	7.80	7.88	7.81	7.80	6.27	7.35	6.64	6.67	**-1.09**
A_09_P190315	FBtr0310005	39919	Rbp6	8.91	9.00	8.63	8.76	9.68	9.86	9.57	9.82	**0.91**
A_09_P190340	FBtr0332873	38389	Hsp83	11.02	11.43	11.09	10.98	10.25	9.41	10.27	10.61	**-1.00**
A_09_P190400	FBtr0100402	3885567	Dyrk3	9.67	10.16	9.93	10.15	9.52	9.57	9.44	9.29	**-0.52**
A_09_P190485	NR_003113	4379815	sphinx	15.06	15.15	15.09	15.17	14.73	14.68	14.85	14.98	**-0.31**
A_09_P190510	FBtr0112904	43839	eIF4G	10.80	10.82	10.90	10.60	10.13	9.71	9.96	10.07	**-0.82**
A_09_P190525	FBtr0082655	41569	Past1	13.91	13.73	13.74	13.86	13.60	13.54	13.61	13.46	**-0.26**
A_09_P190545	FBtr0072552	38070	hipk	5.20	4.39	4.81	4.44	6.14	6.90	5.99	5.91	**1.52**
A_09_P190560	FBtr0075864	39501	CG10171	10.47	10.78	10.66	10.82	9.33	9.25	9.77	10.13	**-1.06**
A_09_P190590	FBtr0083205	41938	Sap47	11.27	11.11	11.01	11.21	11.68	11.68	11.78	11.99	**0.63**

A_09_P190620	FBtr0085302	43385	Hrb98DE	12.28	12.38	12.12	12.31	11.95	11.81	12.02	12.04	**-0.32**
A_09_P190635	FBtr0089141	43782	Nfl	7.87	7.87	7.97	7.76	5.27	5.99	6.57	6.88	**-1.69**
A_09_P190695	FBtr0335518	40720	CG10979	7.22	7.26	7.07	6.99	6.69	6.68	6.89	6.78	**-0.38**
A_09_P190785	FBtr0301417	33277	IA-2	10.49	9.71	10.05	10.18	10.98	11.00	11.32	11.11	**1.00**
A_09_P190795	FBtr0335397	32353	mamo	6.35	7.17	6.63	6.57	5.00	6.11	4.95	5.09	**-1.39**
A_09_P191145	FBtr0301512	4379870	CG34141	8.21	8.09	7.86	7.77	7.38	6.72	7.20	7.46	**-0.79**
A_09_P191230	FBtr0089490	39063	PGRP-LC	6.09	5.70	5.63	5.49	6.52	6.84	6.34	6.40	**0.80**
A_09_P191295	FBtr0070411	31215	CG2918	7.80	7.99	7.72	7.16	6.10	5.68	6.42	7.29	**-1.30**
A_09_P191485	FBtr0306578	38196	n-syb	8.81	8.19	8.59	8.17	8.96	8.92	9.07	9.31	**0.62**
A_09_P191530	FBtr0075773	39557	Hsc70Cb	8.90	8.87	8.94	8.56	7.72	7.28	7.92	8.26	**-1.02**
A_09_P191550	FBtr0340428	32437	Lsd-2	7.74	8.27	8.15	7.96	7.17	7.39	7.09	7.33	**-0.78**
A_09_P191735	FBtr0299961	7354407	CG42362	8.46	8.28	8.28	8.15	10.46	10.34	9.90	9.42	**1.74**
A_09_P191745	FBtr0079937	34338	Pen	7.26	8.16	7.52	7.82	6.59	5.82	6.32	6.50	**-1.39**
A_09_P192085	FBtr0331368	326116	tau	6.75	7.68	7.56	7.56	5.75	5.01	5.79	6.04	**-1.74**
A_09_P192095	FBtr0079485	34058	CG7191	6.76	5.80	6.57	5.73	8.99	8.01	8.32	7.61	**2.02**
A_09_P192230	FBtr0336454	7354470	CG42402	5.96	5.68	5.52	4.86	6.33	6.96	6.43	6.43	**1.03**
A_09_P192280	FBtr0289981	34709	Sfmbt	8.88	9.00	8.92	9.10	8.33	8.34	8.36	8.58	**-0.57**
A_09_P192300	FBtr0331692	40924	CG2993	8.05	7.87	7.99	7.78	8.83	8.94	8.67	8.89	**0.91**
A_09_P192395	FBtr0331423	38589	Rh50	9.59	10.42	10.11	10.49	9.38	9.32	8.98	8.84	**-1.02**
A_09_P192470	FBtr0085456	43498	CG15506	10.69	8.77	9.78	9.00	11.21	11.55	11.25	11.09	**1.72**
A_09_P192480	NR_073904	12798545	CR43334	9.89	9.69	9.84	9.63	10.77	11.59	11.06	11.12	**1.38**
A_09_P192710	FBtr0083677	42258	CG18208	6.89	6.48	6.05	6.23	7.11	7.18	6.94	7.15	**0.68**
A_09_P193015	FBtr0089075	43799	Ephrin	7.97	8.31	8.34	8.26	7.59	7.49	7.35	7.38	**-0.77**
A_09_P193155	FBtr0070379	45278	csw	6.15	5.70	5.44	5.48	6.17	6.70	6.54	6.63	**0.82**
A_09_P193295	FBtr0100255	37786	TM4SF	8.37	7.88	8.23	7.91	8.99	8.84	8.62	8.90	**0.74**
A_09_P193360	FBtr0079602	34144	CG14275	11.07	10.41	10.58	10.50	11.22	11.22	11.52	11.70	**0.77**
A_09_P193426	NR_002020	3772597	mma-element:(	8.05	8.26	7.61	7.76	9.17	8.80	9.21	9.43	**1.23**
A_09_P193475	FBtr0071828	37562	qkr58E-2	7.54	7.80	7.43	7.39	7.12	6.83	6.80	7.10	**-0.58**
A_09_P193500	FBtr0070083	30998	svr	9.61	9.75	9.83	9.93	9.00	9.23	9.29	8.96	**-0.66**
A_09_P193515	FBtr0334334	42922	Pp1alpha-96A	7.47	7.21	6.89	7.11	7.80	8.00	8.02	7.75	**0.72**
A_09_P193560	FBtr0336717	42150	Rim	7.80	7.52	7.45	7.45	8.73	8.83	8.59	8.53	**1.12**

A_09_P193615	FBtr0303486	41817	CG42788	9.06	8.35	8.46	8.35	9.47	9.30	9.27	8.98	0.70
A_09_P193665	FBtr0305197	41094	pum	11.21	11.17	10.86	11.26	11.63	12.06	11.78	11.88	0.71
A_09_P193670	FBtr0082103	41166	Crc	15.30	15.25	15.30	15.08	14.26	13.93	14.66	15.04	-0.76
A_09_P193685	FBtr0100663	31521	Act5C	15.90	15.92	15.96	15.82	16.09	16.09	16.04	16.00	0.16
A_09_P193705	FBtr0330276	45840	cpo	10.23	10.18	10.20	9.92	11.14	11.20	10.81	10.94	0.89
A_09_P193725	FBtr0331790	38714	CG10289	7.58	7.85	7.24	7.51	8.58	8.80	8.21	7.99	0.85
A_09_P193935	FBtr0339892	38102	CG12502	5.18	3.76	5.03	3.96	6.00	6.48	6.11	6.27	1.73
A_09_P194045	FBtr0074750	32977	Ubqn	7.79	8.24	7.94	8.02	7.04	7.38	7.19	7.54	-0.71
A_09_P194100	FBtr0344217	43362	CG34362	6.85	6.56	6.67	6.00	7.40	7.85	7.58	7.47	1.06
A_09_P194140	FBtr0306140	12798465	CG43194	5.08	4.68	5.11	4.93	6.57	6.46	6.93	5.75	1.48
A_09_P194235	FBtr0074635	32871	CG7053	9.75	9.40	9.64	9.38	10.75	10.63	10.48	9.86	0.89
A_09_P194445	FBtr0303120	38863	Ank2	8.35	8.79	8.65	8.59	8.10	7.35	7.95	8.17	-0.71
A_09_P194620	FBtr0332394	32923	CG8028	8.76	8.84	9.37	8.87	7.52	8.16	8.27	8.29	-0.90
A_09_P194760	FBtr0332888	32324	CG15760	5.00	4.69	4.45	4.30	5.77	5.96	5.46	5.69	1.11
A_09_P194785	FBtr0088458	36020	CG1516	8.85	9.45	8.84	8.83	7.84	6.84	7.89	8.33	-1.27
A_09_P194860	FBtr0330706	34038	Slob	5.99	4.90	5.85	5.44	6.55	6.70	6.70	6.20	0.99
A_09_P194900	FBtr0087625	36521	fas	9.24	9.52	9.47	9.40	9.99	10.18	10.04	9.79	0.59
A_09_P194935	FBtr0084786	43005	OstStt3	11.98	12.20	11.80	11.82	10.80	10.41	10.93	11.65	-1.00
A_09_P194960	FBtr0332412	33725	CG3887	8.33	8.12	8.19	7.93	7.68	7.27	7.64	7.75	-0.56
A_09_P194995	FBtr0081519	35408	nrv3	11.13	10.87	11.00	10.70	11.58	11.12	11.42	11.46	0.47
A_09_P195050	FBtr0334984	31550	Ca-alpha1T	5.11	3.89	4.12	4.09	5.54	5.70	5.84	5.83	1.43
A_09_P195065	FBtr0300190	39611	bmm	7.18	6.67	6.61	6.57	8.69	7.82	8.01	7.83	1.33
A_09_P195125	FBtr0309280	39052	nwk	5.80	5.66	5.50	4.49	6.66	6.32	6.19	6.24	0.99
A_09_P195210	FBtr0082717	41603	CG12360	10.33	10.70	10.70	10.85	10.17	9.73	9.87	10.09	-0.68
A_09_P195235	FBtr0076187	39265	Plod	9.61	9.27	9.33	8.65	7.42	7.42	8.04	8.46	-1.38
A_09_P195260	FBtr0079937	34338	Pen	9.21	10.02	9.60	10.10	8.66	8.18	8.42	8.36	-1.33
A_09_P195775	FBtr0070951	31601	Ctr1A	9.42	9.39	9.31	9.38	8.97	9.12	8.97	9.05	-0.35
A_09_P195835	FBtr0084036	42496	RhoGAP93B	8.06	7.93	7.96	7.76	7.56	7.32	7.61	7.63	-0.40
A_09_P195885	FBtr0082854	41666	sqd	10.89	10.71	10.68	10.69	10.42	10.02	10.23	10.31	-0.50
A_09_P196120	FBtr0073145	38424	sty	9.75	9.89	9.75	9.52	11.52	11.38	10.72	10.41	1.28
A_09_P196165	FBtr0330345	39527	bru-3	9.37	9.15	8.48	9.25	10.29	10.49	10.26	10.04	1.21

A_09_P196190	FBtr0309042	44013	sls	10.72	11.00	10.74	10.88	10.32	10.34	10.39	10.38	**-0.48**
A_09_P196330	FBtr0305116	12798282	CG43124	11.17	11.92	11.57	12.02	9.35	9.68	10.01	10.43	**-1.80**
A_09_P196460	FBtr0072143	37805	G-salpha60A	12.74	13.03	12.98	12.99	12.30	12.23	12.49	12.45	**-0.57**
A_09_P196550	FBtr0083146	41889	Rh6	9.77	9.57	9.70	9.52	9.85	10.11	10.11	10.04	**0.39**
A_09_P196650	FBtr0091709	3771920	PGRP-LD	11.15	11.52	11.35	11.54	10.53	10.65	10.36	10.51	**-0.88**
A_09_P196695	NM_001202086	10178854	CG42719	6.85	5.74	6.26	5.36	7.07	7.21	7.30	7.44	**1.20**
A_09_P196710	FBtr0340310	32561	mmd	7.79	7.22	6.86	7.38	7.97	8.62	8.46	8.28	**1.02**
A_09_P196755	FBtr0333228	33824	Gpdh	13.36	13.21	13.14	13.04	12.53	11.94	12.40	12.58	**-0.83**
A_09_P196850	FBtr0306201	3346162	mun	5.91	5.20	4.95	4.68	6.74	7.14	6.78	7.08	**1.75**
A_09_P196905	FBtr0072814	38197	metl	10.86	10.60	10.83	10.59	10.36	10.42	10.46	10.38	**-0.32**
A_09_P196955	FBtr0300491	3346160	dpr4	7.23	6.67	6.67	6.61	7.80	8.14	7.89	7.91	**1.14**
A_09_P197040	FBtr0070905	31577	CG3847	11.99	12.03	12.10	12.06	11.80	11.92	11.83	11.84	**-0.20**
A_09_P197045	FBtr0331603	31456	SK	9.71	9.61	9.47	9.49	10.26	10.58	10.00	10.16	**0.68**
A_09_P197055	FBtr0113232	41711	CCHa1	8.22	7.92	7.88	7.83	9.12	9.41	8.80	8.64	**1.03**
A_09_P197065	FBtr0331983	37953	CG33988	5.93	5.48	5.39	4.88	6.54	7.27	6.26	7.24	**1.41**
A_09_P197075	FBtr0089049	35646	Gadd45	9.22	8.35	9.29	8.56	12.08	11.83	11.32	10.80	**2.65**
A_09_P197095	FBtr0306908	246515	CG43325	13.54	13.54	13.79	13.70	13.20	13.03	13.09	13.08	**-0.54**
A_09_P197105	FBtr0307387	40342	CG10508	7.56	7.85	7.53	7.83	8.33	8.64	8.36	8.01	**0.64**
A_09_P197250	FBtr0113436	40273	CG32425	13.15	12.60	12.70	12.61	11.82	12.05	12.22	12.32	**-0.66**
A_09_P197435	FBtr0081538	40904	alphaTub84D	14.35	14.27	14.37	14.39	14.03	14.11	14.18	14.27	**-0.19**
A_09_P197530	FBtr0305094	39198	A2bp1	10.90	11.32	11.00	10.92	12.09	12.28	11.72	11.82	**0.94**
A_09_P197545	FBtr0112608	42638	CG34376	8.73	9.03	8.98	9.17	9.91	9.74	9.44	9.30	**0.62**
A_09_P197930	FBtr0074881	40160	Kap-alpha1	7.72	7.88	7.80	7.32	7.23	6.85	7.06	7.17	**-0.61**
A_09_P198015	FBtr0081957	41067	skap	10.11	10.53	10.52	10.35	9.99	8.84	9.58	9.55	**-0.88**
A_09_P198160	FBtr0074749	32974	Zw	7.94	8.09	7.56	7.65	5.05	5.56	6.45	7.05	**-1.78**
A_09_P198175	FBtr0078693	117332	Rheb	10.98	11.22	11.02	11.24	12.19	11.38	11.90	11.95	**0.74**
A_09_P198290	FBtr0339588	31104	mei-38	7.32	8.27	7.88	8.11	7.34	6.89	6.89	6.88	**-0.90**
A_09_P198340	FBtr0340145	30996	CG17778	6.53	6.22	6.59	5.92	7.11	7.60	7.08	7.07	**0.90**
A_09_P198355	FBtr0070644	251900	GlcAT-I	8.13	8.27	8.26	8.34	7.49	7.64	7.78	8.12	**-0.49**
A_09_P198375	FBtr0078492	40457	Ddx1	11.11	11.24	11.03	11.43	10.35	10.05	10.50	10.65	**-0.82**
A_09_P198385	FBtr0070501	31255	Csat	8.77	9.05	8.63	8.93	7.59	7.41	7.86	8.18	**-1.09**

A_09_P198390	FBtr0273279	31257	CG2680	11.05	11.01	11.12	10.91	9.92	10.12	10.43	10.69	-0.73
A_09_P198415	FBtr0077782	33434	Slh	12.09	12.17	12.25	12.24	11.25	11.36	11.75	11.95	-0.61
A_09_P198480	FBtr0089998	36018	Prosalpha7	13.70	13.77	13.78	13.81	12.89	12.70	13.24	13.49	-0.68
A_09_P198490	FBtr0071848	37551	T3dh	9.98	10.55	10.44	10.36	9.75	9.35	9.33	9.21	-0.92
A_09_P198585	FBtr0081253	35246	ref(2)P	11.94	12.40	12.15	12.44	13.80	13.65	13.26	12.75	1.13
A_09_P198655	FBtr0085384	43447	Pglym78	14.08	14.35	14.56	14.55	12.75	12.43	13.53	13.87	-1.24
A_09_P198660	FBtr0088420	36060	Pfk	10.88	11.65	11.23	11.54	8.89	8.58	9.63	10.32	-1.97
A_09_P198685	FBtr0089965	41852	Tm1	16.75	16.76	16.82	16.82	16.30	15.95	16.34	16.34	-0.56
A_09_P198700	FBtr0071897	37628	RpL23	16.62	16.59	16.59	16.60	16.71	16.71	16.69	16.67	0.09
A_09_P198735	FBtr0100594	3771877	Adh	16.21	15.98	16.28	15.91	14.37	14.33	15.10	15.41	-1.29
A_09_P199195	FBtr0307056	33308	CG5001	10.34	10.83	10.71	10.70	10.07	9.52	9.70	9.63	-0.92
A_09_P199200	FBtr0304910	33690	pog	6.82	6.61	6.45	5.99	7.52	7.66	7.34	7.57	1.05
A_09_P199285	FBtr0078967	40530	abs	9.61	9.61	9.58	9.79	9.33	9.20	9.23	9.19	-0.41
A_09_P199300	FBtr0089338	41087	Rel	10.60	10.35	10.63	10.98	13.58	13.50	12.96	12.15	2.41
A_09_P199330	FBtr0076526	39048	Tequila	13.11	13.42	13.36	13.29	13.68	13.63	13.72	13.58	0.36
A_09_P199395	FBtr0070108	30970	CG3038	9.62	10.08	9.98	9.98	9.09	9.45	9.39	9.62	-0.53
A_09_P199445	FBtr0080565	2768914	CG33307	13.95	14.23	14.07	14.19	12.98	13.14	13.36	13.64	-0.83
A_09_P199480	FBtr0006151	3772432	Adhr	10.01	10.57	10.21	10.37	9.77	9.61	9.58	9.73	-0.62
A_09_P199525	FBtr0080763	34930	Ku80	8.00	7.74	7.90	7.66	8.79	8.95	8.96	9.18	1.15
A_09_P199570	FBtr0332674	44013	sls	10.80	11.60	11.31	11.40	10.40	10.40	10.30	10.49	-0.88
A_09_P199616	FBtr0086983	36932	Amy-d	4.30	5.10	3.26	3.30	6.61	6.21	6.29	5.13	2.07
A_09_P199620	FBtr0088663	35910	proPO45	9.08	9.79	9.94	9.98	8.19	7.79	8.35	8.50	-1.49
A_09_P199715	FBtr0112691	2768848	mute	8.32	8.54	8.69	8.75	8.21	8.11	7.93	7.90	-0.54
A_09_P199725	FBtr0330272	45840	cpo	8.05	8.07	8.11	7.89	8.70	8.90	8.54	8.96	0.74
A_09_P199775	FBtr0089139	43783	Syt7	7.16	6.72	6.77	6.23	7.27	7.54	7.41	7.62	0.74
A_09_P199895	FBtr0113015	33403	CG15387	8.61	9.04	8.78	9.14	8.17	8.26	8.19	8.49	-0.61
A_09_P199905	FBtr0070598	31335	HIP-R	10.97	11.36	11.30	11.22	10.50	10.48	10.22	10.36	-0.82
A_09_P199915	FBtr0087871	36400	CG8768	10.44	10.25	10.28	10.35	10.12	9.98	10.15	10.17	-0.22
A_09_P199960	FBtr0078950	40556	CG9855	8.72	8.98	8.98	8.89	8.47	7.99	8.39	8.62	-0.53
A_09_P201115	FBtr0073807	32319	Tango2	6.88	7.27	6.70	6.51	5.28	4.98	5.97	6.35	-1.19
A_09_P201260	FBtr0071039	31638	CG32732	8.64	8.57	8.81	8.24	7.88	7.73	7.61	7.88	-0.79

A_09_P201390	FBtr0273217	42336	CG17751	10.91	12.08	11.85	12.20	3.69	6.13	8.70	9.53	**-4.75**
A_09_P201395	FBtr0084830	318734	CG31436	7.48	7.29	7.03	6.88	8.20	7.94	8.02	8.08	**0.89**
A_09_P201635	FBtr0078573	40506	Arf79F	11.51	11.60	11.92	11.46	11.19	10.71	11.02	11.32	**-0.56**
A_09_P201695	FBtr0332935	31513	CG15765	7.23	7.12	7.20	7.17	8.18	9.03	8.36	8.01	**1.22**
A_09_P201995	FBtr0083062	41844	Set	12.20	12.53	12.55	12.37	11.34	11.12	11.50	11.74	**-0.99**
A_09_P202005	FBtr0331187	33928	CG31635	9.57	9.82	9.72	9.69	10.47	10.72	10.24	9.98	**0.65**
A_09_P202010	FBtr0343539	43758	RhoGAP100F	5.20	4.87	5.10	4.75	5.74	6.46	5.85	6.12	**1.06**
A_09_P202065	FBtr0339612	43182	CG12290	9.23	8.73	8.90	8.72	10.66	10.75	10.10	9.53	**1.36**
A_09_P202245	FBtr0087172	36829	CG7997	8.65	9.09	8.64	8.57	6.84	7.26	7.42	7.74	**-1.43**
A_09_P202280	FBtr0070598	31335	HIP-R	11.70	12.07	12.04	11.98	11.29	11.22	11.03	11.20	**-0.77**
A_09_P202450	M61765	33501	gammaTub23C	9.04	9.19	9.23	9.41	8.79	8.54	8.61	8.77	**-0.54**
A_09_P202455	Z86118	41889	Rh6	9.84	9.64	9.72	9.73	9.86	10.32	10.19	10.26	**0.43**
A_09_P202490	FBtr0079852	34284	Pka-C1	11.51	11.81	11.71	11.79	11.22	11.25	10.99	11.29	**-0.52**
A_09_P202600	FBtr0310005	39919	Rbp6	9.35	9.71	9.46	9.35	10.35	10.89	10.26	10.37	**1.00**
A_09_P202610	FBtr0070707	31405	ctp	9.45	9.32	9.20	9.50	9.58	9.62	9.72	9.71	**0.29**
A_09_P202710	FBtr0290238	33498	Rbp9	7.81	8.54	8.55	8.49	7.80	7.30	7.55	7.79	**-0.74**
A_09_P202800	FBtr0302942	35347	CG9331	9.56	9.67	9.66	9.47	8.75	8.24	9.00	9.36	**-0.75**
A_09_P202890	FBtr0073300	38545	nAcRbeta-64B	6.18	5.88	5.66	5.04	6.47	7.06	6.54	6.79	**1.03**
A_09_P202905	FBtr0084047	50050	CG17278	10.22	9.51	9.74	9.61	11.01	10.57	10.58	10.42	**0.88**
A_09_P202970	FBtr0332668	38246	CG12025	11.09	11.38	11.21	11.39	10.95	10.97	10.93	10.86	**-0.34**
A_09_P203010	FBtr0076001	39390	CAH2	10.33	9.41	9.71	9.55	12.37	12.53	11.95	10.76	**2.15**
A_09_P203015	FBtr0080562	34804	Smg5	8.51	8.91	8.47	8.81	9.47	9.51	9.37	8.85	**0.62**
A_09_P203075	FBtr0310161	38160	Ptp61F	8.66	8.95	8.55	8.99	9.54	9.38	9.43	9.17	**0.59**
A_09_P203080	FBtr0076734	44496	Uba2	7.45	7.78	7.41	7.33	6.69	6.39	6.85	7.12	**-0.73**
A_09_P203085	FBtr0076265	39213	CG11811	10.03	9.95	10.27	9.63	9.31	9.08	9.04	8.83	**-0.90**
A_09_P203140	FBtr0310560	44380	fok	8.18	8.65	8.80	8.84	7.25	7.42	7.59	7.66	**-1.14**
A_09_P203155	FBtr0301417	33277	IA-2	5.69	5.18	5.24	4.85	6.89	7.14	6.93	6.71	**1.67**
A_09_P203175	FBtr0076027	39377	yps	9.05	8.84	9.41	8.74	7.49	6.38	7.03	7.23	**-1.98**
A_09_P203330	FBtr0336474	42742	Ir	6.37	6.97	6.84	5.59	7.99	8.34	7.32	7.90	**1.45**
A_09_P203360	FBtr0329892	33944	homer	9.81	10.13	9.83	10.05	9.62	9.24	9.47	9.54	**-0.49**
A_09_P203365	FBtr0299881	3355150	AGO3	7.56	7.05	7.24	7.28	8.64	8.14	8.05	7.80	**0.87**

A_09_P203390	FBtr0304964	36329	Cam	11.19	11.01	10.91	11.07	11.99	12.08	11.98	11.85	0.93
A_09_P203400	FBtr0083231	41955	Mhcl	10.53	11.07	11.05	11.05	10.51	10.36	10.20	10.09	-0.64
A_09_P203445	FBtr0310320	37086	CG14499	11.05	10.99	10.92	11.44	13.65	12.96	13.46	13.42	2.27
A_09_P203710	FBtr0339908	39270	scyl	11.05	11.14	10.68	10.93	12.00	12.18	11.86	11.64	0.97
A_09_P203855	FBtr0333137	38468	CG12605	7.52	7.59	7.30	7.30	7.86	8.21	8.06	8.01	0.61
A_09_P204010	FBtr0079042	33721	Rtnl1	11.76	12.26	11.86	11.84	9.67	10.12	10.65	10.90	-1.60
A_09_P204030	FBtr0088996	45398	Aldh-III	10.44	10.70	10.44	10.50	9.55	9.16	9.80	10.12	-0.86
A_09_P204055	NR_048037	12798533	CR43241	9.06	9.18	8.91	9.16	9.55	9.47	9.40	9.31	0.35
A_09_P204060	FBtr0086637	37168	MFS15	7.08	7.59	7.31	7.05	6.72	6.59	6.48	6.31	-0.73
A_09_P204215	FBtr0100239	32797	Frq1	6.60	6.23	5.63	5.75	7.04	6.94	7.04	7.03	0.96
A_09_P204330	FBtr0073499	32090	HP5	9.17	9.08	9.13	9.19	8.94	8.91	8.89	8.86	-0.24
A_09_P204570	FBtr0333602	31826	rdgA	7.35	7.37	6.66	7.36	8.05	8.35	7.98	7.73	0.84
A_09_P204620	FBtr0304675	39165	CalpB	8.88	8.71	8.84	8.64	10.36	10.42	10.06	9.83	1.40
A_09_P204720	FBtr0303902	192507	CG4747	12.48	12.55	12.54	12.66	12.31	12.28	12.37	12.39	-0.22
A_09_P204730	FBtr0085307	43390	CG9989	6.88	6.57	5.69	5.26	7.71	7.63	7.89	7.97	1.70
A_09_P204735	FBtr0084988	318682	CG31323	8.40	7.63	7.45	7.22	9.85	9.74	9.13	8.58	1.65
A_09_P204885	FBtr0333836	39054	Rdl	8.11	7.57	7.70	7.45	9.52	9.60	9.60	9.48	1.84
A_09_P204905	FBtr0309042	44013	sls	9.79	10.14	9.94	10.09	9.46	9.58	9.43	9.48	-0.50
A_09_P204945	FBtr0083993	42463	CG17271	11.70	12.26	12.02	12.25	10.14	10.12	11.04	11.53	-1.35
A_09_P205005	FBtr0340428	32437	Lsd-2	7.78	8.58	8.13	8.04	7.43	7.54	7.13	7.37	-0.77
A_09_P205015	FBtr0329846	35277	sick	9.81	9.79	9.72	9.72	10.97	11.18	11.02	11.09	1.30
A_09_P205050	FBtr0087437	36636	AttA	10.99	12.12	10.75	11.77	15.90	15.21	15.58	15.35	4.11
A_09_P205180	FBtr0302692	44258	ps	9.99	10.20	9.89	9.88	10.68	10.82	10.26	10.41	0.55
A_09_P205240	NR_048371	12797884	CR43283	12.46	12.32	12.25	11.98	13.53	13.52	13.00	12.93	1.00
A_09_P205250	FBtr0301446	38698	loj	11.00	10.97	10.94	10.61	9.60	9.40	10.20	10.59	-0.93
A_09_P205315	FBtr0339321	5740590	CG34353	6.98	6.77	6.43	6.30	7.19	7.15	7.20	7.24	0.57
A_09_P205360	FBtr0332961	32017	CG2186	8.52	8.44	8.32	8.48	7.89	8.06	7.96	8.00	-0.46
A_09_P205465	FBtr0087004	47764	Amy-p	8.25	9.77	7.11	7.19	11.48	10.66	10.57	9.99	2.60
A_09_P205515	FBtr0089091	43810	CG11360	6.93	6.91	6.86	6.69	5.79	5.67	6.51	6.46	-0.74
A_09_P205565	FBtr0302145	41824	Mf	8.54	9.76	9.13	9.83	8.17	7.65	7.76	7.77	-1.48
A_09_P205660	FBtr0334145	42215	CG44009	10.55	10.76	10.65	10.74	10.47	10.44	10.36	10.22	-0.30

A_09_P205700	FBtr0077492	33602	bowl	9.15	9.50	9.28	9.23	9.80	9.98	9.68	9.50	0.45
A_09_P205785	FBtr0077844	33397	Uch	11.89	12.28	11.92	12.14	11.27	11.07	11.44	11.64	-0.71
A_09_P205840	FBtr0302233	8673970	CG40472	13.54	13.34	13.29	13.53	12.52	12.47	12.87	13.08	-0.69
A_09_P205845	FBtr0077683	33495	NTPase	12.42	12.23	12.34	12.40	11.94	11.89	12.03	12.05	-0.37
A_09_P205865	FBtr0084219	42626	CG7059	3.08	4.34	3.65	4.11	5.92	6.01	5.59	4.80	1.78
A_09_P205885	FBtr0332962	31217	CG2865	8.58	7.46	7.96	7.42	9.67	9.71	9.22	8.97	1.54
A_09_P205945	FBtr0076093	39284	chrb	7.49	7.78	7.50	7.00	8.52	8.67	8.30	8.35	1.02
A_09_P205970	FBtr0331515	39018	orb2	6.05	4.90	5.19	4.51	6.40	6.81	6.64	6.68	1.47
A_09_P206005	FBtr0308309	32468	cngl	4.99	4.28	4.64	4.07	5.30	6.25	5.75	6.15	1.37
A_09_P206105	FBtr0310006	39919	Rbp6	6.97	7.27	6.45	6.25	7.74	8.13	7.35	7.86	1.03
A_09_P206290	FBtr0072677	38154	mtacp1	10.47	10.25	10.34	10.34	9.69	9.34	9.57	9.91	-0.72
A_09_P206295	FBtr0086020	35539	Ars2	10.01	10.08	10.12	10.23	9.65	9.46	9.81	9.95	-0.39
A_09_P206370	FBtr0302575	38001	zip	6.22	6.88	6.39	6.18	7.17	7.27	6.97	7.09	0.71
A_09_P206385	FBtr0077021	38710	Acp65Aa	9.34	10.12	9.91	10.49	7.18	8.43	6.90	6.55	-2.70
A_09_P206410	FBtr0113060	35987	CG12929	9.28	9.33	9.33	9.39	9.00	9.13	9.11	8.88	-0.30
A_09_P206465	FBtr0113210	41185	Pnn	8.23	8.10	8.41	7.97	7.63	7.53	7.73	7.84	-0.49
A_09_P206530	FBtr0331841	39284	chrb	9.65	9.51	9.27	9.46	10.89	11.04	10.47	10.17	1.17
A_09_P206585	FBtr0076229	39251	Sod	10.46	10.57	10.16	10.54	9.48	9.20	9.76	10.14	-0.79
A_09_P206610	FBtr0333700	43824	Cals	9.99	9.62	9.68	9.73	8.91	8.78	9.30	9.28	-0.68
A_09_P206660	FBtr0071845	37557	babos	8.66	8.51	8.33	7.95	9.73	9.06	9.26	9.04	0.91
A_09_P206700	FBtr0083312	41995	Pak3	6.01	5.69	5.74	5.22	7.11	6.67	6.68	6.25	1.01
A_09_P206805	FBtr0331716	32340	rdgB	7.90	7.93	7.55	7.27	8.86	9.10	8.73	8.53	1.14
A_09_P206835	FBtr0091472	3346141	CG33521	12.69	13.39	13.26	13.23	11.78	11.36	11.68	12.09	-1.42
A_09_P206845	FBtr0112749	5740761	CG34445	11.05	10.99	11.21	11.21	10.97	10.59	10.49	10.17	-0.56
A_09_P206890	FBtr0336831	33690	pog	8.29	7.88	7.44	8.06	8.69	9.00	8.91	8.68	0.90
A_09_P206965	FBtr0306738	32245	fne	8.66	8.30	7.82	8.25	8.82	9.34	9.12	9.35	0.90
A_09_P207005	FBtr0302583	39004	Fhos	7.63	8.40	8.75	8.51	6.70	6.74	6.79	6.63	-1.61
A_09_P207135	FBtr0114575	2768718	qvr	9.08	9.14	8.50	8.84	6.75	7.01	7.43	7.97	-1.60
A_09_P207150	FBtr0071313	31872	Dsor1	10.15	10.65	10.19	10.48	9.89	9.78	9.88	9.95	-0.49
A_09_P207185	FBtr0330735	36475	Dh31-R1	4.77	4.36	4.45	3.93	5.88	5.62	5.55	5.56	1.27
A_09_P207235	FBtr0112962	31776	CG1440	9.49	9.66	9.29	9.22	8.27	7.67	8.73	9.14	-0.96

A_09_P207275	FBtr0303198	32457	gce	10.48	10.97	10.92	10.96	9.92	10.05	9.84	9.76	-0.94
A_09_P207295	FBtr0300019	31687	CG1402	7.29	7.33	6.94	6.92	7.55	8.01	7.56	7.88	0.63
A_09_P207430	FBtr0080062	34386	CG5385	9.21	9.50	9.25	9.35	8.42	8.14	8.88	8.93	-0.74
A_09_P207530	FBtr0071682	37468	pirk	10.92	9.79	10.39	10.18	12.88	11.91	12.11	11.74	1.84
A_09_P207600	FBtr0074445	32719	CG8664	13.93	14.92	14.17	14.85	13.11	12.08	12.58	12.44	-1.91
A_09_P207650	FBtr0073774	32292	CG1640	7.49	8.25	8.04	7.94	6.90	6.53	6.81	6.97	-1.12
A_09_P207700	FBtr0076276	39214	CG6463	13.40	13.45	13.42	13.57	13.24	13.28	13.21	13.39	-0.18
A_09_P207735	BP560022	34853	CG15282	8.65	7.89	7.14	7.94	9.69	9.80	9.30	9.20	1.59
A_09_P207745	FBtr0073853	32314	up	9.50	9.78	9.66	9.59	8.19	8.74	8.41	8.82	-1.09
A_09_P207847	FBtr0070160	31005	CG13365	12.75	12.80	12.93	12.83	12.11	12.47	12.35	12.33	-0.52
A_09_P207880	FBtr0113015	33403	CG15387	7.03	7.22	7.23	7.28	6.21	6.11	6.74	6.95	-0.69
A_09_P207940	FBtr0303751	38747	dikar	5.36	5.01	4.81	4.64	7.07	7.04	6.60	6.41	1.82
A_09_P208030	FBtr0077156	38613	Uev1A	12.91	13.12	13.20	13.15	12.68	12.46	12.71	12.76	-0.44
A_09_P208310	FBtr0309054	39054	Rdl	7.75	6.95	6.84	6.94	8.45	8.54	8.34	8.59	1.36
A_09_P208360	FBtr0303687	32619	para	8.57	7.99	7.98	7.80	9.14	9.20	9.07	9.39	1.11
A_09_P208370	FBtr0074915	40178	CG8004	10.49	10.38	10.51	10.27	9.77	9.31	9.74	9.81	-0.76
A_09_P208765	FBtr0310661	43183	Ald	11.87	12.72	12.69	12.73	10.42	10.08	10.90	11.30	-1.83
A_09_P208800	NM_133137	32913	Muc18B	13.13	13.13	13.10	13.03	14.69	14.32	14.27	14.10	1.25
A_09_P208840	FBtr0113345	59144	CG18748	8.73	8.45	8.20	8.19	10.62	10.11	9.92	9.37	1.61
A_09_P208885	FBtr0299862	32033	CG42339	7.80	7.14	7.15	7.03	7.93	8.14	7.99	8.22	0.79
A_09_P208910	FBtr0305557	34829	CG17341	7.14	7.42	7.06	6.74	8.53	8.79	7.81	8.06	1.21
A_09_P208950	FBtr0074895	40177	Grasp65	13.17	13.53	13.33	13.56	12.61	12.46	12.84	13.04	-0.66
A_09_P209345	FBtr0333898	38352	Shab	5.62	5.10	5.30	5.31	5.81	6.22	6.09	6.35	0.78
A_09_P209390	FBtr0302599	38017	Pdk1	5.16	4.69	4.25	4.60	6.62	7.63	6.28	6.89	2.18
A_09_P209420	FBtr0330419	39533	dysc	4.80	3.97	4.47	3.23	6.00	5.86	5.74	5.88	1.75
A_09_P209445	FBtr0303231	5740165	CG34427	8.35	8.06	8.58	8.27	5.96	6.09	7.63	7.89	-1.42
A_09_P209670	FBtr0289953	40896	alpha-Est10	7.20	7.29	7.15	7.18	6.56	6.24	6.00	6.28	-0.94
A_09_P209905	FBtr0075464	39784	CG13068	10.73	10.63	10.59	10.55	9.97	10.07	9.94	9.70	-0.70
A_09_P209925	AJ459772	2768674	Fancd2	6.36	6.58	6.19	6.37	3.86	4.31	3.69	4.71	-2.23
A_09_P209935	FBtr0335397	32353	mamo	8.02	8.96	8.66	8.75	7.12	7.80	7.04	7.20	-1.31
A_09_P209950	FBtr0344083	326250	CG34148	8.80	8.77	8.82	8.81	8.31	8.21	8.41	8.43	-0.46

A_09_P210220	FBtr0305649	2768969	CG33267	5.13	4.36	4.36	4.18	6.58	6.78	6.28	5.80	1.85
A_09_P210235	FBtr0301588	42515	SNF4Agamma	8.97	9.43	9.22	9.13	10.05	10.21	9.73	9.59	0.71
A_09_P210405	FBtr0113401	318653	CG31269	9.97	10.16	10.34	10.33	8.58	8.39	8.52	8.43	-1.72
A_09_P210520	FBtr0112561	5740672	CG34351	7.03	6.30	6.35	6.12	7.55	7.41	7.21	7.23	0.90
A_09_P210545	FBtr0343421	34073	CG14535	6.78	6.34	6.10	5.97	6.91	7.14	7.21	7.18	0.81
A_09_P210615	FBtr0112560	35904	Np	7.46	4.12	2.88	3.90	NA	NA	8.56	9.52	4.45
A_09_P210650	FBtr0112645	37398	CG34396	7.35	7.67	7.41	7.28	6.33	5.13	5.70	6.53	-1.50
A_09_P210875	FBtr0290138	43136	CG42235	10.28	10.82	10.73	10.67	10.14	10.01	10.08	10.05	-0.56
A_09_P210885	FBtr0112563	5740590	CG34353	7.46	8.21	7.35	8.23	9.32	9.37	8.97	8.69	1.27
A_09_P211220	FBtr0085751	43666	CG15550	4.23	3.74	4.26	4.44	5.68	5.32	5.26	5.71	1.33
A_09_P211320	FBtr0300553	50320	dpr12	8.07	8.33	8.27	7.73	9.18	9.46	8.74	9.22	1.05
A_09_P211330	FBtr0300411	40264	CG5910	9.49	9.40	9.46	9.42	10.97	10.79	10.46	9.98	1.11
A_09_P211360	FBtr0085062	43229	His2Av	8.61	8.59	8.78	8.52	7.61	7.75	8.10	8.32	-0.68
A_09_P211370	NR_002465	3772408	snoRNA:U3:9B	8.65	8.92	8.12	8.49	9.41	9.40	9.26	9.53	0.86
A_09_P211630	FBtr0333602	31826	rdgA	8.85	9.33	8.93	9.01	9.84	10.18	9.50	9.57	0.74
A_09_P211845	FBtr0085473	43503	CG7598	10.65	10.49	10.61	10.56	10.24	10.30	10.21	10.20	-0.34
A_09_P211865	FBtr0073414	32009	Imp	8.85	8.80	8.31	8.10	9.41	9.69	9.08	9.78	0.97
A_09_P211880	FBtr0330051	42177	Ssdp	9.34	9.81	9.46	9.53	9.00	9.07	9.01	9.13	-0.48
A_09_P212010	FBtr0088883	35746	rnh1	11.28	11.32	11.26	11.38	11.23	11.13	11.16	11.16	-0.14
A_09_P212030	FBtr0302598	38433	Eip63E	7.07	6.92	6.84	6.70	7.57	7.74	7.58	7.52	0.72
A_09_P212135	FBtr0083755	42318	CG3734	12.52	11.18	11.61	11.14	13.10	12.71	12.77	12.60	1.18
A_09_P212225	FBtr0303465	37644	CG3788	8.04	9.12	9.01	9.54	6.91	6.89	7.23	7.54	-1.79
A_09_P212330	FBtr0072412	37988	CG2811	8.01	8.58	7.92	8.51	9.60	9.66	9.09	8.72	1.01
A_09_P212350	FBtr0070707	31405	ctp	9.39	9.22	9.08	9.36	9.45	9.52	9.56	9.61	0.27
A_09_P212380	FBtr0072889	38301	CG8993	12.87	12.71	12.76	12.85	12.24	12.32	12.46	12.50	-0.42
A_09_P212390	BP548557	42851	CG6000	11.17	10.68	11.00	10.73	9.26	9.16	9.75	9.99	-1.35
A_09_P212540	FBtr0075875	39476	CG11267	10.61	10.28	10.46	10.28	9.83	9.82	9.95	10.09	-0.49
A_09_P212580	FBtr0086215	246487	CG30154	7.68	8.93	8.42	8.87	7.06	7.65	6.91	7.26	-1.25
A_09_P212600	FBtr0070408	31217	CG2865	6.15	5.27	5.47	4.36	7.24	7.33	6.55	6.58	1.61
A_09_P212760	FBtr0072173	37846	eIF-5A	9.93	10.20	10.30	10.18	9.50	9.35	9.47	9.62	-0.67
A_09_P213065	FBtr0333447	38331	Aats-pro	10.52	10.46	10.35	10.37	9.34	9.49	9.63	10.08	-0.78

A_09_P213110	FBtr0331437	33423	CG9866	9.63	10.00	9.95	9.90	9.43	9.52	9.37	9.60	**-0.39**
A_09_P213215	FBtr0331617	35300	Kua	9.49	9.73	9.53	9.73	10.64	10.56	10.43	10.50	**0.91**
A_09_P213495	FBtr0083589	42203	PKD	8.63	9.06	8.78	8.89	9.16	9.62	9.80	9.97	**0.80**
A_09_P213650	FBtr0332867	40019	AlCR2	6.43	5.75	5.62	5.32	7.42	6.97	7.12	6.69	**1.27**
A_09_P213775	FBtr0072275	37879	CG30419	7.93	7.72	7.60	7.48	8.39	8.55	8.32	8.49	**0.75**
A_09_P213790	FBtr0078454	40420	CG14567	8.90	8.28	8.31	8.66	12.47	12.11	11.60	10.48	**3.13**
A_09_P213875	FBtr0072024	37710	CG9815	7.51	7.12	6.95	7.04	8.26	8.14	7.89	7.66	**0.83**
A_09_P213960	FBtr0112766	5740316	CG34460	6.69	6.69	6.66	6.30	3.93	4.14	4.04	5.14	**-2.27**
A_09_P214085	FBtr0300399	43184	CG6154	6.59	5.89	5.64	5.38	6.80	7.10	6.77	6.79	**0.99**
A_09_P214355	FBtr0301141	39026	CG5026	8.52	9.14	8.96	9.09	7.81	8.34	8.13	8.23	**-0.80**
A_09_P214365	FBtr0100033	37953	CG33988	8.98	8.76	8.98	8.73	9.58	10.14	9.58	9.72	**0.89**
A_09_P214780	FBtr0306253	48844	l(2)tid	11.45	11.29	11.43	11.26	11.25	11.16	11.10	11.15	**-0.19**
A_09_P214875	FBtr0076500	39065	UGP	12.81	13.27	13.00	13.23	11.66	11.54	12.18	12.63	**-1.08**
A_09_P214965	FBtr0309221	31940	CG15312	8.27	8.31	8.37	8.42	8.82	9.20	8.78	8.72	**0.54**
A_09_P215080	FBtr0333601	31826	rdgA	8.78	9.29	8.74	8.98	9.92	10.26	9.57	9.60	**0.89**
A_09_P215095	FBtr0091493	3346192	Vmat	10.10	10.05	10.00	9.84	10.90	11.15	10.63	11.05	**0.94**
A_09_P215145	FBtr0306850	38058	mth	9.35	10.17	9.66	9.78	8.46	9.10	9.06	9.13	**-0.80**
A_09_P215210	FBtr0339144	326115	CG31033	9.08	9.36	9.22	9.47	8.67	8.47	8.65	8.87	**-0.62**
A_09_P215295	FBtr0336864	34892	Pol32	8.83	8.88	8.87	8.81	8.73	8.71	8.75	8.66	**-0.14**
A_09_P215370	FBtr0071682	37468	pirk	11.12	9.85	10.58	10.41	12.99	12.09	12.30	11.95	**1.85**
A_09_P215395	FBtr0299780	3771738	CG9510	11.98	12.65	12.39	12.66	11.34	11.28	11.56	11.71	**-0.95**
A_09_P215690	FBtr0079788	34215	C1GalTA	8.59	8.50	8.71	8.67	9.13	9.02	8.83	8.78	**0.32**
A_09_P215705	FBtr0331579	5740442	CG34356	7.15	6.97	6.39	6.38	7.39	7.68	7.51	7.88	**0.89**
A_09_P215760	FBtr0085802	43724	CycG	8.01	8.57	8.26	8.21	7.88	7.72	7.69	7.85	**-0.48**
A_09_P215775	FBtr0085000	43183	Ald	6.10	6.62	6.97	6.67	5.51	5.15	5.51	6.02	**-1.04**
A_09_P215835	FBtr0076949	38750	CG10103	11.02	11.12	11.05	11.24	12.05	12.00	11.64	11.33	**0.65**
A_09_P216020	FBtr0113773	3355155	CG40002	9.71	9.65	9.57	9.58	10.49	10.15	10.53	10.55	**0.80**
A_09_P216215	FBtr0088829	35726	CG12825	13.14	13.10	13.01	13.08	13.97	13.68	13.77	13.51	**0.65**
A_09_P216220	FBtr0339129	246580	CG30381	7.41	7.45	7.39	7.39	6.51	6.67	7.06	7.22	**-0.55**
A_09_P216260	FBtr0113060	35987	CG12929	9.20	9.33	9.20	9.28	9.02	8.96	9.00	8.73	**-0.32**
A_09_P216275	FBtr0088533	246548	CG30338	10.91	10.66	10.75	10.66	10.27	10.23	10.45	10.60	**-0.36**

A_09_P216295	FBtr0088247	36143	CG12343	11.01	11.01	11.06	11.08	11.33	11.15	11.25	11.31	0.22
A_09_P216440	FBtr0086902	36995	CG14483	11.37	11.12	11.15	11.52	10.56	10.54	10.85	10.90	-0.57
A_09_P216450	FBtr0112627	5740303	CG34386	7.44	6.48	6.63	6.54	8.33	7.95	7.74	7.48	1.10
A_09_P216475	FBtr0086482	50203	CG15127	9.96	9.37	9.83	9.68	10.87	10.82	10.55	10.33	0.93
A_09_P216515	FBtr0071628	37413	LSm1	10.92	10.75	10.87	10.81	10.18	10.05	10.46	10.56	-0.53
A_09_P216565	FBtr0071835	37572	CG6018	9.14	9.03	9.00	8.92	10.47	10.11	10.11	9.87	1.12
A_09_P216575	FBtr0302770	37643	CG42678	8.31	8.70	8.65	8.61	7.51	7.31	7.38	7.48	-1.15
A_09_P216605	FBtr0072190	37839	CG13567	9.38	9.17	9.15	9.27	8.96	8.93	8.92	9.05	-0.28
A_09_P216655	FBtr0070342	31167	CG4325	7.02	8.92	7.84	8.80	5.53	5.49	5.48	5.19	-2.72
A_09_P216710	FBtr0071397	31919	CG15247	11.36	11.77	11.96	12.06	11.13	11.03	11.15	11.14	-0.67
A_09_P216740	FBtr0073473	32051	CG15199	11.33	11.02	11.04	11.13	9.50	9.63	9.30	9.08	-1.75
A_09_P216750	FBtr0112979	32127	CG15221	9.52	9.79	10.05	10.16	9.53	9.20	9.09	8.79	-0.72
A_09_P216790	FBtr0073840	32337	Pdcd4	12.38	13.17	12.80	13.35	14.04	14.11	13.85	13.72	1.00
A_09_P216910	FBtr0335172	33446	CG15394	7.63	6.92	7.02	7.00	9.50	9.62	9.25	9.12	2.23
A_09_P217025	FBtr0113031	34306	CG4537	8.00	7.86	7.86	8.02	6.92	7.12	7.48	7.50	-0.68
A_09_P217030	FBtr0079895	318908	Apf	10.14	10.17	10.35	10.38	9.89	9.89	9.99	10.11	-0.29
A_09_P217095	FBtr0080586	34815	nimB5	9.03	9.14	9.06	8.74	7.38	7.69	7.95	8.32	-1.16
A_09_P217215	FBtr0073038	38386	CG14957	12.07	12.26	12.35	12.43	12.53	12.81	12.78	12.82	0.46
A_09_P217250	FBtr0073253	38509	Cpr64Ab	8.08	NA	8.97	NA	2.98	3.50	6.01	6.65	-3.74
A_09_P217305	FBtr0076914	38783	CG8628	15.76	15.27	15.75	15.45	14.79	14.98	14.90	14.65	-0.73
A_09_P217345	FBtr0076592	50301	CG13306	7.24	7.02	7.06	6.98	6.00	5.21	6.60	6.65	-0.96
A_09_P217360	FBtr0091930	39092	CG33926	8.26	8.73	8.18	8.84	10.76	10.55	10.50	10.27	2.02
A_09_P217400	FBtr0113334	50290	CG14132	10.28	10.04	10.16	10.21	11.51	11.90	11.48	11.60	1.45
A_09_P217535	FBtr0078484	40450	CG7130	11.21	12.36	11.97	12.44	10.95	10.43	10.61	10.50	-1.37
A_09_P217580	FBtr0078848	40611	CG14661	8.68	9.44	9.10	9.35	7.49	7.76	7.65	7.84	-1.46
A_09_P217650	FBtr0082077	41165	CG8319	9.23	9.24	9.06	9.06	7.66	7.88	7.95	7.91	-1.30
A_09_P217680	FBtr0082152	41214	CG9458	9.72	10.19	10.15	10.18	8.00	8.77	8.81	8.89	-1.44
A_09_P217720	FBtr0082318	318700	CG31373	11.45	12.37	12.15	12.44	10.70	10.49	10.31	10.07	-1.71
A_09_P217730	FBtr0113217	41304	CG6567	11.40	11.18	11.39	11.34	10.80	10.74	11.06	10.89	-0.46
A_09_P217735	FBtr0082298	41307	CG14694	7.37	6.82	6.56	5.88	8.91	8.85	8.27	7.78	1.79
A_09_P217775	FBtr0113224	41459	CG3532	8.12	8.46	8.23	8.29	7.93	7.96	7.86	7.87	-0.37

A_09_P217825	FBtr0083113	41872	CG6171	7.28	7.23	6.99	7.25	7.65	7.90	7.73	7.73	0.56
A_09_P217845	FBtr0083253	41963	CG8925	7.09	7.02	6.82	6.24	8.01	7.78	7.73	7.42	0.94
A_09_P217855	FBtr0083314	41993	CG14894	12.31	12.45	12.41	12.47	11.96	11.98	12.06	12.00	-0.41
A_09_P217870	FBtr0083418	42067	CG17477	9.26	9.77	9.81	9.96	8.38	8.71	8.31	8.05	-1.33
A_09_P217930	FBtr0083711	42281	CG14285	12.15	12.24	12.33	12.29	10.34	10.23	10.83	11.18	-1.61
A_09_P217950	FBtr0083858	42363	CG17186	8.33	8.53	8.57	8.36	8.03	8.20	8.09	8.15	-0.33
A_09_P217970	FBtr0083894	42421	CG4335	11.07	11.49	11.46	11.55	10.21	10.45	10.79	11.11	-0.76
A_09_P218005	FBtr0112609	42638	CG34376	8.60	8.18	8.26	8.20	9.31	9.04	9.19	8.92	0.81
A_09_P218025	FBtr0084319	42699	CG4725	9.62	10.41	9.99	10.23	7.16	7.32	8.56	9.17	-2.01
A_09_P218030	FBtr0084318	42700	CG4723	8.69	9.88	9.27	9.74	5.60	6.75	7.35	7.77	-2.53
A_09_P218120	FBtr0084790	318595	CG31111	9.31	8.90	9.10	9.00	8.54	8.33	8.69	8.77	-0.49
A_09_P218200	FBtr0085255	43356	CG5527	10.11	9.26	9.71	9.21	10.42	10.30	10.34	10.26	0.75
A_09_P218220	FBtr0085407	43456	Mesh1	9.61	9.54	9.51	9.54	8.35	8.50	8.85	9.26	-0.81
A_09_P218235	FBtr0085470	318567	CG31041	10.52	9.04	9.91	9.66	14.04	13.46	13.38	12.48	3.56
A_09_P218250	FBtr0085574	43588	CG1983	9.42	9.39	9.61	9.44	9.16	8.89	8.98	8.77	-0.51
A_09_P218290	FBtr0332962	31217	CG2865	10.68	9.85	10.41	10.10	11.85	12.14	11.58	11.18	1.43
A_09_P218395	FBtr0077179	38598	CG10672	13.71	13.46	13.50	13.53	13.70	13.89	13.89	14.06	0.33
A_09_P218455	FBtr0075560	39683	AGO2	11.30	11.82	11.53	11.71	12.26	12.08	11.97	12.08	0.50
A_09_P218520	FBtr0075824	39515	CG10116	11.56	11.33	11.30	11.41	11.99	12.22	11.83	11.64	0.52
A_09_P218565	FBtr0112809	45318	kn	6.97	6.59	6.83	6.64	7.82	7.64	7.50	7.17	0.77
A_09_P218590	AJ252068	44002	Sras	8.94	8.61	8.80	8.61	9.41	9.32	9.32	9.20	0.57
A_09_P218610	FBtr0087686	36506	CG6145	11.28	10.86	11.14	10.88	11.86	12.39	11.93	11.57	0.90
A_09_P218615	FBtr0083004	41809	CG3837	7.77	8.04	7.82	7.96	6.89	6.59	6.82	7.07	-1.06
A_09_P218765	FBtr0075050	40034	GNBP1	10.05	10.79	10.54	10.76	9.55	9.80	9.83	10.08	-0.72
A_09_P218780	L08845	39866	Dab	9.26	9.84	9.65	9.83	10.75	11.04	10.58	10.49	1.07
A_09_P218875	FBtr0300555	43787	PMCA	11.69	11.65	11.92	11.71	11.30	10.97	11.19	11.17	-0.58
A_09_P219220	FBtr0308785	37254	sm	7.30	7.44	6.90	7.09	7.66	7.83	7.79	7.81	0.59
A_09_P219335	FBtr0087745	36468	Mp20	10.02	10.79	10.75	10.75	9.36	8.44	9.19	9.31	-1.50
A_09_P219460	FBtr0085614	43597	CecA2	4.46	6.40	3.64	3.91	13.25	11.73	12.24	11.43	7.56
A_09_P219490	FBtr0290328	43213	beat-VII	7.42	7.00	6.94	6.71	8.07	8.15	8.15	7.97	1.07
A_09_P219495	FBtr0340417	32565	CG9172	9.87	9.89	9.83	9.88	9.41	9.08	9.30	9.44	-0.56

A_09_P219520	FBtr0306327	40220	CG17233	8.28	8.16	8.24	8.21	7.11	7.62	7.38	7.47	-0.83
A_09_P219550	FBtr0075699	39611	bmm	8.87	8.68	8.34	8.83	10.16	9.76	9.75	9.58	1.13
A_09_P219585	FBtr0309054	39054	Rdl	6.50	5.72	5.54	5.60	7.14	7.31	7.27	7.60	1.49
A_09_P219640	FBtr0301126	40221	Clc	9.18	9.27	9.47	9.12	8.89	8.51	8.85	9.01	-0.45
A_09_P219665	FBtr0330419	39533	dysc	5.97	5.29	5.18	5.18	6.86	7.49	6.83	7.23	1.70
A_09_P219720	FBtr0334162	33461	Pgk	15.07	15.05	15.20	15.21	13.76	13.94	14.36	14.70	-0.94
A_09_P219730	FBtr0301661	41550	Vha55	12.24	12.28	12.28	12.18	12.12	11.62	11.71	11.55	-0.50
A_09_P219900	FBtr0086864	37021	CG4996	7.57	7.47	7.52	7.38	7.12	7.16	7.04	7.22	-0.35
A_09_P220180	FBtr0337091	34985	CG5953	6.87	7.40	6.44	6.88	9.92	10.20	9.33	8.46	2.58
A_09_P220360	FBtr0075045	40073	CG18135	7.33	7.98	7.51	7.62	6.52	7.06	6.36	6.95	-0.89
A_09_P220485	FBtr0078422	40402	CG11306	11.52	11.82	11.63	11.79	10.94	10.87	11.13	11.19	-0.66
A_09_P220880	FBtr0073776	32296	CG1662	8.29	8.37	8.34	8.18	7.74	8.07	7.60	7.66	-0.53
A_09_P220980	FBtr0304871	43775	Crk	11.97	12.25	11.99	12.22	11.82	11.49	11.71	11.83	-0.39
A_09_P220985	FBtr0073379	32013	CG17333	10.68	10.60	10.62	10.54	9.94	9.61	10.07	10.21	-0.66
A_09_P221035	FBtr0076093	39284	chrb	8.61	8.73	8.46	8.21	9.90	9.97	9.42	9.54	1.21
A_09_P221165	FBtr0332962	31217	CG2865	8.82	7.90	8.31	7.77	9.81	9.91	9.38	9.15	1.36
A_09_P221220	FBtr0073499	32090	HP5	7.12	7.29	7.18	7.26	6.57	6.84	6.68	6.69	-0.52
A_09_P221320	FBtr0331603	31456	SK	8.43	7.92	8.02	7.99	8.54	8.71	8.68	8.41	0.50
A_09_P221415	FBtr0113827	3354930	CG40191	10.81	10.88	10.59	10.71	10.06	9.46	9.98	10.28	-0.80
A_09_P221650	FBtr0085785	43707	CG1607	9.13	9.20	9.34	9.36	8.39	9.04	8.69	8.58	-0.58
A_09_P221685	FBtr0083947	42435	Elongin-B	12.97	12.94	13.15	13.04	12.41	12.37	12.59	12.57	-0.54
A_09_P221730	FBtr0331514	39018	orb2	7.95	7.77	7.60	7.30	8.28	8.19	8.11	8.35	0.58
A_09_P221795	FBtr0304018	39873	Nrt	8.77	9.09	9.02	9.12	8.80	8.71	8.61	8.63	-0.31
A_09_P221830	FBtr0073322	317935	CG32243	8.64	8.74	8.71	8.58	8.06	7.66	8.32	8.49	-0.54
A_09_P221855	FBtr0081611	40852	CG10068	6.67	6.60	6.50	6.14	5.48	5.14	5.31	5.82	-1.04
A_09_P221890	FBtr0301340	43814	bt	10.91	11.66	11.81	11.79	9.96	9.91	10.16	10.30	-1.46
A_09_P221960	FBtr0076669	44291	Idh	12.73	12.64	12.67	12.55	11.87	11.26	12.12	12.41	-0.73
A_09_P222055	FBtr0089224	43832	plexA	9.00	9.31	9.16	9.28	8.66	9.00	8.74	8.82	-0.38
A_09_P222225	FBtr0089233	43836	CG32016	8.86	8.72	8.91	8.84	8.35	8.12	8.32	8.27	-0.57
A_09_P222305	FBtr0076194	39227	CG14147	11.22	NA	11.58	NA	4.77	4.89	9.27	10.13	-4.13
A_09_P222330	FBtr0080230	34542	Reps	8.86	9.10	8.97	8.92	8.26	8.59	8.51	8.58	-0.48

A_09_P222575	FBtr0089960	41852	Tm1	15.48	15.70	15.87	15.69	15.06	14.63	15.01	15.19	-0.71
A_09_P222590	FBtr0075161	39986	CG5567	10.81	10.72	10.89	10.55	10.03	9.28	10.19	10.40	-0.77
A_09_P222640	FBtr0074146	32566	eIF5	14.09	14.20	14.30	14.27	13.79	14.06	13.96	14.06	-0.25
A_09_P222655	FBtr0111248	3355129	CG17715	9.19	9.03	9.18	9.18	8.58	8.42	8.61	8.83	-0.53
A_09_P222720	FBtr0307879	31955	Hk	6.57	6.24	5.73	5.67	7.14	7.16	7.11	6.97	1.04
A_09_P222790	FBtr0332421	36200	CG13220	11.73	11.52	11.61	11.52	10.49	10.08	10.74	10.94	-1.03
A_09_P223095	FBtr0310541	43810	CG11360	6.95	7.09	6.91	6.90	5.88	6.07	6.59	6.54	-0.70
A_09_P223190	FBtr0303159	326255	4EHP	9.27	9.12	9.15	9.13	9.47	9.64	9.40	9.68	0.38
A_09_P223200	FBtr0076161	39260	CG7616	7.34	7.05	7.01	6.90	6.55	6.57	6.49	6.63	-0.52
A_09_P223440	FBtr0333484	46015	tutl	9.67	10.01	9.96	9.69	10.62	10.88	10.41	10.56	0.78
A_09_P223498	FBtr0302182	32156	Sclp	9.94	9.70	9.97	9.56	8.92	8.26	8.94	8.98	-1.02
A_09_P223520	FBtr0302659	31136	CG42666	8.42	7.93	7.72	7.81	10.18	10.18	9.54	8.99	1.76
A_09_P223605	FBtr0089204	43819	pho	8.71	8.98	8.99	8.87	8.59	8.31	8.44	8.41	-0.45
A_09_P223610	FBtr0070993	31597	CG3226	13.46	13.58	13.61	13.64	12.02	12.11	12.93	13.32	-0.98
A_09_P223880	FBtr0308694	32245	fne	8.86	8.04	8.07	8.05	9.30	9.58	9.41	9.65	1.23
A_09_P223950	FBtr0305694	41788	jvl	8.44	9.06	8.56	8.67	7.59	7.74	7.19	7.64	-1.14
A_09_P224090	FBtr0080580	318958	CG31814	4.52	3.54	4.12	4.17	6.08	6.42	5.63	5.68	1.86
A_09_P224170	FBtr0079043	33721	Rtnl1	13.33	13.77	13.57	13.58	12.52	12.06	12.62	12.76	-1.07
A_09_P224180	FBtr0079700	34181	Dh31	8.81	8.02	8.47	7.99	9.99	9.63	9.50	9.36	1.30
A_09_P224190	FBtr0331711	32337	Pdcd4	9.09	9.78	9.42	9.90	10.60	10.68	10.42	10.20	0.93
A_09_P224310	FBtr0073662	32205	CG11138	2.56	2.65	2.79	3.84	5.46	5.26	5.03	4.70	2.15
A_09_P224460	NR_037747	10178962	CR42860	7.20	7.30	7.31	7.28	5.74	5.31	6.55	6.48	-1.25
A_09_P224480	FBtr0100182	36059	14-3-3zeta	12.23	12.01	12.12	12.14	11.87	11.13	11.71	11.60	-0.55
A_09_P224725	FBtr0076168	39270	scyl	8.61	8.56	7.94	8.28	11.08	10.91	10.21	10.22	2.25
A_09_P224820	FBtr0078805	40639	Prosbeta7	13.29	13.36	13.29	13.24	12.44	12.31	12.84	13.16	-0.61
A_09_P224875	FBtr0089747	43587	Mlc2	16.02	16.17	16.45	16.24	15.55	14.96	15.56	15.52	-0.82
A_09_P224941	FBtr0084982	43152	(spl)mbeta-HL	9.12	9.10	8.95	8.88	9.57	9.55	9.42	9.69	0.54
A_09_P224980	FBtr0310057	37384	RIC-3	10.25	10.67	10.65	10.79	9.88	9.67	9.67	9.70	-0.86
A_09_P225345	FBtr0310057	37384	RIC-3	10.34	10.87	10.81	10.98	10.11	9.84	9.99	9.95	-0.78
A_09_P225405	FBtr0333700	43824	Cals	11.94	11.78	11.93	11.76	11.02	10.82	11.44	11.40	-0.68
A_09_P225860	FBtr0091446	3346177	CG33506	12.15	11.84	11.96	11.83	11.32	10.91	11.39	11.52	-0.66

A_09_P226175	FBtr0085224	43334	Ets98B	9.24	9.38	9.09	9.42	9.54	10.10	9.78	9.79	**0.52**
A_09_P226250	FBtr0299929	36002	sqa	10.12	10.74	10.08	10.54	11.60	11.69	11.12	11.00	**0.98**
A_09_P226300	FBtr0299768	42455	CG42322	7.84	7.44	7.37	7.23	8.46	8.79	8.66	8.60	**1.16**
A_09_P226440	FBtr0085877	53554	krz	6.83	6.78	6.85	6.25	5.35	5.93	6.12	5.76	**-0.88**
